# Genome-Wide Differentiation of Various Melon Horticultural Groups for Use in GWAS for Fruit Firmness and Construction of a High Resolution Genetic Map

**DOI:** 10.3389/fpls.2016.01437

**Published:** 2016-09-22

**Authors:** Padma Nimmakayala, Yan R. Tomason, Venkata L. Abburi, Alejandra Alvarado, Thangasamy Saminathan, Venkata G. Vajja, Germania Salazar, Girish K. Panicker, Amnon Levi, William P. Wechter, James D. McCreight, Abraham B. Korol, Yefim Ronin, Jordi Garcia-Mas, Umesh K. Reddy

**Affiliations:** ^1^Gus R. Douglass Institute and Department of Biology, West Virginia State UniversityInstitute, WV, USA; ^2^Department of Selection and Seed Production, Dnepropetrovsk State Agrarian and Economic UniversityDnepropetrovsk, Ukraine; ^3^Department of Agriculture, Alcorn State UniversityLorman, MS, USA; ^4^U.S. Vegetable Laboratory, United States Department of Agriculture, Agricultural Research ServiceCharleston, SC, USA; ^5^U.S. Agricultural Research StationSalinas, CA, USA; ^6^Department of Evolutionary and Environmental Biology, Haifa UniversityHaifa, Israel; ^7^Centre for Research in Agricultural Genomics, Consejo Superior de Investigaciones Científicas-Institute for Food and Agricultural Research and Technology-Universitat Autònoma de Barcelona-Universitat de BarcelonaBarcelona, Spain

**Keywords:** genotyping-by-sequencing, high-resolution genetic map, linkage disequilibrium, melon, GWAS, fruit firmness

## Abstract

Melon (*Cucumis melo* L.) is a phenotypically diverse eudicot diploid (2*n* = 2*x* = 24) has climacteric and non-climacteric morphotypes and show wide variation for fruit firmness, an important trait for transportation and shelf life. We generated 13,789 SNP markers using genotyping-by-sequencing (GBS) and anchored them to chromosomes to understand genome-wide fixation indices (*Fst*) between various melon morphotypes and genomewide linkage disequilibrium (LD) decay. The *F*_*ST*_ between accessions of *cantalupensis* and *inodorus* was 0.23. The *F*_*ST*_ between *cantalupensis* and various *agrestis* accessions was in a range of 0.19–0.53 and between *inodorus* and *agrestis* accessions was in a range of 0.21–0.59 indicating sporadic to wide ranging introgression. The EM (Expectation Maximization) algorithm was used for estimation of 1436 haplotypes. Average genome-wide LD decay for the melon genome was noted to be 9.27 Kb. In the current research, we focused on the genome-wide divergence underlying diverse melon horticultural groups. A high-resolution genetic map with 7153 loci was constructed. Genome-wide segregation distortion and recombination rate across various chromosomes were characterized. Melon has climacteric and non-climacteric morphotypes and wide variation for fruit firmness, a very important trait for transportation and shelf life. Various levels of QTLs were identified with high to moderate stringency and linked to fruit firmness using both genome-wide association study (GWAS) and biparental mapping. Gene annotation revealed some of the SNPs are located in β-D-xylosidase, glyoxysomal malate synthase, chloroplastic anthranilate phosphoribosyltransferase, and histidine kinase, the genes that were previously characterized for fruit ripening and softening in other crops.

## Introduction

Melon (*Cucumis melo* L.) is a phenotypically diverse eudicot diploid (2*n* = 2*x* = 12) which originated in Asia (Silberstein et al., 2003). According to the morphological observations of Jeffrey (1980) and Stepansky et al. (1999), varieties (vars.) *cantalupensis* (cantaloupe) and *inodorus* (honeydew) should be placed in subspecies *melo* and vars. *momordica, conomon, dudaim*, and *chito* in subspecies *agrestis* (Decker-Walters et al., 2002). Pitrat (2008) grouped melons into 15 widely accepted horticultural groups (*cantalupensis, reticulatus, adana, chandalak, ameri, inodorus, chate, flexuosus, dudaim* and *tibish* (in subsp. *melo*), and *momordica, conomon, chinensis, makuwa*, and *acidulous* in subsp. *agrestis*). Consumer demand for sweet melons has stimulated the selection and breeding of hundreds of cultivars belonging to numerous market types, with local, regional, and international distribution (Paris et al., 2012). Domestication of melons has not been intensively studied, the genetic control of domestication traits, and subsequent diversification and selection processes that led to various melon morphotypes is still poorly understood. A genome-wide sequence of melon of size 375 Mb (83.3% of estimated size) has been made available. This genome has enabled an exhaustive phylogenetic comparison of the melon genome with cucumber (Garcia-Mas et al., 2012). SNP discovery in diverse melon botanical groups will allow marker-anchoring to the whole genome sequence (WGS), thus giving researchers a better understanding of the genetic control of domestication and diversification as shown in the study of Argyris et al. (2015).

Advances in next-generation sequencing technologies have driven the costs of DNA sequencing down to the point that genotyping-by-sequencing (GBS) is now feasible for highly diverse species such as melons. This approach involves reduced-representation sequencing of multiplexed samples and is simple, quick, extremely specific, highly reproducible, and may reach important regions of the genome that are inaccessible to sequence capture approaches (Elshire et al., 2011; Poland and Rife, 2012). The flexibility and low cost of GBS makes this an excellent tool for building high density genetic maps and for use in genome-wide association studies (GWAS) (Poland et al., 2012; Nimmakayala et al., 2014; Reddy et al., 2014). A detailed understanding of population structure and linkage disequilibrium (LD) is paramount for association mapping of the QTLs that underlie various complex traits (Flint-Garcia et al., 2003; Wang et al., 2013; Rincent et al., 2014). The distribution pattern of LD across the genome directly depends on evolutionary forces such as genetic drift, population structure, levels of inbreeding across the genome, and map regions contributing genetic differentiation among the subpopulations. Emergence and maintenance of LD is based on these evolutionary forces and the associated pattern of selection (Ersoz et al., 2007). Esteras et al. (2013) developed a genotyping array for 768 SNPs from a collection of 74 melon accessions with the Illumina GoldenGate technology (Illumina Inc., San Diego, CA), identifying relatively low LD in melons. It is very important to precisely characterize LD blocks across various chromosomes for GWAS studies in diverse horticultural groups such as melon.

Melon is an important desert fruit with tremendous diversity that is a product of consumer preferences from different countries, ecologies, and cultures (Tomason et al., 2013). Understanding divergence and adaptation that underlie the formation of various morphotypes is very important to the development of disease resistant and high quality melons. Fruit quality is related to both internal variables such as fruit firmness, sugar content, acid content, and external variables including fruit shape, size, and texture (García-Ramos et al., 2005). Fruit firmness affects the quality of melon fruit, shelf life, and the ability to transport the fruit over long distances (Moreno et al., 2008; Dahmani-Mardas et al., 2010). Moreno et al. (2008) mapped important QTLs in the locations of candidate genes involved in ethylene regulation, biosynthesis and cell wall degradation using near-isogenic lines (NILs) derived from the non-climacteric melon parental lines PI 161375 and “Piel de Sapo.” Périn et al. (2002) performed genetic analysis for non-climacteric phenotype in fruit tissues on a population of recombinant cantaloupe Charentais × PI 161375 inbred lines to identify several QTLs for ethylene regulation. Dahmani-Mardas et al. (2010) constructed a mutant collection of 4023 melon M2 TILLING families to screen for 11 genes, of which four genes were involved in ethylene/fruit firmness/fruit ripening and identified a mutant for fruit firmness in the TILLING platform.

The current study is to resolve the genetic diversity and relatedness of melon germplasm with the melons of Asia and the western hemisphere using high density SNPs mapped to various chromosomes. Our objective is to compare LD across the chromosomes and to perform GWAS for fruit firmness. Other objectives of the current study were to construct a high-density genetic map for validation of QTLs and to understand genomic features such as colinearity with the publicly available melon genome sequence.

## Materials and methods

One hundred and twenty accessions of various melon horticultural groups representing a world-wide distribution were used for field evaluation. For generation of SNPs, 97 of the most diverse Plant Introductions from our collection were selected based on SSR data representing the botanical groups *cantalupensis* (51), *inodorus* (13), *reticulatus* (5), *ameri* (3), *dudaim* (6), *flexuosus* (5), *conomon* (9), *makuwa* (3), *acidulus* (1), and *momordica* (1) (Table S1). These PIs were self-pollinated, and the progeny were tested for two seasons (2013–2014) as three replications in two locations (Agriculture Experimental Station, West Virginia State University, Institute, WV and Alcorn State University, Lorman, MS) using a row to plant spacing of 180 × 70 cm. Ten plants per accession were grown per replication. Data were collected pertaining to fruit firmness by testing pressure in kg/cm2 were measured at full maturity using a FT 011 penetrometer (Model# FT 011, Effigy, Alfonsino, Italy) (Table S2). A cross was made between MR-1 (*momordica*) and “Hale's Best Jumbo” (P2), a western shipper cantaloupe. For building a genetic map, 103 F_2_ progeny were generated from a single F_1_ plant of P1 × P2 cross. Fruits of F_2_ progeny were grown in greenhouse (Table S3). Fruit firmness was measured as pressure to compress (kg/cm2) using a FT 011 penetrometer employing a 0.15 cm tip.

### SNP discovery by GBS

Genomic DNA isolation from the seedlings involved the DNeasy plant mini kit (QIAGEN, Germany), and GBS was as described (Elshire et al., 2011; Reddy et al., 2014). DNA was treated with the restriction enzyme *Ape*KI, a type II restriction endonuclease, barcoded by accession, and sequenced on an Illumina HiSeq 2500 as described (Elshire et al., 2011). SNPs were extracted using TASSEL-GBS Discovery/Production pipeline (https://bitbucket.org/tasseladmin/tassel-5-source/wiki/Tassel5GBSv2Pipeline). Chromosomal assignment and position of SNPs on the physical map was deduced from the WGS draft of melon (version V3.5) at http://www.melonomics.net. SNPs are designated based on chromosome number and position (e.g., S10_172735351 meaning SNP located at 172735351th position on chromosome 10).

### Population structure analysis and divergence

Genetic diversity values were calculated by a neighbor-joining algorithm using TASSEL 5 (www.maizegenetics.net). To further validate the results of NJ-tree, we used principle component analysis (PCA) with the SNP and Variation Suite (SVS v8.1.5) (Golden Helix, Inc., Bozeman, MT, USA; www.goldenhelix.com). Estimation of *F*_*ST*_ was based on Wright's F statistic (Weir and Cockerham, 1984) with use of SVS v8.1.5.

### Characterization of LD

For generating GBS data, we considered only SNPs that mapped to the melon whole-genome sequence draft, as the chromosome location of SNPs helps prevent spurious LD, thus reducing errors in GWAS. For haplotype estimation, we used “Minimize historical recombination,” a block-defining algorithm developed by Gabriel et al. (2002). This method is an iterative technique for obtaining maximum likelihood estimates of sample haplotype frequencies. The EM (Expectation Maximization) algorithm was used to estimate adjacent and pairwise measurements of linkage disequilibrium (LD) blocks using haplotype frequencies as formalized by Dempster et al. (1977).

### Association mapping

A set of markers, derived after removing minor allele frequencies, was used to estimate kinship (K) matrix using the software TASSEL 5.0 that uses the proportion of alleles shared between each pair of accessions in the study. PCA correction and method of stratification was followed as in Price et al. (2006). The mixed linear model (MLM) was used to reduce spurious marker trait associations (Type I error showing false positives) resulting from population structure as PCA vectors and K were used as covariance to adjust polygenic background in the analysis.

### Construction of a high-density genetic map using SNP markers

Linkage analysis and map construction of SNPs generated by GBS were performed using the MultiPoint package (http://www.multiqtl.com) (Mester et al., 2004; Korol et al., 2009; Reddy et al., 2014). GBS resulted in a disproportion between the high number of scored markers for the mapping population and population size. Multilocus ordering aims to pick the most informative markers for building a reliable skeletal map with additional markers being anchored to these framework markers using an algorithm based on evolutionary optimization strategy (Mester et al., 2003). MulitiPoint mapping is based on the maximum likelihood estimation to calculate pairwise recombination fractions (rf) for all marker pairs. In this study, preliminary clustering and assignment of markers to a linkage group (LG) was evaluated at an rf = 0.05 threshold. For example, marker *mi* may be assigned to an LGj if recombination between *mi* and at least one marker from LG j is lower than the threshold rf and is lowest compared to the distance to any other LG (Peleg et al., 2008). Selection of “delegates” (bin markers) with the highest information content and stability of their neighborhoods were tested by jackknife resampling, with repeated verification of marker order and removal of unreliable markers to increase the stability of multilocus ordering. SNP loci that mapped to the same location were binned and represented by a single delegate. Stable LGs were joined terminally by incrementally increasing the recombination threshold, with a final rf of 0.30. To avoid erroneous linkage groups based on incorrect marker phase, genotypes of unlinked loci or loci in fragment groups were converted to the alternate phase, reclustered, and assigned to linkage groups as published by Oliver et al. (2011).

### Validation of GWAS results using biparental QTL mapping

Interval mapping and multiple QTL mapping (MQM) were performed using MapQTL5.0 (van Ooijen, 2004). For various QTLs, the genome-wide LOD significance threshold was calculated by the 1000 × permutation test, which restricted the occurrence of Type I statistical errors (false positives) to <5%.

## Results

### Genotyping-by-sequencing

In total, 13,756 SNPs were called for uniquely aligned sequence tags and hence are singletons: 1391, 1188, 1151, 1467, 1170, 1314, 1121, 1290, 937, 706, 1072, and 949 SNPs were mapped to Chrs. 1–12, respectively. Following filtering for a minor allele frequency of 0.05%, total SNPs were reduced to 7609: 833, 637, 669, 754, 667, 746, 668, 679, 540, 284, 608, and 524 mapping to Chrs. 1–12, respectively. We found SNPs at average intervals of 30 kb across the melon genome.

### Molecular genetic diversity among the melon collections

**Figure 1:**
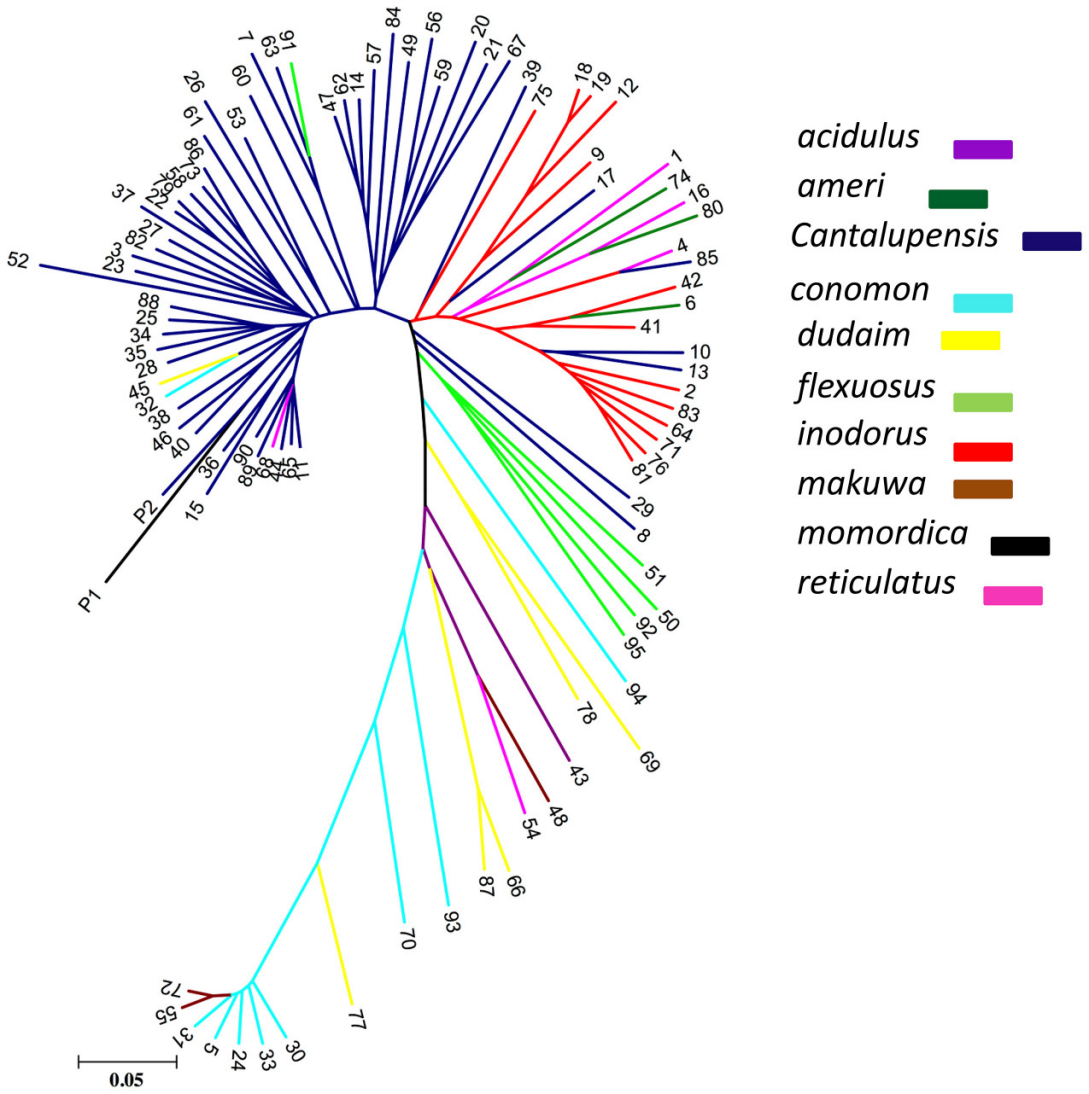
**Phylogenetic tree constructed with neighbor-joining**.

**Figure 2:**
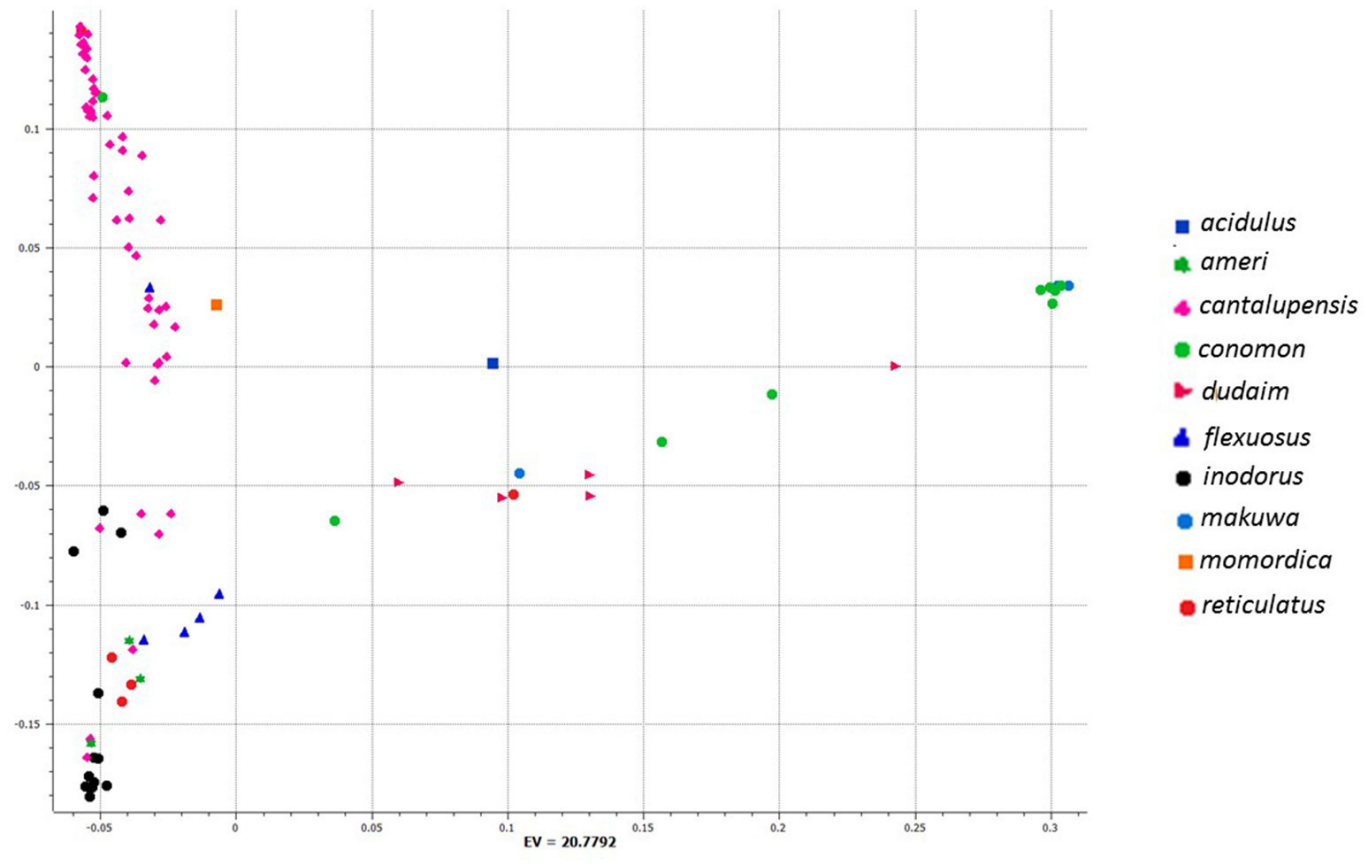
**First and second components of principal component analysis (PCA) of 7609 single nucleotide polymorphisms (SNPs) within a set of 97 melon accessions**. See Table S4 for a list of accessions and respective eigen values for respective positions of individual accessions in the figure.

To investigate genetic differentiation due to population structure among melon horticultural groups as reflected by these 7609 genome-wide SNPs, we used Neighbor Joining (NJ) analysis. In a phylogenetic tree of all analyzed accessions, *cantalupensis* and *inodorus* groups clustered into two separate clusters (subspecies *melo*) along with some mixtures. The cluster of *cantalupensis* is grouped with one accession each of *conomon, flexuosus, reticulatus, dudaim*, and *momordica*. Cluster of *inodorus* had 5 *cantalupensis*, 3 *reticulatus*, and 3 *ameri*. The remaining melon horticultural groups vars. *momordica, conomon, dudaim, flexousus, makuwa*, and *acidulous* (subspecies *agrestis*) were grouped into a widely distributed third cluster (Figure 1). Principle component analysis (PCA) was carried out using 7609 SNPs. A PCA scatterplot of individuals on the first two dimensions corroborated the NJ clustering of samples *cantalupensis, inodorus*, and other groups, with some exceptions as described earlier (Figure 2 and Table S4).

To further resolve differentiation between *cantalupensis* and *inodorus*, we estimated pairwise fixation index (*F*_*ST*_) across all polymorphisms with MAF ≥ 0.05 (Table 1). All *F*_*ST*_ were highly significant (*P* < 0.001). The *F*_*ST*_ between accessions of *cantalupensis* and *inodorus* was 0.23. The *F*_*ST*_ between *cantalupensis* and various *agrestis* accessions was in a range of 0.19–0.53 and between *inodorus* and *agrestis* accessions was in a range of 0.21–0.59. Variety *reticulatus* is ancestral to the genomes of *cantalupensis* and *inodorus*, and very close and equidistant to both the groups with a *F*_*ST*_ value of 0.09.

**Table 1:** **Pairwise ***F***_***ST***_ for combinations of various melon horticultural groups**.

**S. No**	**First sub-population**	**Second sub-population**	***F_st_***	***F_st_* lower conf. bound**	***F_st_* upper conf. bound**
1	*inodorus*	*cantalupensis*	0.22853	0.22179	0.23593
2	*inodorus*	*conomon*	0.50653	0.49872	0.51347
3	*inodorus*	*dudadium*	0.36573	0.35671	0.37435
4	*inodorus*	*flexousus*	0.20508	0.19624	0.21372
5	*inodorus*	*makuwa*	0.59074	0.58191	0.59894
6	*inodorus*	*reticulatus*	0.09514	0.08827	0.10186
7	*cantalupensis*	*conomon*	0.45245	0.44456	0.46045
8	*cantalupensis*	*dudadium*	0.29908	0.29059	0.30749
9	*cantalupensis*	*flexousus*	0.18880	0.18141	0.19691
10	*cantalupensis*	*makuwa*	0.52749	0.51851	0.53702
11	*cantalupensis*	*reticulatus*	0.09288	0.08725	0.09934
12	*conomon*	*dudadium*	0.11659	0.11008	0.12321
13	*conomon*	*flexousus*	0.38349	0.37687	0.39040
14	*conomon*	*makuwa*	−0.08350	−0.08939	−0.07707
15	*conomon*	*reticulatus*	0.32557	0.31873	0.33279
16	*dudadium*	*flexousus*	0.21058	0.20315	0.21858
17	*dudadium*	*makuwa*	0.11820	0.10931	0.12787
18	*dudadium*	*reticulatus*	0.11960	0.11228	0.12717
19	*flexousus*	*makuwa*	0.45809	0.44908	0.46710
20	*flexousus*	*reticulatus*	0.05242	0.04539	0.05989
21	*makuwa*	*reticulatus*	0.36520	0.35630	0.37471

### Haplotypes, LD decay, and chromosome-wise analysis of LD blocks

**Figure 3:**
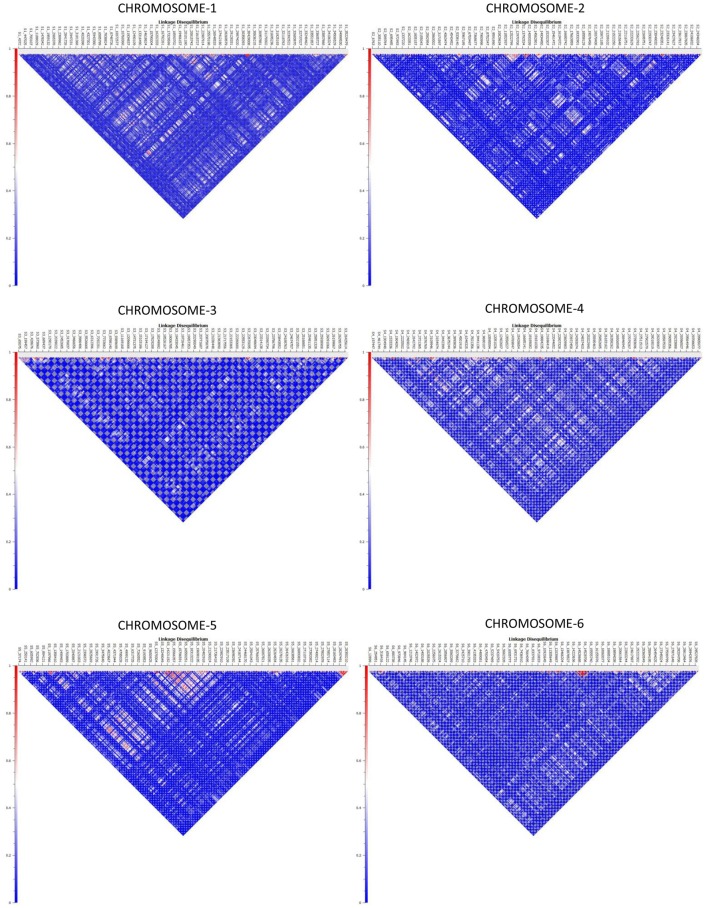
**Genome-wide distribution of LD (***r***^**2**^) across melon chromosomes 1–6**. X-axis contains the SNP markers positioned on the physical map. Y-axis represents the intensity of LD as shown in the scale.

**Figure 4:**
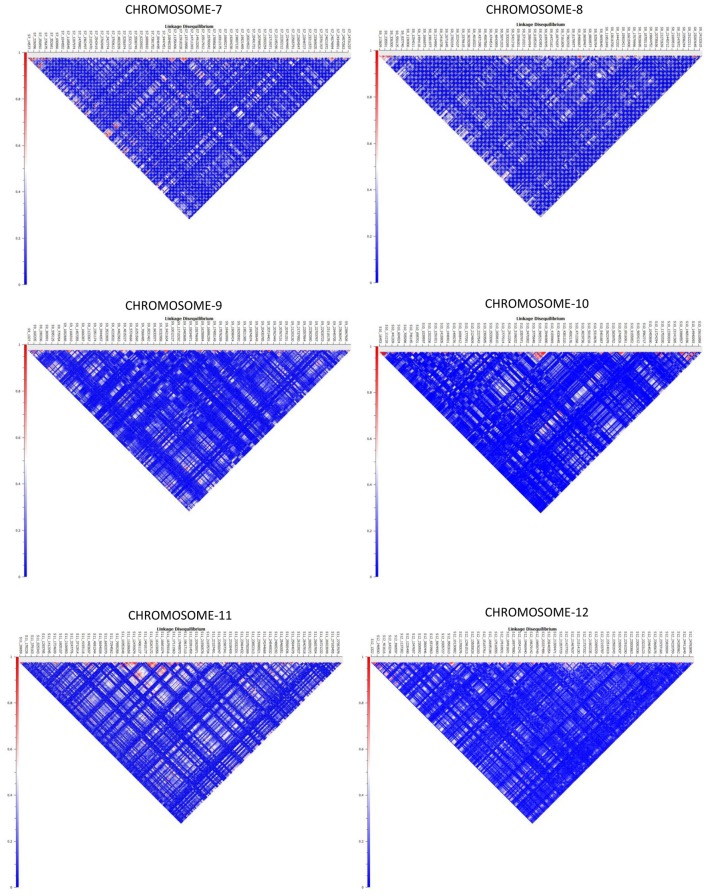
**Genome-wide distribution of LD (***r***^**2**^) across melon chromosomes 7–12**. X-axis contains the SNP markers positioned on the physical map. Y-axis represents the intensity of LD as shown in the scale.

Haplotype distribution is important in comparing common and unique patterns of genetic variation of melon gene pools and has a wide range of applications. The two major processes that shape haplotype structure are the divergence process and breeding history. We used “Minimize historical recombination,” a block-defining algorithm developed by Gabriel et al. (2002). The upper confidence bound was set to 0.98 and the lower bound was set to 0.70. SNPs below MAF of 0.05 were skipped. Maximum block length was set to 160 Kb. The EM (Expectation Maximization) algorithm was used for haplotype estimation with convergence tolerance 0.0001 and frequency threshold of 0.01. Maximum EM iterations were set to 50. We identified 4028 SNPs in 1436 haplotypes from the entire set of melon morphotypes studied (Table S5). We estimated LD by using an entire marker set with MAF ≥ 0.05 and identified 1937 associations in the entire melon collection used in the study (Table S6). The average genome-wide LD decay for the melon genome was noted to be 9.27 Kb, with the means across chromosomes 1–12 being 9.52, 10.11, 4.64, 8.97, 11.07, 7.15, 9.12, 8.87, 8.61, 8.92, 16.46, 7.85 Kb, respectively. Heat maps depicting individual LD blocks (*r*^2^ for set of markers in LD) across the length of chromosomes are presented in Figures 3, 4.

### High density genetic map

**Figure 5:**
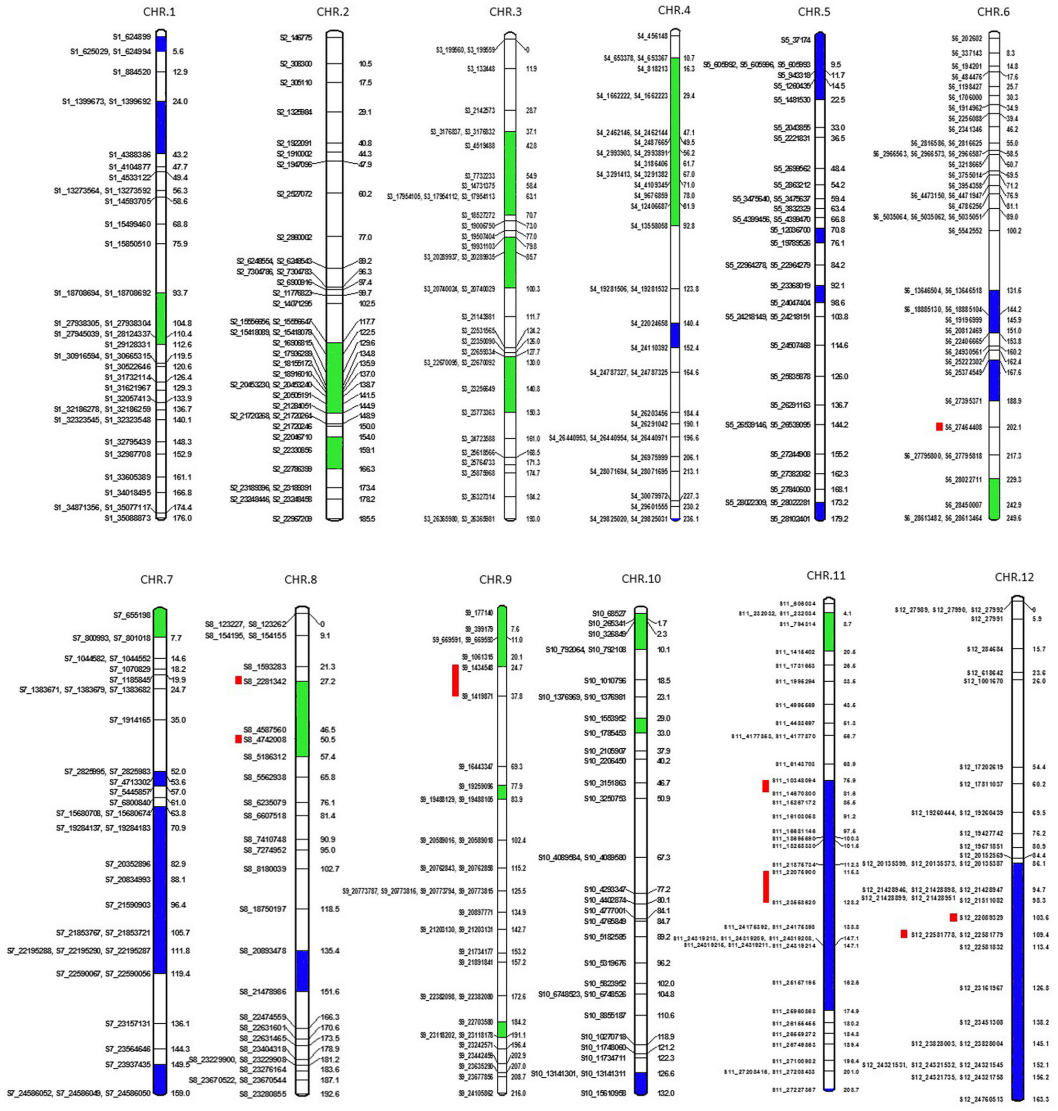
**Genetic map showing positions of skeletal markers on which a high-density genetic map is constructed**. Blue is segregation distortion skewed toward female parent and green toward male parent. Red bars show the location of QTLs for fruit firmness.

A total of 7896 polymorphic SNPs were used to assemble the genetic linkage map using a mapping population that contained 91 F2 progeny, generated from a cross of MR-1 (*momordica*) and “Hales Best Jumbo” (P2), a Western Shipped Cantaloupe. We generated 807, 964, 628, 606, 724, 743, 520, 687, 631, 551, 450, and 585 SNPs for Chr-1 to Chr-12, respectively. Chromosome distribution of 431 skeletal markers is presented in Figure 5. The remaining “add on” or anchor markers are presented in Figure S1. Skeletal markers are the framework markers that have high confidence. In order to select skeletal markers, SNPs that are violating map stability upon mapping were removed and linkage groups were re-analyzed several times until the map attained complete stability. Chromosomes 1 through 12 consisted of 38, 39, 35, 36, 35, 42, 36, 28, 34, 32, 39, and 37 skeletal markers, respectively with the genetic lengths (cM) of 176, 185.5, 193, 236.1, 179.2, 249.6, 159, 192.6, 216, 132, 208.7, and 163.3, respectively. In addition, the current genetic map defines 1837 recombination events within the skeletal map. Each recombination bin or skeletal marker segregated with multiple add-on markers that aided in the development of the proposed high-density genetic map. This map consists of 79, 54, 79, 95, 91, 100, 57, 85, 65, 43, 63, and 54 markers on Chr-1 through 12, respectively.

**Figure 6:**
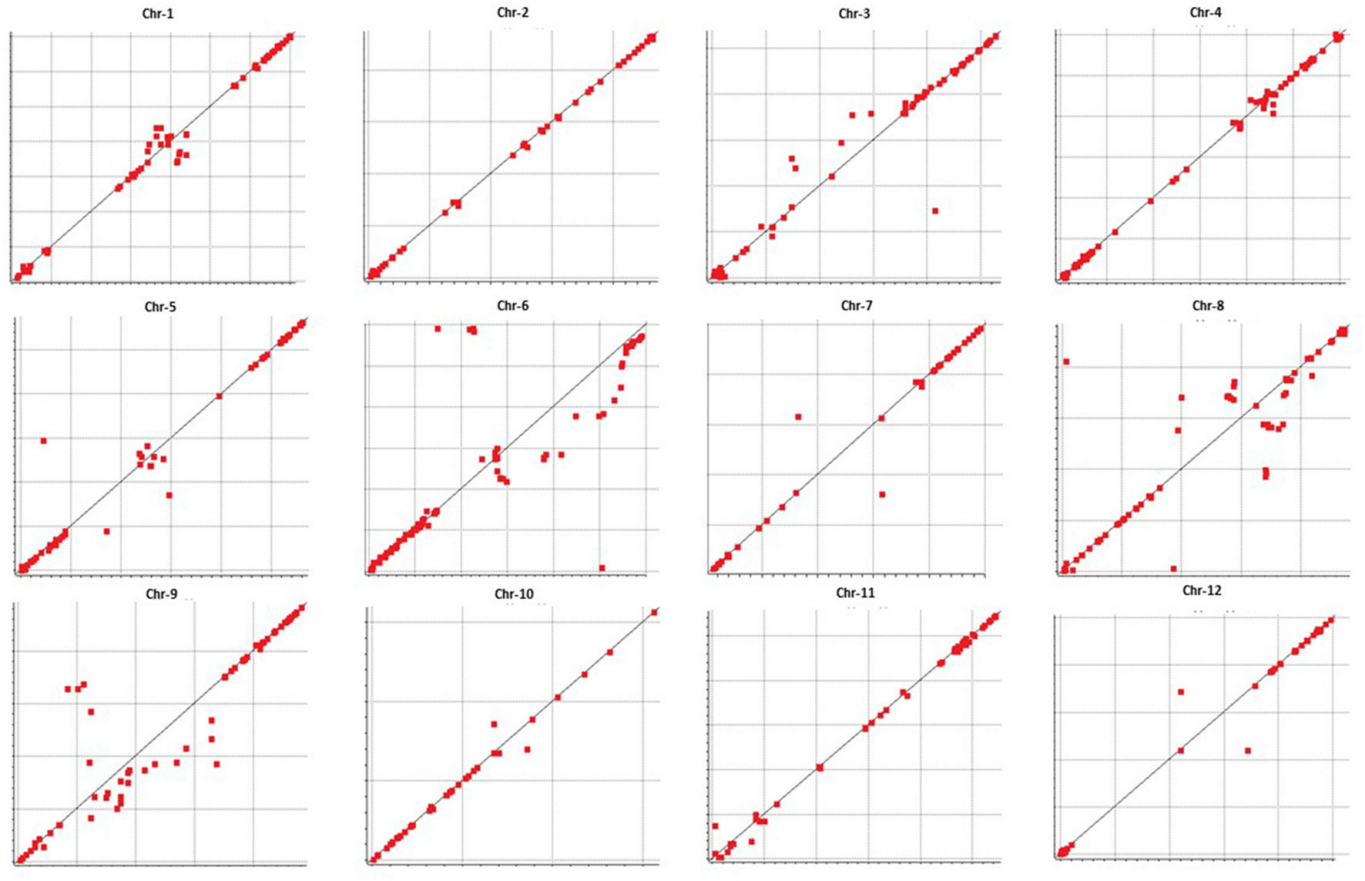
**Collinearity between genetic (vertical axis) and physical maps (horizontal axis) (markers that are distant from the “line of best fit” are not collinear)**.

We examined colinearity of genetic and physical maps for various chromosomes (Figure 6). Markers on Chr-2, -4, -7, -10, -11, and -12 were highly co-linear with respect to their physical locations. Chr-1 and -5 were moderately in agreement with the melon reference sequence. Chr-6, -8, and -9 showed the highest disagreement between the genetic and physical map having a large segment which was not in colinearity with the physical map.

**Figure 7:**
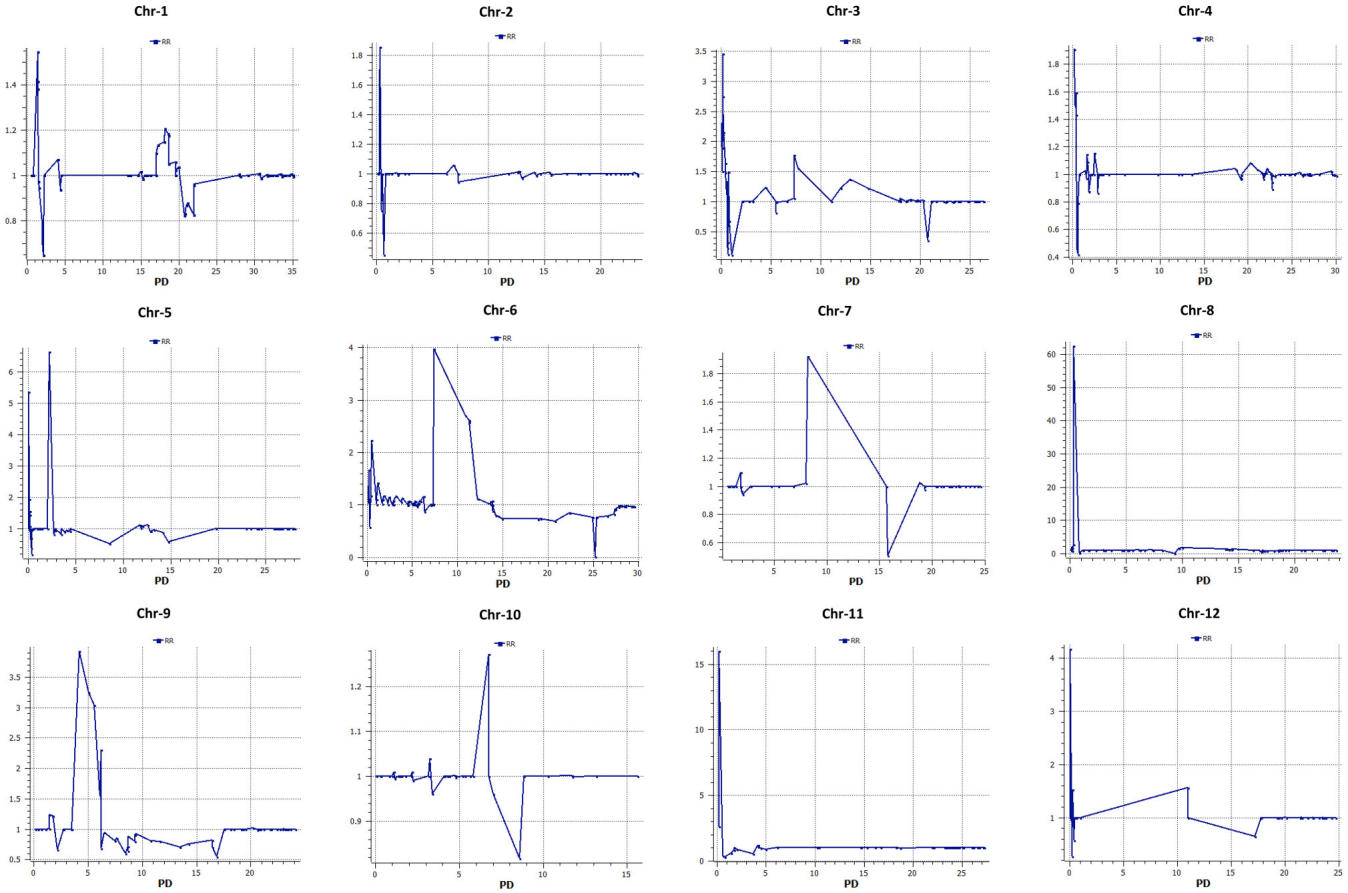
**Distribution of genome-wide recombination rate (GWRR) along chromosomes in the melon genome**. In each plot, the horizontal axis (in Mb) represents the physical distance (PD) along the reference chromosomes and the vertical axis (cM/Mb) the genetic-to-physical distance ratio (green).

Six hundred and thirty-two loci exhibited significant segregation distortion based on χ^2^ test (*P* < 0.05), and those locations were mapped onto the final map (Figure 5). Sixteen segregation distortion regions were skewed toward the female parent MR-1 (*momordica*), and 15 regions showed segregation distortion toward male parent Hale's Best Jumbo. Chromosome-wide distribution of loci skewed toward either of the parent is shown in Table S7. Genome-wide Recombination Rate (GWRR) reflects recombination landscape and is one of the critical factors in shaping the cultivar divergence. GWRR was estimated using the formula cM/Mb. We observed wide variation of GWRRs within and among the chromosomes. A range of 0.01–60.2 noted across the genome (Figure 7). The highest GWRR was on chromosome 12 followed by chromosome 5.

### Implementation of a medium-resolution genome-wide association study for fruit firmness

A MLM was used in the current study to locate QTLs for fruit firmness two consecutive years in two locations. Lists of markers that were linked to various traits are listed along with their corresponding *R*^2^, *P*-values, and allelic contributions across all the years are listed in Table 2. A majority of the QTLs detected are repeated across the years and locations indicating the robustness of the identified QTLs. In this study, we found 11 SNPs across five (6, 8, 9, 11, and 12) chromosomes tightly linked to fruit firmness, which is an important trait for ripening as well as shelf life and transportation of melon fruits. All SNPs except one (S11_14670800) were located on the coding regions of annotated genes (Table 3).

**Table 2:** **Common QTL for fruit firmness identified using GWAS across various years and locations**.

**Marker**	**Chr #**	**Year**	***P*-value**	***R*^2^ Value**	**No. of observations**	**Allele**	**Alleic estimate**
S6_27464408	6	WVSU14	0.00636697	0.11068	16	G	0.3804
					45	A	0.0000
		ASU14	0.01190832	0.08363	18	G	0.2576
					50	A	0.0000
		ASU13	0.03077768	0.07494	19	G	0.2445
					42	A	0.0000
S8_2281342	8	ASU13	0.00859468	0.10439	61	A	−0.6225
					3	G	0.0000
S8_4742008	8	WVSU14	0.00019337	0.18744	29	C	0.4892
					36	T	0.0000
		ASU13	0.00086684	0.16225	31	C	0.3651
					33	T	0.0000
		ASU14	0.00284445	0.10946	31	C	0.2931
					42	T	0.0000
S9_1434548	9	WVSU14	0.03443006	0.06522	49	T	−0.5132
					15	C	0.0000
S9_1419871	9	WVSU13	0.03278305	0.06609	61	C	1.0610
					9	G	0.0000
S11_22075900	11	WVSU14	0.00179356	0.14021	35	T	0.4178
					28	C	0.0000
		ASU14	0.00728561	0.09129	39	T	0.2624
					33	C	0.0000
		ASU13	0.04171158	0.06471	39	T	0.2257
					24	C	0.0000
S11_23558620	11	ASU13	0.03427684	0.06903	47	G	0.2845
					17	C	0.0000
S11_10348094	11	WVSU14	0.00013635	0.19062	56	T	−0.6107
					10	A	0.0000
		ASU14	0.02314808	0.06354	67	T	−0.3195
					8	A	0.0000
S11_14670800	11	WVSU14	0.00451343	0.11068	59	G	−0.5091
					8	C	0.0000
S12_22581778	12	WVSU14	0.03977535	0.06171	30	C	0.2738
					35	A	0.0000
S12_22089329	12	ASU14	0.03383172	0.06011	18	C	0.2196
					52	T	0.0000

**Table 3:** **Fruit firmness QTL identified using MQM mapping in a biparental cross**.

**Chro**	**Position**	**Locus**	**LOD**	**No. Iter**.	**mu_A**	**mu_H**	**mu_B**	**Variance**
6	209.1	S6_27464408	4.71	57	1.72964	1.07326	1.00067	0.014284
8	33.2	S8_2281342	4.23	69	0.944673	1.63268	1.22436	0.009593
8	51.5	S8_4742008	4.05	10	0.979054	0.549409	1.44223	0.033548
9	31.7	S9_1419871	7.17	40	0.961504	0.912009	1.56174	0.012992
9	29.7	S9_1434548	6.1	33	0.945704	0.93609	1.51578	0.014465
11	114.3	S11_22075900	5.57	42	0.765521	1.164	1.65131	0.010346
11	122.3	S11_23558620	7.59	40	1.4391	0.8246	1.22233	0.012139
11	77.9	S11_10348094	3.3	31	0.916911	0.872679	1.55102	0.021879
11	80.9	S11_14670800	3.46	19	1.47125	0.867167	0.812748	0.023819
12	106.6	S12_22581778	4.03	13	1.13738	1.09658	1.75754	0.024096
12	105.6	S12_22089329	3.64	17	1.13641	1.09944	1.75566	0.024156

*mu_A, mu_H, and mu_B are the allelic effects of female parent, heterozygote, and male parent respectively*.

### QTL validation in biparental mapping

F_2_ progeny of MR-1 and Hale's Best Jumbo were evaluated in controlled conditions to validate various QTLs identified using GWAS. Entire list of QTLs identified using GWAS in the current study could be validated with the results of MQM mapping. Additional QTLs that are identified in MQM mapping but not in GWAS were not shown as these QTLs need further validation. Details pertaining to the QTLs that are validated in GWAS are presented in Table 4.

**Table 4:** **Location of SNPs on various chromosomes and annotation of corresponding genes**.

SNP locus (±)	Ma 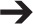 Mi	Sequence (coding sequence from mRNA)	Annotation	Function
S6_27464408 (+)	A 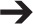 G	CTGCTTCAAAGGGAGAAAGAAACGTACAGTCAGTCATTGAAGAGACTACAAAGACCAAGGAGGA	E3 ubiquitin-protein ligase	Protein degradation
S8_2281342 (−)	T 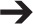 C	CAGCTTCTTTCTGCTCTCTGTTGTTGCCGTTTCGTAAGAGATAAACCAGAGAACGAACTGCATA	U-box domain-containing protein	Protein degradation
S8_4742008 (+)	T 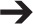 C	CAGCCCAATGAGCCTTGTTGAGGGATTTCAAGAGGATGCAGAGACTATATTCTTTGCATCTGGC	Beta-D-xylosidase	Modification of secondary cell wall
S9_1419871 (−)	C 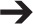 G	CTGCGGAAGATGTTGCTGAAGGTCTAGATGACTGACTTGAACTACTTGAAGACATGGCTTCTAC	Transmembrane protein	Molecules transport
S9_1434548 (+)	A 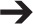 G	CTGCAAACATTTTGATACAAACAAATTTCGGTGAGTAGCCAAGCATCCATGGTCAGTGGCAGAA	Putative nuclear matrix constituent protein	Gene transcription and DNA replication
S11_10348094 (+)	A 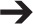 T	CAGCTATAAGTCGGATTGCAAAAGCGGAGAATGATTTTCACAAGATGATGGATCTTAAAAGCCT	Histidine kinase	Signal transduction from external cues
S11_14670800 (−)	A 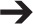 C	CTGCCCACCACTTCACATATTAAGGCCACGCAAAAAAAGTTGTCGATTTCCCTTGCTTGCCGCA	Intergenic	N/A
S11_22075900 (−)	G 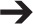 A	CAGCCACGTGGCATCCATGTAGTCCTCAAAGAGAGGAGGAATTAACTAAAGAAGCCAACCTGGT	MLO-like protein	Defense response
S11_23558620 (−)	G 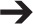 C	CTGCGGCGGCGACTGCCTTTTACGACCACGCCGGCGGTGGGGCCCTGCATAATGCAGGTCCCAC	Kinesin-13	Motor protein—cell division
S12_22089329 (+)	T 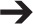 C	CAGCATCGTCACGTCTCATGGATCGAATTTGGTTGGGGGCATTCCCCATGTTGTTGGGGAAGAC	Glyoxysomal malate synthase	Carbohydrate metabolism
S12_22581778 (+)	A 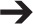 C	CAGCTGATAAAAGCCGGATTATCTTCTTTGAAACAACTTGTCTGACATTCAAAAGAAACAAGCT	Chloroplastic anthranilate phosphoribosyltransferase	Hormone signaling

*±, gene orientation along chromosome; Ma
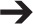
Mi, major and minor allele of SNPs; red bold letter, SNP location within coding region*.

## Discussion

As genotypic data become easier to obtain, it is possible to analyse a more complete and accurate landscape of genetic diversity and linkage disequilibrium, reduce false positives arising from population structure, and target true biological associations (Lipka et al., 2015). In this research, we took advantage of whole-genome sequence data of melon to map SNPs generated by GBS to various chromosomes, thus estimating chromosome-wise divergence and characterized genome-wide LD. To date, the genetic basis of this diversity and the consequences of selection on genetic variation involving various horticultural groups have not yet been studied on a genome-wide basis (Blanca et al., 2012).

Several important studies have been conducted to generate SNPs for the melon genome for use in characterization of genetic variation, population structure, and LD (Blanca et al., 2012; Esteras et al., 2013; Leida et al., 2015). A true sense of genomic characterization is only possible after anchoring to the WGS (Argyris et al., 2015; Sanseverino et al., 2015). Sanseverino et al. (2015) resequenced seven melon varieties using a paired-end approach to generate 4,556,377 SNPs between *melo* and *agrestis* to study genome-wide nucleotide diversity. However, a large number of diverse accessions needs to be included, such as in the current study, to accurately understand genome-wide distribution of LD and to identify markers linked to various traits.

Our genetic diversity study clearly differentiated *melo* and *agrestis* into various clusters, this is in agreement with several previous studies (Stepansky et al., 1999; Garcia-Mas et al., 2000; Mliki et al., 2001; Staub et al., 2004; Nakata et al., 2005; Nimmakayala et al., 2009; Tomason et al., 2013). An admixture of *melo* and *agrestis* genomes by intentional and unintentional crossing is evident as shown in several previous genetic diversity studies in melon (Blanca et al., 2012; Esteras et al., 2013; Hu et al., 2015; Sanseverino et al., 2015). As *cantalupensis* and *inodorus* cultivars are fully cross-compatible with the groups of *agrestis*, the variability found in *agrestis* groups (such as *conomon* and *momordica*) has been used as a source of disease resistance for breeding purposes (Blanca et al., 2012; Sanseverino et al., 2015). In spite of *conomon, flexuosus*, and *momordica* mixtures in clusters belonging to *cantalupensis* and *inodorus*, our study clearly differentiates genetic boundaries as shown in NJ tree analysis and principle component analysis between *cantalupensis* and *inodorus*.

Pairwise *F*_*ST*_ indices for various SNP markers across the length of chromosomes could be used to identify important genomic regions contributing to genetic differentiation among horticultural groups in the study. Similar to our results, Sanseverino et al. (2015) compared *melo* and *agrestis* and identified genomic regions exhibiting extreme population differentiation regions on chromosome 1, 3, 7, 8, and 11, indicating that the process of genetic differentiation of melon subspecies is a genome-wide process.

In spite of rapid progress in sequencing technologies for creating affordable physical maps, high density genetic linkage maps are still indispensable for identification of genomic regions carrying quantitative trait loci (QTL) controlling agronomical traits. In addition, genetic linkage maps with high density SNPs and structural variants are a prerequisite for further map-based cloning and comparative genome analysis (Delourme et al., 2013; Reddy et al., 2014; Diaz et al., 2015; Ren et al., 2015). The high-resolution genetic map presented in the current study should prove useful in associating colinearity with the physical map. The disagreements between the genetic map and the physical map should be extremely useful for future melon genome sequencing endevours. Since the parents of the genetic mapping population are *momordica* and *cantalupensis*, map regions that are skewed to female or male parent will shed light on cryptic recombination sites between the morphotype genomes. A MLM was used in the current study to locate QTLs for two consecutive years in two locations for fruit firmness. The most intriguing part of the current study is that many of the QTLs identified based on GWAS could be validated with the QTL analysis using biparental mapping.

From our association study, we mapped S8_4742008, S12_22089329, S12_22581778, and S11_10348094 to the coding region of β-D-xylosidase, glyoxysomal malate synthase, chloroplastic anthranilate phosphoribosyltransferase, and histidine kinase respectively that are strongly associated with cell wall metabolism (Fischer and Bennett, 1991; Huber and O'Donoghue, 1993; Zablackis et al., 1995; Brummell and Harpster, 2001; Minorsky, 2002; Goujon et al., 2003; Itai et al., 2003; Tateishi et al., 2005; Di Santo et al., 2009) thereby affecting fruit firmness. β-xylosidase (*AtBXL1*) in *Arabidopsis* is thought to be involved in the organization and loosening cellulose deposition in the secondary cell wall (Goujon et al., 2003). During fruit ripening, several cell wall metabolizing enzymes contribute to changes in cell wall architecture (Fischer and Bennett, 1991). In addition, pectic and hemicellulosic polysaccharides become soluble causing depolymerization of the cells with the help of neutral sugars (Huber and O'Donoghue, 1993; Brummell and Harpster, 2001). The hemicellulosic components of the primary cell wall in dicots consist of well-characterized xyloglucans (Zablackis et al., 1995). The metabolism of xyloglucans in the cellulose microfibril network is believed to be important for cell-wall expansion. This loosening of the cell wall happens when xyloglucans tether the adjacent microfibrils for further modification (Minorsky, 2002). Various modifications of the cell-wall in developing and ripening fruits are thought to be mediated by cell-wall degrading enzymes such as β-D-xylosidase, which is involved in the breakdown of xylans. Earlier studies demonstrated that one of the functions of β-D-xylosidase is to control fruit development and ripening in tomato (Itai et al., 2003) and Japanese pear (Tateishi et al., 2005). This gene may play a role in melon fruit firmness by regulating rind thickness and pressure. Interestingly, ethylene production and accumulation of transcripts of β-D-xylosidase coincidentally occurred with increased fruit firmness in peach (Di Santo et al., 2009). Ethylene-stimulated malate synthase, a key enzyme responsible for malic acid synthesis in the glyoxylate cycle, converts lipids to carbohydrates and was found to be highly expressed during fruit ripening in banana (Pua et al., 2003). Moreover, malate has been shown to be involved in starch metabolism, ripening and postharvest softening in tomato (Centeno et al., 2011). Our GWAS identified another SNP (S12_22581778) located in the coding domain of a putative anthranilate phosphoribosyl-transferase, a gene that plays a role in cell wall metabolism in the presence of ethylene (Li et al., 2012). Histidine kinases (HKs) showed strong association with fruit firmness in the current research. HKs function in two-component regulation systems to transduce signals from hormones and external cues to multiple downstream functions in fruit development (Hwang et al., 2002; Singh and Kumar, 2012).

We characterized a GWAS panel offering the best operational representation of melon diversity so far. This study showed that the melon is one of the most diverse cultivars with considerably rapid decay of LD when assessed at the genome-wide scale. Rapid decay of LD in melon indicates a need for higher-density SNP panels for performing GWAS effectively. These GWAS results demonstrate that high-density SNP markers developed in the study provide an effective tool to dissect the genetic architecture of fruit firmness, although additional evidence is needed to support the identified loci and candidate genes.

## Author contributions

PN, UR, AL, WW, JM, and JG designed the study and drafted the manuscript. PN, YT, VA, AA, VV, GS, and GP generated field and fruit firmness phenotyping. PN, VA, AA, and VV extracted DNA and assisted to generate genomewide SNPs. AK, YR, and UR generated high resolution genetic map. JG provided whole genome sequence draft and mapped SNPs to the genome. UR, YT, PN, VA, and TS performed GWAS, biparental QTL analysis, and gene annotation.

### Conflict of interest statement

The authors declare that the research was conducted in the absence of any commercial or financial relationships that could be construed as a potential conflict of interest.

## Supplementary material

Figure S1 **High resolution genetic map of melon with 7153 loci**.

Table S1 **List of melon accessions used in the current study**.

Table S2 **Fruit pressure across various years and locations**.

Table S3 **Fruit pressure distribution in F2 progeny of a cross of MR-1 (***momordica***) and “Hale's Best Jumbo” (P2), a Western Shipped Cantaloupe**.

Table S4 **Eigen values for the first 10 principle components estimated for various accessions**.

Table S5 **Haplotype blocks across entire melon collection in the study**.

Table S6 **Linkage disequilibrium (LD) analysis for adjacent SNP pairs across melon genome**.

Table S7 **Linkage disequilibrium (LD) analysis for adjacent SNP pairs across melon genome**.
