# Supplementary figures and images for: Genome-Wide Differentiation of Various Melon Horticultural Groups for Use in GWAS for Fruit Firmness and Construction of a High Resolution Genetic Map
Source: Front Plant Sci. 2016 Sep 22;7:1437. doi: 10.3389/fpls.2016.01437 (PMC5031849; doi:10.3389/fpls.2016.01437)

# Chr-1

I

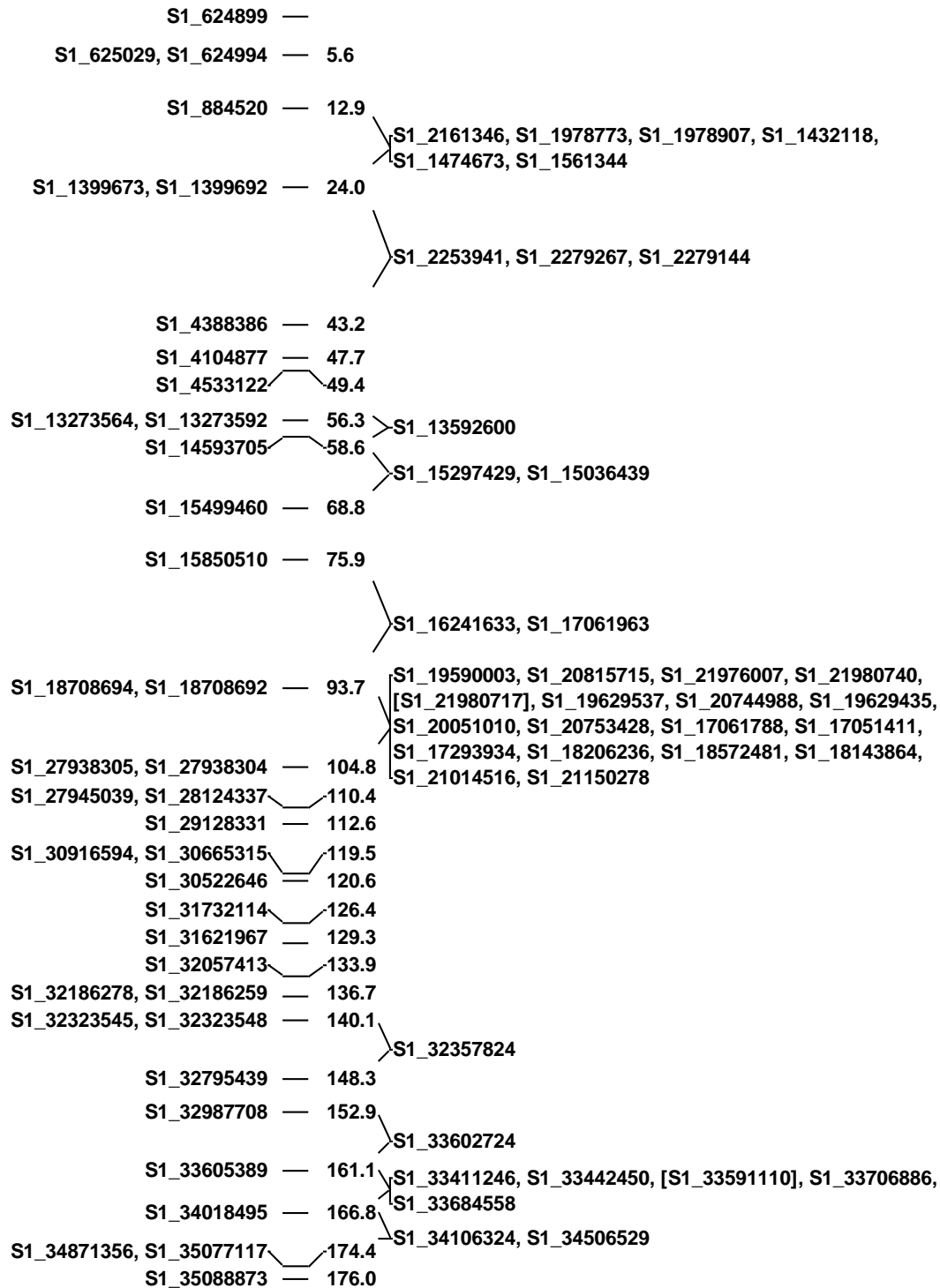

## Chr-2

I

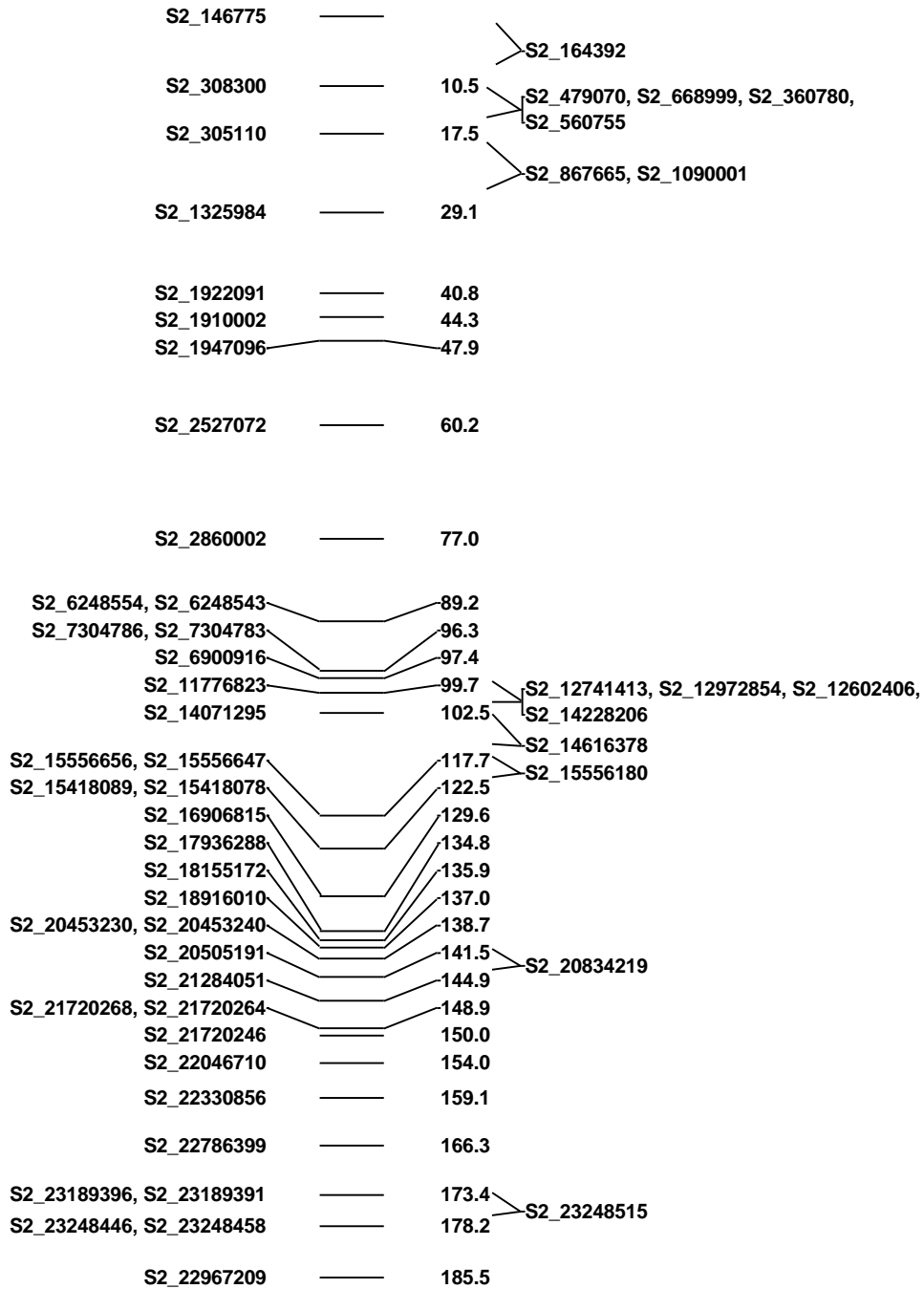

# Chr-3

I

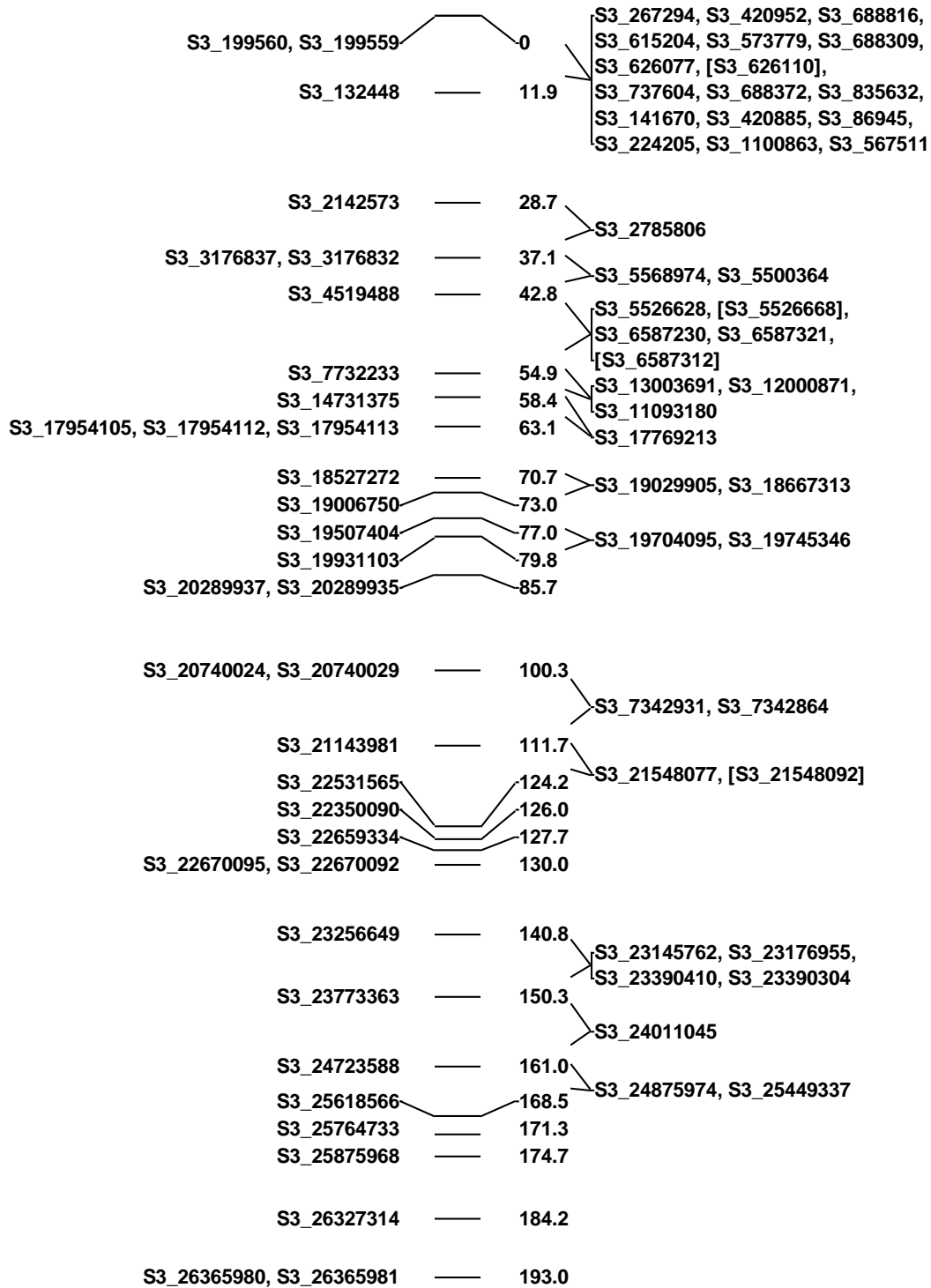

# Chr-4

I

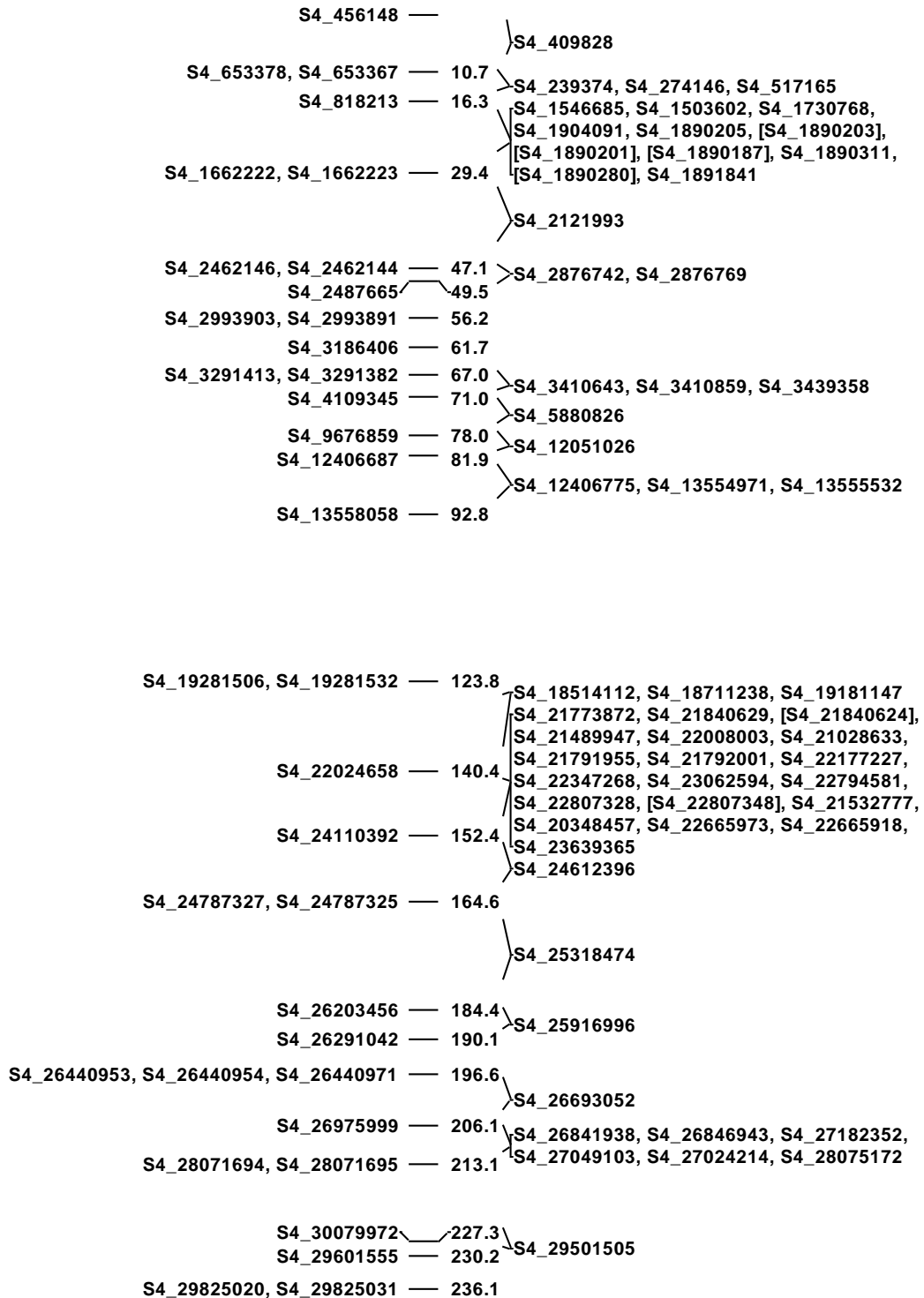

# Chr-5

I

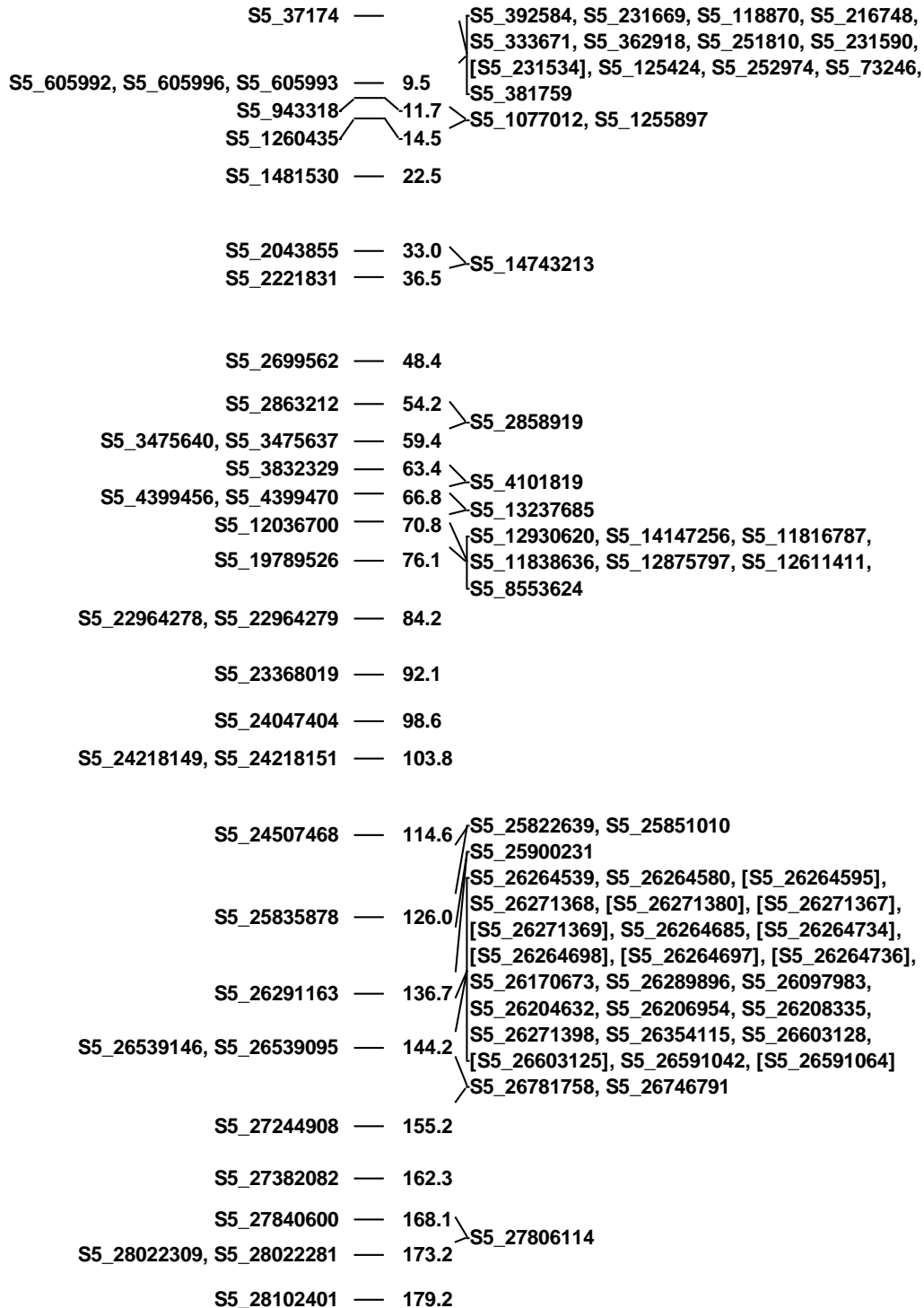

# Chr-6

I

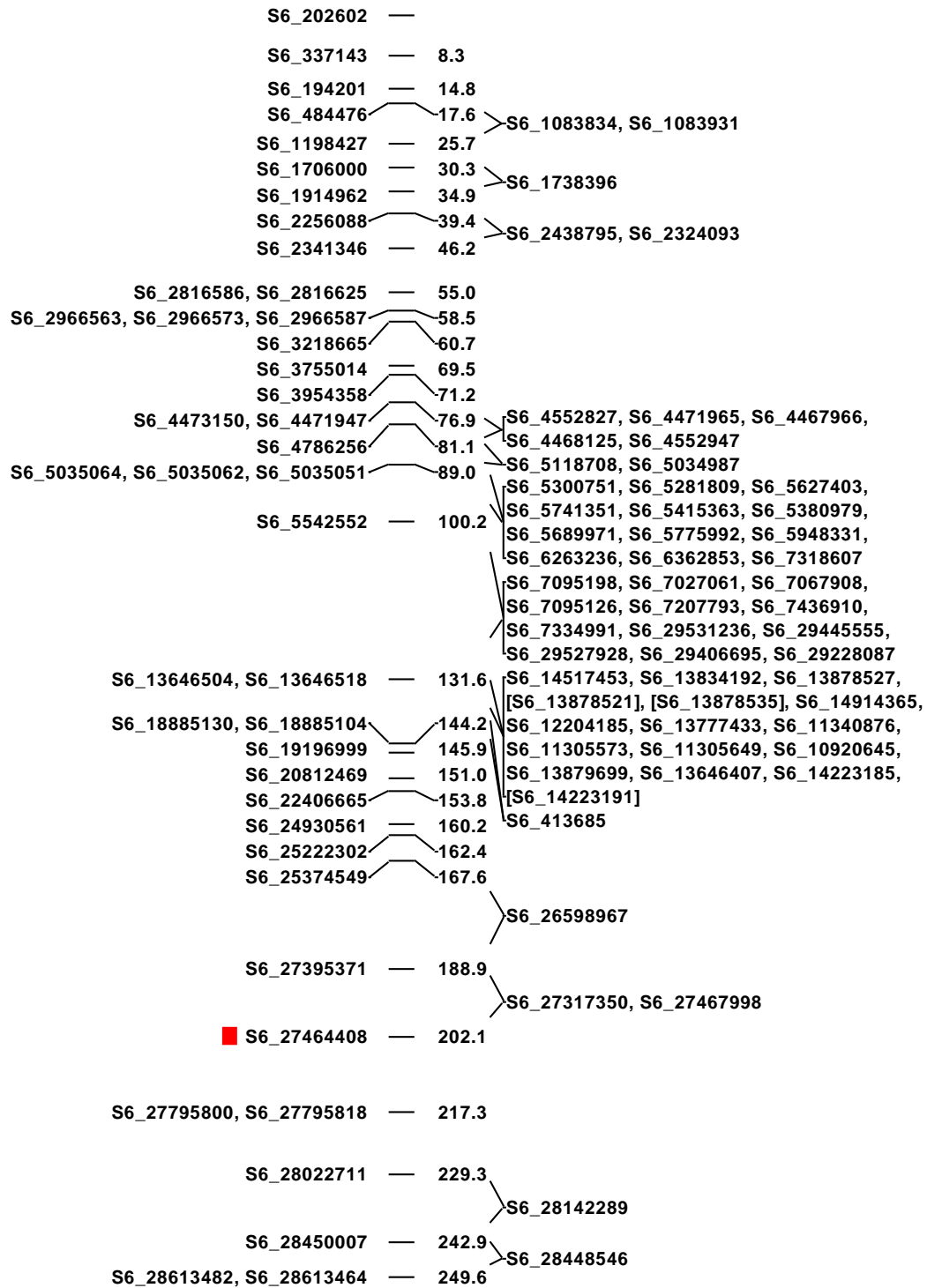

# Chr-7

I

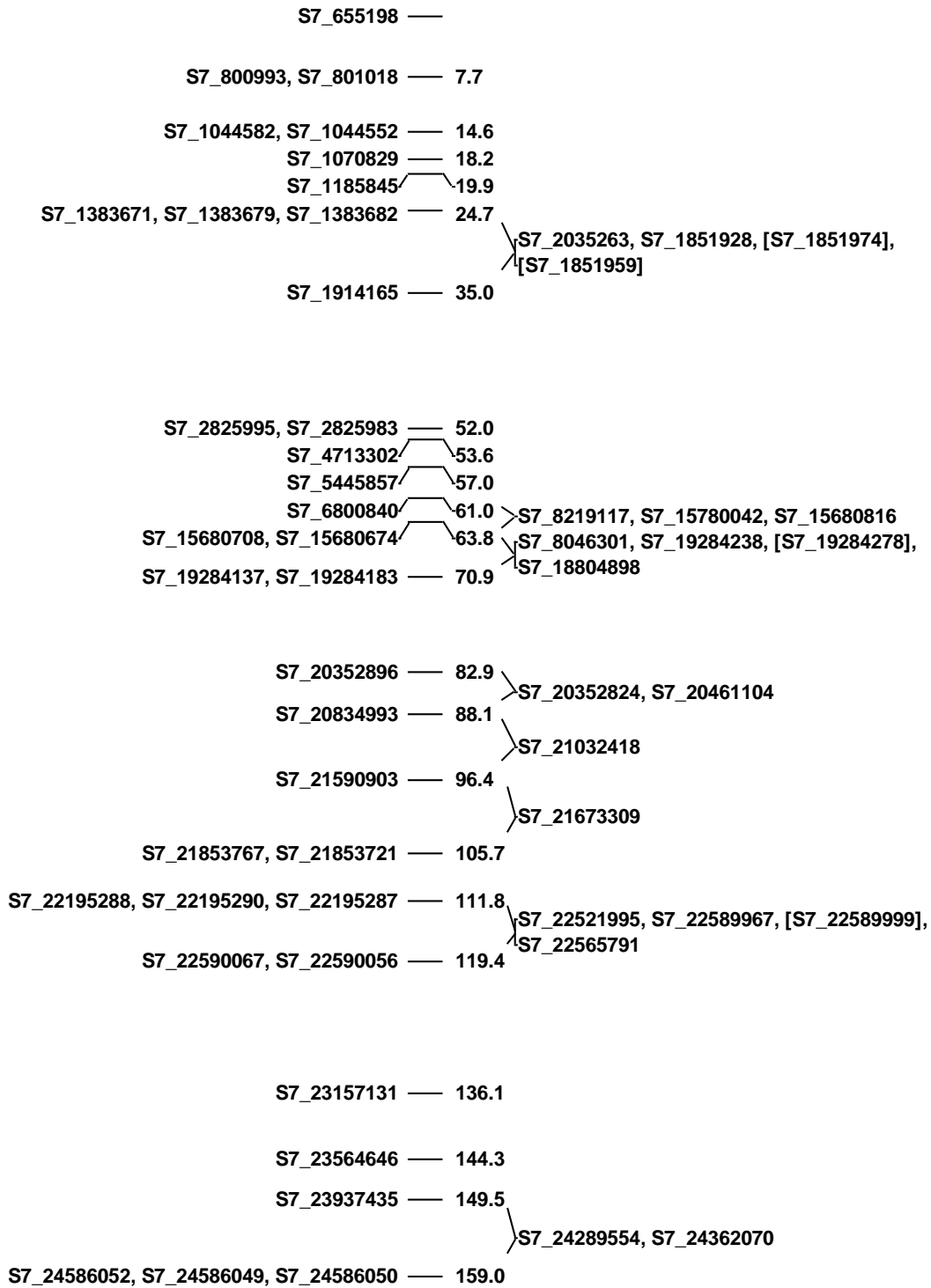

# Chr-8

I

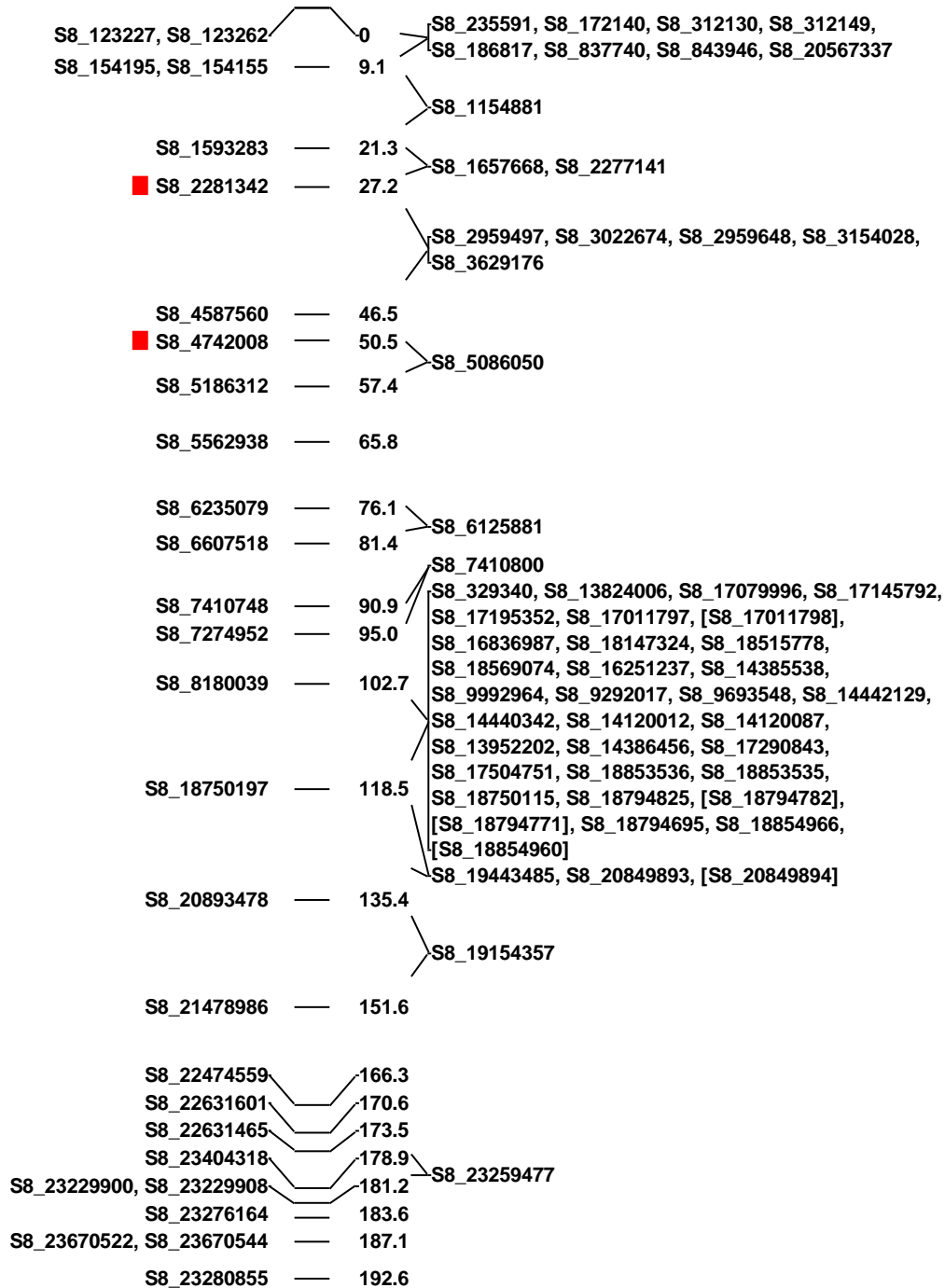

# Chr-9

I

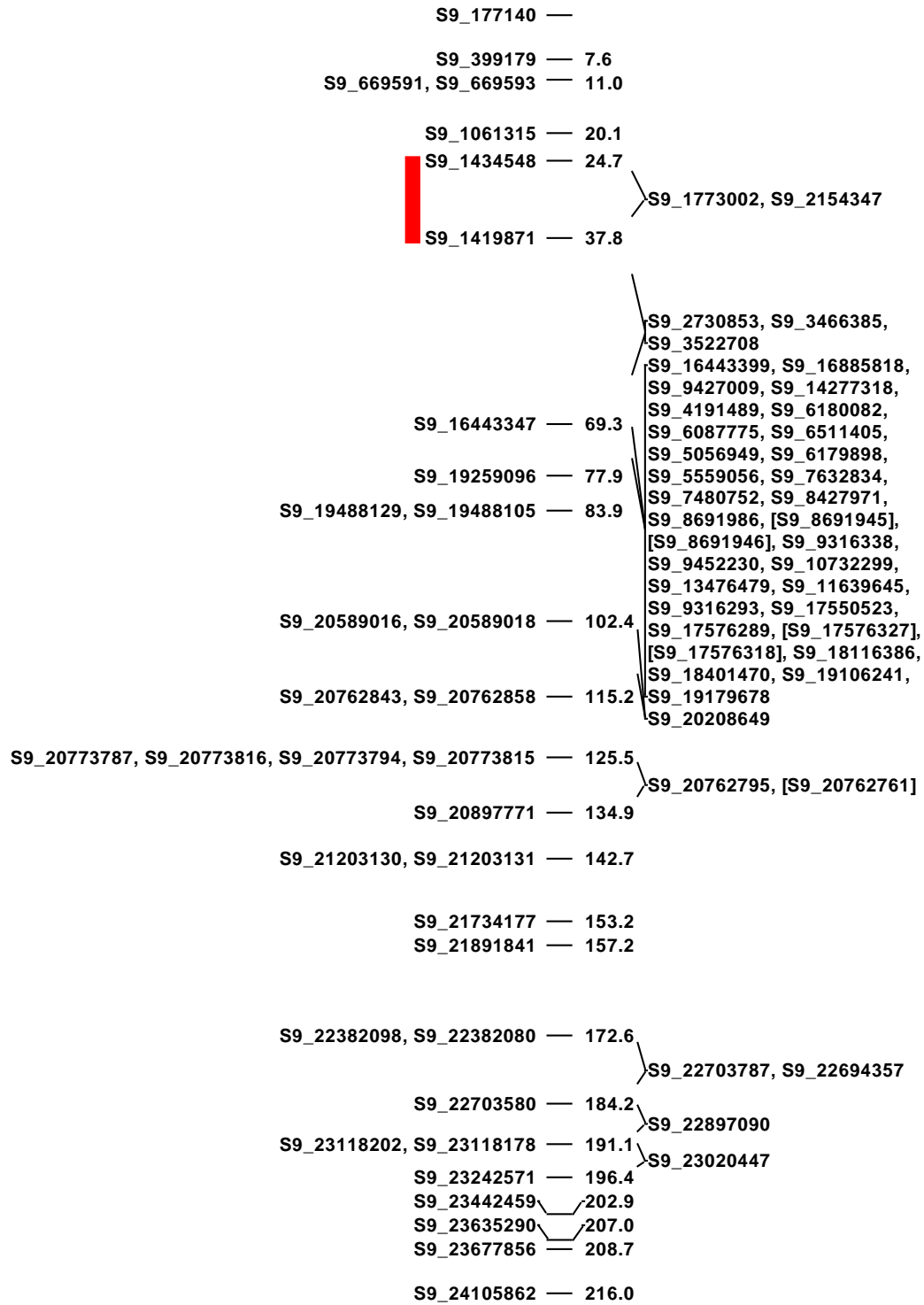

# Chr-10

I

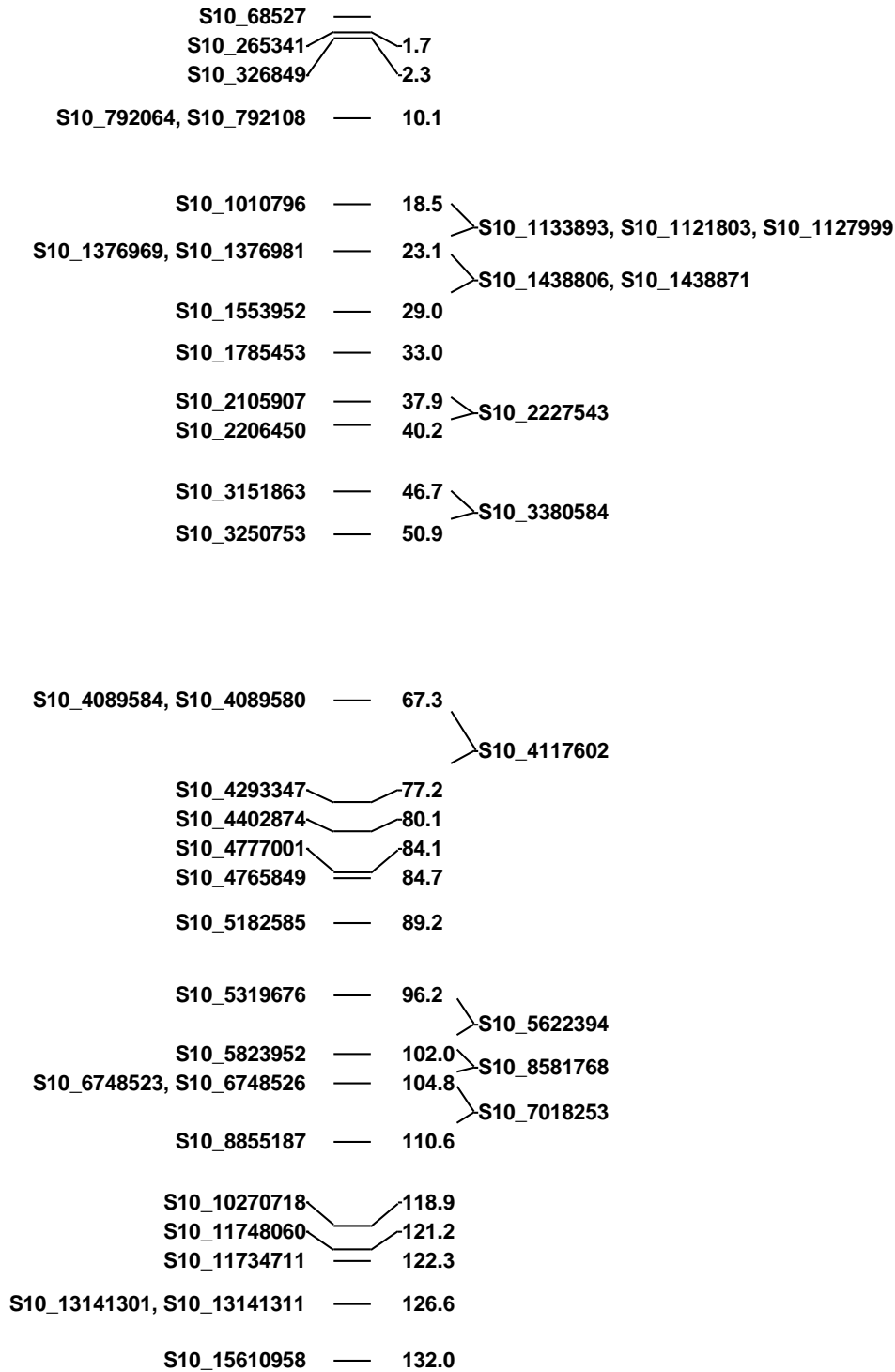

# Chr-11

I

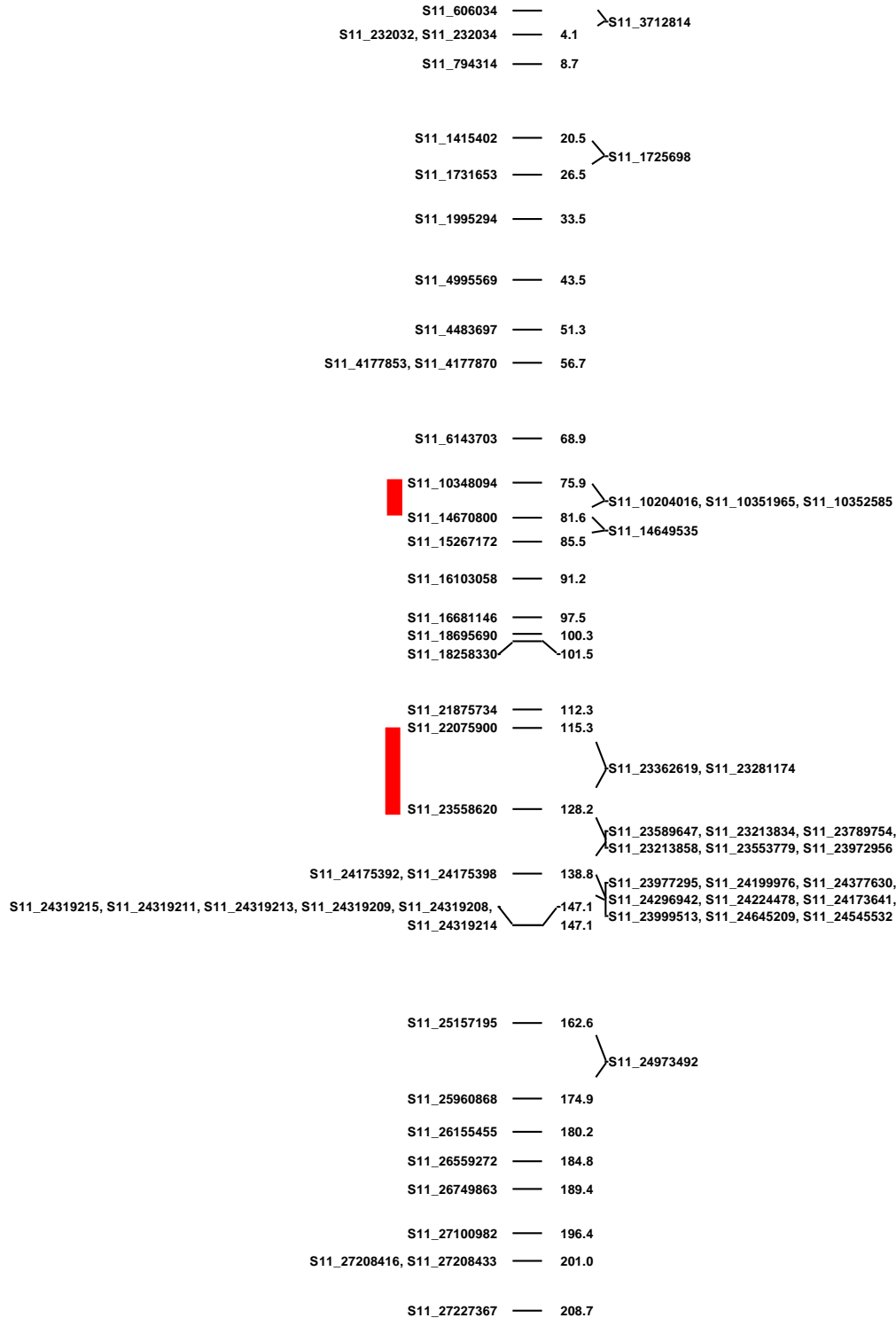

# Chr-12

I

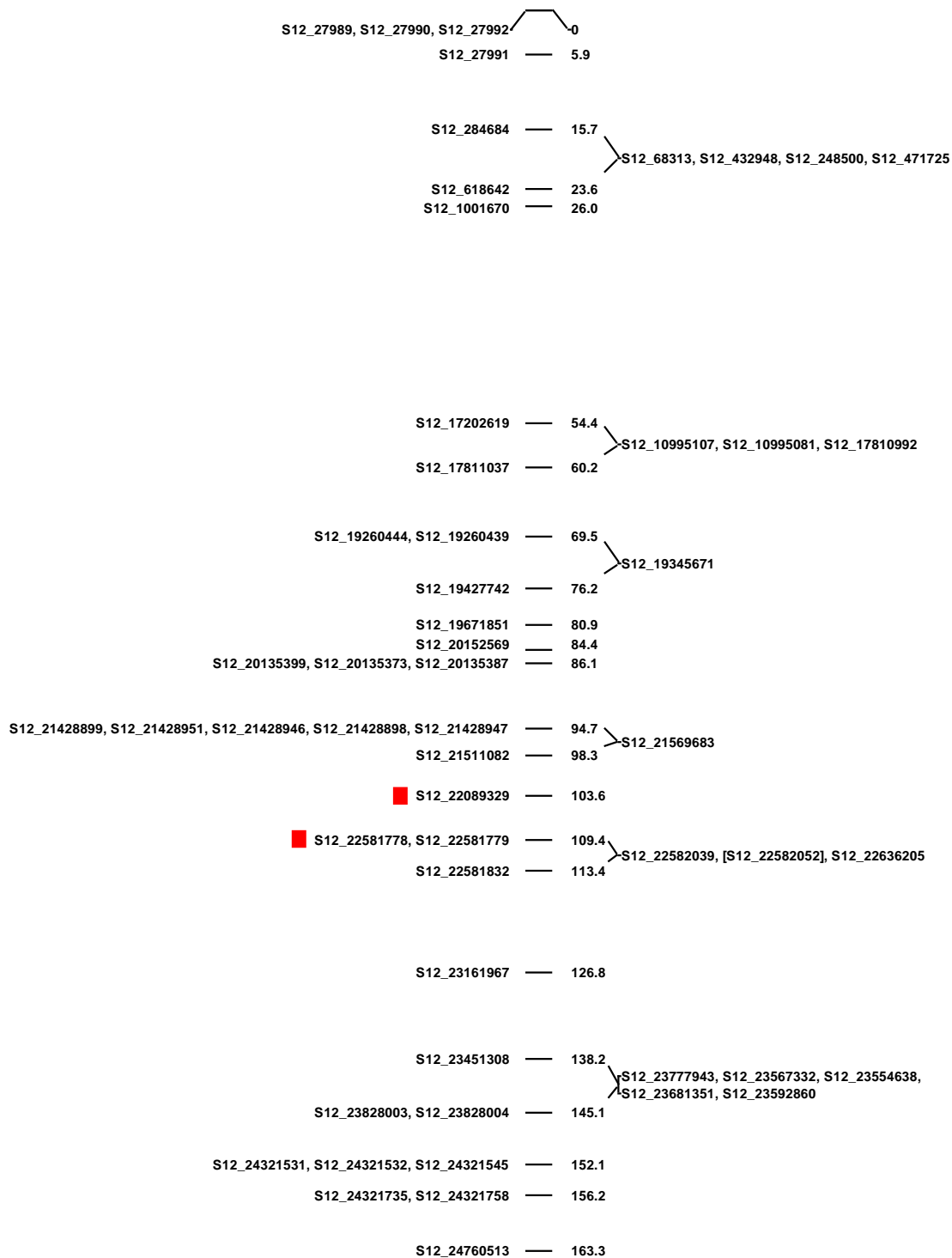

Supplement: Figure S1 — High resolution genetic map of melon with 7153 loci. [file Image1.PDF]
