# Supplementary material for: Genome-Wide Differentiation of Various Melon Horticultural Groups for Use in GWAS for Fruit Firmness and Construction of a High Resolution Genetic Map
Source: Front Plant Sci. 2016 Sep 22;7:1437. doi: 10.3389/fpls.2016.01437 (PMC5031849; doi:10.3389/fpls.2016.01437)
Supplement: Table S1 — List of melon accessions used in the current study. [file Table1.PDF]

Table S1: List of melon accessions used in the current study

S.No	Accsession Name	Group
CM1	Afghan Honeydew Melon	*reticulatus*
CM2	Amarillo Oro Melon	*inodorus*
CM3	Ananas D' Amerique A Chair Verte melon	*cantalupensis*
CM4	Ananas Melon	*reticulatus*
CM5	Apple Melon	*conomon*
CM6	Ashkahabad Melon	*ameri*
CM7	Banana Melon	*cantalupensis*
CM8	Barese Melon	*cantalupensis*
CM9	Bidwell Casaba Melon	*inodorus*
CM10	Boule d'Or Melon	*cantalupensis*
CM11	Burrell's Jumbo Melon	*cantalupensis*
CM12	Casaba-Golden Beauty Melon	*inodorus*
CM13	Cavaillon Espagnol Melon	*cantalupensis*
CM14	Charentais Melon	*cantalupensis*
CM15	Coban Orange Flesh Melon	*cantalupensis*
CM16	Collective Farm Woman Melon	*reticulatus*
CM17	Crane Melon	*cantalupensis*
CM18	Crenshaw Blanco Melon	*inodorus*
CM19	Crenshaw Melon	*inodorus*
CM20	D'Alger Melon	*cantalupensis*
CM21	Delice de la Table Melon	*cantalupensis*
CM22	Delicious 51 Melon	*cantalupensis*
CM23	Early Hanover Melon	*cantalupensis*
CM24	Early Silver Line Melon	*conomon*
CM25	Eden's Gem (Rocky Ford) Melon	*cantalupensis*
CM26	Edisto 47 Melon	*cantalupensis*
CM27	Emerald Gem Melon	*cantalupensis*
CM28	Fordhook Gem Melon	*cantalupensis*
CM29	Gaucho Melon	*cantalupensis*
CM30	Giant Katsura Melon	*conomon*
CM31	Golden Cripsy Melon	*conomon*
CM32	Golden Jenny Melo	*conomon*
CM33	Golden Sweet Melon	*conomon*
CM34	Green Machine Melon	*cantalupensis*
CM35	Green Nutmeg Melon	*cantalupensis*
CM36	Hale's Best 45 Melon	*cantalupensis*
CM37	Healy's Pride Melon	*cantalupensis*
CM38	Hearts of Gold Melon (Hoodoo)	*cantalupensis*
CM39	Hero of Lockinge Melon	*cantalupensis*
CM40	Honey Rock	*cantalupensis*
CM41	Honewdew Orangeflesh Melon	*inodorus*
CM42	Honewdew Tam Dew Melon	*inodorus*
CM43	Indian Cream Cobra Melon	*acidulus*
CM44	Iroquois Melon	*cantalupensis*

S.No	Accsession Name	Group
CM45	Jenny Lind Melon	*dudaim*
CM46	Kansas Melon	*cantalupensis*
CM47	Petit Gris de Rennes Melon	*cantalupensis*
CM48	Mango Melon (Vine Peach)	*makuwa*
CM49	Melon de Luneville	*cantalupensis*
CM50	Metki Dark Green Serp	*flexuosus*
CM51	Metki White Serpent Melon	*flexuosus*
CM52	Minnesota Midget Melon	*cantalupensis*
CM53	Missouri Gold Melon	*cantalupensis*
CM54	Mother Mary's Pie Melon	*reticulatus*
CM55	New Melon	*makuwa*
CM56	Noir de Carmes Melon	*cantalupensis*
CM57	Obus or Kroumir Melon	*cantalupensis*
CM58	Oka melon (Bizard Island Strain)	*cantalupensis*
CM59	Old Greek Melon	*cantalupensis*
CM60	Old Time Tennessee Melon	*cantalupensis*
CM61	Oran's Melon	*cantalupensis*
CM62	Orlinabel Melon	*cantalupensis*
CM63	Pear Melon	*cantalupensis*
CM64	Piel de Sapo Melon	*inodorus*
CM65	Planter's Jumbo Melon	*cantalupensis*
CM66	Plum Granny (Queen Anne) Melon	*dudaim*
CM67	Prescott Fond Blanc Melon	*cantalupensis*
CM68	Rampicante Zuccherino	*reticulatus*
CM69	Rich Sweetness 132 Melon	*dudaim*
CM70	Riddle Melon	*conomon*
CM71	Rugoso di Consenza Melon	*inodorus*
CM72	Sakata's Sweet Melon	*makuwa*
CM73	Schoon's Hardshell Melon	*cantalupensis*
CM74	Small Persian Melon	*ameri*
CM75	Swan Lake Melon	*inodorus*
CM76	Tendral Verde Tardif Melon	*inodorus*
CM77	Thai Golden Round Melon	*dudaim*
CM78	Tigger Melon	*dudaim*
CM79	Tip Top Melon	*cantalupensis*
CM80	Uzbek Sweetness Melon	*ameri*
CM81	Valencia Winter Melon	*inodorus*
CM82	Vert Grimmpant Melon	*cantalupensis*
CM83	Yellow Canary Melon	*inodorus*
CM84	Zatta Melon	*cantalupensis*
CM85	Old Original Israeli Melon	*cantalupensis*
CM86	Pride of Wisconsin Melon	*cantalupensis*
CM87	Queen Anne Pocket	*dudaim*
CM88	Rocky Ford Green Flesh	*cantalupensis*
CM89	Sweet Passion Melon	*cantalupensis*
CM90	Top Mark Melon	*cantalupensis*

S.No	Accsession Name	Group
CM91	CGN20821	*flexuosus*
CM92	CGN23412	*flexuosus*
CM93	CGN23623	*conomon*
CM94	CGN24609	*conomon*
CM95	CGN24616	*flexuosus*
P1	MR-1	*momordica*
P2	Hale's Best Jumbo	*cantalupensis*
