# Supplementary material for: Genome-Wide Differentiation of Various Melon Horticultural Groups for Use in GWAS for Fruit Firmness and Construction of a High Resolution Genetic Map
Source: Front Plant Sci. 2016 Sep 22;7:1437. doi: 10.3389/fpls.2016.01437 (PMC5031849; doi:10.3389/fpls.2016.01437)
Supplement: Table S2 — Fruit pressure across various years and locations. [file Table2.PDF]

Table S2: Fruit pressure across various years and locations.

Acession Number	WVSU13	ASU13	WVSU14	ASU14
CM1	–	–	–	–
CM2	2.39	1.81	–	1.21
CM3	–	–	–	–
CM4	–	0.87	1.09	1.9
CM5	–	0.96	0.53	0.47
CM6	–	0.96	1.29	1.73
CM7	–	0.56	–	0.9
CM8	–	0.52	0.68	0.5
CM9	1.05	–	1.22	1.21
CM10	2.9	1.1	1.68	1.89
CM11	1.54	1.4	1.3	0.95
CM12	–	1.14	1.48	1.8
CM13	1.97	2.18	0.88	1.92
CM14	1.41	1.19	1.71	1.8
CM15	1.77	1.22	–	1.36
CM16	2.21	2.04	–	2.39
CM17	2.11	–	–	0.96
CM18	–	1.37	0.92	0.95
CM19	–	0.45	–	0.86
CM20	–	0.86	0.81	0.69
CM21	–	1.27	1.35	1.21
CM22	–	–	1.48	0.65
CM23	0.51	0.91	0.59	0.77
CM24	1.17	1.22	0.97	0.82
CM25	2.74	1.08	1.23	1.01
CM26	2.06	1.55	1.03	1.29
CM27	1.23	1.05	1.25	1.01
CM28	0.86	0.67	–	1.11
CM29	1.16	0.73	1.4	1.04
CM30	–	1.09	1.06	0.88
CM31	2.73	1.23	–	1.31
CM32	2.41	1.36	–	1.12
CM33	3.06	1.06	–	1.06
CM34	1.31	0.98	1.47	1.25
CM35	1.24	1.04	1.26	0.83
CM36	1.76	1.33	1.52	1.53
CM37	2.08	–	1.12	0.83
CM38	2.58	–	1.11	1.29
CM39	1.9	1.47	2.16	1.33
CM40	1.47	1.59	1.54	1.65
CM41	1.25	2.33	2.73	1.75
CM42	2.65	2.33	2.21	1.62
CM43	1.56	0.83	0.73	0.61
CM44	1.44	1.36	0.81	1

Acession Number	WVSU13	ASU13	WVSU14	ASU14
CM45	1.61	1.22	1.1	0.87
CM46	0.38	1.43	1.25	1.1
CM47	–	–	0.98	–
CM48	2.1	1.87	1.87	1.23
CM49	1.36	1.28	–	1.75
CM50	1.09	0.54	1.02	0.4
CM51	2.09	–	1.15	0.63
CM52	2.88	–	1.07	0.94
CM53	2.47	1.39	1.93	1.82
CM54	1.48	–	1.99	1.54
CM55	1.24	1.17	1.1	0.96
CM56	1.19	–	1.44	1.33
CM57	2.38	–	1.42	1.34
CM58	1.27	0.88	1.48	1.23
CM59	1.26	–	1.26	1.14
CM60	1.28	1.31	–	1.2
CM61	2.35	1.42	–	1.4
CM62	2.76	1.2	1.15	1.33
CM63	2.07	1.06	1.55	1.19
CM64	0	1.76	1.85	1.98
CM65	2.21	–	1.79	1.85
CM66	0.83	–	–	1.12
CM67	0	–	1.24	0.96
CM68	1.8	1.18	1.28	1.01
CM69	1.55	0.89	1.13	1.04
CM70	2.56	0.97	1.78	0.6
CM71	1.4	1.6	2.23	1.28
CM72	1.59	0.81	0.88	0.93
CM73	1.66	–	–	0.79
CM74	0	0.9	1.38	–
CM75	1.9	1.33	1.76	0.7
CM76	2.71	1.35	2.33	–
CM77	2.42	1.37	1.11	–
CM78	0	–	1.48	0.89
CM79	3.17	–	1.73	1.16
CM80	2.78	2.13	2.53	1.12
CM81	–	–	3.18	–
CM82	1.96	0.96	1.27	0.83
CM83	1.5	1.53	2.06	1.49
CM84	2.83	1.73	1.36	–
CM85	0.65	1.75	–	–
CM86	1.98	1.14	0.92	0.92
CM87	0.78	1.16	1.32	1.13
CM88	1.55	–	–	–
CM89	–	0.98	–	–
CM90	1.96	–	–	–

Acession Number	WVSU13	ASU13	WVSU14	ASU14
CM91	1.97	–	–	–
CM92	–	–	–	–
CM93	1.61	–	–	–
CM94	–	0.56	–	–
CM95	–	0.74	–	–
P1	–	–	–	–
P2	–	–	–	_
