# Supplementary material for: Genome-Wide Differentiation of Various Melon Horticultural Groups for Use in GWAS for Fruit Firmness and Construction of a High Resolution Genetic Map
Source: Front Plant Sci. 2016 Sep 22;7:1437. doi: 10.3389/fpls.2016.01437 (PMC5031849; doi:10.3389/fpls.2016.01437)
Supplement: Table S3 — Fruit pressure distribution in F2 progeny of a cross of MR-1 (momordica) and “Hale's Best Jumbo” (P2), a Western Shipped Cantaloupe. [file Table3.PDF]

Table S3: Fruit pressure distribution in F2 progeny of a cross of MR-1 (momordica) and 'Hale's Best Jumbo' (P2), a Western Shipped Cantaloupe.

S.No	Pressure
1	0.65
2	0.8
3	0.8
4	0.65
5	0.75
6	1
7	0.6
8	1
9	0.8
10	1.1
11	0.5
12	1.65
13	1.2
14	1.6
15	1.1
16	1
17	1.25
18	1
19	1.75
20	0.5
21	0.5
22	0.85
22b	0.8
23	0.5
24	0.52
25	0.6
26	0.8
27	1.5
28	1.2
29	0.8
30	0.6
31	1
32	0.65
33	0.8
34	0.75
35	0.8
36	0.8
37	0.65
38	0.5
39	0.8
40	1.1
41	0.65
42	1.2
43	0.8
44	0.8
45	0.8
46	1
47	0.6
48	0.85
49	1.1
50	0.8
51	0.65
52	–
53	1
54	0.8
55	0.5
56	0.5
57	–
58	0.5
59	0.5
60	1.2
61	–
62	0.55
63	–
64	0.6
65	0.6
66	–
67	0.6
68	0.5
69	1.2
70	1
71	0.65
72	1
73	–
74	–
75	1.1
76	–
77	0.8
78	–
79	1.1
80	–
81	–
82	1
83	–
84	–
85	–
86	–
87	1.2
88	1
100	1.8
101	0.7
102	0.5
103	1.2
104	–
105	1.2
106	0.8
107	–
108	–
109	1.2
110	1
111	–
112	1
113	1.35
P1	0.5
P2	1.8
