# Supplementary material for: Genome-Wide Differentiation of Various Melon Horticultural Groups for Use in GWAS for Fruit Firmness and Construction of a High Resolution Genetic Map
Source: Front Plant Sci. 2016 Sep 22;7:1437. doi: 10.3389/fpls.2016.01437 (PMC5031849; doi:10.3389/fpls.2016.01437)
Supplement: Table S4 — Eigen values for the first 10 principle components estimated for various accessions. [file Table4.PDF]

Table S4: Eigen values for the first ten principle components estimated for various accessions.

| Accession Name | EV = 20.7792 | EV = 7.26617 | EV = 3.67222 |
|---|---|---|---|
| CM1 | -0.04227475 | -0.14024782 | -0.00245955 |
| CM2 | -0.051121149 | -0.164166809 | -0.098031816 |
| CM3 | -0.052603022 | 0.080407095 | -0.026219969 |
| CM4 | -0.045890519 | -0.121683479 | -0.025244571 |
| CM5 | 0.301213492 | 0.032147077 | -0.144487911 |
| CM6 | -0.053458567 | -0.157886164 | -0.084615509 |
| CM7 | -0.022576733 | 0.01686813 | 0.031282218 |
| CM8 | -0.024229063 | -0.061624331 | 0.043534121 |
| CM9 | -0.049160153 | -0.060114975 | -0.044968163 |
| CM10 | -0.055139672 | -0.163937011 | -0.086898008 |
| CM11 | -0.057246366 | 0.135615847 | -0.014465622 |
| CM12 | -0.047885055 | -0.175652504 | -0.05925205 |
| CM13 | -0.053828073 | -0.155953542 | -0.084368963 |
| CM14 | -0.039700188 | 0.050126836 | -0.013001918 |
| CM15 | -0.041920966 | 0.090916992 | -0.017138126 |
| CM16 | -0.038918182 | -0.13320672 | -0.035846296 |
| CM17 | -0.05037831 | -0.067601049 | -0.0374188 |
| CM18 | -0.05259861 | -0.174204446 | -0.056107156 |
| CM19 | -0.053277291 | -0.17651941 | -0.056216852 |
| CM20 | -0.028504584 | 0.001866794 | 0.018809197 |
| CM21 | -0.026187623 | 0.025354994 | 0.02263731 |
| CM22 | -0.054026482 | 0.105102505 | -0.010554311 |
| CM23 | -0.041996933 | 0.096532315 | -0.0055667 |
| CM24 | 0.299354984 | 0.033460601 | -0.157267883 |
| CM25 | -0.056300908 | 0.136236645 | -0.012197274 |
| CM26 | -0.028040979 | 0.061847335 | -0.003602376 |
| CM27 | -0.053033833 | 0.111423921 | -0.004193484 |
| CM28 | -0.055333995 | 0.130062231 | -0.011725037 |
| CM29 | -0.02844936 | -0.070031159 | 0.019774251 |
| CM30 | 0.295981295 | 0.032610365 | -0.132499339 |
| CM31 | 0.303379092 | 0.034265602 | -0.14916871 |
| CM32 | -0.049414601 | 0.113408561 | -0.013589087 |
| CM33 | 0.300235125 | 0.026680775 | -0.154571619 |
| CM34 | -0.054966137 | 0.12994232 | -0.010315581 |
| CM35 | -0.055593923 | 0.124850757 | -0.012200745 |
| CM36 | -0.052052407 | 0.115175978 | -0.009691645 |
| CM37 | -0.046588403 | 0.093388482 | 0.00241408 |
| CM38 | -0.052787362 | 0.120783657 | -0.013506572 |
| CM39 | -0.034951071 | -0.061555035 | 0.004073496 |
| CM40 | -0.055284686 | 0.10917704 | -0.018368615 |
| CM41 | -0.059923556 | -0.077382121 | -0.070731982 |
| CM42 | -0.050963835 | -0.13680897 | -0.076759024 |
| CM43 | 0.094164924 | 0.001690453 | 0.150772649 |

| Accession Name | EV = 20.7792 | EV = 7.26617 | EV = 3.67222 |
|---|---|---|---|
| CM44 | -0.057707808 | 0.142918916 | -0.014648932 |
| CM45 | -0.050599326 | 0.115083068 | -0.014071847 |
| CM46 | -0.053693062 | 0.108087174 | -0.013152454 |
| CM47 | -0.044185483 | 0.061637565 | -0.019990915 |
| CM48 | 0.104188608 | -0.04457527 | 0.322357246 |
| CM49 | -0.028415294 | 0.024056187 | 0.004234483 |
| CM50 | -0.006400877 | -0.095231515 | 0.080919172 |
| CM51 | -0.034052624 | -0.11430616 | 0.05443043 |
| CM52 | -0.030347848 | 0.017892035 | 0.027640802 |
| CM53 | -0.0528047 | 0.071009718 | -0.00104288 |
| CM54 | 0.102020874 | -0.053365585 | 0.35597723 |
| CM55 | 0.306125369 | 0.03440116 | -0.157599872 |
| CM56 | -0.02566832 | 0.004183465 | 0.031767725 |
| CM57 | -0.032367427 | 0.028996446 | 0.002724646 |
| CM58 | -0.05300767 | 0.10475598 | -0.015254299 |
| CM59 | -0.037009384 | 0.046863888 | 0.014575766 |
| CM60 | -0.04072477 | 0.001750351 | -0.000417076 |
| CM61 | -0.039868421 | 0.073725755 | -0.01040444 |
| CM62 | -0.039478436 | 0.062368546 | -0.008198629 |
| CM63 | -0.032560025 | 0.024735549 | 0.018857884 |
| CM64 | -0.054178887 | -0.180256978 | -0.108554165 |
| CM65 | -0.057906336 | 0.139301946 | -0.014122994 |
| CM66 | 0.12990727 | -0.045388705 | 0.418366675 |
| CM67 | -0.030020003 | -0.005482983 | 0.031905436 |
| CM68 | -0.057380165 | 0.141523416 | -0.014343586 |
| CM69 | 0.097971546 | -0.054957393 | 0.140202256 |
| CM70 | 0.197130182 | -0.01151884 | -0.04520848 |
| CM71 | -0.054540674 | -0.17171318 | -0.108622792 |
| CM72 | 0.302180125 | 0.034309738 | -0.160464108 |
| CM73 | -0.052433001 | 0.117112114 | -0.008754733 |
| CM74 | -0.039616819 | -0.114584872 | 0.004464291 |
| CM75 | -0.042479499 | -0.069326567 | -0.044144205 |
| CM76 | -0.054484998 | -0.176428948 | -0.104471794 |
| CM77 | 0.242760379 | 0.00022274 | 0.017506952 |
| CM78 | 0.05978887 | -0.048375266 | 0.082875511 |
| CM79 | -0.053552523 | 0.107029296 | -0.007179197 |
| CM80 | -0.035263258 | -0.130779801 | -0.018010644 |
| CM81 | -0.055733715 | -0.176089657 | -0.100513667 |
| CM82 | -0.054699741 | 0.10784518 | -0.01983302 |
| CM83 | -0.052681184 | -0.163841071 | -0.102886969 |
| CM84 | -0.029322017 | 0.001181897 | 0.01778642 |
| CM85 | -0.038327598 | -0.118730093 | -0.010698343 |
| CM86 | -0.047560644 | 0.105666165 | -0.006316789 |
| CM87 | 0.130344029 | -0.054216417 | 0.414056674 |
| CM88 | -0.0566848 | 0.131621093 | -0.0131582 |

| Accession Name | EV = 20.7792 | EV = 7.26617 | EV = 3.67222 |
| --- | --- | --- | --- |
| CM89 | -0.055141313 | 0.133549244 | -0.010219681 |
| CM90 | -0.054813163 | 0.139659058 | -0.01616208 |
| CM91 | -0.03201675 | 0.033561404 | 0.018852826 |
| CM92 | -0.019305687 | -0.111311246 | 0.103244207 |
| CM93 | 0.156556086 | -0.031499068 | 0.173105377 |
| CM94 | 0.03606477 | -0.06459642 | 0.142101792 |
| CM95 | -0.01344742 | -0.105101242 | 0.101742875 |
| P1 | -0.007499764 | 0.026504771 | 0.039228635 |
| P2 | -0.034778242 | 0.088755645 | 0.00947542 |
