# Supplementary material for: Genome-Wide Differentiation of Various Melon Horticultural Groups for Use in GWAS for Fruit Firmness and Construction of a High Resolution Genetic Map
Source: Front Plant Sci. 2016 Sep 22;7:1437. doi: 10.3389/fpls.2016.01437 (PMC5031849; doi:10.3389/fpls.2016.01437)
Supplement: Table S5 — Haplotype blocks across entire melon collection in the study. [file Table5.PDF]

Table S5: Haplotype blocks across entire melon collection in the study

Markers	Chromosome	Position	Block #
S1_410695	1	410695	1
S1_422883	1	422883	1
S1_445936	1	445936	2
S1_445937	1	445937	2
S1_445938	1	445938	2
S1_967688	1	967688	3
S1_967706	1	967706	3
S1_1088695	1	1088695	4
S1_1088697	1	1088697	4
S1_1088706	1	1088706	4
S1_1095486	1	1095486	5
S1_1095493	1	1095493	5
S1_1474681	1	1474681	6
S1_1474748	1	1474748	6
S1_1821158	1	1821158	7
S1_1821191	1	1821191	7
S1_1959150	1	1959150	8
S1_1959340	1	1959340	8
S1_1978816	1	1978816	9
S1_1978820	1	1978820	9
S1_2065378	1	2065378	10
S1_2065379	1	2065379	10
S1_2065380	1	2065380	10
S1_2065431	1	2065431	11
S1_2065432	1	2065432	11
S1_2065433	1	2065433	11
S1_2065434	1	2065434	11
S1_2066702	1	2066702	12
S1_2077108	1	2077108	12
S1_2277582	1	2277582	13
S1_2279144	1	2279144	13
S1_2389920	1	2389920	14
S1_2389962	1	2389962	14
S1_2439081	1	2439081	15
S1_2469547	1	2469547	15
S1_2476259	1	2476259	16
S1_2499198	1	2499198	16
S1_2553940	1	2553940	16
S1_2591201	1	2591201	16
S1_2629086	1	2629086	16
S1_2629173	1	2629173	16
S1_2711268	1	2711268	17
S1_2821526	1	2821526	17

Markers	Chromosome	Position	Block #
S1_2841728	1	2841728	18
S1_2841729	1	2841729	18
S1_2841890	1	2841890	18
S1_2866270	1	2866270	19
S1_2866432	1	2866432	19
S1_2866450	1	2866450	20
S1_2868703	1	2868703	20
S1_2872204	1	2872204	20
S1_3039283	1	3039283	21
S1_3039301	1	3039301	21
S1_3181368	1	3181368	22
S1_3238807	1	3238807	22
S1_3248561	1	3248561	23
S1_3248562	1	3248562	23
S1_3338743	1	3338743	24
S1_3346163	1	3346163	24
S1_4029973	1	4029973	25
S1_4029989	1	4029989	25
S1_4037334	1	4037334	26
S1_4037474	1	4037474	26
S1_4177848	1	4177848	27
S1_4201369	1	4201369	27
S1_4208168	1	4208168	27
S1_4327126	1	4327126	28
S1_4327200	1	4327200	28
S1_5050434	1	5050434	29
S1_5059593	1	5059593	29
S1_5098507	1	5098507	29
S1_5199990	1	5199990	30
S1_5200948	1	5200948	30
S1_5885779	1	5885779	31
S1_5910878	1	5910878	31
S1_5910882	1	5910882	31
S1_5922191	1	5922191	31
S1_6319033	1	6319033	32
S1_6319292	1	6319292	32
S1_6373106	1	6373106	33
S1_6373107	1	6373107	33
S1_6483432	1	6483432	34
S1_6502853	1	6502853	34
S1_7316126	1	7316126	35
S1_7316128	1	7316128	35
S1_7316129	1	7316129	35
S1_7316130	1	7316130	36
S1_7316131	1	7316131	36
S1_7600588	1	7600588	37

Markers	Chromosome	Position	Block #
S1_7616769	1	7616769	37
S1_7950048	1	7950048	38
S1_7950165	1	7950165	38
S1_8748654	1	8748654	39
S1_8748726	1	8748726	39
S1_8774902	1	8774902	39
S1_8828772	1	8828772	39
S1_8858478	1	8858478	40
S1_8999772	1	8999772	40
S1_9229580	1	9229580	41
S1_9229624	1	9229624	41
S1_9917532	1	9917532	42
S1_9917565	1	9917565	42
S1_10072577	1	10072577	43
S1_10154387	1	10154387	43
S1_10186966	1	10186966	44
S1_10186973	1	10186973	44
S1_10473985	1	10473985	45
S1_10473987	1	10473987	45
S1_10763657	1	10763657	46
S1_10763668	1	10763668	46
S1_10769356	1	10769356	47
S1_10769368	1	10769368	47
S1_10958959	1	10958959	48
S1_10967208	1	10967208	48
S1_11157882	1	11157882	49
S1_11159715	1	11159715	49
S1_11297264	1	11297264	50
S1_11331929	1	11331929	50
S1_11642270	1	11642270	51
S1_11651112	1	11651112	51
S1_11668343	1	11668343	51
S1_11934548	1	11934548	52
S1_11934629	1	11934629	52
S1_11938633	1	11938633	53
S1_11938634	1	11938634	53
S1_12382636	1	12382636	54
S1_12382669	1	12382669	54
S1_12393771	1	12393771	55
S1_12397960	1	12397960	55
S1_12405774	1	12405774	56
S1_12405804	1	12405804	56
S1_12490090	1	12490090	57
S1_12490091	1	12490091	57
S1_12490101	1	12490101	57
S1_12490144	1	12490144	58

Markers	Chromosome	Position	Block #
S1_12490251	1	12490251	58
S1_12490287	1	12490287	59
S1_12531680	1	12531680	59
S1_12543226	1	12543226	60
S1_12543318	1	12543318	60
S1_12672535	1	12672535	61
S1_12783334	1	12783334	61
S1_13237927	1	13237927	62
S1_13273564	1	13273564	62
S1_13273592	1	13273592	62
S1_13316490	1	13316490	62
S1_13316491	1	13316491	62
S1_13316617	1	13316617	62
S1_13592388	1	13592388	63
S1_13592600	1	13592600	63
S1_13679723	1	13679723	64
S1_13679730	1	13679730	64
S1_14508867	1	14508867	65
S1_14508874	1	14508874	65
S1_14847050	1	14847050	66
S1_14852228	1	14852228	66
S1_14852237	1	14852237	67
S1_14852281	1	14852281	67
S1_15789964	1	15789964	68
S1_15790004	1	15790004	68
S1_15850510	1	15850510	69
S1_15850721	1	15850721	69
S1_15961435	1	15961435	70
S1_15984481	1	15984481	70
S1_15986136	1	15986136	70
S1_15986209	1	15986209	70
S1_16164203	1	16164203	71
S1_16180513	1	16180513	71
S1_16182249	1	16182249	71
S1_16448884	1	16448884	72
S1_16448908	1	16448908	72
S1_16638975	1	16638975	73
S1_16638978	1	16638978	73
S1_16639010	1	16639010	73
S1_16639013	1	16639013	73
S1_17051411	1	17051411	74
S1_17061788	1	17061788	74
S1_17061963	1	17061963	75
S1_17061967	1	17061967	75
S1_17599369	1	17599369	76
S1_17599370	1	17599370	76

Markers	Chromosome	Position	Block #
S1_17599371	1	17599371	76
S1_19344198	1	19344198	77
S1_19344240	1	19344240	77
S1_19426454	1	19426454	78
S1_19433819	1	19433819	78
S1_19433984	1	19433984	79
S1_19433996	1	19433996	79
S1_19629435	1	19629435	80
S1_19629537	1	19629537	80
S1_19651791	1	19651791	80
S1_19844177	1	19844177	81
S1_19844260	1	19844260	81
S1_19877622	1	19877622	81
S1_19877652	1	19877652	81
S1_19887160	1	19887160	81
S1_20051010	1	20051010	82
S1_20101090	1	20101090	82
S1_20101123	1	20101123	82
S1_20101125	1	20101125	82
S1_20101180	1	20101180	82
S1_20128996	1	20128996	82
S1_20129013	1	20129013	82
S1_20129057	1	20129057	82
S1_20324887	1	20324887	83
S1_20324890	1	20324890	83
S1_20324893	1	20324893	83
S1_20324894	1	20324894	83
S1_20324895	1	20324895	83
S1_20332951	1	20332951	83
S1_20333007	1	20333007	83
S1_20419824	1	20419824	83
S1_20438230	1	20438230	83
S1_20744988	1	20744988	84
S1_20753018	1	20753018	84
S1_20753240	1	20753240	84
S1_20753428	1	20753428	85
S1_20815715	1	20815715	85
S1_20996968	1	20996968	86
S1_21002359	1	21002359	86
S1_21021981	1	21021981	87
S1_21022029	1	21022029	87
S1_21615713	1	21615713	88
S1_21615727	1	21615727	88
S1_21976007	1	21976007	89
S1_21980717	1	21980717	89
S1_21980740	1	21980740	89

Markers	Chromosome	Position	Block #
S1_22277483	1	22277483	90
S1_22298582	1	22298582	90
S1_22620761	1	22620761	91
S1_22620762	1	22620762	91
S1_22620763	1	22620763	91
S1_22635905	1	22635905	92
S1_22669192	1	22669192	92
S1_22669541	1	22669541	92
S1_22695261	1	22695261	92
S1_22695291	1	22695291	92
S1_22695314	1	22695314	92
S1_22695339	1	22695339	92
S1_22987914	1	22987914	93
S1_22987949	1	22987949	93
S1_22988151	1	22988151	93
S1_23116440	1	23116440	93
S1_25191928	1	25191928	94
S1_25191930	1	25191930	94
S1_25191933	1	25191933	94
S1_25192008	1	25192008	94
S1_25570198	1	25570198	95
S1_25571483	1	25571483	95
S1_25746889	1	25746889	96
S1_25755285	1	25755285	96
S1_25955137	1	25955137	97
S1_25955609	1	25955609	97
S1_25962057	1	25962057	97
S1_25962058	1	25962058	97
S1_25962371	1	25962371	97
S1_26348807	1	26348807	98
S1_26410718	1	26410718	98
S1_26411546	1	26411546	98
S1_26531567	1	26531567	99
S1_26548533	1	26548533	99
S1_27113206	1	27113206	100
S1_27134578	1	27134578	100
S1_27555109	1	27555109	101
S1_27556580	1	27556580	101
S1_27560182	1	27560182	101
S1_27560257	1	27560257	102
S1_27613340	1	27613340	102
S1_27613343	1	27613343	102
S1_27938304	1	27938304	103
S1_27938305	1	27938305	103
S1_27945039	1	27945039	103
S1_27945136	1	27945136	103

Markers	Chromosome	Position	Block #
S1_27951804	1	27951804	104
S1_27951805	1	27951805	104
S1_27951808	1	27951808	104
S1_27951842	1	27951842	105
S1_28034804	1	28034804	105
S1_28269579	1	28269579	106
S1_28269602	1	28269602	106
S1_28269680	1	28269680	106
S1_28625558	1	28625558	107
S1_28625559	1	28625559	107
S1_28625560	1	28625560	107
S1_28625656	1	28625656	108
S1_28682987	1	28682987	108
S1_28861268	1	28861268	109
S1_29012469	1	29012469	109
S1_29128331	1	29128331	110
S1_29205773	1	29205773	110
S1_29215710	1	29215710	110
S1_29283385	1	29283385	111
S1_29283393	1	29283393	111
S1_29466386	1	29466386	112
S1_29467858	1	29467858	112
S1_29467885	1	29467885	112
S1_29867805	1	29867805	113
S1_29874017	1	29874017	113
S1_29874020	1	29874020	114
S1_29874085	1	29874085	114
S1_30427528	1	30427528	115
S1_30427573	1	30427573	115
S1_30427675	1	30427675	115
S1_30427677	1	30427677	115
S1_30427867	1	30427867	115
S1_30427876	1	30427876	115
S1_30428799	1	30428799	115
S1_30428823	1	30428823	115
S1_30428853	1	30428853	115
S1_30428977	1	30428977	115
S1_30428983	1	30428983	115
S1_30429008	1	30429008	115
S1_30432198	1	30432198	115
S1_30432212	1	30432212	115
S1_30432247	1	30432247	115
S1_30636173	1	30636173	116
S1_30636178	1	30636178	116
S1_30909295	1	30909295	117
S1_30909296	1	30909296	117

Markers	Chromosome	Position	Block #
S1_30942620	1	30942620	118
S1_30942635	1	30942635	118
S1_31148968	1	31148968	119
S1_31148978	1	31148978	119
S1_31149021	1	31149021	119
S1_31179299	1	31179299	120
S1_31179302	1	31179302	120
S1_31422987	1	31422987	121
S1_31423008	1	31423008	121
S1_31453983	1	31453983	122
S1_31464678	1	31464678	122
S1_31616406	1	31616406	123
S1_31616430	1	31616430	123
S1_31651969	1	31651969	124
S1_31652001	1	31652001	124
S1_31652002	1	31652002	124
S1_31735826	1	31735826	125
S1_31736671	1	31736671	125
S1_31736707	1	31736707	125
S1_31843785	1	31843785	126
S1_31843786	1	31843786	126
S1_31925260	1	31925260	127
S1_31928764	1	31928764	127
S1_32057413	1	32057413	128
S1_32065430	1	32065430	128
S1_32070366	1	32070366	128
S1_32070504	1	32070504	128
S1_32070527	1	32070527	128
S1_32070533	1	32070533	128
S1_32118738	1	32118738	129
S1_32118739	1	32118739	129
S1_32118751	1	32118751	129
S1_32118763	1	32118763	129
S1_32118765	1	32118765	129
S1_32133410	1	32133410	130
S1_32133589	1	32133589	130
S1_32186259	1	32186259	131
S1_32186278	1	32186278	131
S1_32186408	1	32186408	131
S1_32217015	1	32217015	132
S1_32217025	1	32217025	132
S1_32323545	1	32323545	133
S1_32323548	1	32323548	133
S1_32392522	1	32392522	134
S1_32392554	1	32392554	134
S1_32443379	1	32443379	135

Markers	Chromosome	Position	Block #
S1_32443670	1	32443670	135
S1_32484151	1	32484151	136
S1_32484226	1	32484226	136
S1_32561673	1	32561673	137
S1_32561726	1	32561726	137
S1_32605230	1	32605230	137
S1_32633744	1	32633744	138
S1_32633756	1	32633756	138
S1_32633761	1	32633761	138
S1_32633768	1	32633768	138
S1_32736752	1	32736752	139
S1_32737027	1	32737027	139
S1_32737051	1	32737051	139
S1_33435266	1	33435266	140
S1_33437852	1	33437852	140
S1_33442450	1	33442450	140
S1_33551958	1	33551958	141
S1_33552004	1	33552004	141
S1_33552005	1	33552005	141
S1_33552045	1	33552045	141
S1_33552049	1	33552049	141
S1_33552077	1	33552077	141
S1_33552138	1	33552138	141
S1_33591110	1	33591110	142
S1_33602641	1	33602641	142
S1_33602645	1	33602645	142
S1_33602724	1	33602724	143
S1_33605389	1	33605389	143
S1_33605727	1	33605727	143
S1_33633943	1	33633943	143
S1_33694591	1	33694591	144
S1_33694662	1	33694662	144
S1_33792208	1	33792208	145
S1_33792292	1	33792292	145
S1_34026945	1	34026945	146
S1_34027052	1	34027052	146
S1_34051307	1	34051307	147
S1_34051326	1	34051326	147
S1_34289744	1	34289744	148
S1_34297341	1	34297341	148
S1_34309486	1	34309486	148
S1_34311423	1	34311423	148
S1_34311605	1	34311605	148
S1_34311734	1	34311734	148
S1_34311810	1	34311810	148
S1_34311811	1	34311811	148

Markers	Chromosome	Position	Block #
S1_34506525	1	34506525	149
S1_34506529	1	34506529	149
S1_34567655	1	34567655	150
S1_34569144	1	34569144	150
S1_34851217	1	34851217	151
S1_34859222	1	34859222	151
S1_34990762	1	34990762	152
S1_34990793	1	34990793	152
S1_34999530	1	34999530	153
S1_34999589	1	34999589	153
S1_35123513	1	35123513	154
S1_35127217	1	35127217	154
S1_35156294	1	35156294	154
S1_35156452	1	35156452	154
S1_35181663	1	35181663	154
S1_35229479	1	35229479	154
S2_146775	2	146775	155
S2_164392	2	164392	155
S2_170681	2	170681	156
S2_171317	2	171317	156
S2_247209	2	247209	157
S2_305110	2	305110	157
S2_308272	2	308272	157
S2_308300	2	308300	157
S2_360780	2	360780	157
S2_554462	2	554462	158
S2_554483	2	554483	158
S2_554484	2	554484	158
S2_589764	2	589764	159
S2_589765	2	589765	159
S2_695358	2	695358	160
S2_695427	2	695427	160
S2_695485	2	695485	161
S2_695534	2	695534	161
S2_1063431	2	1063431	162
S2_1063446	2	1063446	162
S2_1063458	2	1063458	162
S2_1591748	2	1591748	163
S2_1592034	2	1592034	163
S2_1621015	2	1621015	164
S2_1621032	2	1621032	164
S2_1621033	2	1621033	164
S2_1621068	2	1621068	164
S2_1623351	2	1623351	165
S2_1679255	2	1679255	165
S2_1679268	2	1679268	166

Markers	Chromosome	Position	Block #
S2_1679269	2	1679269	166
S2_1679354	2	1679354	167
S2_1680344	2	1680344	167
S2_1805157	2	1805157	168
S2_1805170	2	1805170	168
S2_1805185	2	1805185	168
S2_1805471	2	1805471	169
S2_1805472	2	1805472	169
S2_1805473	2	1805473	169
S2_1805474	2	1805474	169
S2_1805484	2	1805484	170
S2_1805524	2	1805524	170
S2_1819256	2	1819256	170
S2_1834327	2	1834327	170
S2_1891052	2	1891052	170
S2_2601269	2	2601269	171
S2_2601333	2	2601333	171
S2_3107847	2	3107847	172
S2_3109092	2	3109092	172
S2_3109168	2	3109168	173
S2_3109169	2	3109169	173
S2_3128830	2	3128830	174
S2_3128852	2	3128852	174
S2_3128855	2	3128855	174
S2_3238537	2	3238537	175
S2_3238550	2	3238550	175
S2_4178031	2	4178031	176
S2_4185514	2	4185514	176
S2_4543452	2	4543452	177
S2_4543504	2	4543504	177
S2_4543608	2	4543608	177
S2_5214330	2	5214330	178
S2_5214348	2	5214348	178
S2_5338140	2	5338140	179
S2_5338280	2	5338280	179
S2_5338373	2	5338373	179
S2_5338413	2	5338413	179
S2_5360639	2	5360639	179
S2_5360640	2	5360640	179
S2_5389237	2	5389237	179
S2_5390442	2	5390442	179
S2_5895586	2	5895586	180
S2_5895727	2	5895727	180
S2_6248554	2	6248554	181
S2_6250775	2	6250775	181
S2_7279885	2	7279885	182

Markers	Chromosome	Position	Block #
S2_7280861	2	7280861	182
S2_7281378	2	7281378	182
S2_7292914	2	7292914	182
S2_7368740	2	7368740	183
S2_7375537	2	7375537	183
S2_7605964	2	7605964	184
S2_7644352	2	7644352	184
S2_7819145	2	7819145	185
S2_7819165	2	7819165	185
S2_8591619	2	8591619	186
S2_8591626	2	8591626	186
S2_8595851	2	8595851	186
S2_8595864	2	8595864	187
S2_8595888	2	8595888	187
S2_8752307	2	8752307	188
S2_8752347	2	8752347	188
S2_8756273	2	8756273	188
S2_8895643	2	8895643	189
S2_8975324	2	8975324	189
S2_8994193	2	8994193	189
S2_9591890	2	9591890	190
S2_9591941	2	9591941	190
S2_10561529	2	10561529	191
S2_10561537	2	10561537	191
S2_10829634	2	10829634	192
S2_10829637	2	10829637	192
S2_12207164	2	12207164	193
S2_12207203	2	12207203	193
S2_12208474	2	12208474	193
S2_12249486	2	12249486	193
S2_12288666	2	12288666	193
S2_12353175	2	12353175	193
S2_12353432	2	12353432	194
S2_12370274	2	12370274	194
S2_12522799	2	12522799	195
S2_12602347	2	12602347	195
S2_12778543	2	12778543	196
S2_12785838	2	12785838	196
S2_12829503	2	12829503	197
S2_12964911	2	12964911	197
S2_12970708	2	12970708	197
S2_12970871	2	12970871	197
S2_12970875	2	12970875	197
S2_12972854	2	12972854	197
S2_13295265	2	13295265	198
S2_13295285	2	13295285	198

Markers	Chromosome	Position	Block #
S2_13295303	2	13295303	198
S2_13296167	2	13296167	198
S2_13298685	2	13298685	198
S2_13792789	2	13792789	199
S2_13792800	2	13792800	199
S2_13799066	2	13799066	199
S2_13803586	2	13803586	199
S2_13803595	2	13803595	199
S2_14068379	2	14068379	200
S2_14071295	2	14071295	200
S2_14093339	2	14093339	200
S2_14104172	2	14104172	200
S2_14109274	2	14109274	200
S2_14229246	2	14229246	201
S2_14232689	2	14232689	201
S2_14604518	2	14604518	202
S2_14606566	2	14606566	202
S2_14616378	2	14616378	203
S2_14765349	2	14765349	203
S2_14766131	2	14766131	203
S2_14786447	2	14786447	204
S2_14786458	2	14786458	204
S2_14969757	2	14969757	205
S2_14969796	2	14969796	205
S2_14969845	2	14969845	206
S2_14969882	2	14969882	206
S2_14996180	2	14996180	207
S2_14997885	2	14997885	207
S2_14997911	2	14997911	207
S2_15202307	2	15202307	208
S2_15202638	2	15202638	208
S2_15369460	2	15369460	209
S2_15370079	2	15370079	209
S2_15370110	2	15370110	210
S2_15370356	2	15370356	210
S2_15370373	2	15370373	211
S2_15370385	2	15370385	211
S2_15418078	2	15418078	212
S2_15418089	2	15418089	212
S2_15448280	2	15448280	213
S2_15448306	2	15448306	213
S2_15537282	2	15537282	214
S2_15556180	2	15556180	214
S2_15556647	2	15556647	214
S2_15816858	2	15816858	215
S2_15816859	2	15816859	215

Markers	Chromosome	Position	Block #
S2_15884922	2	15884922	216
S2_15962857	2	15962857	216
S2_16078517	2	16078517	217
S2_16138325	2	16138325	217
S2_16261690	2	16261690	218
S2_16286568	2	16286568	218
S2_16301414	2	16301414	219
S2_16301437	2	16301437	219
S2_16301449	2	16301449	219
S2_16301450	2	16301450	219
S2_17163044	2	17163044	220
S2_17229030	2	17229030	220
S2_17337396	2	17337396	221
S2_17337397	2	17337397	221
S2_17389425	2	17389425	222
S2_17389426	2	17389426	222
S2_17436044	2	17436044	223
S2_17436149	2	17436149	223
S2_17663959	2	17663959	224
S2_17699772	2	17699772	224
S2_17762155	2	17762155	225
S2_17834292	2	17834292	225
S2_17936204	2	17936204	226
S2_17951450	2	17951450	226
S2_17951500	2	17951500	227
S2_17951511	2	17951511	227
S2_18205263	2	18205263	228
S2_18205264	2	18205264	228
S2_18205265	2	18205265	228
S2_18429953	2	18429953	229
S2_18430166	2	18430166	229
S2_18522406	2	18522406	230
S2_18522407	2	18522407	230
S2_18522449	2	18522449	230
S2_18529828	2	18529828	231
S2_18536671	2	18536671	231
S2_18536677	2	18536677	231
S2_18756157	2	18756157	232
S2_18839996	2	18839996	232
S2_18879765	2	18879765	233
S2_18908380	2	18908380	233
S2_18908477	2	18908477	233
S2_18916010	2	18916010	234
S2_18916042	2	18916042	234
S2_19076456	2	19076456	235
S2_19079925	2	19079925	235

Markers	Chromosome	Position	Block #
S2_20453230	2	20453230	236
S2_20453240	2	20453240	236
S2_20505073	2	20505073	237
S2_20505192	2	20505192	237
S2_20791297	2	20791297	238
S2_20834219	2	20834219	238
S2_20870112	2	20870112	238
S2_20870192	2	20870192	238
S2_20871185	2	20871185	238
S2_20875690	2	20875690	238
S2_20875718	2	20875718	238
S2_20875730	2	20875730	238
S2_20891968	2	20891968	238
S2_20974603	2	20974603	239
S2_20974604	2	20974604	239
S2_21209068	2	21209068	240
S2_21209077	2	21209077	240
S2_21209102	2	21209102	240
S2_21209159	2	21209159	240
S2_21209171	2	21209171	240
S2_21298758	2	21298758	241
S2_21300090	2	21300090	241
S2_21339215	2	21339215	242
S2_21358372	2	21358372	242
S2_21358902	2	21358902	242
S2_21368011	2	21368011	243
S2_21368014	2	21368014	243
S2_21522250	2	21522250	244
S2_21522263	2	21522263	244
S2_21525689	2	21525689	244
S2_21695763	2	21695763	245
S2_21705052	2	21705052	245
S2_21720246	2	21720246	245
S2_21720264	2	21720264	245
S2_21720268	2	21720268	245
S2_21915648	2	21915648	246
S2_21915649	2	21915649	246
S2_21915651	2	21915651	247
S2_21915652	2	21915652	247
S2_22116732	2	22116732	248
S2_22116751	2	22116751	248
S2_22116753	2	22116753	248
S2_22116754	2	22116754	248
S2_22116887	2	22116887	248
S2_22116934	2	22116934	248
S2_22117028	2	22117028	248

Markers	Chromosome	Position	Block #
S2_22117081	2	22117081	248
S2_22447252	2	22447252	249
S2_22447314	2	22447314	249
S2_22562671	2	22562671	250
S2_22562761	2	22562761	250
S2_22567330	2	22567330	251
S2_22567357	2	22567357	251
S2_22634067	2	22634067	251
S2_22639434	2	22639434	251
S2_22693495	2	22693495	251
S2_22695714	2	22695714	252
S2_22695722	2	22695722	252
S2_22695793	2	22695793	253
S2_22780924	2	22780924	253
S2_22781086	2	22781086	253
S2_22786333	2	22786333	254
S2_22786363	2	22786363	254
S2_22975263	2	22975263	255
S2_22975304	2	22975304	255
S2_22975325	2	22975325	255
S2_22975340	2	22975340	255
S2_23239618	2	23239618	256
S2_23248224	2	23248224	256
S2_23248351	2	23248351	256
S2_23248361	2	23248361	256
S2_23248391	2	23248391	256
S2_23248446	2	23248446	256
S2_23248458	2	23248458	256
S2_23248515	2	23248515	256
S2_23312722	2	23312722	257
S2_23312725	2	23312725	257
S2_23326141	2	23326141	258
S2_23327238	2	23327238	258
S2_23327334	2	23327334	258
S2_23327971	2	23327971	258
S2_23336249	2	23336249	259
S2_23336286	2	23336286	259
S2_23340093	2	23340093	260
S2_23340154	2	23340154	260
S2_23345208	2	23345208	261
S2_23345243	2	23345243	261
S2_23345264	2	23345264	261
S2_23345277	2	23345277	261
S2_23345293	2	23345293	261
S2_23427623	2	23427623	262
S2_23427627	2	23427627	262

Markers	Chromosome	Position	Block #
S2_23428322	2	23428322	262
S2_23442015	2	23442015	263
S2_23443289	2	23443289	263
S2_23485285	2	23485285	264
S2_23486300	2	23486300	264
S2_23559582	2	23559582	265
S2_23576258	2	23576258	265
S2_23617669	2	23617669	265
S2_23617817	2	23617817	265
S2_23617962	2	23617962	265
S2_23617966	2	23617966	265
S2_23617988	2	23617988	265
S2_23618001	2	23618001	265
S2_23769220	2	23769220	266
S2_23769235	2	23769235	266
S2_23771660	2	23771660	266
S2_23782926	2	23782926	266
S2_23873539	2	23873539	267
S2_23873569	2	23873569	267
S2_23954958	2	23954958	268
S2_23976584	2	23976584	268
S2_24052173	2	24052173	269
S2_24054406	2	24054406	269
S2_24054415	2	24054415	269
S2_24170825	2	24170825	270
S2_24180581	2	24180581	270
S2_24208490	2	24208490	270
S2_24332069	2	24332069	271
S2_24346034	2	24346034	271
S2_24359265	2	24359265	272
S2_24359266	2	24359266	272
S2_24448282	2	24448282	273
S2_24533068	2	24533068	273
S2_24554918	2	24554918	273
S2_24555148	2	24555148	273
S2_24574237	2	24574237	273
S3_65075	3	65075	274
S3_67348	3	67348	274
S3_86977	3	86977	275
S3_127260	3	127260	275
S3_199457	3	199457	276
S3_199559	3	199559	276
S3_199560	3	199560	276
S3_219381	3	219381	277
S3_223975	3	223975	277
S3_224195	3	224195	278

Markers	Chromosome	Position	Block #
S3_260821	3	260821	278
S3_490863	3	490863	279
S3_492484	3	492484	279
S3_568915	3	568915	280
S3_569126	3	569126	280
S3_573779	3	573779	281
S3_573838	3	573838	281
S3_573859	3	573859	281
S3_573860	3	573860	281
S3_573862	3	573862	281
S3_688309	3	688309	282
S3_688372	3	688372	282
S3_688816	3	688816	282
S3_689437	3	689437	282
S3_745877	3	745877	283
S3_745888	3	745888	283
S3_930588	3	930588	284
S3_940282	3	940282	284
S3_1015252	3	1015252	285
S3_1100863	3	1100863	285
S3_2038961	3	2038961	286
S3_2039022	3	2039022	286
S3_2291343	3	2291343	287
S3_2291379	3	2291379	287
S3_2792335	3	2792335	288
S3_2792348	3	2792348	288
S3_3025881	3	3025881	289
S3_3025908	3	3025908	289
S3_3382809	3	3382809	290
S3_3389136	3	3389136	290
S3_3389137	3	3389137	290
S3_3389138	3	3389138	290
S3_3439646	3	3439646	291
S3_3439648	3	3439648	291
S3_3439860	3	3439860	292
S3_3439896	3	3439896	292
S3_3466656	3	3466656	293
S3_3466698	3	3466698	293
S3_3523483	3	3523483	294
S3_3523501	3	3523501	294
S3_3638411	3	3638411	295
S3_3639874	3	3639874	295
S3_3639897	3	3639897	295
S3_5525883	3	5525883	296
S3_5525905	3	5525905	296
S3_5525923	3	5525923	296

Markers	Chromosome	Position	Block #
S3_5526628	3	5526628	297
S3_5526668	3	5526668	297
S3_5750431	3	5750431	298
S3_5750432	3	5750432	298
S3_6149988	3	6149988	299
S3_6150214	3	6150214	299
S3_6306421	3	6306421	300
S3_6312154	3	6312154	300
S3_6587312	3	6587312	301
S3_6587321	3	6587321	301
S3_7084179	3	7084179	302
S3_7084232	3	7084232	302
S3_7084320	3	7084320	302
S3_7084323	3	7084323	302
S3_7084324	3	7084324	302
S3_7292180	3	7292180	303
S3_7292201	3	7292201	303
S3_7293644	3	7293644	303
S3_7342864	3	7342864	304
S3_7342931	3	7342931	304
S3_7550886	3	7550886	305
S3_7561562	3	7561562	305
S3_7658304	3	7658304	306
S3_7658320	3	7658320	306
S3_7732233	3	7732233	306
S3_7732321	3	7732321	306
S3_7733662	3	7733662	306
S3_7734012	3	7734012	306
S3_8570119	3	8570119	307
S3_8570122	3	8570122	307
S3_8772894	3	8772894	308
S3_8772896	3	8772896	308
S3_8772900	3	8772900	308
S3_8774897	3	8774897	308
S3_8788140	3	8788140	308
S3_8788261	3	8788261	308
S3_8788263	3	8788263	308
S3_8980131	3	8980131	309
S3_8997780	3	8997780	309
S3_9376259	3	9376259	310
S3_9377029	3	9377029	310
S3_9903356	3	9903356	311
S3_9903974	3	9903974	311
S3_10144486	3	10144486	312
S3_10144493	3	10144493	312
S3_11061295	3	11061295	313

Markers	Chromosome	Position	Block #
S3_11078456	3	11078456	313
S3_11102665	3	11102665	313
S3_11112187	3	11112187	313
S3_14731261	3	14731261	314
S3_14731289	3	14731289	314
S3_14731304	3	14731304	314
S3_14954857	3	14954857	315
S3_14954917	3	14954917	315
S3_15762885	3	15762885	316
S3_15762986	3	15762986	316
S3_15763023	3	15763023	316
S3_15959751	3	15959751	317
S3_15971327	3	15971327	317
S3_15980914	3	15980914	317
S3_16110867	3	16110867	318
S3_16141813	3	16141813	318
S3_16142962	3	16142962	318
S3_16148061	3	16148061	318
S3_17072690	3	17072690	319
S3_17072707	3	17072707	319
S3_17434839	3	17434839	320
S3_17434871	3	17434871	320
S3_17434874	3	17434874	320
S3_17467577	3	17467577	321
S3_17467578	3	17467578	321
S3_17598091	3	17598091	322
S3_17598192	3	17598192	322
S3_17636887	3	17636887	323
S3_17636892	3	17636892	323
S3_17768333	3	17768333	324
S3_17769213	3	17769213	324
S3_17934709	3	17934709	325
S3_17934744	3	17934744	325
S3_17954071	3	17954071	325
S3_17954105	3	17954105	326
S3_17954112	3	17954112	326
S3_17954113	3	17954113	326
S3_17954141	3	17954141	326
S3_18148177	3	18148177	327
S3_18148303	3	18148303	327
S3_18366316	3	18366316	328
S3_18366318	3	18366318	328
S3_18366392	3	18366392	328
S3_18396388	3	18396388	329
S3_18411505	3	18411505	329
S3_18526166	3	18526166	330

Markers	Chromosome	Position	Block #
S3_18526167	3	18526167	330
S3_18526170	3	18526170	330
S3_18526194	3	18526194	330
S3_18528496	3	18528496	331
S3_18554916	3	18554916	331
S3_18667313	3	18667313	332
S3_18708739	3	18708739	332
S3_19175371	3	19175371	333
S3_19175380	3	19175380	333
S3_19348148	3	19348148	334
S3_19352715	3	19352715	334
S3_19410141	3	19410141	335
S3_19432891	3	19432891	335
S3_19433031	3	19433031	335
S3_19433045	3	19433045	335
S3_19682057	3	19682057	336
S3_19682453	3	19682453	336
S3_19682480	3	19682480	336
S3_19833243	3	19833243	337
S3_19833261	3	19833261	337
S3_19931694	3	19931694	338
S3_19931731	3	19931731	338
S3_19977076	3	19977076	339
S3_19977077	3	19977077	339
S3_19980709	3	19980709	339
S3_20289935	3	20289935	340
S3_20289937	3	20289937	340
S3_20347799	3	20347799	341
S3_20347833	3	20347833	341
S3_20481508	3	20481508	342
S3_20509769	3	20509769	342
S3_20509772	3	20509772	342
S3_20556812	3	20556812	343
S3_20556819	3	20556819	343
S3_20751801	3	20751801	344
S3_20771687	3	20771687	344
S3_20789425	3	20789425	345
S3_20789445	3	20789445	345
S3_20852029	3	20852029	346
S3_20852127	3	20852127	346
S3_20865752	3	20865752	347
S3_20865753	3	20865753	347
S3_20875857	3	20875857	348
S3_20875878	3	20875878	348
S3_20875947	3	20875947	348
S3_20876046	3	20876046	348

Markers	Chromosome	Position	Block #
S3_21005190	3	21005190	349
S3_21005211	3	21005211	349
S3_21041029	3	21041029	350
S3_21046923	3	21046923	350
S3_21363717	3	21363717	351
S3_21363890	3	21363890	351
S3_21468859	3	21468859	352
S3_21468887	3	21468887	352
S3_21548077	3	21548077	353
S3_21548092	3	21548092	353
S3_21735942	3	21735942	354
S3_21735943	3	21735943	354
S3_22161054	3	22161054	355
S3_22161060	3	22161060	355
S3_22161078	3	22161078	355
S3_22229398	3	22229398	356
S3_22229455	3	22229455	356
S3_22531466	3	22531466	357
S3_22531565	3	22531565	357
S3_22630590	3	22630590	358
S3_22633279	3	22633279	358
S3_22647873	3	22647873	359
S3_22647995	3	22647995	359
S3_22648051	3	22648051	359
S3_22657440	3	22657440	359
S3_22659334	3	22659334	359
S3_22670092	3	22670092	360
S3_22670095	3	22670095	360
S3_22670125	3	22670125	360
S3_22700253	3	22700253	360
S3_22808950	3	22808950	361
S3_22808991	3	22808991	361
S3_22885886	3	22885886	362
S3_22885890	3	22885890	362
S3_23048694	3	23048694	363
S3_23054984	3	23054984	363
S3_23314138	3	23314138	364
S3_23314139	3	23314139	364
S3_23314140	3	23314140	364
S3_23317576	3	23317576	365
S3_23317577	3	23317577	365
S3_23317585	3	23317585	366
S3_23325427	3	23325427	366
S3_23387004	3	23387004	367
S3_23387021	3	23387021	367
S3_23387027	3	23387027	367

Markers	Chromosome	Position	Block #
S3_23409864	3	23409864	368
S3_23409865	3	23409865	368
S3_23409866	3	23409866	368
S3_23438864	3	23438864	369
S3_23439037	3	23439037	369
S3_23485104	3	23485104	370
S3_23485355	3	23485355	370
S3_23756796	3	23756796	371
S3_23772511	3	23772511	371
S3_23772615	3	23772615	371
S3_23772832	3	23772832	371
S3_23772837	3	23772837	371
S3_23773363	3	23773363	372
S3_23773484	3	23773484	372
S3_23843551	3	23843551	373
S3_23843552	3	23843552	373
S3_24069637	3	24069637	374
S3_24069638	3	24069638	374
S3_24074882	3	24074882	375
S3_24074893	3	24074893	375
S3_24197689	3	24197689	376
S3_24197690	3	24197690	376
S3_24197691	3	24197691	376
S3_24228511	3	24228511	377
S3_24228583	3	24228583	377
S3_24441403	3	24441403	378
S3_24441438	3	24441438	378
S3_24797760	3	24797760	379
S3_24799999	3	24799999	379
S3_24800655	3	24800655	379
S3_24876003	3	24876003	380
S3_24899414	3	24899414	380
S3_24899482	3	24899482	380
S3_24920422	3	24920422	380
S3_24993753	3	24993753	381
S3_24993764	3	24993764	381
S3_25058370	3	25058370	382
S3_25058403	3	25058403	382
S3_25180508	3	25180508	383
S3_25182611	3	25182611	383
S3_25182650	3	25182650	384
S3_25182651	3	25182651	384
S3_25182652	3	25182652	384
S3_25225358	3	25225358	385
S3_25225359	3	25225359	385
S3_25225360	3	25225360	385

Markers	Chromosome	Position	Block #
S3_25281079	3	25281079	386
S3_25329043	3	25329043	386
S3_25329232	3	25329232	386
S3_25337337	3	25337337	386
S3_25384667	3	25384667	386
S3_25415121	3	25415121	386
S3_25454038	3	25454038	387
S3_25454047	3	25454047	387
S3_25480716	3	25480716	388
S3_25481028	3	25481028	388
S3_25562363	3	25562363	389
S3_25566903	3	25566903	389
S3_25618566	3	25618566	390
S3_25618664	3	25618664	390
S3_25761803	3	25761803	391
S3_25764733	3	25764733	391
S3_25875968	3	25875968	392
S3_25876015	3	25876015	392
S3_25876105	3	25876105	393
S3_25882191	3	25882191	393
S3_25938906	3	25938906	394
S3_25938907	3	25938907	394
S3_25938908	3	25938908	394
S3_25939298	3	25939298	395
S3_25941815	3	25941815	395
S3_26041244	3	26041244	396
S3_26049178	3	26049178	396
S3_26049206	3	26049206	396
S3_26117111	3	26117111	397
S3_26117125	3	26117125	397
S3_26175228	3	26175228	398
S3_26188028	3	26188028	398
S3_26189955	3	26189955	398
S3_26190126	3	26190126	398
S3_26194682	3	26194682	399
S3_26194800	3	26194800	399
S3_26194899	3	26194899	399
S3_26199867	3	26199867	399
S3_26204845	3	26204845	399
S3_26204871	3	26204871	399
S3_26247448	3	26247448	400
S3_26247472	3	26247472	400
S3_26294742	3	26294742	401
S3_26294800	3	26294800	401
S3_26294850	3	26294850	401
S3_26295755	3	26295755	402

Markers	Chromosome	Position	Block #
S3_26295788	3	26295788	402
S3_26295810	3	26295810	402
S3_26375911	3	26375911	403
S3_26375912	3	26375912	403
S3_26379387	3	26379387	404
S3_26382658	3	26382658	404
S3_26382663	3	26382663	404
S3_26411486	3	26411486	405
S3_26411487	3	26411487	405
S3_26411488	3	26411488	405
S3_26425597	3	26425597	406
S3_26425614	3	26425614	406
S3_26425634	3	26425634	406
S3_26484221	3	26484221	407
S3_26537286	3	26537286	407
S3_26537288	3	26537288	407
S3_26564924	3	26564924	407
S3_26564938	3	26564938	407
S3_26565265	3	26565265	407
S3_26565315	3	26565315	407
S4_197447	4	197447	408
S4_197448	4	197448	408
S4_229167	4	229167	409
S4_239354	4	239354	409
S4_274146	4	274146	409
S4_303003	4	303003	410
S4_328431	4	328431	410
S4_409828	4	409828	411
S4_409833	4	409833	411
S4_413001	4	413001	411
S4_1245870	4	1245870	412
S4_1249850	4	1249850	412
S4_1249874	4	1249874	412
S4_1304938	4	1304938	413
S4_1304948	4	1304948	413
S4_1306145	4	1306145	413
S4_1366284	4	1366284	414
S4_1366314	4	1366314	414
S4_1366319	4	1366319	414
S4_1366359	4	1366359	414
S4_1849326	4	1849326	415
S4_1890187	4	1890187	415
S4_1890201	4	1890201	415
S4_1890203	4	1890203	415
S4_1890205	4	1890205	415
S4_1890280	4	1890280	415

Markers	Chromosome	Position	Block #
S4_1890311	4	1890311	415
S4_1891841	4	1891841	415
S4_1904091	4	1904091	415
S4_1968899	4	1968899	416
S4_1969028	4	1969028	416
S4_2179040	4	2179040	417
S4_2179165	4	2179165	417
S4_2190875	4	2190875	417
S4_2202204	4	2202204	418
S4_2217965	4	2217965	418
S4_2217975	4	2217975	418
S4_2266748	4	2266748	419
S4_2266810	4	2266810	419
S4_2371313	4	2371313	420
S4_2371388	4	2371388	420
S4_2371418	4	2371418	420
S4_2371458	4	2371458	420
S4_2389707	4	2389707	420
S4_2459203	4	2459203	421
S4_2462144	4	2462144	421
S4_2462146	4	2462146	421
S4_2470123	4	2470123	422
S4_2470127	4	2470127	422
S4_2487658	4	2487658	423
S4_2487665	4	2487665	423
S4_2584947	4	2584947	424
S4_2584950	4	2584950	424
S4_2584951	4	2584951	424
S4_2584952	4	2584952	424
S4_2642792	4	2642792	425
S4_2642795	4	2642795	425
S4_2654489	4	2654489	425
S4_2674636	4	2674636	425
S4_2674903	4	2674903	425
S4_2674978	4	2674978	425
S4_2677147	4	2677147	425
S4_2677353	4	2677353	426
S4_2685273	4	2685273	426
S4_2685276	4	2685276	426
S4_2739828	4	2739828	427
S4_2739845	4	2739845	427
S4_2747820	4	2747820	427
S4_2755375	4	2755375	428
S4_2755490	4	2755490	428
S4_2775010	4	2775010	428
S4_2876742	4	2876742	429

Markers	Chromosome	Position	Block #
S4_2876769	4	2876769	429
S4_2947491	4	2947491	430
S4_2947554	4	2947554	430
S4_2991022	4	2991022	431
S4_2993891	4	2993891	431
S4_2993903	4	2993903	432
S4_2993999	4	2993999	432
S4_3109935	4	3109935	433
S4_3109945	4	3109945	433
S4_3109946	4	3109946	433
S4_3109950	4	3109950	433
S4_3112068	4	3112068	434
S4_3112086	4	3112086	434
S4_3112096	4	3112096	434
S4_3112206	4	3112206	434
S4_3112230	4	3112230	434
S4_3117460	4	3117460	434
S4_3120686	4	3120686	435
S4_3120687	4	3120687	435
S4_3137672	4	3137672	436
S4_3154882	4	3154882	436
S4_3154974	4	3154974	436
S4_3162514	4	3162514	436
S4_3217031	4	3217031	437
S4_3247166	4	3247166	437
S4_3291382	4	3291382	438
S4_3291413	4	3291413	438
S4_3313404	4	3313404	438
S4_3410544	4	3410544	439
S4_3410612	4	3410612	439
S4_3442460	4	3442460	440
S4_3442596	4	3442596	440
S4_3512527	4	3512527	441
S4_3512532	4	3512532	441
S4_3540910	4	3540910	442
S4_3569523	4	3569523	442
S4_3569524	4	3569524	443
S4_3570964	4	3570964	443
S4_3609668	4	3609668	444
S4_3609685	4	3609685	444
S4_3766272	4	3766272	445
S4_3766273	4	3766273	445
S4_3766274	4	3766274	445
S4_3920450	4	3920450	446
S4_3920460	4	3920460	446
S4_3920498	4	3920498	446

Markers	Chromosome	Position	Block #
S4_3925413	4	3925413	446
S4_4883769	4	4883769	447
S4_4932562	4	4932562	447
S4_5238915	4	5238915	448
S4_5238939	4	5238939	448
S4_5670570	4	5670570	449
S4_5671120	4	5671120	449
S4_5757215	4	5757215	449
S4_6328212	4	6328212	450
S4_6328233	4	6328233	450
S4_7728100	4	7728100	451
S4_7729036	4	7729036	451
S4_7777402	4	7777402	452
S4_7820582	4	7820582	452
S4_7820594	4	7820594	452
S4_7821342	4	7821342	453
S4_7821356	4	7821356	453
S4_8027535	4	8027535	454
S4_8027676	4	8027676	454
S4_8103264	4	8103264	455
S4_8183349	4	8183349	455
S4_8183390	4	8183390	455
S4_8207976	4	8207976	455
S4_8894636	4	8894636	456
S4_8958274	4	8958274	456
S4_8961505	4	8961505	456
S4_9378114	4	9378114	457
S4_9441138	4	9441138	457
S4_9446976	4	9446976	458
S4_9447070	4	9447070	458
S4_9447090	4	9447090	459
S4_9447131	4	9447131	459
S4_9447533	4	9447533	460
S4_9448365	4	9448365	460
S4_10109682	4	10109682	461
S4_10109705	4	10109705	461
S4_10109718	4	10109718	461
S4_10220204	4	10220204	461
S4_10355843	4	10355843	462
S4_10413307	4	10413307	462
S4_10418823	4	10418823	462
S4_10438223	4	10438223	462
S4_10595594	4	10595594	463
S4_10595753	4	10595753	463
S4_10730981	4	10730981	463
S4_11001241	4	11001241	464

Markers	Chromosome	Position	Block #
S4_11003110	4	11003110	464
S4_11003137	4	11003137	464
S4_11852354	4	11852354	465
S4_11852356	4	11852356	465
S4_12051026	4	12051026	466
S4_12064649	4	12064649	466
S4_12132824	4	12132824	467
S4_12132992	4	12132992	467
S4_12195979	4	12195979	467
S4_12197454	4	12197454	467
S4_12395788	4	12395788	468
S4_12395789	4	12395789	468
S4_12395790	4	12395790	468
S4_12406687	4	12406687	469
S4_12406775	4	12406775	469
S4_12850189	4	12850189	470
S4_12859048	4	12859048	470
S4_12938785	4	12938785	470
S4_13224671	4	13224671	471
S4_13224850	4	13224850	471
S4_14412068	4	14412068	472
S4_14412069	4	14412069	472
S4_14412077	4	14412077	472
S4_14504373	4	14504373	473
S4_14506375	4	14506375	473
S4_15070024	4	15070024	474
S4_15070026	4	15070026	474
S4_15211104	4	15211104	475
S4_15211116	4	15211116	475
S4_15428654	4	15428654	476
S4_15428672	4	15428672	476
S4_16177707	4	16177707	477
S4_16327097	4	16327097	477
S4_16709900	4	16709900	478
S4_16709901	4	16709901	478
S4_18142322	4	18142322	479
S4_18188800	4	18188800	479
S4_18189082	4	18189082	479
S4_18215664	4	18215664	479
S4_18666667	4	18666667	480
S4_18711254	4	18711254	480
S4_18951366	4	18951366	481
S4_18962106	4	18962106	481
S4_19181030	4	19181030	482
S4_19181045	4	19181045	482
S4_19186319	4	19186319	483

Markers	Chromosome	Position	Block #
S4_19186353	4	19186353	483
S4_19281503	4	19281503	484
S4_19281547	4	19281547	484
S4_19281669	4	19281669	484
S4_19281671	4	19281671	484
S4_19281673	4	19281673	484
S4_19281674	4	19281674	484
S4_20348457	4	20348457	485
S4_20348478	4	20348478	485
S4_21772440	4	21772440	486
S4_21772446	4	21772446	486
S4_21793447	4	21793447	487
S4_21795647	4	21795647	487
S4_21962757	4	21962757	488
S4_21962758	4	21962758	488
S4_21962759	4	21962759	488
S4_22044681	4	22044681	489
S4_22044728	4	22044728	489
S4_22164780	4	22164780	490
S4_22233620	4	22233620	490
S4_22651310	4	22651310	491
S4_22665918	4	22665918	491
S4_22807328	4	22807328	492
S4_22807348	4	22807348	492
S4_23258067	4	23258067	493
S4_23258166	4	23258166	493
S4_23350453	4	23350453	494
S4_23354401	4	23354401	494
S4_23639365	4	23639365	495
S4_23639401	4	23639401	495
S4_23667854	4	23667854	496
S4_23667866	4	23667866	496
S4_23667972	4	23667972	497
S4_23667973	4	23667973	497
S4_23667975	4	23667975	497
S4_23972458	4	23972458	498
S4_23972579	4	23972579	498
S4_23990650	4	23990650	499
S4_23990727	4	23990727	499
S4_24393074	4	24393074	500
S4_24407032	4	24407032	500
S4_24733298	4	24733298	501
S4_24787325	4	24787325	501
S4_24787327	4	24787327	501
S4_24802699	4	24802699	501
S4_24837365	4	24837365	502

Markers	Chromosome	Position	Block #
S4_24837403	4	24837403	502
S4_24837404	4	24837404	502
S4_24908086	4	24908086	503
S4_24908092	4	24908092	503
S4_25322990	4	25322990	504
S4_25387545	4	25387545	504
S4_25400331	4	25400331	505
S4_25400332	4	25400332	505
S4_25572569	4	25572569	506
S4_25572695	4	25572695	506
S4_25762458	4	25762458	507
S4_25762511	4	25762511	507
S4_25773640	4	25773640	508
S4_25823403	4	25823403	508
S4_25852541	4	25852541	509
S4_25852620	4	25852620	509
S4_25879325	4	25879325	510
S4_25879412	4	25879412	510
S4_26194584	4	26194584	511
S4_26210438	4	26210438	511
S4_26225700	4	26225700	512
S4_26225732	4	26225732	512
S4_26440928	4	26440928	513
S4_26440929	4	26440929	513
S4_26440953	4	26440953	514
S4_26440954	4	26440954	514
S4_26440971	4	26440971	514
S4_26480634	4	26480634	515
S4_26506595	4	26506595	515
S4_26506621	4	26506621	515
S4_26594218	4	26594218	516
S4_26594269	4	26594269	516
S4_26693052	4	26693052	517
S4_26705555	4	26705555	517
S4_26737767	4	26737767	518
S4_26737768	4	26737768	518
S4_26882612	4	26882612	519
S4_26882617	4	26882617	519
S4_26882619	4	26882619	519
S4_26975999	4	26975999	520
S4_26976036	4	26976036	520
S4_27006001	4	27006001	521
S4_27006205	4	27006205	521
S4_27006214	4	27006214	521
S4_27370604	4	27370604	522
S4_27371889	4	27371889	522

Markers	Chromosome	Position	Block #
S4_27373852	4	27373852	522
S4_27393453	4	27393453	522
S4_27393483	4	27393483	522
S4_27393486	4	27393486	522
S4_27393696	4	27393696	522
S4_27393732	4	27393732	522
S4_27411015	4	27411015	522
S4_27443946	4	27443946	523
S4_27443950	4	27443950	523
S4_27490726	4	27490726	524
S4_27490735	4	27490735	524
S4_27510607	4	27510607	525
S4_27511513	4	27511513	525
S4_27511601	4	27511601	526
S4_27532372	4	27532372	526
S4_27725896	4	27725896	527
S4_27744942	4	27744942	527
S4_27745019	4	27745019	527
S4_27752996	4	27752996	527
S4_27753116	4	27753116	527
S4_27753190	4	27753190	527
S4_27902579	4	27902579	528
S4_27903126	4	27903126	528
S4_27936272	4	27936272	529
S4_27936276	4	27936276	529
S4_27942244	4	27942244	530
S4_27942375	4	27942375	530
S4_27975991	4	27975991	531
S4_27976105	4	27976105	531
S4_27983873	4	27983873	531
S4_27983967	4	27983967	531
S4_28071694	4	28071694	532
S4_28071695	4	28071695	532
S4_28071842	4	28071842	532
S4_28113058	4	28113058	533
S4_28113097	4	28113097	533
S4_28468113	4	28468113	534
S4_28472508	4	28472508	534
S4_28549025	4	28549025	535
S4_28549093	4	28549093	535
S4_28549095	4	28549095	535
S4_28587506	4	28587506	536
S4_28587519	4	28587519	536
S4_28769353	4	28769353	537
S4_28772328	4	28772328	537
S4_29166209	4	29166209	538

Markers	Chromosome	Position	Block #
S4_29169317	4	29169317	538
S4_29169354	4	29169354	538
S4_29240303	4	29240303	539
S4_29250227	4	29250227	539
S4_29250230	4	29250230	539
S4_29250258	4	29250258	540
S4_29254417	4	29254417	540
S4_29343261	4	29343261	541
S4_29343710	4	29343710	541
S4_29396505	4	29396505	542
S4_29396507	4	29396507	542
S4_29396509	4	29396509	542
S4_29397705	4	29397705	542
S4_29397890	4	29397890	542
S4_29402115	4	29402115	543
S4_29402195	4	29402195	543
S4_29402488	4	29402488	543
S4_29501518	4	29501518	544
S4_29510545	4	29510545	544
S4_29564946	4	29564946	545
S4_29566910	4	29566910	545
S4_29571735	4	29571735	546
S4_29571857	4	29571857	546
S4_29825020	4	29825020	547
S4_29825031	4	29825031	547
S4_29847180	4	29847180	548
S4_29856813	4	29856813	548
S4_29927912	4	29927912	549
S4_29930626	4	29930626	549
S4_29931057	4	29931057	549
S4_29931098	4	29931098	549
S4_29936595	4	29936595	549
S4_29936631	4	29936631	549
S4_29950541	4	29950541	550
S4_29950624	4	29950624	550
S4_29993169	4	29993169	550
S4_30004572	4	30004572	550
S4_30048541	4	30048541	550
S4_30074906	4	30074906	550
S5_118870	5	118870	551
S5_125424	5	125424	551
S5_231534	5	231534	552
S5_231590	5	231590	552
S5_250141	5	250141	553
S5_250144	5	250144	553
S5_250145	5	250145	553

Markers	Chromosome	Position	Block #
S5_251810	5	251810	554
S5_252974	5	252974	554
S5_605992	5	605992	555
S5_605993	5	605993	555
S5_605996	5	605996	555
S5_641240	5	641240	556
S5_641266	5	641266	556
S5_690783	5	690783	557
S5_690799	5	690799	557
S5_844615	5	844615	558
S5_844673	5	844673	558
S5_844686	5	844686	558
S5_844780	5	844780	558
S5_1019194	5	1019194	559
S5_1019195	5	1019195	559
S5_1019196	5	1019196	559
S5_1150405	5	1150405	560
S5_1197615	5	1197615	560
S5_1197966	5	1197966	560
S5_1202703	5	1202703	560
S5_1223471	5	1223471	561
S5_1223474	5	1223474	561
S5_1255888	5	1255888	562
S5_1255897	5	1255897	562
S5_1259246	5	1259246	562
S5_1260435	5	1260435	562
S5_1297362	5	1297362	562
S5_1315066	5	1315066	562
S5_1331479	5	1331479	562
S5_1435104	5	1435104	563
S5_1435189	5	1435189	563
S5_1435190	5	1435190	563
S5_1435198	5	1435198	563
S5_1616110	5	1616110	564
S5_1623931	5	1623931	564
S5_1702592	5	1702592	565
S5_1702607	5	1702607	565
S5_1703889	5	1703889	566
S5_1705879	5	1705879	566
S5_1795426	5	1795426	567
S5_1795431	5	1795431	567
S5_1908137	5	1908137	568
S5_1908167	5	1908167	568
S5_2000399	5	2000399	569
S5_2007151	5	2007151	569
S5_2007183	5	2007183	569

Markers	Chromosome	Position	Block #
S5_2040887	5	2040887	570
S5_2040955	5	2040955	570
S5_2042697	5	2042697	571
S5_2043654	5	2043654	571
S5_2043855	5	2043855	572
S5_2043925	5	2043925	572
S5_2180136	5	2180136	573
S5_2180151	5	2180151	573
S5_2221831	5	2221831	573
S5_2396673	5	2396673	574
S5_2398417	5	2398417	574
S5_2797288	5	2797288	575
S5_2797300	5	2797300	575
S5_2798703	5	2798703	576
S5_2798738	5	2798738	576
S5_2798765	5	2798765	576
S5_2798779	5	2798779	576
S5_2825645	5	2825645	576
S5_2825669	5	2825669	576
S5_2825686	5	2825686	576
S5_2858726	5	2858726	576
S5_2859581	5	2859581	576
S5_2910064	5	2910064	577
S5_2910108	5	2910108	577
S5_2910114	5	2910114	577
S5_2952159	5	2952159	578
S5_2952186	5	2952186	578
S5_3186464	5	3186464	579
S5_3186489	5	3186489	579
S5_3475637	5	3475637	580
S5_3475640	5	3475640	580
S5_3750247	5	3750247	581
S5_3750398	5	3750398	581
S5_4101856	5	4101856	582
S5_4101905	5	4101905	582
S5_4186736	5	4186736	583
S5_4194580	5	4194580	583
S5_4209280	5	4209280	584
S5_4209532	5	4209532	584
S5_4372042	5	4372042	585
S5_4399456	5	4399456	585
S5_4399470	5	4399470	585
S5_4758533	5	4758533	586
S5_4758534	5	4758534	586
S5_4758536	5	4758536	586
S5_4794996	5	4794996	587

Markers	Chromosome	Position	Block #
S5_4795028	5	4795028	587
S5_4999060	5	4999060	588
S5_4999081	5	4999081	588
S5_4999132	5	4999132	588
S5_5084333	5	5084333	589
S5_5194293	5	5194293	589
S5_5227563	5	5227563	590
S5_5366085	5	5366085	590
S5_5369824	5	5369824	590
S5_6519295	5	6519295	591
S5_6641188	5	6641188	591
S5_6659643	5	6659643	592
S5_6659666	5	6659666	592
S5_6844686	5	6844686	593
S5_6849272	5	6849272	593
S5_7021827	5	7021827	594
S5_7021836	5	7021836	594
S5_8638408	5	8638408	595
S5_8638419	5	8638419	595
S5_8638430	5	8638430	595
S5_8638448	5	8638448	596
S5_8638532	5	8638532	596
S5_8638558	5	8638558	596
S5_8638593	5	8638593	596
S5_8638638	5	8638638	596
S5_8638732	5	8638732	596
S5_9038609	5	9038609	597
S5_9193953	5	9193953	597
S5_9258879	5	9258879	598
S5_9311201	5	9311201	598
S5_9371947	5	9371947	599
S5_9371960	5	9371960	599
S5_9548853	5	9548853	600
S5_9548888	5	9548888	600
S5_9650925	5	9650925	601
S5_9660257	5	9660257	601
S5_10767173	5	10767173	602
S5_10767275	5	10767275	602
S5_11028835	5	11028835	603
S5_11028836	5	11028836	603
S5_11028839	5	11028839	603
S5_12240278	5	12240278	604
S5_12240486	5	12240486	604
S5_12242856	5	12242856	604
S5_12379304	5	12379304	604
S5_12599778	5	12599778	605

Markers	Chromosome	Position	Block #
S5_12599779	5	12599779	605
S5_12599780	5	12599780	605
S5_12875797	5	12875797	606
S5_12930620	5	12930620	606
S5_13249049	5	13249049	607
S5_13279981	5	13279981	607
S5_13281675	5	13281675	607
S5_14089818	5	14089818	608
S5_14094433	5	14094433	608
S5_14147256	5	14147256	609
S5_14221152	5	14221152	609
S5_14284899	5	14284899	609
S5_14284921	5	14284921	609
S5_14299205	5	14299205	610
S5_14299259	5	14299259	610
S5_14452328	5	14452328	610
S5_14526119	5	14526119	611
S5_14526121	5	14526121	611
S5_14743213	5	14743213	612
S5_14743228	5	14743228	612
S5_15566357	5	15566357	613
S5_15566366	5	15566366	613
S5_15566381	5	15566381	613
S5_15618868	5	15618868	613
S5_15752467	5	15752467	614
S5_15754825	5	15754825	614
S5_15754855	5	15754855	615
S5_15796591	5	15796591	615
S5_15796642	5	15796642	615
S5_15796670	5	15796670	616
S5_15797132	5	15797132	616
S5_16277003	5	16277003	617
S5_16277009	5	16277009	617
S5_16652194	5	16652194	618
S5_16656895	5	16656895	618
S5_16679253	5	16679253	619
S5_16733825	5	16733825	619
S5_16955902	5	16955902	620
S5_16967685	5	16967685	620
S5_17000146	5	17000146	620
S5_17240714	5	17240714	621
S5_17242077	5	17242077	621
S5_17842737	5	17842737	622
S5_17842764	5	17842764	622
S5_17842792	5	17842792	622
S5_17977074	5	17977074	623

Markers	Chromosome	Position	Block #
S5_18086955	5	18086955	623
S5_19698064	5	19698064	624
S5_19698176	5	19698176	624
S5_19782125	5	19782125	625
S5_19789526	5	19789526	625
S5_19789643	5	19789643	625
S5_19789779	5	19789779	625
S5_19789793	5	19789793	625
S5_19835842	5	19835842	625
S5_19851788	5	19851788	625
S5_19855371	5	19855371	625
S5_19855402	5	19855402	625
S5_19855403	5	19855403	625
S5_19869862	5	19869862	625
S5_20482918	5	20482918	626
S5_20490456	5	20490456	626
S5_20850943	5	20850943	627
S5_20863953	5	20863953	627
S5_20948439	5	20948439	627
S5_20948481	5	20948481	627
S5_21290347	5	21290347	628
S5_21290348	5	21290348	628
S5_21465665	5	21465665	629
S5_21472954	5	21472954	629
S5_22964278	5	22964278	630
S5_22964279	5	22964279	630
S5_23032658	5	23032658	631
S5_23032669	5	23032669	631
S5_23032682	5	23032682	631
S5_23032685	5	23032685	631
S5_23116617	5	23116617	632
S5_23212658	5	23212658	632
S5_23553223	5	23553223	633
S5_23553364	5	23553364	633
S5_23565973	5	23565973	634
S5_23566176	5	23566176	634
S5_23737584	5	23737584	635
S5_23737585	5	23737585	635
S5_23737638	5	23737638	635
S5_23876198	5	23876198	636
S5_23974004	5	23974004	636
S5_24030311	5	24030311	637
S5_24030323	5	24030323	637
S5_24149689	5	24149689	638
S5_24151933	5	24151933	638
S5_24167144	5	24167144	639

Markers	Chromosome	Position	Block #
S5_24167150	5	24167150	639
S5_24466903	5	24466903	640
S5_24466941	5	24466941	640
S5_24977067	5	24977067	641
S5_24977176	5	24977176	641
S5_25706179	5	25706179	642
S5_25706200	5	25706200	642
S5_25765994	5	25765994	643
S5_25766006	5	25766006	643
S5_25766030	5	25766030	643
S5_25766037	5	25766037	643
S5_25834139	5	25834139	644
S5_25850173	5	25850173	644
S5_25851010	5	25851010	644
S5_26087821	5	26087821	645
S5_26087848	5	26087848	645
S5_26194314	5	26194314	646
S5_26194316	5	26194316	646
S5_26208335	5	26208335	647
S5_26244626	5	26244626	647
S5_26244627	5	26244627	647
S5_26246216	5	26246216	648
S5_26251525	5	26251525	648
S5_26251539	5	26251539	648
S5_26264580	5	26264580	649
S5_26264595	5	26264595	649
S5_26264697	5	26264697	650
S5_26264698	5	26264698	650
S5_26271225	5	26271225	651
S5_26271226	5	26271226	651
S5_26271333	5	26271333	651
S5_26271367	5	26271367	652
S5_26271368	5	26271368	652
S5_26271369	5	26271369	652
S5_26271380	5	26271380	652
S5_26271398	5	26271398	652
S5_26278133	5	26278133	652
S5_26278135	5	26278135	652
S5_26289896	5	26289896	652
S5_26291163	5	26291163	652
S5_26354302	5	26354302	653
S5_26358941	5	26358941	653
S5_26358959	5	26358959	653
S5_26361514	5	26361514	654
S5_26361516	5	26361516	654
S5_26591042	5	26591042	655

Markers	Chromosome	Position	Block #
S5_26591064	5	26591064	655
S5_26603125	5	26603125	656
S5_26603128	5	26603128	656
S5_26647046	5	26647046	657
S5_26647049	5	26647049	657
S5_26711939	5	26711939	658
S5_26742679	5	26742679	658
S5_26746604	5	26746604	659
S5_26746791	5	26746791	659
S5_26777775	5	26777775	660
S5_26781670	5	26781670	660
S5_26890088	5	26890088	661
S5_26893832	5	26893832	661
S5_27110716	5	27110716	662
S5_27110718	5	27110718	662
S5_27192719	5	27192719	663
S5_27192780	5	27192780	663
S5_27200659	5	27200659	663
S5_27413999	5	27413999	664
S5_27414002	5	27414002	664
S5_27414003	5	27414003	664
S5_27414004	5	27414004	664
S5_27414005	5	27414005	664
S5_27686424	5	27686424	665
S5_27686513	5	27686513	665
S5_27759547	5	27759547	666
S5_27759548	5	27759548	666
S5_27844864	5	27844864	667
S5_27844881	5	27844881	667
S5_27844884	5	27844884	667
S5_27857075	5	27857075	667
S5_27857114	5	27857114	667
S5_28022281	5	28022281	668
S5_28022309	5	28022309	668
S5_28053391	5	28053391	669
S5_28056620	5	28056620	669
S5_28075264	5	28075264	670
S5_28075504	5	28075504	670
S5_28181196	5	28181196	671
S5_28181199	5	28181199	671
S5_28181200	5	28181200	671
S5_28196759	5	28196759	672
S5_28212447	5	28212447	672
S5_28222523	5	28222523	673
S5_28222555	5	28222555	673
S5_28256255	5	28256255	674

Markers	Chromosome	Position	Block #
S5_28258653	5	28258653	674
S5_28297486	5	28297486	674
S5_28297491	5	28297491	674
S5_28306399	5	28306399	674
S5_28306476	5	28306476	674
S5_28320997	5	28320997	674
S5_28325493	5	28325493	674
S5_28325528	5	28325528	674
S5_28325579	5	28325579	674
S5_28325602	5	28325602	674
S5_28350120	5	28350120	674
S5_28350172	5	28350172	674
S5_28350210	5	28350210	674
S5_28397992	5	28397992	674
S6_95582	6	95582	675
S6_95618	6	95618	675
S6_147144	6	147144	676
S6_157987	6	157987	676
S6_162168	6	162168	676
S6_187074	6	187074	677
S6_187075	6	187075	677
S6_254951	6	254951	678
S6_267162	6	267162	678
S6_484476	6	484476	679
S6_484477	6	484477	679
S6_507668	6	507668	680
S6_507761	6	507761	680
S6_516413	6	516413	680
S6_516416	6	516416	680
S6_516441	6	516441	680
S6_524359	6	524359	680
S6_527486	6	527486	680
S6_543412	6	543412	680
S6_658122	6	658122	681
S6_663212	6	663212	681
S6_663237	6	663237	681
S6_875904	6	875904	682
S6_876012	6	876012	682
S6_906984	6	906984	683
S6_908493	6	908493	683
S6_908595	6	908595	683
S6_916580	6	916580	684
S6_916747	6	916747	684
S6_973680	6	973680	685
S6_973782	6	973782	685
S6_973846	6	973846	686

Markers	Chromosome	Position	Block #
S6_973972	6	973972	686
S6_975636	6	975636	687
S6_975637	6	975637	687
S6_975638	6	975638	687
S6_975640	6	975640	687
S6_975642	6	975642	687
S6_983587	6	983587	688
S6_983600	6	983600	688
S6_983624	6	983624	688
S6_1083834	6	1083834	689
S6_1083931	6	1083931	689
S6_1105384	6	1105384	690
S6_1105389	6	1105389	690
S6_1189474	6	1189474	691
S6_1198427	6	1198427	691
S6_1274460	6	1274460	692
S6_1302815	6	1302815	692
S6_1363953	6	1363953	693
S6_1369646	6	1369646	693
S6_1449724	6	1449724	694
S6_1451557	6	1451557	694
S6_1453469	6	1453469	694
S6_1964074	6	1964074	695
S6_1964077	6	1964077	695
S6_2255865	6	2255865	696
S6_2256075	6	2256075	696
S6_2306140	6	2306140	697
S6_2306181	6	2306181	697
S6_2341346	6	2341346	698
S6_2375504	6	2375504	698
S6_2375511	6	2375511	698
S6_2559235	6	2559235	699
S6_2586420	6	2586420	699
S6_2890008	6	2890008	700
S6_2890225	6	2890225	700
S6_2966563	6	2966563	701
S6_2966587	6	2966587	701
S6_2973905	6	2973905	702
S6_2973923	6	2973923	702
S6_2999468	6	2999468	703
S6_2999484	6	2999484	703
S6_2999513	6	2999513	703
S6_3060289	6	3060289	704
S6_3061650	6	3061650	704
S6_3180492	6	3180492	705
S6_3180506	6	3180506	705

Markers	Chromosome	Position	Block #
S6_3218313	6	3218313	706
S6_3218627	6	3218627	706
S6_3404454	6	3404454	707
S6_3416558	6	3416558	707
S6_3416632	6	3416632	708
S6_3416636	6	3416636	708
S6_3416644	6	3416644	708
S6_3552112	6	3552112	709
S6_3557410	6	3557410	709
S6_3696172	6	3696172	710
S6_3698514	6	3698514	710
S6_3901793	6	3901793	711
S6_3901958	6	3901958	711
S6_4133365	6	4133365	712
S6_4133366	6	4133366	712
S6_4133391	6	4133391	712
S6_4133401	6	4133401	712
S6_4135294	6	4135294	712
S6_4135333	6	4135333	712
S6_4467966	6	4467966	713
S6_4468125	6	4468125	713
S6_4471947	6	4471947	713
S6_4471965	6	4471965	713
S6_4473150	6	4473150	713
S6_4473185	6	4473185	713
S6_4545831	6	4545831	714
S6_4545878	6	4545878	714
S6_4877574	6	4877574	715
S6_4877684	6	4877684	715
S6_5034987	6	5034987	716
S6_5035051	6	5035051	716
S6_5035062	6	5035062	716
S6_5118568	6	5118568	717
S6_5118708	6	5118708	717
S6_5233209	6	5233209	718
S6_5237489	6	5237489	718
S6_5246797	6	5246797	718
S6_5248700	6	5248700	718
S6_5265364	6	5265364	718
S6_5269877	6	5269877	718
S6_5278753	6	5278753	718
S6_5384680	6	5384680	719
S6_5384681	6	5384681	719
S6_5415363	6	5415363	720
S6_5417728	6	5417728	720
S6_5542552	6	5542552	721

Markers	Chromosome	Position	Block #
S6_5593685	6	5593685	721
S6_5593733	6	5593733	721
S6_5593866	6	5593866	721
S6_5627403	6	5627403	721
S6_5638258	6	5638258	722
S6_5638269	6	5638269	722
S6_5655454	6	5655454	723
S6_5657755	6	5657755	723
S6_6027523	6	6027523	724
S6_6038373	6	6038373	724
S6_6044492	6	6044492	725
S6_6063081	6	6063081	725
S6_6491771	6	6491771	726
S6_6491793	6	6491793	726
S6_6931736	6	6931736	727
S6_6931737	6	6931737	727
S6_7066125	6	7066125	728
S6_7067908	6	7067908	728
S6_7318607	6	7318607	729
S6_7318620	6	7318620	729
S6_7344330	6	7344330	730
S6_7344361	6	7344361	730
S6_7344364	6	7344364	730
S6_7498707	6	7498707	731
S6_7498716	6	7498716	731
S6_7642841	6	7642841	732
S6_7645851	6	7645851	732
S6_7645860	6	7645860	732
S6_7653426	6	7653426	733
S6_7653427	6	7653427	733
S6_7679936	6	7679936	734
S6_7679938	6	7679938	734
S6_7679986	6	7679986	734
S6_7753657	6	7753657	735
S6_7753683	6	7753683	735
S6_8023360	6	8023360	736
S6_8042369	6	8042369	736
S6_8213143	6	8213143	737
S6_8213170	6	8213170	737
S6_8337787	6	8337787	738
S6_8343811	6	8343811	738
S6_8394272	6	8394272	738
S6_9471109	6	9471109	739
S6_9536763	6	9536763	739
S6_9536769	6	9536769	739
S6_9608123	6	9608123	740

Markers	Chromosome	Position	Block #
S6_9612944	6	9612944	740
S6_9710831	6	9710831	741
S6_9710832	6	9710832	741
S6_9710833	6	9710833	741
S6_9710972	6	9710972	742
S6_9710987	6	9710987	742
S6_9718897	6	9718897	743
S6_9718940	6	9718940	743
S6_9793414	6	9793414	744
S6_9793416	6	9793416	744
S6_9793422	6	9793422	744
S6_9793423	6	9793423	744
S6_10884661	6	10884661	745
S6_10920645	6	10920645	745
S6_11201458	6	11201458	746
S6_11201908	6	11201908	746
S6_11202028	6	11202028	747
S6_11202301	6	11202301	747
S6_11237645	6	11237645	747
S6_11305573	6	11305573	747
S6_11305649	6	11305649	747
S6_11340847	6	11340847	747
S6_11341487	6	11341487	747
S6_11981108	6	11981108	748
S6_11995870	6	11995870	748
S6_11998088	6	11998088	748
S6_12039887	6	12039887	749
S6_12039895	6	12039895	749
S6_12043352	6	12043352	750
S6_12043354	6	12043354	750
S6_12043355	6	12043355	750
S6_12043356	6	12043356	750
S6_12048210	6	12048210	750
S6_12204185	6	12204185	751
S6_12351588	6	12351588	751
S6_12351612	6	12351612	751
S6_13031747	6	13031747	752
S6_13074491	6	13074491	752
S6_13465025	6	13465025	753
S6_13466204	6	13466204	753
S6_13646504	6	13646504	754
S6_13646518	6	13646518	754
S6_13815972	6	13815972	755
S6_13815975	6	13815975	755
S6_13878521	6	13878521	756
S6_13878527	6	13878527	756

Markers	Chromosome	Position	Block #
S6_13878535	6	13878535	756
S6_14223263	6	14223263	757
S6_14223599	6	14223599	757
S6_14223636	6	14223636	757
S6_14517453	6	14517453	758
S6_14527972	6	14527972	758
S6_14527974	6	14527974	758
S6_14528685	6	14528685	758
S6_14528686	6	14528686	758
S6_14528687	6	14528687	758
S6_14528688	6	14528688	758
S6_14528689	6	14528689	758
S6_14528690	6	14528690	758
S6_14528691	6	14528691	758
S6_14528692	6	14528692	758
S6_14528693	6	14528693	758
S6_14528696	6	14528696	758
S6_14528699	6	14528699	758
S6_14528700	6	14528700	758
S6_14528701	6	14528701	758
S6_14528702	6	14528702	758
S6_14528705	6	14528705	758
S6_14528706	6	14528706	758
S6_14528707	6	14528707	758
S6_14528708	6	14528708	758
S6_14528710	6	14528710	758
S6_14767583	6	14767583	759
S6_14767584	6	14767584	759
S6_14767585	6	14767585	759
S6_15016293	6	15016293	760
S6_15082105	6	15082105	760
S6_15340282	6	15340282	761
S6_15353467	6	15353467	761
S6_15555740	6	15555740	762
S6_15556698	6	15556698	762
S6_15556699	6	15556699	762
S6_15556778	6	15556778	762
S6_15866656	6	15866656	763
S6_15866708	6	15866708	763
S6_16155976	6	16155976	764
S6_16156024	6	16156024	764
S6_16233618	6	16233618	765
S6_16236696	6	16236696	765
S6_16560975	6	16560975	766
S6_16561051	6	16561051	766
S6_16821151	6	16821151	767

Markers	Chromosome	Position	Block #
S6_16846420	6	16846420	767
S6_18059911	6	18059911	768
S6_18060077	6	18060077	768
S6_18071295	6	18071295	769
S6_18071296	6	18071296	769
S6_18190554	6	18190554	770
S6_18194993	6	18194993	770
S6_18195620	6	18195620	770
S6_19616245	6	19616245	771
S6_19616318	6	19616318	771
S6_19623934	6	19623934	771
S6_19787827	6	19787827	772
S6_19894038	6	19894038	772
S6_19894145	6	19894145	772
S6_20433874	6	20433874	773
S6_20433875	6	20433875	773
S6_20433876	6	20433876	773
S6_20610839	6	20610839	774
S6_20639963	6	20639963	774
S6_20661563	6	20661563	775
S6_20661564	6	20661564	775
S6_20661565	6	20661565	775
S6_20661567	6	20661567	775
S6_20661582	6	20661582	775
S6_22382220	6	22382220	776
S6_22382236	6	22382236	776
S6_22382275	6	22382275	776
S6_22406681	6	22406681	777
S6_22486409	6	22486409	777
S6_23964843	6	23964843	778
S6_23965103	6	23965103	778
S6_24280497	6	24280497	779
S6_24280515	6	24280515	779
S6_24617530	6	24617530	780
S6_24617987	6	24617987	780
S6_24924606	6	24924606	781
S6_24930561	6	24930561	781
S6_25091365	6	25091365	782
S6_25091391	6	25091391	782
S6_25099323	6	25099323	783
S6_25099527	6	25099527	783
S6_25222292	6	25222292	784
S6_25222293	6	25222293	784
S6_25222302	6	25222302	784
S6_25222311	6	25222311	784
S6_25229908	6	25229908	785

Markers	Chromosome	Position	Block #
S6_25231860	6	25231860	785
S6_25246620	6	25246620	785
S6_25247658	6	25247658	785
S6_25247736	6	25247736	785
S6_25259805	6	25259805	785
S6_25260214	6	25260214	785
S6_25267372	6	25267372	785
S6_25273783	6	25273783	785
S6_25279002	6	25279002	785
S6_25360549	6	25360549	785
S6_25374549	6	25374549	785
S6_25644947	6	25644947	786
S6_25656435	6	25656435	786
S6_25683450	6	25683450	786
S6_25855716	6	25855716	787
S6_25856917	6	25856917	787
S6_26232374	6	26232374	788
S6_26232382	6	26232382	788
S6_26232487	6	26232487	788
S6_26266190	6	26266190	789
S6_26302680	6	26302680	789
S6_26378562	6	26378562	790
S6_26391515	6	26391515	790
S6_26454025	6	26454025	791
S6_26454057	6	26454057	791
S6_26505383	6	26505383	792
S6_26565310	6	26565310	792
S6_26614401	6	26614401	793
S6_26614408	6	26614408	793
S6_26614413	6	26614413	793
S6_26614419	6	26614419	793
S6_27634954	6	27634954	794
S6_27702886	6	27702886	794
S6_27747535	6	27747535	794
S6_27752794	6	27752794	794
S6_27762053	6	27762053	794
S6_27795800	6	27795800	795
S6_27795818	6	27795818	795
S6_27863356	6	27863356	796
S6_27871603	6	27871603	796
S6_27871641	6	27871641	796
S6_27871648	6	27871648	797
S6_27871649	6	27871649	797
S6_27871651	6	27871651	797
S6_27994413	6	27994413	798
S6_28002154	6	28002154	798

Markers	Chromosome	Position	Block #
S6_28189440	6	28189440	799
S6_28204708	6	28204708	799
S6_28260082	6	28260082	800
S6_28269548	6	28269548	800
S6_28269727	6	28269727	800
S6_28293511	6	28293511	801
S6_28294334	6	28294334	801
S6_28448546	6	28448546	802
S6_28450007	6	28450007	802
S6_28450082	6	28450082	802
S6_28613464	6	28613464	803
S6_28613482	6	28613482	803
S6_28645037	6	28645037	804
S6_28645050	6	28645050	804
S6_28919195	6	28919195	805
S6_28920735	6	28920735	805
S6_29092759	6	29092759	806
S6_29092777	6	29092777	806
S6_29141136	6	29141136	807
S6_29141156	6	29141156	807
S6_29174101	6	29174101	808
S6_29174112	6	29174112	808
S6_29180833	6	29180833	808
S6_29527928	6	29527928	809
S6_29531221	6	29531221	809
S6_29531236	6	29531236	809
S6_29546112	6	29546112	809
S7_14577	7	14577	810
S7_45233	7	45233	810
S7_82922	7	82922	810
S7_129216	7	129216	810
S7_142221	7	142221	811
S7_144340	7	144340	811
S7_176927	7	176927	812
S7_200572	7	200572	812
S7_211947	7	211947	812
S7_226086	7	226086	812
S7_226096	7	226096	812
S7_226112	7	226112	812
S7_226162	7	226162	812
S7_226279	7	226279	812
S7_248811	7	248811	813
S7_250280	7	250280	813
S7_250771	7	250771	813
S7_250902	7	250902	814
S7_252210	7	252210	814

Markers	Chromosome	Position	Block #
S7_272695	7	272695	815
S7_272705	7	272705	815
S7_276635	7	276635	815
S7_276649	7	276649	815
S7_276651	7	276651	815
S7_276653	7	276653	815
S7_276670	7	276670	815
S7_276682	7	276682	816
S7_276699	7	276699	816
S7_295198	7	295198	817
S7_302252	7	302252	817
S7_311815	7	311815	817
S7_317432	7	317432	817
S7_368182	7	368182	818
S7_368184	7	368184	818
S7_614603	7	614603	819
S7_617515	7	617515	819
S7_1044552	7	1044552	820
S7_1044582	7	1044582	820
S7_1069410	7	1069410	821
S7_1070829	7	1070829	821
S7_1106234	7	1106234	822
S7_1107947	7	1107947	822
S7_1213345	7	1213345	823
S7_1213435	7	1213435	823
S7_1338217	7	1338217	824
S7_1338224	7	1338224	824
S7_1338226	7	1338226	824
S7_1338238	7	1338238	824
S7_1484192	7	1484192	825
S7_1484995	7	1484995	825
S7_1962792	7	1962792	826
S7_1962819	7	1962819	826
S7_2035197	7	2035197	827
S7_2035204	7	2035204	827
S7_2434656	7	2434656	828
S7_2435959	7	2435959	828
S7_2437414	7	2437414	829
S7_2437415	7	2437415	829
S7_2438081	7	2438081	830
S7_2488638	7	2488638	830
S7_2610945	7	2610945	831
S7_2610978	7	2610978	831
S7_2641358	7	2641358	832
S7_2655314	7	2655314	832
S7_2655316	7	2655316	832

Markers	Chromosome	Position	Block #
S7_2676556	7	2676556	833
S7_2676606	7	2676606	833
S7_2677355	7	2677355	833
S7_2680359	7	2680359	833
S7_2725530	7	2725530	834
S7_2727866	7	2727866	834
S7_2729988	7	2729988	835
S7_2730017	7	2730017	835
S7_2760948	7	2760948	836
S7_2763311	7	2763311	836
S7_2763540	7	2763540	836
S7_2825983	7	2825983	837
S7_2825995	7	2825995	837
S7_3483273	7	3483273	838
S7_3483281	7	3483281	838
S7_4323647	7	4323647	839
S7_4323724	7	4323724	839
S7_4737787	7	4737787	840
S7_4753569	7	4753569	840
S7_4796769	7	4796769	841
S7_4802791	7	4802791	841
S7_4832357	7	4832357	842
S7_4832536	7	4832536	842
S7_4995467	7	4995467	843
S7_4997966	7	4997966	843
S7_5256974	7	5256974	844
S7_5259864	7	5259864	844
S7_5264321	7	5264321	845
S7_5264475	7	5264475	845
S7_5264506	7	5264506	845
S7_5327093	7	5327093	846
S7_5327109	7	5327109	846
S7_5381201	7	5381201	847
S7_5381344	7	5381344	847
S7_5445857	7	5445857	847
S7_5598747	7	5598747	848
S7_5598748	7	5598748	848
S7_5598749	7	5598749	848
S7_6185461	7	6185461	849
S7_6185462	7	6185462	849
S7_6185463	7	6185463	849
S7_6185464	7	6185464	849
S7_6220932	7	6220932	850
S7_6220933	7	6220933	850
S7_6673564	7	6673564	851
S7_6674353	7	6674353	851

Markers	Chromosome	Position	Block #
S7_6721461	7	6721461	851
S7_6721503	7	6721503	851
S7_6728457	7	6728457	851
S7_6795232	7	6795232	851
S7_6800840	7	6800840	852
S7_6888692	7	6888692	852
S7_7381601	7	7381601	853
S7_7381613	7	7381613	853
S7_7381752	7	7381752	853
S7_7689985	7	7689985	854
S7_7689986	7	7689986	854
S7_7689987	7	7689987	854
S7_7689988	7	7689988	854
S7_9205291	7	9205291	855
S7_9205296	7	9205296	855
S7_9364833	7	9364833	855
S7_9447413	7	9447413	856
S7_9447451	7	9447451	856
S7_9613998	7	9613998	857
S7_9614137	7	9614137	857
S7_9939850	7	9939850	858
S7_9940331	7	9940331	858
S7_10034504	7	10034504	858
S7_10039974	7	10039974	858
S7_10196472	7	10196472	859
S7_10221643	7	10221643	859
S7_10797836	7	10797836	860
S7_10797843	7	10797843	860
S7_10797933	7	10797933	860
S7_11103638	7	11103638	861
S7_11103792	7	11103792	861
S7_11119307	7	11119307	861
S7_11120892	7	11120892	861
S7_11332651	7	11332651	862
S7_11356316	7	11356316	862
S7_12077715	7	12077715	863
S7_12077799	7	12077799	863
S7_12542147	7	12542147	864
S7_12542148	7	12542148	864
S7_12542173	7	12542173	864
S7_12542334	7	12542334	865
S7_12542335	7	12542335	865
S7_12542336	7	12542336	865
S7_12918228	7	12918228	866
S7_13014054	7	13014054	866
S7_13591516	7	13591516	867

Markers	Chromosome	Position	Block #
S7_13596428	7	13596428	867
S7_13637597	7	13637597	867
S7_13637655	7	13637655	867
S7_13690465	7	13690465	867
S7_13694191	7	13694191	867
S7_13698182	7	13698182	867
S7_13719933	7	13719933	867
S7_13719958	7	13719958	867
S7_13991473	7	13991473	868
S7_14037250	7	14037250	868
S7_14147343	7	14147343	869
S7_14147354	7	14147354	869
S7_14618793	7	14618793	870
S7_14619774	7	14619774	870
S7_14702976	7	14702976	871
S7_14711393	7	14711393	871
S7_14711394	7	14711394	871
S7_14711404	7	14711404	872
S7_14711406	7	14711406	872
S7_14830113	7	14830113	873
S7_14830294	7	14830294	873
S7_15058583	7	15058583	874
S7_15058649	7	15058649	874
S7_15058650	7	15058650	874
S7_15058651	7	15058651	874
S7_15058656	7	15058656	874
S7_15229916	7	15229916	875
S7_15230544	7	15230544	875
S7_15517905	7	15517905	876
S7_15517912	7	15517912	876
S7_15517985	7	15517985	876
S7_15518020	7	15518020	876
S7_15680708	7	15680708	877
S7_15680816	7	15680816	877
S7_15701211	7	15701211	877
S7_15749852	7	15749852	878
S7_15749882	7	15749882	878
S7_15780042	7	15780042	878
S7_15897101	7	15897101	878
S7_17500421	7	17500421	879
S7_17500533	7	17500533	879
S7_17627895	7	17627895	880
S7_17628030	7	17628030	880
S7_17694172	7	17694172	881
S7_17765118	7	17765118	881
S7_17765123	7	17765123	881

Markers	Chromosome	Position	Block #
S7_17765161	7	17765161	882
S7_17770548	7	17770548	882
S7_18189649	7	18189649	883
S7_18211450	7	18211450	883
S7_18211556	7	18211556	884
S7_18211558	7	18211558	884
S7_18211732	7	18211732	885
S7_18307790	7	18307790	885
S7_18312175	7	18312175	885
S7_18391465	7	18391465	886
S7_18392200	7	18392200	886
S7_18392236	7	18392236	887
S7_18521349	7	18521349	887
S7_18804917	7	18804917	888
S7_18825236	7	18825236	888
S7_18825264	7	18825264	888
S7_19089573	7	19089573	889
S7_19135412	7	19135412	889
S7_19280115	7	19280115	890
S7_19280116	7	19280116	890
S7_19280117	7	19280117	890
S7_19284188	7	19284188	891
S7_19284189	7	19284189	891
S7_19284190	7	19284190	891
S7_19284191	7	19284191	891
S7_19284238	7	19284238	892
S7_19284278	7	19284278	892
S7_19484531	7	19484531	893
S7_19484542	7	19484542	893
S7_19507018	7	19507018	894
S7_19519544	7	19519544	894
S7_19520098	7	19520098	894
S7_19807132	7	19807132	895
S7_19814936	7	19814936	895
S7_19867715	7	19867715	896
S7_19867728	7	19867728	896
S7_20008792	7	20008792	897
S7_20008796	7	20008796	897
S7_20008894	7	20008894	897
S7_20022577	7	20022577	898
S7_20022706	7	20022706	898
S7_20024519	7	20024519	899
S7_20024520	7	20024520	899
S7_20024521	7	20024521	899
S7_20219527	7	20219527	900
S7_20221146	7	20221146	900

Markers	Chromosome	Position	Block #
S7_20221322	7	20221322	900
S7_20352824	7	20352824	900
S7_20352896	7	20352896	900
S7_20491729	7	20491729	901
S7_20491731	7	20491731	901
S7_20562770	7	20562770	902
S7_20567170	7	20567170	902
S7_20567180	7	20567180	902
S7_21029174	7	21029174	903
S7_21029215	7	21029215	903
S7_21032418	7	21032418	903
S7_21153478	7	21153478	904
S7_21153526	7	21153526	904
S7_21681012	7	21681012	905
S7_21681253	7	21681253	905
S7_21697389	7	21697389	906
S7_21697416	7	21697416	906
S7_21697521	7	21697521	906
S7_21846395	7	21846395	907
S7_21846396	7	21846396	907
S7_21846397	7	21846397	907
S7_21853633	7	21853633	908
S7_21853721	7	21853721	908
S7_21853767	7	21853767	908
S7_22195287	7	22195287	909
S7_22195288	7	22195288	909
S7_22195290	7	22195290	909
S7_22250439	7	22250439	910
S7_22250441	7	22250441	910
S7_22250442	7	22250442	910
S7_22250443	7	22250443	910
S7_22250444	7	22250444	910
S7_22264417	7	22264417	911
S7_22264418	7	22264418	911
S7_22264448	7	22264448	911
S7_22264465	7	22264465	911
S7_22282205	7	22282205	912
S7_22282212	7	22282212	912
S7_22282221	7	22282221	912
S7_22386051	7	22386051	913
S7_22386063	7	22386063	913
S7_22396054	7	22396054	913
S7_22396918	7	22396918	913
S7_22396942	7	22396942	913
S7_22396964	7	22396964	913
S7_22396990	7	22396990	913

Markers	Chromosome	Position	Block #
S7_22464460	7	22464460	914
S7_22464536	7	22464536	914
S7_22464581	7	22464581	914
S7_22496536	7	22496536	915
S7_22496560	7	22496560	915
S7_22496620	7	22496620	915
S7_22521435	7	22521435	916
S7_22521990	7	22521990	916
S7_22565791	7	22565791	917
S7_22585869	7	22585869	917
S7_22589967	7	22589967	917
S7_22589999	7	22589999	917
S7_22590056	7	22590056	917
S7_22590067	7	22590067	917
S7_22592335	7	22592335	918
S7_22592346	7	22592346	918
S7_22654547	7	22654547	919
S7_22654706	7	22654706	919
S7_22665372	7	22665372	920
S7_22665378	7	22665378	920
S7_23006904	7	23006904	921
S7_23007056	7	23007056	921
S7_23282061	7	23282061	922
S7_23290781	7	23290781	922
S7_23290906	7	23290906	922
S7_23299233	7	23299233	923
S7_23303405	7	23303405	923
S7_23303418	7	23303418	923
S7_23305745	7	23305745	923
S7_23311157	7	23311157	923
S7_23369025	7	23369025	924
S7_23369061	7	23369061	924
S7_23369074	7	23369074	924
S7_23378651	7	23378651	925
S7_23467123	7	23467123	925
S7_23467181	7	23467181	925
S7_23493169	7	23493169	926
S7_23495349	7	23495349	926
S7_23884186	7	23884186	927
S7_23884190	7	23884190	927
S7_24021872	7	24021872	928
S7_24021878	7	24021878	928
S7_24021879	7	24021879	928
S7_24065322	7	24065322	929
S7_24066878	7	24066878	929
S7_24072687	7	24072687	930

Markers	Chromosome	Position	Block #
S7_24072780	7	24072780	930
S7_24072817	7	24072817	931
S7_24072822	7	24072822	931
S7_24074894	7	24074894	931
S7_24074982	7	24074982	931
S7_24075071	7	24075071	931
S7_24094209	7	24094209	932
S7_24094210	7	24094210	932
S7_24542539	7	24542539	933
S7_24572520	7	24572520	933
S7_24586049	7	24586049	934
S7_24586050	7	24586050	934
S7_24586052	7	24586052	934
S7_24705704	7	24705704	935
S7_24709560	7	24709560	935
S8_113609	8	113609	936
S8_123227	8	123227	936
S8_123262	8	123262	936
S8_154155	8	154155	936
S8_154195	8	154195	936
S8_172140	8	172140	937
S8_186817	8	186817	937
S8_196583	8	196583	937
S8_207031	8	207031	937
S8_220621	8	220621	937
S8_235591	8	235591	937
S8_312130	8	312130	937
S8_312149	8	312149	937
S8_329316	8	329316	938
S8_329339	8	329339	938
S8_329369	8	329369	939
S8_329384	8	329384	939
S8_329405	8	329405	939
S8_329440	8	329440	939
S8_336300	8	336300	939
S8_336420	8	336420	939
S8_350862	8	350862	940
S8_374532	8	374532	940
S8_407789	8	407789	941
S8_408751	8	408751	941
S8_408752	8	408752	941
S8_409276	8	409276	941
S8_646666	8	646666	942
S8_678802	8	678802	942
S8_859273	8	859273	943
S8_859282	8	859282	943

Markers	Chromosome	Position	Block #
S8_859440	8	859440	943
S8_1136866	8	1136866	944
S8_1136867	8	1136867	944
S8_1213985	8	1213985	945
S8_1221818	8	1221818	945
S8_1221911	8	1221911	946
S8_1226471	8	1226471	946
S8_1254192	8	1254192	947
S8_1255335	8	1255335	947
S8_1339133	8	1339133	948
S8_1348701	8	1348701	948
S8_1490557	8	1490557	949
S8_1494116	8	1494116	949
S8_1494165	8	1494165	949
S8_1494349	8	1494349	949
S8_1541486	8	1541486	949
S8_1573926	8	1573926	950
S8_1573956	8	1573956	950
S8_2037236	8	2037236	951
S8_2037237	8	2037237	951
S8_2085144	8	2085144	952
S8_2085164	8	2085164	952
S8_2134958	8	2134958	953
S8_2134990	8	2134990	953
S8_2316226	8	2316226	954
S8_2316328	8	2316328	954
S8_2322026	8	2322026	954
S8_2551864	8	2551864	955
S8_2551950	8	2551950	955
S8_2558861	8	2558861	955
S8_2633472	8	2633472	956
S8_2633533	8	2633533	956
S8_2671645	8	2671645	957
S8_2671656	8	2671656	957
S8_2795992	8	2795992	958
S8_2795994	8	2795994	958
S8_2818844	8	2818844	959
S8_2818921	8	2818921	959
S8_2878637	8	2878637	960
S8_2878647	8	2878647	960
S8_3342265	8	3342265	961
S8_3342267	8	3342267	961
S8_3346083	8	3346083	962
S8_3346093	8	3346093	962
S8_3346186	8	3346186	963
S8_3346203	8	3346203	963

Markers	Chromosome	Position	Block #
S8_3346233	8	3346233	963
S8_3346236	8	3346236	963
S8_3378579	8	3378579	964
S8_3378608	8	3378608	964
S8_3378615	8	3378615	964
S8_3833920	8	3833920	965
S8_3836268	8	3836268	965
S8_3836284	8	3836284	965
S8_4261174	8	4261174	966
S8_4261175	8	4261175	966
S8_4303552	8	4303552	967
S8_4314363	8	4314363	967
S8_4450717	8	4450717	968
S8_4450720	8	4450720	968
S8_4490771	8	4490771	969
S8_4490776	8	4490776	969
S8_4744506	8	4744506	970
S8_4744508	8	4744508	970
S8_4744511	8	4744511	970
S8_4744514	8	4744514	970
S8_4805337	8	4805337	971
S8_4805338	8	4805338	971
S8_4890203	8	4890203	972
S8_4910084	8	4910084	972
S8_4911306	8	4911306	972
S8_5064054	8	5064054	973
S8_5064055	8	5064055	973
S8_5064752	8	5064752	973
S8_5071323	8	5071323	973
S8_5086050	8	5086050	973
S8_5096181	8	5096181	974
S8_5096292	8	5096292	974
S8_5322915	8	5322915	975
S8_5322918	8	5322918	975
S8_5324316	8	5324316	975
S8_5373587	8	5373587	976
S8_5381241	8	5381241	976
S8_5455330	8	5455330	977
S8_5487213	8	5487213	977
S8_5487248	8	5487248	977
S8_5494704	8	5494704	977
S8_5616976	8	5616976	978
S8_5623204	8	5623204	978
S8_5710291	8	5710291	979
S8_5710312	8	5710312	979
S8_5710318	8	5710318	979

Markers	Chromosome	Position	Block #
S8_5710321	8	5710321	979
S8_5710348	8	5710348	979
S8_5837517	8	5837517	980
S8_5837569	8	5837569	980
S8_5837730	8	5837730	981
S8_5837754	8	5837754	981
S8_5839144	8	5839144	981
S8_6125092	8	6125092	982
S8_6125200	8	6125200	982
S8_6243138	8	6243138	983
S8_6243141	8	6243141	983
S8_6244444	8	6244444	984
S8_6265636	8	6265636	984
S8_6291150	8	6291150	984
S8_6326878	8	6326878	984
S8_6327050	8	6327050	984
S8_6342669	8	6342669	984
S8_6462306	8	6462306	985
S8_6462307	8	6462307	985
S8_6462308	8	6462308	985
S8_6464624	8	6464624	986
S8_6464985	8	6464985	986
S8_6827621	8	6827621	987
S8_6845491	8	6845491	987
S8_6895515	8	6895515	988
S8_6895521	8	6895521	988
S8_6895530	8	6895530	988
S8_6980608	8	6980608	989
S8_7023749	8	7023749	989
S8_7054109	8	7054109	990
S8_7055401	8	7055401	990
S8_8133583	8	8133583	991
S8_8134963	8	8134963	991
S8_8179977	8	8179977	992
S8_8180039	8	8180039	992
S8_8391544	8	8391544	993
S8_8395532	8	8395532	993
S8_8454519	8	8454519	994
S8_8481454	8	8481454	994
S8_8485676	8	8485676	995
S8_8485814	8	8485814	995
S8_8486571	8	8486571	995
S8_8486667	8	8486667	995
S8_8496900	8	8496900	995
S8_8499120	8	8499120	995
S8_8499256	8	8499256	995

Markers	Chromosome	Position	Block #
S8_8567675	8	8567675	996
S8_8567741	8	8567741	996
S8_8596289	8	8596289	997
S8_8596334	8	8596334	997
S8_8677211	8	8677211	998
S8_8677228	8	8677228	998
S8_8677655	8	8677655	998
S8_8809574	8	8809574	999
S8_8809575	8	8809575	999
S8_8809577	8	8809577	999
S8_8809578	8	8809578	999
S8_9256764	8	9256764	1000
S8_9256783	8	9256783	1000
S8_9990658	8	9990658	1001
S8_9990696	8	9990696	1001
S8_9992964	8	9992964	1001
S8_10003037	8	10003037	1001
S8_10232237	8	10232237	1002
S8_10239335	8	10239335	1002
S8_11747278	8	11747278	1003
S8_11747320	8	11747320	1003
S8_12506367	8	12506367	1004
S8_12506368	8	12506368	1004
S8_13319277	8	13319277	1005
S8_13324329	8	13324329	1005
S8_13616683	8	13616683	1006
S8_13616700	8	13616700	1006
S8_14171827	8	14171827	1007
S8_14171934	8	14171934	1007
S8_14197720	8	14197720	1008
S8_14202528	8	14202528	1008
S8_14386456	8	14386456	1009
S8_14440342	8	14440342	1009
S8_14442202	8	14442202	1009
S8_14758503	8	14758503	1010
S8_14760052	8	14760052	1010
S8_14874102	8	14874102	1011
S8_14874167	8	14874167	1011
S8_16190621	8	16190621	1012
S8_16251233	8	16251233	1012
S8_16251323	8	16251323	1012
S8_16561801	8	16561801	1013
S8_16609647	8	16609647	1013
S8_16615408	8	16615408	1013
S8_16619432	8	16619432	1013
S8_16854404	8	16854404	1014

Markers	Chromosome	Position	Block #
S8_16854422	8	16854422	1014
S8_16940645	8	16940645	1015
S8_16940653	8	16940653	1015
S8_17011797	8	17011797	1015
S8_17011798	8	17011798	1015
S8_17079996	8	17079996	1016
S8_17195205	8	17195205	1016
S8_17195206	8	17195206	1016
S8_17195268	8	17195268	1016
S8_17195352	8	17195352	1016
S8_17195354	8	17195354	1016
S8_17828695	8	17828695	1017
S8_17865920	8	17865920	1017
S8_18054923	8	18054923	1018
S8_18054924	8	18054924	1018
S8_18054925	8	18054925	1018
S8_18054930	8	18054930	1018
S8_18147331	8	18147331	1019
S8_18246933	8	18246933	1019
S8_18515778	8	18515778	1020
S8_18515793	8	18515793	1020
S8_18696582	8	18696582	1021
S8_18750115	8	18750115	1021
S8_18763862	8	18763862	1022
S8_18763877	8	18763877	1022
S8_19664737	8	19664737	1023
S8_19664752	8	19664752	1023
S8_20559515	8	20559515	1024
S8_20559547	8	20559547	1024
S8_20849893	8	20849893	1025
S8_20849894	8	20849894	1025
S8_20893478	8	20893478	1025
S8_21365821	8	21365821	1026
S8_21365823	8	21365823	1026
S8_21492521	8	21492521	1027
S8_21492522	8	21492522	1027
S8_21492523	8	21492523	1027
S8_21665318	8	21665318	1028
S8_21665434	8	21665434	1028
S8_21675763	8	21675763	1029
S8_21689519	8	21689519	1029
S8_21719128	8	21719128	1029
S8_21719163	8	21719163	1029
S8_21721599	8	21721599	1029
S8_22216940	8	22216940	1030
S8_22216964	8	22216964	1030

Markers	Chromosome	Position	Block #
S8_22474357	8	22474357	1031
S8_22474358	8	22474358	1031
S8_22704205	8	22704205	1032
S8_22704219	8	22704219	1032
S8_23122211	8	23122211	1033
S8_23125253	8	23125253	1033
S8_23229900	8	23229900	1034
S8_23229908	8	23229908	1034
S8_23259454	8	23259454	1035
S8_23259477	8	23259477	1035
S8_23276145	8	23276145	1035
S8_23280855	8	23280855	1036
S8_23280921	8	23280921	1036
S8_23287645	8	23287645	1036
S8_23491657	8	23491657	1037
S8_23491678	8	23491678	1037
S8_23491704	8	23491704	1037
S8_23491706	8	23491706	1037
S8_23578708	8	23578708	1038
S8_23670544	8	23670544	1038
S8_23834259	8	23834259	1039
S8_23904383	8	23904383	1039
S8_24022489	8	24022489	1040
S8_24107075	8	24107075	1040
S9_1327	9	1327	1041
S9_1391	9	1391	1041
S9_35658	9	35658	1041
S9_88935	9	88935	1042
S9_151390	9	151390	1042
S9_151405	9	151405	1042
S9_151492	9	151492	1042
S9_151510	9	151510	1043
S9_167601	9	167601	1043
S9_168205	9	168205	1044
S9_170704	9	170704	1044
S9_177091	9	177091	1045
S9_177123	9	177123	1045
S9_290206	9	290206	1046
S9_303830	9	303830	1046
S9_303961	9	303961	1046
S9_304014	9	304014	1046
S9_304038	9	304038	1046
S9_310308	9	310308	1046
S9_310336	9	310336	1047
S9_343084	9	343084	1047
S9_504661	9	504661	1048

Markers	Chromosome	Position	Block #
S9_504670	9	504670	1048
S9_540689	9	540689	1049
S9_540700	9	540700	1049
S9_545323	9	545323	1050
S9_545437	9	545437	1050
S9_545530	9	545530	1050
S9_545554	9	545554	1050
S9_641350	9	641350	1051
S9_641362	9	641362	1051
S9_768453	9	768453	1052
S9_768470	9	768470	1052
S9_779650	9	779650	1053
S9_779667	9	779667	1053
S9_779757	9	779757	1054
S9_779764	9	779764	1054
S9_811474	9	811474	1055
S9_811486	9	811486	1055
S9_811614	9	811614	1055
S9_860999	9	860999	1056
S9_861010	9	861010	1056
S9_861028	9	861028	1056
S9_1109849	9	1109849	1057
S9_1109867	9	1109867	1057
S9_1109906	9	1109906	1057
S9_1143401	9	1143401	1057
S9_1143456	9	1143456	1057
S9_1149544	9	1149544	1057
S9_1166898	9	1166898	1057
S9_1166910	9	1166910	1057
S9_1166930	9	1166930	1057
S9_1167001	9	1167001	1057
S9_1540368	9	1540368	1058
S9_1545309	9	1545309	1058
S9_1556691	9	1556691	1058
S9_1556692	9	1556692	1058
S9_1732810	9	1732810	1059
S9_1732869	9	1732869	1059
S9_1732892	9	1732892	1059
S9_2556989	9	2556989	1060
S9_2556996	9	2556996	1060
S9_3141873	9	3141873	1061
S9_3141881	9	3141881	1061
S9_3466385	9	3466385	1062
S9_3468796	9	3468796	1062
S9_3876843	9	3876843	1063
S9_4014494	9	4014494	1063

Markers	Chromosome	Position	Block #
S9_4191489	9	4191489	1064
S9_4223532	9	4223532	1064
S9_4223533	9	4223533	1065
S9_4258181	9	4258181	1065
S9_4258281	9	4258281	1065
S9_4258396	9	4258396	1065
S9_4259479	9	4259479	1065
S9_4259571	9	4259571	1065
S9_4260860	9	4260860	1065
S9_4308164	9	4308164	1066
S9_4308198	9	4308198	1066
S9_4462963	9	4462963	1067
S9_4465017	9	4465017	1067
S9_4535923	9	4535923	1068
S9_4535924	9	4535924	1068
S9_4653828	9	4653828	1069
S9_4653830	9	4653830	1069
S9_4670131	9	4670131	1069
S9_4670153	9	4670153	1069
S9_5057080	9	5057080	1070
S9_5057152	9	5057152	1070
S9_5546816	9	5546816	1071
S9_5559056	9	5559056	1071
S9_5724665	9	5724665	1072
S9_5724668	9	5724668	1072
S9_6087775	9	6087775	1073
S9_6087817	9	6087817	1073
S9_6087820	9	6087820	1073
S9_6251546	9	6251546	1074
S9_6251568	9	6251568	1074
S9_6251637	9	6251637	1075
S9_6251656	9	6251656	1075
S9_6478721	9	6478721	1076
S9_6485739	9	6485739	1076
S9_7009141	9	7009141	1077
S9_7068485	9	7068485	1077
S9_7995766	9	7995766	1078
S9_7996638	9	7996638	1078
S9_8031462	9	8031462	1078
S9_8427971	9	8427971	1079
S9_8431491	9	8431491	1079
S9_8431492	9	8431492	1079
S9_8691945	9	8691945	1080
S9_8691946	9	8691946	1080
S9_8691986	9	8691986	1080
S9_8692016	9	8692016	1080

Markers	Chromosome	Position	Block #
S9_8692057	9	8692057	1080
S9_8715989	9	8715989	1080
S9_8741703	9	8741703	1080
S9_9232996	9	9232996	1081
S9_9235284	9	9235284	1081
S9_9316293	9	9316293	1082
S9_9316338	9	9316338	1082
S9_9417371	9	9417371	1083
S9_9417417	9	9417417	1083
S9_9417707	9	9417707	1083
S9_9421604	9	9421604	1083
S9_9421630	9	9421630	1083
S9_9427009	9	9427009	1083
S9_9446159	9	9446159	1083
S9_9452230	9	9452230	1083
S9_9452297	9	9452297	1083
S9_13475306	9	13475306	1084
S9_13476479	9	13476479	1084
S9_13479997	9	13479997	1085
S9_13479999	9	13479999	1085
S9_13480000	9	13480000	1085
S9_13483685	9	13483685	1086
S9_13484202	9	13484202	1086
S9_13484301	9	13484301	1086
S9_13639097	9	13639097	1087
S9_13639103	9	13639103	1087
S9_14237173	9	14237173	1088
S9_14237181	9	14237181	1088
S9_14277318	9	14277318	1088
S9_15034871	9	15034871	1089
S9_15040549	9	15040549	1089
S9_15617413	9	15617413	1090
S9_15617461	9	15617461	1090
S9_15617632	9	15617632	1090
S9_15732341	9	15732341	1090
S9_15773062	9	15773062	1090
S9_16140442	9	16140442	1091
S9_16140564	9	16140564	1091
S9_16212944	9	16212944	1091
S9_16258884	9	16258884	1091
S9_16263582	9	16263582	1091
S9_16263583	9	16263583	1091
S9_16443347	9	16443347	1092
S9_16443399	9	16443399	1092
S9_17455120	9	17455120	1093
S9_17455123	9	17455123	1093

Markers	Chromosome	Position	Block #
S9_17455149	9	17455149	1094
S9_17455171	9	17455171	1094
S9_17455205	9	17455205	1095
S9_17455216	9	17455216	1095
S9_17455236	9	17455236	1095
S9_17455248	9	17455248	1095
S9_17550491	9	17550491	1096
S9_17555469	9	17555469	1096
S9_17575809	9	17575809	1097
S9_17575859	9	17575859	1097
S9_17575879	9	17575879	1097
S9_17576318	9	17576318	1098
S9_17576327	9	17576327	1098
S9_17576343	9	17576343	1099
S9_17580874	9	17580874	1099
S9_18460952	9	18460952	1100
S9_18460999	9	18460999	1100
S9_18461607	9	18461607	1100
S9_18461647	9	18461647	1100
S9_18814147	9	18814147	1101
S9_18814174	9	18814174	1101
S9_19074660	9	19074660	1102
S9_19098398	9	19098398	1102
S9_19098424	9	19098424	1102
S9_19098532	9	19098532	1102
S9_19100567	9	19100567	1102
S9_19106241	9	19106241	1102
S9_19488105	9	19488105	1103
S9_19488129	9	19488129	1103
S9_19509850	9	19509850	1104
S9_19509851	9	19509851	1104
S9_19509852	9	19509852	1104
S9_19697053	9	19697053	1105
S9_19715019	9	19715019	1105
S9_20089889	9	20089889	1106
S9_20089945	9	20089945	1106
S9_20179596	9	20179596	1107
S9_20208649	9	20208649	1107
S9_20251540	9	20251540	1108
S9_20252472	9	20252472	1108
S9_20430701	9	20430701	1109
S9_20430712	9	20430712	1109
S9_20430902	9	20430902	1110
S9_20430903	9	20430903	1110
S9_20497046	9	20497046	1111
S9_20497047	9	20497047	1111

Markers	Chromosome	Position	Block #
S9_20502687	9	20502687	1111
S9_20502836	9	20502836	1111
S9_20502881	9	20502881	1111
S9_20589016	9	20589016	1112
S9_20589018	9	20589018	1112
S9_20714401	9	20714401	1113
S9_20714418	9	20714418	1113
S9_20714420	9	20714420	1113
S9_20714466	9	20714466	1113
S9_20714554	9	20714554	1113
S9_20714644	9	20714644	1113
S9_20762720	9	20762720	1114
S9_20762761	9	20762761	1114
S9_20762843	9	20762843	1115
S9_20762858	9	20762858	1115
S9_20767085	9	20767085	1116
S9_20767111	9	20767111	1116
S9_20776433	9	20776433	1117
S9_20776438	9	20776438	1117
S9_20776444	9	20776444	1118
S9_20776473	9	20776473	1118
S9_20776474	9	20776474	1118
S9_20776517	9	20776517	1118
S9_20777991	9	20777991	1119
S9_20777992	9	20777992	1119
S9_20834611	9	20834611	1120
S9_20834689	9	20834689	1120
S9_21203130	9	21203130	1121
S9_21203131	9	21203131	1121
S9_21505138	9	21505138	1122
S9_21505188	9	21505188	1122
S9_21652254	9	21652254	1123
S9_21652434	9	21652434	1123
S9_21733484	9	21733484	1124
S9_21734177	9	21734177	1124
S9_21737950	9	21737950	1125
S9_21738139	9	21738139	1125
S9_21738140	9	21738140	1125
S9_22033231	9	22033231	1126
S9_22033240	9	22033240	1126
S9_22057864	9	22057864	1127
S9_22093357	9	22093357	1127
S9_22113253	9	22113253	1128
S9_22113557	9	22113557	1128
S9_22154572	9	22154572	1129
S9_22159336	9	22159336	1129

Markers	Chromosome	Position	Block #
S9_22254315	9	22254315	1129
S9_22382080	9	22382080	1130
S9_22382098	9	22382098	1130
S9_22531898	9	22531898	1131
S9_22531924	9	22531924	1131
S9_22859273	9	22859273	1132
S9_22859387	9	22859387	1132
S9_22897125	9	22897125	1133
S9_22897208	9	22897208	1133
S9_23118141	9	23118141	1134
S9_23118178	9	23118178	1134
S9_23118186	9	23118186	1134
S9_23118202	9	23118202	1134
S9_23128498	9	23128498	1135
S9_23242572	9	23242572	1135
S9_23293192	9	23293192	1136
S9_23319225	9	23319225	1136
S9_23329822	9	23329822	1136
S9_23442367	9	23442367	1136
S9_23442459	9	23442459	1136
S9_23445705	9	23445705	1136
S9_23744870	9	23744870	1137
S9_23744881	9	23744881	1137
S9_23943308	9	23943308	1138
S9_23943320	9	23943320	1138
S9_24058424	9	24058424	1139
S9_24105992	9	24105992	1139
S10_38125	10	38125	1140
S10_68527	10	68527	1140
S10_71248	10	71248	1140
S10_77384	10	77384	1140
S10_91258	10	91258	1140
S10_94131	10	94131	1140
S10_99499	10	99499	1140
S10_111359	10	111359	1141
S10_111370	10	111370	1141
S10_231901	10	231901	1141
S10_405964	10	405964	1142
S10_441209	10	441209	1142
S10_441737	10	441737	1142
S10_443437	10	443437	1143
S10_443512	10	443512	1143
S10_604024	10	604024	1144
S10_604043	10	604043	1144
S10_604071	10	604071	1144
S10_711705	10	711705	1145

Markers	Chromosome	Position	Block #
S10_711803	10	711803	1145
S10_711868	10	711868	1145
S10_720305	10	720305	1146
S10_724764	10	724764	1146
S10_724795	10	724795	1146
S10_746143	10	746143	1147
S10_746144	10	746144	1147
S10_746145	10	746145	1147
S10_747681	10	747681	1148
S10_747696	10	747696	1148
S10_747828	10	747828	1148
S10_748099	10	748099	1148
S10_757253	10	757253	1148
S10_1010796	10	1010796	1149
S10_1021141	10	1021141	1149
S10_1039591	10	1039591	1150
S10_1039597	10	1039597	1150
S10_1202206	10	1202206	1151
S10_1203261	10	1203261	1151
S10_1232666	10	1232666	1152
S10_1232844	10	1232844	1152
S10_1232862	10	1232862	1152
S10_1291780	10	1291780	1153
S10_1291921	10	1291921	1153
S10_1376969	10	1376969	1154
S10_1376981	10	1376981	1154
S10_1398632	10	1398632	1155
S10_1398683	10	1398683	1155
S10_1415805	10	1415805	1156
S10_1415860	10	1415860	1156
S10_1438806	10	1438806	1157
S10_1438871	10	1438871	1157
S10_1441130	10	1441130	1157
S10_1446217	10	1446217	1157
S10_1448387	10	1448387	1157
S10_1448401	10	1448401	1157
S10_1448499	10	1448499	1157
S10_1486096	10	1486096	1158
S10_1486105	10	1486105	1158
S10_1486148	10	1486148	1159
S10_1486749	10	1486749	1159
S10_1498840	10	1498840	1160
S10_1499219	10	1499219	1160
S10_1499276	10	1499276	1160
S10_1709565	10	1709565	1161
S10_1709640	10	1709640	1161

Markers	Chromosome	Position	Block #
S10_1777091	10	1777091	1162
S10_1785453	10	1785453	1162
S10_2206450	10	2206450	1163
S10_2207870	10	2207870	1163
S10_2227433	10	2227433	1164
S10_2227434	10	2227434	1164
S10_2227543	10	2227543	1165
S10_2227568	10	2227568	1165
S10_2246820	10	2246820	1166
S10_2251704	10	2251704	1166
S10_2393585	10	2393585	1167
S10_2393839	10	2393839	1167
S10_2603419	10	2603419	1168
S10_2603628	10	2603628	1168
S10_2684693	10	2684693	1169
S10_2715483	10	2715483	1169
S10_2777014	10	2777014	1170
S10_2777015	10	2777015	1170
S10_2777016	10	2777016	1170
S10_2842851	10	2842851	1171
S10_2848636	10	2848636	1171
S10_3299693	10	3299693	1172
S10_3356546	10	3356546	1172
S10_3356695	10	3356695	1173
S10_3357756	10	3357756	1173
S10_3380555	10	3380555	1174
S10_3380584	10	3380584	1174
S10_3415789	10	3415789	1174
S10_3443111	10	3443111	1175
S10_3443138	10	3443138	1175
S10_3541676	10	3541676	1176
S10_3541866	10	3541866	1176
S10_3649694	10	3649694	1177
S10_3667586	10	3667586	1177
S10_3677010	10	3677010	1177
S10_3775830	10	3775830	1177
S10_3775848	10	3775848	1177
S10_3802662	10	3802662	1177
S10_3802858	10	3802858	1177
S10_3806705	10	3806705	1177
S10_3827075	10	3827075	1178
S10_3870253	10	3870253	1178
S10_3905231	10	3905231	1179
S10_3939171	10	3939171	1179
S10_3946736	10	3946736	1179
S10_3946889	10	3946889	1179

Markers	Chromosome	Position	Block #
S10_3946894	10	3946894	1179
S10_3946922	10	3946922	1179
S10_3946931	10	3946931	1179
S10_3946964	10	3946964	1179
S10_3946988	10	3946988	1179
S10_3947000	10	3947000	1179
S10_4210686	10	4210686	1180
S10_4226224	10	4226224	1180
S10_4381310	10	4381310	1181
S10_4402874	10	4402874	1181
S10_4620467	10	4620467	1182
S10_4620504	10	4620504	1182
S10_4623463	10	4623463	1182
S10_4651214	10	4651214	1182
S10_4651262	10	4651262	1182
S10_4712268	10	4712268	1182
S10_4765849	10	4765849	1183
S10_4772501	10	4772501	1183
S10_4890428	10	4890428	1184
S10_4890664	10	4890664	1184
S10_5013281	10	5013281	1185
S10_5013289	10	5013289	1185
S10_5018218	10	5018218	1185
S10_5317405	10	5317405	1186
S10_5319676	10	5319676	1186
S10_5352241	10	5352241	1187
S10_5352262	10	5352262	1187
S10_5401860	10	5401860	1188
S10_5401887	10	5401887	1188
S10_5401922	10	5401922	1189
S10_5403341	10	5403341	1189
S10_5403382	10	5403382	1189
S10_5743914	10	5743914	1190
S10_5757812	10	5757812	1190
S10_5774449	10	5774449	1191
S10_5790263	10	5790263	1191
S10_5790276	10	5790276	1191
S10_6748523	10	6748523	1192
S10_6748526	10	6748526	1192
S10_7018236	10	7018236	1193
S10_7018253	10	7018253	1193
S10_7018503	10	7018503	1193
S10_7376951	10	7376951	1194
S10_7376997	10	7376997	1194
S10_7667839	10	7667839	1195
S10_7721606	10	7721606	1195

Markers	Chromosome	Position	Block #
S10_9155852	10	9155852	1196
S10_9156617	10	9156617	1196
S10_9370771	10	9370771	1197
S10_9393279	10	9393279	1197
S10_9393284	10	9393284	1197
S10_9393316	10	9393316	1197
S10_9706319	10	9706319	1198
S10_9706320	10	9706320	1198
S10_9706321	10	9706321	1198
S10_10270718	10	10270718	1199
S10_10270766	10	10270766	1199
S10_10311344	10	10311344	1200
S10_10311849	10	10311849	1200
S10_11671624	10	11671624	1201
S10_11671672	10	11671672	1201
S10_11724294	10	11724294	1201
S10_11734542	10	11734542	1201
S10_11734628	10	11734628	1201
S10_11734711	10	11734711	1201
S10_11736190	10	11736190	1201
S10_11753315	10	11753315	1202
S10_11753330	10	11753330	1202
S10_11753361	10	11753361	1203
S10_11763990	10	11763990	1203
S10_11764010	10	11764010	1203
S10_12080880	10	12080880	1204
S10_12084150	10	12084150	1204
S10_12393034	10	12393034	1205
S10_12393035	10	12393035	1205
S10_12393069	10	12393069	1205
S10_12393092	10	12393092	1205
S10_13141301	10	13141301	1206
S10_13141311	10	13141311	1206
S10_13362887	10	13362887	1207
S10_13362917	10	13362917	1207
S10_13362940	10	13362940	1207
S10_13807940	10	13807940	1208
S10_13966767	10	13966767	1208
S10_13988557	10	13988557	1209
S10_14047711	10	14047711	1209
S10_14047776	10	14047776	1209
S10_14047809	10	14047809	1209
S10_14047898	10	14047898	1209
S10_14564819	10	14564819	1210
S10_14564820	10	14564820	1210
S10_14651936	10	14651936	1211

Markers	Chromosome	Position	Block #
S10_14692420	10	14692420	1211
S10_14692442	10	14692442	1211
S10_14939979	10	14939979	1212
S10_14939980	10	14939980	1212
S10_14940060	10	14940060	1213
S10_14940093	10	14940093	1213
S10_14959578	10	14959578	1214
S10_14959592	10	14959592	1214
S10_15269521	10	15269521	1215
S10_15269555	10	15269555	1215
S10_15768156	10	15768156	1216
S10_15768170	10	15768170	1216
S11_28659	11	28659	1217
S11_33212	11	33212	1217
S11_46545	11	46545	1217
S11_46584	11	46584	1217
S11_46619	11	46619	1217
S11_51990	11	51990	1217
S11_53325	11	53325	1217
S11_131216	11	131216	1218
S11_135599	11	135599	1218
S11_281031	11	281031	1219
S11_281035	11	281035	1219
S11_281036	11	281036	1219
S11_479034	11	479034	1220
S11_500910	11	500910	1220
S11_541028	11	541028	1220
S11_606034	11	606034	1221
S11_672728	11	672728	1221
S11_1068801	11	1068801	1222
S11_1068804	11	1068804	1222
S11_1229670	11	1229670	1223
S11_1231800	11	1231800	1223
S11_1233477	11	1233477	1223
S11_1325831	11	1325831	1224
S11_1325847	11	1325847	1224
S11_1337626	11	1337626	1225
S11_1337761	11	1337761	1225
S11_1356826	11	1356826	1225
S11_1380885	11	1380885	1225
S11_1380920	11	1380920	1225
S11_1380990	11	1380990	1226
S11_1382918	11	1382918	1226
S11_1417567	11	1417567	1227
S11_1460770	11	1460770	1227
S11_1462355	11	1462355	1227

Markers	Chromosome	Position	Block #
S11_1581824	11	1581824	1228
S11_1581851	11	1581851	1228
S11_1724609	11	1724609	1229
S11_1724615	11	1724615	1229
S11_1724797	11	1724797	1229
S11_1725698	11	1725698	1229
S11_1725894	11	1725894	1229
S11_1726969	11	1726969	1229
S11_1727143	11	1727143	1229
S11_1731653	11	1731653	1229
S11_2008668	11	2008668	1230
S11_2012678	11	2012678	1230
S11_2012817	11	2012817	1231
S11_2071043	11	2071043	1231
S11_2485963	11	2485963	1232
S11_2557173	11	2557173	1232
S11_2621445	11	2621445	1233
S11_2621446	11	2621446	1233
S11_2624112	11	2624112	1233
S11_3227590	11	3227590	1234
S11_3247921	11	3247921	1234
S11_3327154	11	3327154	1234
S11_3387303	11	3387303	1235
S11_3387327	11	3387327	1235
S11_3391273	11	3391273	1236
S11_3396418	11	3396418	1236
S11_3403527	11	3403527	1236
S11_3433803	11	3433803	1237
S11_3433805	11	3433805	1237
S11_3433835	11	3433835	1237
S11_3454413	11	3454413	1237
S11_3478148	11	3478148	1237
S11_3968947	11	3968947	1238
S11_3968950	11	3968950	1238
S11_3970093	11	3970093	1239
S11_3970102	11	3970102	1239
S11_4177786	11	4177786	1240
S11_4177853	11	4177853	1240
S11_4177870	11	4177870	1240
S11_4261063	11	4261063	1241
S11_4315854	11	4315854	1241
S11_4396488	11	4396488	1242
S11_4396676	11	4396676	1242
S11_4396835	11	4396835	1242
S11_4403094	11	4403094	1242
S11_4403190	11	4403190	1242

Markers	Chromosome	Position	Block #
S11_4483697	11	4483697	1243
S11_4572819	11	4572819	1243
S11_4858156	11	4858156	1244
S11_4932526	11	4932526	1244
S11_4991234	11	4991234	1245
S11_4991254	11	4991254	1245
S11_4991344	11	4991344	1245
S11_4997766	11	4997766	1246
S11_4997829	11	4997829	1246
S11_5694008	11	5694008	1247
S11_5716486	11	5716486	1247
S11_5716518	11	5716518	1248
S11_5716519	11	5716519	1248
S11_6143661	11	6143661	1249
S11_6143703	11	6143703	1249
S11_6540273	11	6540273	1250
S11_6540319	11	6540319	1250
S11_6692070	11	6692070	1251
S11_6692072	11	6692072	1251
S11_6692073	11	6692073	1251
S11_6692074	11	6692074	1251
S11_7153732	11	7153732	1252
S11_7153754	11	7153754	1252
S11_7153763	11	7153763	1252
S11_7294402	11	7294402	1252
S11_7294548	11	7294548	1252
S11_7581953	11	7581953	1253
S11_7590511	11	7590511	1253
S11_7755317	11	7755317	1254
S11_7755324	11	7755324	1254
S11_7877392	11	7877392	1254
S11_7950823	11	7950823	1255
S11_7953675	11	7953675	1255
S11_7955258	11	7955258	1255
S11_8262863	11	8262863	1256
S11_8262880	11	8262880	1256
S11_8656444	11	8656444	1257
S11_8703314	11	8703314	1257
S11_8703350	11	8703350	1257
S11_8783466	11	8783466	1257
S11_8783583	11	8783583	1257
S11_9214087	11	9214087	1258
S11_9253439	11	9253439	1258
S11_9253458	11	9253458	1258
S11_9320544	11	9320544	1258
S11_9881674	11	9881674	1259

Markers	Chromosome	Position	Block #
S11_10006867	11	10006867	1259
S11_10204016	11	10204016	1260
S11_10348094	11	10348094	1260
S11_10351965	11	10351965	1260
S11_10352585	11	10352585	1260
S11_10482420	11	10482420	1261
S11_10604340	11	10604340	1261
S11_10604388	11	10604388	1261
S11_11255498	11	11255498	1262
S11_11385820	11	11385820	1262
S11_11385866	11	11385866	1262
S11_11422523	11	11422523	1263
S11_11576622	11	11576622	1263
S11_11586618	11	11586618	1264
S11_11638658	11	11638658	1264
S11_11638659	11	11638659	1264
S11_11910298	11	11910298	1265
S11_11923233	11	11923233	1265
S11_11963281	11	11963281	1265
S11_11998928	11	11998928	1266
S11_12123995	11	12123995	1266
S11_12358915	11	12358915	1267
S11_12359206	11	12359206	1267
S11_12470979	11	12470979	1267
S11_12470996	11	12470996	1267
S11_12471022	11	12471022	1267
S11_13208976	11	13208976	1268
S11_13208997	11	13208997	1268
S11_14061081	11	14061081	1269
S11_14061082	11	14061082	1269
S11_14986475	11	14986475	1270
S11_14987387	11	14987387	1270
S11_15019160	11	15019160	1270
S11_15075142	11	15075142	1270
S11_15075182	11	15075182	1270
S11_15091873	11	15091873	1270
S11_15890783	11	15890783	1271
S11_15890787	11	15890787	1271
S11_15890797	11	15890797	1271
S11_15890809	11	15890809	1271
S11_16009913	11	16009913	1271
S11_16017096	11	16017096	1271
S11_16091614	11	16091614	1272
S11_16091634	11	16091634	1272
S11_16103058	11	16103058	1272
S11_16190823	11	16190823	1272

Markers	Chromosome	Position	Block #
S11_16218609	11	16218609	1272
S11_16252288	11	16252288	1273
S11_16252537	11	16252537	1273
S11_16252990	11	16252990	1273
S11_16255267	11	16255267	1273
S11_16368412	11	16368412	1274
S11_16401326	11	16401326	1274
S11_16499762	11	16499762	1274
S11_16709151	11	16709151	1275
S11_16709154	11	16709154	1275
S11_16709155	11	16709155	1275
S11_16752817	11	16752817	1276
S11_16757467	11	16757467	1276
S11_16758855	11	16758855	1276
S11_16786270	11	16786270	1277
S11_16786465	11	16786465	1277
S11_16808056	11	16808056	1277
S11_16873673	11	16873673	1277
S11_16890437	11	16890437	1277
S11_16890442	11	16890442	1277
S11_17111963	11	17111963	1278
S11_17179905	11	17179905	1278
S11_17244284	11	17244284	1278
S11_17256854	11	17256854	1278
S11_17612413	11	17612413	1279
S11_17612420	11	17612420	1279
S11_17612423	11	17612423	1279
S11_17612424	11	17612424	1279
S11_17650099	11	17650099	1279
S11_17650109	11	17650109	1279
S11_17711420	11	17711420	1280
S11_17711444	11	17711444	1280
S11_17848873	11	17848873	1280
S11_17848885	11	17848885	1280
S11_18695690	11	18695690	1281
S11_18698502	11	18698502	1281
S11_18698530	11	18698530	1281
S11_18698531	11	18698531	1281
S11_18698622	11	18698622	1281
S11_18924670	11	18924670	1282
S11_18924689	11	18924689	1282
S11_18924755	11	18924755	1282
S11_19121917	11	19121917	1283
S11_19134172	11	19134172	1283
S11_19251738	11	19251738	1283
S11_19616979	11	19616979	1284

Markers	Chromosome	Position	Block #
S11_19616997	11	19616997	1284
S11_19617110	11	19617110	1284
S11_19647690	11	19647690	1284
S11_19647692	11	19647692	1284
S11_20391459	11	20391459	1285
S11_20391466	11	20391466	1285
S11_20391483	11	20391483	1285
S11_20726709	11	20726709	1286
S11_20726764	11	20726764	1286
S11_20796769	11	20796769	1287
S11_20925900	11	20925900	1287
S11_21056055	11	21056055	1288
S11_21069706	11	21069706	1288
S11_21069739	11	21069739	1288
S11_21069859	11	21069859	1288
S11_21294914	11	21294914	1289
S11_21349831	11	21349831	1289
S11_21353380	11	21353380	1289
S11_21353459	11	21353459	1289
S11_21591279	11	21591279	1290
S11_21591326	11	21591326	1290
S11_21660462	11	21660462	1290
S11_21718635	11	21718635	1290
S11_21722613	11	21722613	1290
S11_21742524	11	21742524	1290
S11_22019819	11	22019819	1291
S11_22022626	11	22022626	1291
S11_22023380	11	22023380	1291
S11_22157260	11	22157260	1292
S11_22157266	11	22157266	1292
S11_22386456	11	22386456	1293
S11_22386457	11	22386457	1293
S11_22629114	11	22629114	1294
S11_22629507	11	22629507	1294
S11_22779181	11	22779181	1295
S11_22800505	11	22800505	1295
S11_22857466	11	22857466	1296
S11_22861038	11	22861038	1296
S11_22891696	11	22891696	1297
S11_22891698	11	22891698	1297
S11_23068032	11	23068032	1298
S11_23068075	11	23068075	1298
S11_23075097	11	23075097	1298
S11_23079657	11	23079657	1298
S11_23079753	11	23079753	1298
S11_23105595	11	23105595	1299

Markers	Chromosome	Position	Block #
S11_23105689	11	23105689	1299
S11_23106176	11	23106176	1299
S11_23213834	11	23213834	1300
S11_23213858	11	23213858	1300
S11_23272945	11	23272945	1301
S11_23272987	11	23272987	1301
S11_23332317	11	23332317	1302
S11_23332331	11	23332331	1302
S11_23337294	11	23337294	1303
S11_23337452	11	23337452	1303
S11_23449871	11	23449871	1304
S11_23450053	11	23450053	1304
S11_23450054	11	23450054	1304
S11_23451756	11	23451756	1304
S11_23464167	11	23464167	1305
S11_23464209	11	23464209	1305
S11_23464432	11	23464432	1305
S11_23464438	11	23464438	1305
S11_23678567	11	23678567	1306
S11_23678568	11	23678568	1306
S11_23750393	11	23750393	1307
S11_23750399	11	23750399	1307
S11_23750404	11	23750404	1307
S11_23836475	11	23836475	1308
S11_23836500	11	23836500	1308
S11_23836517	11	23836517	1308
S11_23852313	11	23852313	1308
S11_23862058	11	23862058	1308
S11_23970245	11	23970245	1309
S11_23970247	11	23970247	1309
S11_23970248	11	23970248	1310
S11_23970250	11	23970250	1310
S11_23998920	11	23998920	1311
S11_23998921	11	23998921	1311
S11_24049264	11	24049264	1312
S11_24049328	11	24049328	1312
S11_24049400	11	24049400	1312
S11_24049466	11	24049466	1312
S11_24049496	11	24049496	1312
S11_24129399	11	24129399	1313
S11_24129447	11	24129447	1313
S11_24173641	11	24173641	1313
S11_24175317	11	24175317	1313
S11_24175392	11	24175392	1313
S11_24175398	11	24175398	1313
S11_24199837	11	24199837	1314

Markers	Chromosome	Position	Block #
S11_24199890	11	24199890	1314
S11_24199976	11	24199976	1314
S11_24369991	11	24369991	1315
S11_24374053	11	24374053	1315
S11_24672995	11	24672995	1316
S11_24673133	11	24673133	1316
S11_24673141	11	24673141	1316
S11_24832788	11	24832788	1317
S11_24832812	11	24832812	1317
S11_25059160	11	25059160	1318
S11_25059188	11	25059188	1318
S11_25426440	11	25426440	1319
S11_25433091	11	25433091	1319
S11_25433136	11	25433136	1319
S11_25480121	11	25480121	1320
S11_25480310	11	25480310	1320
S11_25480440	11	25480440	1321
S11_25492624	11	25492624	1321
S11_25515248	11	25515248	1321
S11_25515292	11	25515292	1321
S11_25842807	11	25842807	1322
S11_25842827	11	25842827	1322
S11_25844053	11	25844053	1322
S11_25844079	11	25844079	1322
S11_25844140	11	25844140	1322
S11_25844569	11	25844569	1322
S11_26104881	11	26104881	1323
S11_26106963	11	26106963	1323
S11_26153927	11	26153927	1324
S11_26155338	11	26155338	1324
S11_26155455	11	26155455	1324
S11_26234271	11	26234271	1324
S11_26244265	11	26244265	1324
S11_26278941	11	26278941	1325
S11_26279021	11	26279021	1325
S11_26326127	11	26326127	1325
S11_26539994	11	26539994	1326
S11_26539995	11	26539995	1326
S11_26540048	11	26540048	1327
S11_26559272	11	26559272	1327
S11_26650033	11	26650033	1328
S11_26650241	11	26650241	1328
S11_26650242	11	26650242	1328
S11_26650270	11	26650270	1328
S11_26683930	11	26683930	1329
S11_26683955	11	26683955	1329

Markers	Chromosome	Position	Block #
S11_26736573	11	26736573	1330
S11_26736591	11	26736591	1330
S11_26749863	11	26749863	1330
S11_26749931	11	26749931	1331
S11_26749932	11	26749932	1331
S11_26749933	11	26749933	1331
S11_26923049	11	26923049	1332
S11_26950648	11	26950648	1332
S11_27111786	11	27111786	1333
S11_27111818	11	27111818	1333
S11_27153355	11	27153355	1333
S11_27183484	11	27183484	1334
S11_27208416	11	27208416	1334
S11_27208433	11	27208433	1334
S11_27209349	11	27209349	1334
S11_27227367	11	27227367	1334
S11_27270494	11	27270494	1335
S11_27270631	11	27270631	1335
S11_27451178	11	27451178	1336
S11_27459944	11	27459944	1336
S11_27460078	11	27460078	1337
S11_27460080	11	27460080	1337
S11_27484864	11	27484864	1338
S11_27496446	11	27496446	1338
S11_27496755	11	27496755	1338
S11_27497494	11	27497494	1338
S11_27497495	11	27497495	1338
S11_27568651	11	27568651	1338
S12_22366	12	22366	1339
S12_27989	12	27989	1339
S12_27990	12	27990	1339
S12_27991	12	27991	1339
S12_27992	12	27992	1339
S12_68313	12	68313	1339
S12_218728	12	218728	1340
S12_248500	12	248500	1340
S12_261976	12	261976	1341
S12_261977	12	261977	1341
S12_284684	12	284684	1342
S12_369786	12	369786	1342
S12_401616	12	401616	1343
S12_401701	12	401701	1343
S12_471725	12	471725	1344
S12_472751	12	472751	1344
S12_472781	12	472781	1344
S12_749232	12	749232	1345

Markers	Chromosome	Position	Block #
S12_749337	12	749337	1345
S12_1053374	12	1053374	1346
S12_1055849	12	1055849	1346
S12_1084320	12	1084320	1347
S12_1084342	12	1084342	1347
S12_1137513	12	1137513	1348
S12_1137514	12	1137514	1348
S12_1138746	12	1138746	1348
S12_1145296	12	1145296	1348
S12_1214330	12	1214330	1349
S12_1214331	12	1214331	1349
S12_1214333	12	1214333	1349
S12_1214410	12	1214410	1350
S12_1215392	12	1215392	1350
S12_1270858	12	1270858	1351
S12_1270875	12	1270875	1351
S12_2213598	12	2213598	1352
S12_2213765	12	2213765	1352
S12_2308592	12	2308592	1352
S12_3281751	12	3281751	1353
S12_3284633	12	3284633	1353
S12_3707100	12	3707100	1354
S12_3707106	12	3707106	1354
S12_3860446	12	3860446	1355
S12_3882021	12	3882021	1355
S12_3885644	12	3885644	1355
S12_4225543	12	4225543	1356
S12_4225559	12	4225559	1356
S12_5184139	12	5184139	1357
S12_5184175	12	5184175	1357
S12_5184191	12	5184191	1357
S12_7274009	12	7274009	1358
S12_7274060	12	7274060	1358
S12_7296867	12	7296867	1358
S12_7902983	12	7902983	1359
S12_7933453	12	7933453	1359
S12_8604900	12	8604900	1360
S12_8604901	12	8604901	1360
S12_8604902	12	8604902	1360
S12_8854756	12	8854756	1361
S12_8936932	12	8936932	1361
S12_8937088	12	8937088	1361
S12_9312774	12	9312774	1362
S12_9312877	12	9312877	1362
S12_9312912	12	9312912	1362
S12_9786958	12	9786958	1363

Markers	Chromosome	Position	Block #
S12_9911841	12	9911841	1363
S12_9915377	12	9915377	1363
S12_10014043	12	10014043	1364
S12_10014107	12	10014107	1364
S12_10014138	12	10014138	1364
S12_10130882	12	10130882	1365
S12_10131421	12	10131421	1365
S12_11957639	12	11957639	1366
S12_11957641	12	11957641	1366
S12_11957682	12	11957682	1366
S12_11957685	12	11957685	1366
S12_12375085	12	12375085	1367
S12_12375225	12	12375225	1367
S12_12587679	12	12587679	1368
S12_12729237	12	12729237	1368
S12_13740569	12	13740569	1369
S12_13740610	12	13740610	1369
S12_13770094	12	13770094	1370
S12_13770110	12	13770110	1370
S12_13770121	12	13770121	1370
S12_13770135	12	13770135	1370
S12_14676203	12	14676203	1371
S12_14676204	12	14676204	1371
S12_14676275	12	14676275	1371
S12_14807434	12	14807434	1372
S12_14807435	12	14807435	1372
S12_14873347	12	14873347	1373
S12_14903006	12	14903006	1373
S12_14943935	12	14943935	1374
S12_14955004	12	14955004	1374
S12_15860794	12	15860794	1375
S12_15860952	12	15860952	1375
S12_16684521	12	16684521	1376
S12_16691210	12	16691210	1376
S12_16695188	12	16695188	1376
S12_16696789	12	16696789	1376
S12_16696897	12	16696897	1376
S12_16696916	12	16696916	1376
S12_16697103	12	16697103	1376
S12_17518555	12	17518555	1377
S12_17525402	12	17525402	1377
S12_17525446	12	17525446	1377
S12_17738701	12	17738701	1378
S12_17738703	12	17738703	1378
S12_17739231	12	17739231	1378
S12_17810992	12	17810992	1379

Markers	Chromosome	Position	Block #
S12_17811037	12	17811037	1379
S12_17819931	12	17819931	1379
S12_17902031	12	17902031	1380
S12_18048906	12	18048906	1380
S12_18231734	12	18231734	1381
S12_18318554	12	18318554	1381
S12_18457851	12	18457851	1382
S12_18461450	12	18461450	1382
S12_18713839	12	18713839	1383
S12_18714071	12	18714071	1383
S12_18714094	12	18714094	1383
S12_18714107	12	18714107	1383
S12_18728808	12	18728808	1383
S12_18728841	12	18728841	1383
S12_19240071	12	19240071	1384
S12_19240136	12	19240136	1384
S12_19250140	12	19250140	1384
S12_19340454	12	19340454	1385
S12_19340479	12	19340479	1385
S12_19341211	12	19341211	1386
S12_19341241	12	19341241	1386
S12_19345566	12	19345566	1386
S12_19345634	12	19345634	1386
S12_19345644	12	19345644	1386
S12_19466940	12	19466940	1387
S12_19471218	12	19471218	1387
S12_19649015	12	19649015	1388
S12_19649233	12	19649233	1388
S12_19671851	12	19671851	1389
S12_19671939	12	19671939	1389
S12_19688946	12	19688946	1389
S12_19698806	12	19698806	1389
S12_19700012	12	19700012	1389
S12_19700995	12	19700995	1389
S12_19701148	12	19701148	1389
S12_19701204	12	19701204	1389
S12_19820777	12	19820777	1389
S12_20135324	12	20135324	1390
S12_20135373	12	20135373	1390
S12_20135387	12	20135387	1390
S12_20135399	12	20135399	1390
S12_20247645	12	20247645	1390
S12_20637068	12	20637068	1391
S12_20637255	12	20637255	1391
S12_20648279	12	20648279	1392
S12_20648300	12	20648300	1392

Markers	Chromosome	Position	Block #
S12_20648354	12	20648354	1392
S12_21310417	12	21310417	1393
S12_21310420	12	21310420	1393
S12_21310426	12	21310426	1393
S12_21357709	12	21357709	1394
S12_21357729	12	21357729	1394
S12_21357749	12	21357749	1394
S12_21403499	12	21403499	1395
S12_21403595	12	21403595	1395
S12_21403643	12	21403643	1395
S12_21457268	12	21457268	1396
S12_21457362	12	21457362	1396
S12_21467627	12	21467627	1397
S12_21467637	12	21467637	1397
S12_21467646	12	21467646	1397
S12_21511082	12	21511082	1398
S12_21511310	12	21511310	1398
S12_21514104	12	21514104	1399
S12_21514130	12	21514130	1399
S12_21592658	12	21592658	1400
S12_21592667	12	21592667	1400
S12_21606754	12	21606754	1401
S12_21606755	12	21606755	1401
S12_21737332	12	21737332	1402
S12_21737388	12	21737388	1402
S12_21739295	12	21739295	1402
S12_21739309	12	21739309	1402
S12_21808935	12	21808935	1403
S12_21809056	12	21809056	1403
S12_21902217	12	21902217	1404
S12_21902235	12	21902235	1404
S12_21998026	12	21998026	1405
S12_21998032	12	21998032	1405
S12_22052186	12	22052186	1406
S12_22052358	12	22052358	1406
S12_22072570	12	22072570	1407
S12_22072572	12	22072572	1407
S12_22089317	12	22089317	1408
S12_22089329	12	22089329	1408
S12_22098752	12	22098752	1409
S12_22102287	12	22102287	1409
S12_22355333	12	22355333	1410
S12_22355350	12	22355350	1410
S12_22513871	12	22513871	1411
S12_22514019	12	22514019	1411
S12_22574877	12	22574877	1412

Markers	Chromosome	Position	Block #
S12_22574901	12	22574901	1412
S12_22581778	12	22581778	1413
S12_22581779	12	22581779	1413
S12_22581832	12	22581832	1413
S12_22582039	12	22582039	1414
S12_22582043	12	22582043	1414
S12_22582052	12	22582052	1414
S12_22583853	12	22583853	1414
S12_22583880	12	22583880	1414
S12_22762229	12	22762229	1415
S12_22766143	12	22766143	1415
S12_22840504	12	22840504	1416
S12_22854722	12	22854722	1416
S12_22910242	12	22910242	1417
S12_22910245	12	22910245	1417
S12_22910290	12	22910290	1417
S12_22999506	12	22999506	1418
S12_22999629	12	22999629	1418
S12_23000863	12	23000863	1418
S12_23000889	12	23000889	1418
S12_23001518	12	23001518	1418
S12_23001542	12	23001542	1418
S12_23001572	12	23001572	1418
S12_23001609	12	23001609	1418
S12_23064931	12	23064931	1419
S12_23067689	12	23067689	1419
S12_23082938	12	23082938	1419
S12_23175105	12	23175105	1420
S12_23175229	12	23175229	1420
S12_23223123	12	23223123	1421
S12_23223127	12	23223127	1421
S12_23397338	12	23397338	1422
S12_23397344	12	23397344	1422
S12_23405131	12	23405131	1422
S12_23405176	12	23405176	1422
S12_23665674	12	23665674	1423
S12_23665678	12	23665678	1423
S12_23665685	12	23665685	1423
S12_23723877	12	23723877	1424
S12_23723880	12	23723880	1424
S12_23723985	12	23723985	1424
S12_23747591	12	23747591	1425
S12_23762640	12	23762640	1425
S12_23828003	12	23828003	1426
S12_23828004	12	23828004	1426
S12_23963194	12	23963194	1427

Markers	Chromosome	Position	Block #
S12_23963195	12	23963195	1427
S12_23994280	12	23994280	1428
S12_23995911	12	23995911	1428
S12_23995935	12	23995935	1428
S12_24018837	12	24018837	1429
S12_24018889	12	24018889	1429
S12_24220712	12	24220712	1430
S12_24220759	12	24220759	1430
S12_24220760	12	24220760	1430
S12_24233839	12	24233839	1431
S12_24238335	12	24238335	1431
S12_24238467	12	24238467	1431
S12_24282982	12	24282982	1432
S12_24283083	12	24283083	1432
S12_24283198	12	24283198	1432
S12_24285959	12	24285959	1432
S12_24285962	12	24285962	1432
S12_24286071	12	24286071	1432
S12_24289561	12	24289561	1432
S12_24321830	12	24321830	1433
S12_24321835	12	24321835	1433
S12_24400771	12	24400771	1434
S12_24407742	12	24407742	1434
S12_24411649	12	24411649	1434
S12_24760513	12	24760513	1435
S12_24763895	12	24763895	1435
S12_24786330	12	24786330	1436
S12_24786531	12	24786531	1436
S12_24788462	12	24788462	1436
