# Supplementary material for: Genome-Wide Differentiation of Various Melon Horticultural Groups for Use in GWAS for Fruit Firmness and Construction of a High Resolution Genetic Map
Source: Front Plant Sci. 2016 Sep 22;7:1437. doi: 10.3389/fpls.2016.01437 (PMC5031849; doi:10.3389/fpls.2016.01437)
Supplement: Table S6 — Linkage disequilibrium (LD) analysis for adjacent SNP pairs across melon genome. [file Table6.pdf]

Table S6: Linkage disequilibrium (LD) analysis for adjacent SNP pairs across melon genome.

Marker 1	Chr.	Position	Marker 2	Distance in markers	Distance in kb	EM - R Squared	EM - D Prime	CHM - R Squared	CHM - D Prime	CHM - signed D
S1_1978816	1	1978816	S1_1978820	1	0.004	1.0000	1.0000	1.0000	1.0000	0.1015
S1_2866450	1	2866450	S1_2868703	1	2.253	1.0000	1.0000	1.0000	1.0000	0.1106
S1_5910878	1	5910878	S1_5910882	1	0.004	1.0000	1.0000	1.0000	1.0000	0.0909
S1_9917532	1	9917532	S1_9917565	1	0.033	1.0000	1.0000	1.0000	1.0000	0.2498
S1_10473985	1	10473985	S1_10473987	1	0.002	1.0000	1.0000	1.0000	1.0000	0.0732
S1_13316490	1	13316490	S1_13316491	1	0.001	1.0000	1.0000	1.0000	1.0000	0.2148
S1_19629435	1	19629435	S1_19629537	1	0.102	1.0000	1.0000	1.0000	1.0000	0.1401
S1_19877622	1	19877622	S1_19877652	1	0.03	1.0000	1.0000	1.0000	1.0000	0.0942
S1_19877652	1	19877652	S1_19887160	1	9.508	1.0000	1.0000	1.0000	1.0000	0.0997
S1_20753018	1	20753018	S1_20753240	2	0.222	1.0000	1.0000	1.0000	1.0000	0.1224
S1_28861268	1	28861268	S1_29012469	1	151.201	1.0000	1.0000	1.0000	1.0000	0.1044
S1_30427867	1	30427867	S1_30427876	1	0.009	1.0000	1.0000	1.0000	1.0000	0.1053
S1_34311605	1	34311605	S1_34311734	1	0.129	1.0000	1.0000	1.0000	1.0000	0.1134
S1_34311810	1	34311810	S1_34311811	1	0.001	1.0000	1.0000	1.0000	1.0000	0.1389
S1_11934548	1	11934548	S1_11934629	1	0.081	1.0000	1.0000	1.0000	1.0000	0.0764
S1_16448884	1	16448884	S1_16448908	1	0.024	1.0000	1.0000	1.0000	1.0000	0.1321
S1_20324887	1	20324887	S1_20324890	1	0.003	1.0000	1.0000	1.0000	1.0000	0.0779
S1_20324890	1	20324890	S1_20324893	1	0.003	1.0000	1.0000	1.0000	1.0000	0.0779
S1_20324893	1	20324893	S1_20324894	1	0.001	1.0000	1.0000	1.0000	1.0000	0.0779
S1_20324894	1	20324894	S1_20324895	1	0.001	1.0000	1.0000	1.0000	1.0000	0.0779
S1_22695291	1	22695291	S1_22695314	1	0.023	1.0000	1.0000	1.0000	1.0000	0.0858
S1_32737027	1	32737027	S1_32737051	1	0.024	1.0000	1.0000	1.0000	1.0000	0.1629
S1_11642270	1	11642270	S1_11651112	1	8.842	1.0000	1.0000	1.0000	1.0000	0.1365
S1_30942620	1	30942620	S1_30942635	1	0.015	1.0000	1.0000	1.0000	1.0000	0.0671
S1_32561673	1	32561673	S1_32561726	1	0.053	1.0000	1.0000	1.0000	1.0000	0.1005

S1_3039283	1	3039283	S1_3039301	1	0.018	1.0000	1.0000	1.0000	1.0000	0.2500
S1_2389920	1	2389920	S1_2389962	1	0.042	1.0000	1.0000	1.0000	1.0000	0.2491
S1_2065378	1	2065378	S1_2065379	1	0.001	1.0000	1.0000	1.0000	1.0000	0.1340
S1_2065379	1	2065379	S1_2065380	1	0.001	1.0000	1.0000	1.0000	1.0000	0.1340
S1_12382636	1	12382636	S1_12382669	1	0.033	1.0000	1.0000	1.0000	1.0000	0.1906
S1_16180513	1	16180513	S1_16182249	1	1.736	1.0000	1.0000	1.0000	1.0000	0.1282
S1_4029973	1	4029973	S1_4029989	1	0.016	1.0000	1.0000	1.0000	1.0000	0.1198
S1_6373106	1	6373106	S1_6373107	1	0.001	1.0000	1.0000	1.0000	1.0000	0.1219
S1_30428799	1	30428799	S1_30428823	1	0.024	1.0000	1.0000	1.0000	1.0000	0.1123
S1_30428823	1	30428823	S1_30428853	1	0.03	1.0000	1.0000	1.0000	1.0000	0.1123
S1_30428853	1	30428853	S1_30428977	1	0.124	1.0000	1.0000	1.0000	1.0000	0.1123
S1_30428977	1	30428977	S1_30428983	1	0.006	1.0000	1.0000	1.0000	1.0000	0.1123
S1_30428983	1	30428983	S1_30429008	1	0.025	1.0000	1.0000	1.0000	1.0000	0.1123
S1_30429008	1	30429008	S1_30432198	1	3.19	1.0000	1.0000	1.0000	1.0000	0.1123
S1_30432198	1	30432198	S1_30432212	1	0.014	1.0000	1.0000	1.0000	1.0000	0.1123
S1_17599370	1	17599370	S1_17599371	1	0.001	1.0000	1.0000	1.0000	1.0000	0.1288
S1_17599369	1	17599369	S1_17599370	1	0.001	1.0000	1.0000	1.0000	1.0000	0.1288
S1_30432212	1	30432212	S1_30432247	1	0.035	1.0000	1.0000	1.0000	1.0000	0.1163
S1_1474681	1	1474681	S1_1474748	1	0.067	1.0000	1.0000	1.0000	1.0000	0.1045
S1_32392522	1	32392522	S1_32392554	1	0.032	1.0000	1.0000	1.0000	1.0000	0.1045
S1_30427528	1	30427528	S1_30427573	1	0.045	1.0000	1.0000	1.0000	1.0000	0.1084
S1_30427675	1	30427675	S1_30427677	1	0.002	1.0000	1.0000	1.0000	1.0000	0.1104
S1_25955609	1	25955609	S1_25962057	2	6.448	1.0000	1.0000	1.0000	1.0000	0.1125
S1_25962057	1	25962057	S1_25962058	1	0.001	1.0000	1.0000	1.0000	1.0000	0.1125
S1_15986136	1	15986136	S1_15986209	1	0.073	1.0000	1.0000	1.0000	1.0000	0.1642
S1_30427573	1	30427573	S1_30427675	1	0.102	1.0000	1.0000	1.0000	1.0000	0.1051
S1_7316130	1	7316130	S1_7316131	1	0.001	1.0000	1.0000	1.0000	1.0000	0.0926
S1_30636173	1	30636173	S1_30636178	1	0.005	1.0000	1.0000	1.0000	1.0000	0.2493
S1_32484151	1	32484151	S1_32484226	1	0.075	1.0000	1.0000	1.0000	1.0000	0.0720
S1_32217015	1	32217015	S1_32217025	1	0.01	1.0000	1.0000	1.0000	1.0000	0.0772

S1_21615713	1	21615713	S1_21615727	1	0.014	1.0000	1.0000	1.0000	1.0000	0.2424
S1_13273564	1	13273564	S1_13273592	1	0.028	1.0000	1.0000	1.0000	1.0000	0.2175
S1_19433984	1	19433984	S1_19433996	1	0.012	1.0000	1.0000	1.0000	1.0000	0.2456
S1_20419824	1	20419824	S1_20438230	2	18.406	1.0000	1.0000	1.0000	1.0000	0.0822
S1_32633756	1	32633756	S1_32633761	1	0.005	1.0000	1.0000	1.0000	1.0000	0.2031
S1_32633761	1	32633761	S1_32633768	1	0.007	1.0000	1.0000	1.0000	1.0000	0.2031
S1_32633744	1	32633744	S1_32633756	1	0.012	1.0000	1.0000	1.0000	1.0000	0.2031
S1_32070504	1	32070504	S1_32070527	1	0.023	1.0000	1.0000	1.0000	1.0000	0.1715
S1_32070527	1	32070527	S1_32070533	1	0.006	1.0000	1.0000	1.0000	1.0000	0.1715
S1_1088695	1	1088695	S1_1088697	1	0.002	1.0000	1.0000	1.0000	1.0000	0.2483
S1_1088697	1	1088697	S1_1088706	1	0.009	1.0000	1.0000	1.0000	1.0000	0.2483
S1_33602724	1	33602724	S1_33605389	1	2.665	1.0000	1.0000	1.0000	1.0000	0.2432
S1_31736671	1	31736671	S1_31736707	2	0.036	1.0000	1.0000	1.0000	1.0000	0.2394
S1_3248561	1	3248561	S1_3248562	1	0.001	1.0000	1.0000	1.0000	1.0000	0.0717
S1_1821158	1	1821158	S1_1821191	1	0.033	1.0000	1.0000	1.0000	1.0000	0.2129
S1_2066702	1	2066702	S1_2077108	1	10.406	1.0000	1.0000	1.0000	1.0000	0.1150
S1_31616406	1	31616406	S1_31616430	1	0.024	1.0000	1.0000	1.0000	1.0000	0.2500
S1_32323545	1	32323545	S1_32323548	1	0.003	1.0000	1.0000	1.0000	1.0000	0.2498
S1_2065431	1	2065431	S1_2065432	1	0.001	1.0000	1.0000	1.0000	1.0000	0.0637
S1_2065432	1	2065432	S1_2065433	1	0.001	1.0000	1.0000	1.0000	1.0000	0.0637
S1_2065433	1	2065433	S1_2065434	1	0.001	1.0000	1.0000	1.0000	1.0000	0.0637
S1_27938304	1	27938304	S1_27938305	1	0.001	1.0000	1.0000	1.0000	1.0000	0.2353
S1_34051307	1	34051307	S1_34051326	1	0.019	1.0000	1.0000	1.0000	1.0000	0.2175
S1_21980717	1	21980717	S1_21980740	1	0.023	1.0000	1.0000	1.0000	1.0000	0.1664
S1_31652001	1	31652001	S1_31652002	1	0.001	1.0000	1.0000	1.0000	1.0000	0.1123
S1_33602641	1	33602641	S1_33602645	1	0.004	1.0000	1.0000	1.0000	1.0000	0.2414
S1_33552004	1	33552004	S1_33552005	1	0.001	1.0000	1.0000	1.0000	1.0000	0.2371
S1_27951804	1	27951804	S1_27951805	1	0.001	1.0000	1.0000	1.0000	1.0000	0.0540
S1_27951805	1	27951805	S1_27951808	1	0.003	1.0000	1.0000	1.0000	1.0000	0.0540
S1_27951842	1	27951842	S1_28034804	1	82.962	1.0000	1.0000	1.0000	1.0000	0.2294

S1_34851217	1	34851217	S1_34859222	2	8.005	1.0000	1.0000	1.0000	1.0000	0.0935
S1_31843785	1	31843785	S1_31843786	1	0.001	1.0000	1.0000	1.0000	1.0000	0.2082
S1_31179299	1	31179299	S1_31179302	1	0.003	1.0000	1.0000	1.0000	1.0000	0.2494
S1_32186259	1	32186259	S1_32186278	1	0.019	1.0000	1.0000	1.0000	1.0000	0.0800
S1_32118738	1	32118738	S1_32118739	1	0.001	1.0000	1.0000	1.0000	1.0000	0.1449
S1_32118763	1	32118763	S1_32118765	1	0.002	1.0000	1.0000	1.0000	1.0000	0.1449
S1_30909295	1	30909295	S1_30909296	1	0.001	1.0000	1.0000	1.0000	1.0000	0.2491
S1_445936	1	445936	S1_445937	1	0.001	1.0000	1.0000	1.0000	1.0000	0.0616
S1_445937	1	445937	S1_445938	1	0.001	1.0000	1.0000	1.0000	1.0000	0.0616
S1_15789964	1	15789964	S1_15790004	1	0.04	1.0000	1.0000	1.0000	1.0000	0.2472
S1_31148968	1	31148968	S1_31148978	1	0.01	1.0000	1.0000	1.0000	1.0000	0.2500
S1_31148978	1	31148978	S1_31149021	1	0.043	1.0000	1.0000	1.0000	1.0000	0.2500
S1_22620761	1	22620761	S1_22620762	1	0.001	1.0000	1.0000	1.0000	1.0000	0.2483
S1_22620762	1	22620762	S1_22620763	1	0.001	1.0000	1.0000	1.0000	1.0000	0.2483
S1_20128996	1	20128996	S1_20129013	1	0.017	1.0000	1.0000	1.0000	1.0000	0.1145
S1_28625558	1	28625558	S1_28625559	1	0.001	1.0000	1.0000	1.0000	1.0000	0.2121
S1_28625559	1	28625559	S1_28625560	1	0.001	1.0000	1.0000	1.0000	1.0000	0.2121
S1_10769356	1	10769356	S1_10769368	1	0.012	1.0000	1.0000	1.0000	1.0000	0.1343
S1_12490090	1	12490090	S1_12490091	1	0.001	1.0000	1.0000	1.0000	1.0000	0.2493
S1_12490091	1	12490091	S1_12490101	1	0.01	1.0000	1.0000	1.0000	1.0000	0.2493
S1_16638978	1	16638978	S1_16639010	1	0.032	1.0000	1.0000	1.0000	1.0000	0.2423
S1_16639010	1	16639010	S1_16639013	1	0.003	1.0000	1.0000	1.0000	1.0000	0.2423
S1_27613340	1	27613340	S1_27613343	1	0.003	1.0000	1.0000	1.0000	1.0000	0.1938
S1_25191928	1	25191928	S1_25191930	1	0.002	1.0000	1.0000	1.0000	1.0000	0.2359
S1_25191930	1	25191930	S1_25191933	1	0.003	1.0000	1.0000	1.0000	1.0000	0.2359
S1_7316126	1	7316126	S1_7316128	1	0.002	1.0000	1.0000	1.0000	1.0000	0.1094
S1_7316128	1	7316128	S1_7316129	1	0.001	1.0000	1.0000	1.0000	1.0000	0.1094
S1_29467858	1	29467858	S1_29467885	1	0.027	0.9791	1.0000	0.9894	1.0000	0.2478
S1_34567655	1	34567655	S1_34569144	2	1.489	0.9787	1.0000	0.9888	1.0000	0.2403
S1_33551958	1	33551958	S1_33552004	1	0.046	0.9785	1.0000	0.9888	1.0000	0.2364

S1_13316491	1	13316491	S1_13316617	1	0.126	0.9760	1.0000	0.9880	1.0000	0.2157
S1_20996968	1	20996968	S1_21002359	4	5.391	0.9751	1.0000	0.9872	1.0000	0.2350
S1_1959150	1	1959150	S1_1959340	1	0.19	0.9693	1.0000	0.9841	1.0000	0.1831
S1_5050434	1	5050434	S1_5059593	1	9.159	0.9663	1.0000	0.9830	1.0000	0.1589
S1_15984481	1	15984481	S1_15986136	1	1.655	0.9655	1.0000	0.9829	1.0000	0.1810
S1_4201369	1	4201369	S1_4208168	1	6.799	0.9637	1.0000	0.9818	1.0000	0.1382
S1_6319033	1	6319033	S1_6319292	2	0.259	0.9636	1.0000	0.9817	1.0000	0.1405
S1_16164203	1	16164203	S1_16180513	1	16.31	0.9618	1.0000	0.9808	1.0000	0.1312
S1_29466386	1	29466386	S1_29467858	1	1.472	0.9590	1.0000	0.9794	1.0000	0.2464
S1_34309486	1	34309486	S1_34311423	1	1.937	0.9582	1.0000	0.9788	1.0000	0.1448
S1_21021981	1	21021981	S1_21022029	1	0.048	0.9580	1.0000	0.9782	1.0000	0.2377
S1_19344198	1	19344198	S1_19344240	1	0.042	0.9579	0.9787	0.9773	0.9886	0.2396
S1_13679723	1	13679723	S1_13679730	1	0.007	0.9578	1.0000	0.9539	0.9979	0.2338
S1_8858478	1	8858478	S1_8999772	1	141.294	0.9569	1.0000	0.9782	1.0000	0.1603
S1_25955137	1	25955137	S1_25955609	1	0.472	0.9569	1.0000	0.9781	1.0000	0.1219
S1_33552049	1	33552049	S1_33552077	1	0.028	0.9563	1.0000	0.9537	0.9987	0.2298
S1_27938305	1	27938305	S1_27945039	1	6.734	0.9560	1.0000	0.9771	1.0000	0.2313
S1_16638975	1	16638975	S1_16638978	1	0.003	0.9555	1.0000	0.9433	0.9936	0.2343
S1_33605389	1	33605389	S1_33605727	2	0.338	0.9546	1.0000	0.9767	1.0000	0.2390
S1_13273592	1	13273592	S1_13316490	1	42.898	0.9531	1.0000	0.9763	1.0000	0.2142
S1_20332951	1	20332951	S1_20333007	1	0.056	0.9527	1.0000	0.9753	1.0000	0.1061
S1_32443379	1	32443379	S1_32443670	1	0.291	0.9526	0.9760	0.9756	0.9877	0.2342
S1_8748654	1	8748654	S1_8748726	1	0.072	0.9520	1.0000	0.9745	1.0000	0.2113
S1_30427876	1	30427876	S1_30428799	1	0.923	0.9506	1.0000	0.9748	1.0000	0.1059
S1_30427677	1	30427677	S1_30427867	1	0.19	0.9463	1.0000	0.9726	1.0000	0.1015
S1_12393771	1	12393771	S1_12397960	1	4.189	0.9446	1.0000	0.9714	1.0000	0.0891
S1_22695314	1	22695314	S1_22695339	1	0.025	0.9419	1.0000	0.9702	1.0000	0.0865
S1_5910882	1	5910882	S1_5922191	1	11.309	0.9410	1.0000	0.9698	1.0000	0.0975
S1_9229580	1	9229580	S1_9229624	1	0.044	0.9398	1.0000	0.9426	1.0000	0.2419
S1_22695261	1	22695261	S1_22695291	1	0.03	0.9390	1.0000	0.9703	1.0000	0.0823

S1_20101090	1	20101090	S1_20101123	2	0.033	0.9387	1.0000	0.9675	1.0000	0.0855
S1_5885779	1	5885779	S1_5910878	1	25.099	0.9386	1.0000	0.9701	1.0000	0.0873
S1_17061963	1	17061963	S1_17061967	1	0.004	0.9352	1.0000	0.9342	0.9994	0.1827
S1_2868703	1	2868703	S1_2872204	1	3.501	0.9348	1.0000	0.9665	1.0000	0.0889
S1_33552077	1	33552077	S1_33552138	1	0.061	0.9332	0.9770	0.9412	0.9812	0.2274
S1_2277582	1	2277582	S1_2279144	1	1.562	0.9310	1.0000	0.9639	1.0000	0.1430
S1_34999530	1	34999530	S1_34999589	1	0.059	0.9308	0.9648	0.9645	0.9821	0.2286
S1_34990762	1	34990762	S1_34990793	1	0.031	0.9280	1.0000	0.9640	1.0000	0.0692
S1_25962058	1	25962058	S1_25962371	1	0.313	0.9274	1.0000	0.9625	1.0000	0.0765
S1_27113206	1	27113206	S1_27134578	1	21.372	0.9241	1.0000	0.9603	1.0000	0.1280
S1_27560257	1	27560257	S1_27613340	1	53.083	0.9224	0.9732	0.9586	0.9921	0.1909
S1_17051411	1	17051411	S1_17061788	1	10.377	0.9220	0.9730	0.9589	0.9923	0.2093
S1_10186966	1	10186966	S1_10186973	1	0.007	0.9217	1.0000	0.9294	1.0000	0.1906
S1_12672535	1	12672535	S1_12783334	1	110.799	0.9216	1.0000	0.9184	0.9982	0.1586
S1_10763657	1	10763657	S1_10763668	1	0.011	0.9208	1.0000	0.9596	1.0000	0.2446
S1_4327126	1	4327126	S1_4327200	1	0.074	0.9184	0.9583	0.9579	0.9787	0.2446
S1_2866270	1	2866270	S1_2866432	1	0.162	0.9165	0.9711	0.9558	0.9917	0.2417
S1_35156294	1	35156294	S1_35156452	1	0.158	0.9165	0.9711	0.9564	0.9920	0.2382
S1_27945039	1	27945039	S1_27945136	1	0.097	0.9145	1.0000	0.9315	1.0000	0.2225
S1_25191933	1	25191933	S1_25192008	1	0.075	0.9144	0.9562	0.9527	0.9761	0.2303
S1_25570198	1	25570198	S1_25571483	2	1.285	0.9135	1.0000	0.9543	1.0000	0.1241
S1_4037334	1	4037334	S1_4037474	1	0.14	0.9118	0.9549	0.9549	0.9772	0.1230
S1_11297264	1	11297264	S1_11331929	2	34.665	0.9115	1.0000	0.9115	1.0000	0.1120
S1_33435266	1	33435266	S1_33437852	2	2.586	0.9095	0.9537	0.9520	0.9757	0.2342
S1_19426454	1	19426454	S1_19433819	2	7.365	0.9082	0.9758	0.9527	0.9994	0.2400
S1_20129013	1	20129013	S1_20129057	1	0.044	0.9054	0.9515	0.9491	0.9742	0.1170
S1_967688	1	967688	S1_967706	1	0.018	0.9053	0.9670	0.9136	0.9714	0.1520
S1_20744988	1	20744988	S1_20753018	1	8.03	0.9041	1.0000	0.9041	1.0000	0.1122
S1_7600588	1	7600588	S1_7616769	2	16.181	0.9040	1.0000	0.9498	1.0000	0.0513
S1_12490144	1	12490144	S1_12490251	1	0.107	0.9026	0.9660	0.9495	0.9908	0.1568

S1_26348807	1	26348807	S1_26410718	1	61.911	0.9021	0.9498	0.9492	0.9743	0.1129
S1_2629086	1	2629086	S1_2629173	1	0.087	0.9001	0.9487	0.9487	0.9740	0.0979
S1_33591110	1	33591110	S1_33602641	1	11.531	0.8959	0.9567	0.9240	0.9716	0.2316
S1_10072577	1	10072577	S1_10154387	1	81.81	0.8951	0.9564	0.9424	0.9813	0.2397
S1_33437852	1	33437852	S1_33442450	4	4.598	0.8942	0.9773	0.9436	1.0000	0.2353
S1_29867805	1	29867805	S1_29874017	1	6.212	0.8897	0.9540	0.9405	0.9809	0.2273
S1_22277483	1	22277483	S1_22298582	2	21.099	0.8897	1.0000	0.8897	1.0000	0.0840
S1_410695	1	410695	S1_422883	2	12.188	0.8893	1.0000	0.9160	1.0000	0.1753
S1_22635905	1	22635905	S1_22669192	1	33.287	0.8889	1.0000	0.8889	1.0000	0.0889
S1_21976007	1	21976007	S1_21980717	1	4.71	0.8874	0.9605	0.9011	0.9678	0.1551
S1_22987949	1	22987949	S1_22988151	1	0.202	0.8846	1.0000	0.8812	0.9980	0.0789
S1_13237927	1	13237927	S1_13273564	1	35.637	0.8845	0.9750	0.9394	1.0000	0.2190
S1_32186278	1	32186278	S1_32186408	1	0.13	0.8845	1.0000	0.9440	1.0000	0.0824
S1_20101123	1	20101123	S1_20101125	2	0.002	0.8843	1.0000	0.9373	1.0000	0.0837
S1_22988151	1	22988151	S1_23116440	1	128.289	0.8822	1.0000	0.8786	0.9979	0.0934
S1_28269579	1	28269579	S1_28269602	1	0.023	0.8819	1.0000	0.8975	1.0000	0.1284
S1_29874020	1	29874020	S1_29874085	1	0.065	0.8817	0.9506	0.9382	0.9805	0.2155
S1_8748726	1	8748726	S1_8774902	1	26.176	0.8797	0.9739	0.9104	0.9907	0.1981
S1_22669192	1	22669192	S1_22669541	1	0.349	0.8779	1.0000	0.8779	1.0000	0.0808
S1_33605727	1	33605727	S1_33633943	2	28.216	0.8705	0.9456	0.9323	0.9786	0.2368
S1_5059593	1	5059593	S1_5098507	2	38.914	0.8695	1.0000	0.8672	0.9987	0.1386
S1_2841729	1	2841729	S1_2841890	1	0.161	0.8609	1.0000	0.9333	1.0000	0.1208
S1_35127217	1	35127217	S1_35156294	2	29.077	0.8583	0.9738	0.8787	0.9853	0.2265
S1_8774902	1	8774902	S1_8828772	1	53.87	0.8545	0.9730	0.8956	0.9961	0.1945
S1_13592388	1	13592388	S1_13592600	2	0.212	0.8534	1.0000	0.8496	0.9978	0.2051
S1_32133410	1	32133410	S1_32133589	2	0.179	0.8481	0.9584	0.9185	0.9974	0.1319
S1_35181663	1	35181663	S1_35229479	2	47.816	0.8460	1.0000	0.8801	1.0000	0.2311
S1_33792208	1	33792208	S1_33792292	1	0.084	0.8454	0.9301	0.8699	0.9435	0.2204
S1_6483432	1	6483432	S1_6502853	1	19.421	0.8437	0.9439	0.8903	0.9696	0.2163
S1_35156452	1	35156452	S1_35181663	1	25.211	0.8428	0.9746	0.8724	0.9915	0.2297

S1_20051010	1	20051010	S1_20101090	1	50.08	0.8415	1.0000	0.8646	1.0000	0.0887
S1_7950048	1	7950048	S1_7950165	1	0.117	0.8413	1.0000	0.8644	1.0000	0.0895
S1_12490287	1	12490287	S1_12531680	2	41.393	0.8392	0.9422	0.9119	0.9821	0.1090
S1_2841728	1	2841728	S1_2841729	1	0.001	0.8376	1.0000	0.8342	0.9979	0.1240
S1_3338743	1	3338743	S1_3346163	1	7.42	0.8361	0.9302	0.8455	0.9354	0.1474
S1_34311423	1	34311423	S1_34311605	2	0.182	0.8319	1.0000	0.8319	1.0000	0.1238
S1_11651112	1	11651112	S1_11668343	1	17.231	0.8301	1.0000	0.9017	1.0000	0.1132
S1_11938633	1	11938633	S1_11938634	1	0.001	0.8271	1.0000	0.7902	0.9775	0.1205
S1_15850510	1	15850510	S1_15850721	1	0.211	0.8261	0.9518	0.9070	0.9973	0.1308
S1_34311734	1	34311734	S1_34311810	1	0.076	0.8223	1.0000	0.8223	1.0000	0.1129
S1_29283385	1	29283385	S1_29283393	1	0.008	0.8219	1.0000	0.8219	1.0000	0.0592
S1_19844260	1	19844260	S1_19877622	3	33.362	0.8193	0.9344	0.8469	0.9500	0.0832
S1_12405774	1	12405774	S1_12405804	1	0.03	0.8188	0.9342	0.8402	0.9463	0.0846
S1_22669541	1	22669541	S1_22695261	1	25.72	0.8119	0.9314	0.8390	0.9468	0.0719
S1_34026945	1	34026945	S1_34027052	2	0.107	0.8111	1.0000	0.8080	0.9981	0.1445
S1_32736752	1	32736752	S1_32737027	1	0.275	0.8100	1.0000	0.8287	1.0000	0.1418
S1_29128331	1	29128331	S1_29205773	4	77.442	0.8079	1.0000	0.8352	1.0000	0.0746
S1_22987914	1	22987914	S1_22987949	1	0.035	0.8072	1.0000	0.8023	0.9970	0.0861
S1_28269602	1	28269602	S1_28269680	1	0.078	0.8053	0.9160	0.8954	0.9659	0.1370
S1_26531567	1	26531567	S1_26548533	2	16.966	0.8020	0.9546	0.8166	0.9632	0.1117
S1_15961435	1	15961435	S1_15984481	1	23.046	0.7936	0.9652	0.8322	0.9884	0.1776
S1_28625656	1	28625656	S1_28682987	1	57.331	0.7885	0.9398	0.8353	0.9674	0.0957
S1_19844177	1	19844177	S1_19844260	2	0.083	0.7866	1.0000	0.7806	0.9962	0.0929
S1_19629537	1	19629537	S1_19651791	2	22.254	0.7841	0.9202	0.7841	0.9202	0.1188
S1_31925260	1	31925260	S1_31928764	1	3.504	0.7831	0.9631	0.8210	0.9861	0.1610
S1_2591201	1	2591201	S1_2629086	2	37.885	0.7718	0.9464	0.7877	0.9561	0.1076
S1_34289744	1	34289744	S1_34297341	1	7.597	0.7696	1.0000	0.7696	1.0000	0.1101
S1_25746889	1	25746889	S1_25755285	3	8.396	0.7671	1.0000	0.8229	1.0000	0.0875
S1_26410718	1	26410718	S1_26411546	2	0.828	0.7636	0.8962	0.7837	0.9080	0.1182
S1_14508867	1	14508867	S1_14508874	1	0.007	0.7616	1.0000	0.7671	1.0000	0.1290

S1_29205773	1	29205773	S1_29215710	1	9.937	0.7583	0.9094	0.7914	0.9290	0.0633
S1_35123513	1	35123513	S1_35127217	1	3.704	0.7518	1.0000	0.7925	1.0000	0.2174
S1_20101180	1	20101180	S1_20128996	2	27.816	0.7241	0.8946	0.8000	0.9403	0.1015
S1_3181368	1	3181368	S1_3238807	1	57.439	0.7228	0.9559	0.7476	0.9721	0.1227
S1_4177848	1	4177848	S1_4201369	2	23.521	0.7207	0.9168	0.7159	0.9137	0.1134
S1_33694591	1	33694591	S1_33694662	1	0.071	0.7159	1.0000	0.7847	1.0000	0.0694
S1_20333007	1	20333007	S1_20419824	2	86.817	0.7116	1.0000	0.7042	0.9948	0.0827
S1_20324895	1	20324895	S1_20332951	1	8.056	0.7019	1.0000	0.7400	1.0000	0.0772
S1_10958959	1	10958959	S1_10967208	3	8.249	0.6956	0.8818	0.8172	0.9558	0.0471
S1_20753428	1	20753428	S1_20815715	2	62.287	0.6889	0.9422	0.7402	0.9767	0.0909
S1_2499198	1	2499198	S1_2553940	4	54.742	0.6817	0.9197	0.7567	0.9690	0.0784
S1_5199990	1	5199990	S1_5200948	2	0.958	0.6786	0.9014	0.8154	0.9881	0.0603
S1_31735826	1	31735826	S1_31736671	1	0.845	0.6759	0.9731	0.6768	0.9738	0.1877
S1_34297341	1	34297341	S1_34309486	1	12.145	0.6659	1.0000	0.6794	1.0000	0.1079
S1_14852237	1	14852237	S1_14852281	1	0.044	0.6354	1.0000	0.6339	0.9988	0.1101
S1_2476259	1	2476259	S1_2499198	2	22.939	0.6089	0.8403	0.6089	0.8403	0.0693
S1_27556580	1	27556580	S1_27560182	1	3.602	0.5877	1.0000	0.5935	1.0000	0.0517
S1_2553940	1	2553940	S1_2591201	3	37.261	0.5753	0.8228	0.5831	0.8284	0.0925
S1_31651969	1	31651969	S1_31652001	1	0.032	0.5520	1.0000	0.5602	1.0000	0.1024
S1_14847050	1	14847050	S1_14852228	2	5.178	0.5215	0.9047	0.5503	0.9293	0.0662
S1_34506525	1	34506525	S1_34506529	1	0.004	0.5134	1.0000	0.5184	1.0000	0.1585
S1_27555109	1	27555109	S1_27556580	1	1.471	0.5073	0.9004	0.5508	0.9381	0.0485
S1_2711268	1	2711268	S1_2821526	4	110.258	0.5050	0.7296	0.5189	0.7396	0.0743
S1_32070366	1	32070366	S1_32070504	1	0.138	0.4967	0.9240	0.5127	0.9387	0.1426
S1_20101125	1	20101125	S1_20101180	1	0.055	0.4826	0.8636	0.5746	0.9423	0.0759
S1_32561726	1	32561726	S1_32605230	3	43.504	0.4808	0.8004	0.4968	0.8137	0.0644
S1_11157882	1	11157882	S1_11159715	2	1.833	0.4744	1.0000	0.4694	0.9947	0.1527
S1_32065430	1	32065430	S1_32070366	1	4.936	0.4725	0.8840	0.4898	0.9000	0.1393
S1_31453983	1	31453983	S1_31464678	1	10.695	0.4657	0.7595	0.4735	0.7659	0.0401
S1_2439081	1	2439081	S1_2469547	2	30.466	0.3990	1.0000	0.4448	1.0000	0.0289

S1_12543226	1	12543226	S1_12543318	1	0.092	0.3575	0.7229	0.3696	0.7350	0.0457
S1_32057413	1	32057413	S1_32065430	1	8.017	0.3048	1.0000	0.2998	0.9919	0.1136
S1_32118739	1	32118739	S1_32118751	1	0.012	0.2844	1.0000	0.3049	1.0000	0.1040
S1_32118751	1	32118751	S1_32118763	1	0.012	0.2844	1.0000	0.3049	1.0000	0.1040
S1_1095486	1	1095486	S1_1095493	2	0.007	0.2772	0.5989	0.3681	0.6901	0.0363
S1_33552045	1	33552045	S1_33552049	1	0.004	0.2431	1.0000	0.2392	0.9919	-0.1067
S1_33552005	1	33552005	S1_33552045	1	0.04	0.2431	1.0000	0.2392	0.9919	-0.1067
S1_31422987	1	31422987	S1_31423008	1	0.021	0.0000	0.0000	0.0000	0.0000	0.0000
S2_1063431	2	1063431	S2_1063446	1	0.015	1.0000	1.0000	1.0000	1.0000	0.0566
S2_1063446	2	1063446	S2_1063458	1	0.012	1.0000	1.0000	1.0000	1.0000	0.0566
S2_1591748	2	1591748	S2_1592034	1	0.286	1.0000	1.0000	1.0000	1.0000	0.2469
S2_3238537	2	3238537	S2_3238550	1	0.013	1.0000	1.0000	1.0000	1.0000	0.0530
S2_15448280	2	15448280	S2_15448306	1	0.026	1.0000	1.0000	1.0000	1.0000	0.0519
S2_21209068	2	21209068	S2_21209077	1	0.009	1.0000	1.0000	1.0000	1.0000	0.0951
S2_22567330	2	22567330	S2_22567357	1	0.027	1.0000	1.0000	1.0000	1.0000	0.0880
S2_23345208	2	23345208	S2_23345243	1	0.035	1.0000	1.0000	1.0000	1.0000	0.0798
S2_23617962	2	23617962	S2_23617966	1	0.004	1.0000	1.0000	1.0000	1.0000	0.0871
S2_23617966	2	23617966	S2_23617988	1	0.022	1.0000	1.0000	1.0000	1.0000	0.0871
S2_8752307	2	8752307	S2_8752347	1	0.04	1.0000	1.0000	1.0000	1.0000	0.1181
S2_23345243	2	23345243	S2_23345264	1	0.021	1.0000	1.0000	1.0000	1.0000	0.0781
S2_1621032	2	1621032	S2_1621033	1	0.001	1.0000	1.0000	1.0000	1.0000	0.0499
S2_21368011	2	21368011	S2_21368014	1	0.003	1.0000	1.0000	1.0000	1.0000	0.0910
S2_15556180	2	15556180	S2_15556647	1	0.467	1.0000	1.0000	1.0000	1.0000	0.2075
S2_1805157	2	1805157	S2_1805170	1	0.013	1.0000	1.0000	1.0000	1.0000	0.2359
S2_17389425	2	17389425	S2_17389426	1	0.001	1.0000	1.0000	1.0000	1.0000	0.0525
S2_23248446	2	23248446	S2_23248458	1	0.012	1.0000	1.0000	1.0000	1.0000	0.1412
S2_23248351	2	23248351	S2_23248361	1	0.01	1.0000	1.0000	1.0000	1.0000	0.1401
S2_23248361	2	23248361	S2_23248391	1	0.03	1.0000	1.0000	1.0000	1.0000	0.1401
S2_9591890	2	9591890	S2_9591941	1	0.051	1.0000	1.0000	1.0000	1.0000	0.1413
S2_589764	2	589764	S2_589765	1	0.001	1.0000	1.0000	1.0000	1.0000	0.1560

S2_21209159	2	21209159	S2_21209171	1	0.012	1.0000	1.0000	1.0000	1.0000	0.0974
S2_15816858	2	15816858	S2_15816859	1	0.001	1.0000	1.0000	1.0000	1.0000	0.0883
S2_18205263	2	18205263	S2_18205264	1	0.001	1.0000	1.0000	1.0000	1.0000	0.1479
S2_18205264	2	18205264	S2_18205265	1	0.001	1.0000	1.0000	1.0000	1.0000	0.1479
S2_3109168	2	3109168	S2_3109169	1	0.001	1.0000	1.0000	1.0000	1.0000	0.1377
S2_17337396	2	17337396	S2_17337397	1	0.001	1.0000	1.0000	1.0000	1.0000	0.1377
S2_14071295	2	14071295	S2_14093339	3	22.044	1.0000	1.0000	1.0000	1.0000	0.2373
S2_13295303	2	13295303	S2_13296167	1	0.864	1.0000	1.0000	1.0000	1.0000	0.0684
S2_1805484	2	1805484	S2_1805524	1	0.04	1.0000	1.0000	1.0000	1.0000	0.0714
S2_13296167	2	13296167	S2_13298685	2	2.518	1.0000	1.0000	1.0000	1.0000	0.0755
S2_24359265	2	24359265	S2_24359266	1	0.001	1.0000	1.0000	1.0000	1.0000	0.1606
S2_18522406	2	18522406	S2_18522407	1	0.001	1.0000	1.0000	1.0000	1.0000	0.2100
S2_18522407	2	18522407	S2_18522449	1	0.042	1.0000	1.0000	1.0000	1.0000	0.2100
S2_18536671	2	18536671	S2_18536677	1	0.006	1.0000	1.0000	1.0000	1.0000	0.1860
S2_10829634	2	10829634	S2_10829637	1	0.003	1.0000	1.0000	1.0000	1.0000	0.1467
S2_5360639	2	5360639	S2_5360640	1	0.001	1.0000	1.0000	1.0000	1.0000	0.1553
S2_23873539	2	23873539	S2_23873569	1	0.03	1.0000	1.0000	1.0000	1.0000	0.2364
S2_23312722	2	23312722	S2_23312725	1	0.003	1.0000	1.0000	1.0000	1.0000	0.1005
S2_22695714	2	22695714	S2_22695722	1	0.008	1.0000	1.0000	1.0000	1.0000	0.1986
S2_22116732	2	22116732	S2_22116751	1	0.019	1.0000	1.0000	1.0000	1.0000	0.2321
S2_22116751	2	22116751	S2_22116753	1	0.002	1.0000	1.0000	1.0000	1.0000	0.2321
S2_22116753	2	22116753	S2_22116754	1	0.001	1.0000	1.0000	1.0000	1.0000	0.2321
S2_22562671	2	22562671	S2_22562761	1	0.09	1.0000	1.0000	1.0000	1.0000	0.1794
S2_21915648	2	21915648	S2_21915649	1	0.001	1.0000	1.0000	1.0000	1.0000	0.1007
S2_21915651	2	21915651	S2_21915652	1	0.001	1.0000	1.0000	1.0000	1.0000	0.0818
S2_20870192	2	20870192	S2_20871185	2	0.993	1.0000	1.0000	1.0000	1.0000	0.0649
S2_20974603	2	20974603	S2_20974604	1	0.001	1.0000	1.0000	1.0000	1.0000	0.2432
S2_23427627	2	23427627	S2_23428322	3	0.695	1.0000	1.0000	1.0000	1.0000	0.2371
S2_14765349	2	14765349	S2_14766131	1	0.782	1.0000	1.0000	1.0000	1.0000	0.2261
S2_20453230	2	20453230	S2_20453240	1	0.01	1.0000	1.0000	1.0000	1.0000	0.2287

S2_695485	2	695485	S2_695534	1	0.049	1.0000	1.0000	1.0000	1.0000	0.2487
S2_8595864	2	8595864	S2_8595888	1	0.024	1.0000	1.0000	1.0000	1.0000	0.2490
S2_14969757	2	14969757	S2_14969796	1	0.039	1.0000	1.0000	1.0000	1.0000	0.1834
S2_22975263	2	22975263	S2_22975304	1	0.041	1.0000	1.0000	1.0000	1.0000	0.1161
S2_22975304	2	22975304	S2_22975325	1	0.021	1.0000	1.0000	1.0000	1.0000	0.1161
S2_22975325	2	22975325	S2_22975340	1	0.015	1.0000	1.0000	1.0000	1.0000	0.1161
S2_3128830	2	3128830	S2_3128852	1	0.022	1.0000	1.0000	1.0000	1.0000	0.2327
S2_3128852	2	3128852	S2_3128855	1	0.003	1.0000	1.0000	1.0000	1.0000	0.2327
S2_14997885	2	14997885	S2_14997911	1	0.026	1.0000	1.0000	1.0000	1.0000	0.1725
S2_5214330	2	5214330	S2_5214348	1	0.018	1.0000	1.0000	1.0000	1.0000	0.2496
S2_10561529	2	10561529	S2_10561537	1	0.008	1.0000	1.0000	1.0000	1.0000	0.2302
S2_14786447	2	14786447	S2_14786458	1	0.011	1.0000	1.0000	1.0000	1.0000	0.2228
S2_554462	2	554462	S2_554483	1	0.021	1.0000	1.0000	1.0000	1.0000	0.1511
S2_554483	2	554483	S2_554484	1	0.001	1.0000	1.0000	1.0000	1.0000	0.1511
S2_15418078	2	15418078	S2_15418089	1	0.011	1.0000	1.0000	1.0000	1.0000	0.2334
S2_16301414	2	16301414	S2_16301437	1	0.023	1.0000	1.0000	1.0000	1.0000	0.1007
S2_1805471	2	1805471	S2_1805472	1	0.001	1.0000	1.0000	1.0000	1.0000	0.0507
S2_1805472	2	1805472	S2_1805473	1	0.001	1.0000	1.0000	1.0000	1.0000	0.0507
S2_1805473	2	1805473	S2_1805474	1	0.001	1.0000	1.0000	1.0000	1.0000	0.0507
S2_17951500	2	17951500	S2_17951511	1	0.011	1.0000	1.0000	1.0000	1.0000	0.0933
S2_23485285	2	23485285	S2_23486300	1	1.015	0.9794	1.0000	0.9894	1.0000	0.2457
S2_13803586	2	13803586	S2_13803595	1	0.009	0.9784	1.0000	0.9890	1.0000	0.2352
S2_23427623	2	23427623	S2_23427627	1	0.004	0.9784	1.0000	0.9886	1.0000	0.2352
S2_22447252	2	22447252	S2_22447314	2	0.062	0.9765	1.0000	0.9874	1.0000	0.2405
S2_23340093	2	23340093	S2_23340154	1	0.061	0.9762	1.0000	0.9874	1.0000	0.2144
S2_17663959	2	17663959	S2_17699772	1	35.813	0.9745	1.0000	0.9863	1.0000	0.1983
S2_12778543	2	12778543	S2_12785838	1	7.295	0.9677	1.0000	0.9837	1.0000	0.1641
S2_24554918	2	24554918	S2_24555148	2	0.23	0.9644	1.0000	0.9820	1.0000	0.1589
S2_4543452	2	4543452	S2_4543504	1	0.052	0.9644	1.0000	0.9813	1.0000	0.1588
S2_17436044	2	17436044	S2_17436149	1	0.105	0.9643	1.0000	0.9816	1.0000	0.1451

S2_4543504	2	4543504	S2_4543608	1	0.104	0.9637	1.0000	0.9810	1.0000	0.1675
S2_5389237	2	5389237	S2_5390442	2	1.205	0.9636	1.0000	0.9817	1.0000	0.1691
S2_23248458	2	23248458	S2_23248515	1	0.057	0.9636	1.0000	0.9816	1.0000	0.1405
S2_23248224	2	23248224	S2_23248351	1	0.127	0.9611	1.0000	0.9803	1.0000	0.1418
S2_23769220	2	23769220	S2_23769235	1	0.015	0.9559	1.0000	0.9527	0.9983	0.2327
S2_14093339	2	14093339	S2_14104172	1	10.833	0.9555	1.0000	0.9773	1.0000	0.2333
S2_14068379	2	14068379	S2_14071295	3	2.916	0.9520	1.0000	0.9756	1.0000	0.2390
S2_18916010	2	18916010	S2_18916042	1	0.032	0.9460	1.0000	0.9712	1.0000	0.1847
S2_20505073	2	20505073	S2_20505192	2	0.119	0.9378	0.9787	0.9663	0.9934	0.2403
S2_23327238	2	23327238	S2_23327334	1	0.096	0.9376	0.9683	0.9673	0.9835	0.2216
S2_8975324	2	8975324	S2_8994193	1	18.869	0.9347	1.0000	0.9665	1.0000	0.0899
S2_23769235	2	23769235	S2_23771660	1	2.425	0.9344	1.0000	0.9413	1.0000	0.2307
S2_23771660	2	23771660	S2_23782926	1	11.266	0.9315	0.9764	0.9634	0.9930	0.2294
S2_8591626	2	8591626	S2_8595851	2	4.225	0.9281	1.0000	0.9629	1.0000	0.0685
S2_2601269	2	2601269	S2_2601333	1	0.064	0.9263	0.9746	0.9607	0.9925	0.2035
S2_12970871	2	12970871	S2_12970875	1	0.004	0.9256	1.0000	0.9619	1.0000	0.1350
S2_23248391	2	23248391	S2_23248446	1	0.055	0.9239	0.9612	0.9608	0.9802	0.1373
S2_24054406	2	24054406	S2_24054415	1	0.009	0.9221	1.0000	0.9189	0.9983	0.1218
S2_13799066	2	13799066	S2_13803586	1	4.52	0.9212	1.0000	0.9599	1.0000	0.2237
S2_21522250	2	21522250	S2_21522263	1	0.013	0.9194	1.0000	0.9160	0.9981	0.2349
S2_18908380	2	18908380	S2_18908477	2	0.097	0.9194	0.9721	0.9575	0.9921	0.1835
S2_7819145	2	7819145	S2_7819165	1	0.02	0.9180	1.0000	0.9591	1.0000	0.0590
S2_22786333	2	22786333	S2_22786363	1	0.03	0.9180	1.0000	0.9591	1.0000	0.0590
S2_21720264	2	21720264	S2_21720268	1	0.004	0.9145	1.0000	0.9116	0.9984	0.2224
S2_12522799	2	12522799	S2_12602347	3	79.548	0.9122	0.9695	0.9542	0.9916	0.1915
S2_7605964	2	7605964	S2_7644352	3	38.388	0.9121	1.0000	0.9236	1.0000	0.1615
S2_18429953	2	18429953	S2_18430166	1	0.213	0.9113	0.9692	0.9230	0.9754	0.2296
S2_20871185	2	20871185	S2_20875690	2	4.505	0.9111	1.0000	0.9572	1.0000	0.1180
S2_13792789	2	13792789	S2_13792800	2	0.011	0.9098	1.0000	0.9543	1.0000	0.2295
S2_19076456	2	19076456	S2_19079925	1	3.469	0.9076	0.9756	0.9531	0.9998	0.2385

S2_24555148	2	24555148	S2_24574237	1	19.089	0.9054	0.9671	0.9513	0.9913	0.1551
S2_22639434	2	22639434	S2_22693495	1	54.061	0.9048	1.0000	0.9048	1.0000	0.1080
S2_22780924	2	22780924	S2_22781086	1	0.162	0.9047	0.9511	0.9499	0.9746	0.1446
S2_15537282	2	15537282	S2_15556180	1	18.898	0.9042	0.9747	0.9260	0.9863	0.2028
S2_22116887	2	22116887	S2_22116934	1	0.047	0.9015	0.9495	0.9470	0.9731	0.2295
S2_17163044	2	17163044	S2_17229030	1	65.986	0.9014	1.0000	0.9474	1.0000	0.1053
S2_22116754	2	22116754	S2_22116887	1	0.133	0.8995	1.0000	0.9477	1.0000	0.2268
S2_146775	2	146775	S2_164392	2	17.617	0.8990	0.9481	0.9481	0.9737	0.1055
S2_1805170	2	1805170	S2_1805185	1	0.015	0.8977	1.0000	0.7840	0.9345	0.2113
S2_21298758	2	21298758	S2_21300090	2	1.332	0.8974	1.0000	0.9436	1.0000	0.1020
S2_21720246	2	21720246	S2_21720264	1	0.018	0.8926	0.9769	0.8996	0.9807	0.2216
S2_247209	2	247209	S2_305110	2	57.901	0.8892	0.9611	0.9045	0.9693	0.1326
S2_16261690	2	16261690	S2_16286568	1	24.878	0.8887	0.9536	0.9192	0.9698	0.2298
S2_5895586	2	5895586	S2_5895727	1	0.141	0.8860	0.9695	0.9122	0.9837	0.1857
S2_14229246	2	14229246	S2_14232689	3	3.443	0.8815	0.9505	0.9149	0.9683	0.2221
S2_5360640	2	5360640	S2_5389237	2	28.597	0.8790	0.9574	0.9372	0.9885	0.1632
S2_23954958	2	23954958	S2_23976584	2	21.626	0.8789	1.0000	0.9366	1.0000	0.0772
S2_7368740	2	7368740	S2_7375537	2	6.797	0.8782	1.0000	0.9390	1.0000	0.2359
S2_23327334	2	23327334	S2_23327971	1	0.637	0.8772	1.0000	0.8732	0.9977	0.1980
S2_23442015	2	23442015	S2_23443289	1	1.274	0.8760	1.0000	0.9050	1.0000	0.1752
S2_13792800	2	13792800	S2_13799066	3	6.266	0.8722	1.0000	0.9354	1.0000	0.2236
S2_14969845	2	14969845	S2_14969882	1	0.037	0.8713	1.0000	0.8669	0.9975	0.0776
S2_8591619	2	8591619	S2_8591626	1	0.007	0.8653	1.0000	0.9285	1.0000	0.0686
S2_23336249	2	23336249	S2_23336286	1	0.037	0.8641	1.0000	0.8641	1.0000	0.0731
S2_23617988	2	23617988	S2_23618001	1	0.013	0.8635	1.0000	0.8635	1.0000	0.0762
S2_15202307	2	15202307	S2_15202638	1	0.331	0.8625	0.9746	0.9050	0.9984	0.2263
S2_23617817	2	23617817	S2_23617962	1	0.145	0.8615	1.0000	0.9356	1.0000	0.0901
S2_21358372	2	21358372	S2_21358902	1	0.53	0.8598	1.0000	0.8778	1.0000	0.0958
S2_21522263	2	21522263	S2_21525689	1	3.426	0.8576	0.9561	0.8832	0.9702	0.2302
S2_14616378	2	14616378	S2_14765349	1	148.971	0.8574	0.9492	0.8731	0.9578	0.2053

S2_695358	2	695358	S2_695427	1	0.069	0.8557	0.9732	0.8740	0.9835	0.1898
S2_16301437	2	16301437	S2_16301449	1	0.012	0.8528	1.0000	0.8488	0.9976	0.0986
S2_16301449	2	16301449	S2_16301450	1	0.001	0.8528	1.0000	0.8488	0.9976	0.0986
S2_23326141	2	23326141	S2_23327238	1	1.097	0.8513	0.9671	0.9220	1.0000	0.2068
S2_305110	2	305110	S2_308272	1	3.162	0.8496	1.0000	0.8854	1.0000	0.1214
S2_14604518	2	14604518	S2_14606566	2	2.048	0.8487	0.9458	0.9182	0.9838	0.2166
S2_18529828	2	18529828	S2_18536671	1	6.843	0.8470	0.9351	0.9186	0.9739	0.1768
S2_170681	2	170681	S2_171317	1	0.636	0.8435	1.0000	0.8643	1.0000	0.1926
S2_4178031	2	4178031	S2_4185514	1	7.483	0.8274	0.9521	0.8592	0.9702	0.1224
S2_22567357	2	22567357	S2_22634067	6	66.71	0.8250	1.0000	0.8503	1.0000	0.0876
S2_18879765	2	18879765	S2_18908380	1	28.615	0.8232	0.9359	0.8447	0.9480	0.1723
S2_1679354	2	1679354	S2_1680344	1	0.99	0.8223	1.0000	0.8276	1.0000	0.2222
S2_12288666	2	12288666	S2_12353175	2	64.509	0.8194	0.9226	0.8355	0.9316	0.1253
S2_15370110	2	15370110	S2_15370356	1	0.246	0.8185	0.9341	0.9009	0.9799	0.0885
S2_15369460	2	15369460	S2_15370079	1	0.619	0.8172	1.0000	0.8482	1.0000	0.0804
S2_16078517	2	16078517	S2_16138325	1	59.808	0.8133	1.0000	0.8449	1.0000	0.0952
S2_24533068	2	24533068	S2_24554918	3	21.85	0.8119	1.0000	0.8119	1.0000	0.1542
S2_24448282	2	24448282	S2_24533068	1	84.786	0.7947	1.0000	0.8089	1.0000	0.1470
S2_14104172	2	14104172	S2_14109274	3	5.102	0.7902	1.0000	0.8289	1.0000	0.2243
S2_18756157	2	18756157	S2_18839996	1	83.839	0.7900	0.9645	0.8309	0.9892	0.1678
S2_14996180	2	14996180	S2_14997885	2	1.705	0.7806	1.0000	0.8493	1.0000	0.1476
S2_1679268	2	1679268	S2_1679269	1	0.001	0.7762	1.0000	0.7359	0.9736	0.1501
S2_23345264	2	23345264	S2_23345277	1	0.013	0.7738	0.9159	0.8075	0.9356	0.0678
S2_23345277	2	23345277	S2_23345293	1	0.016	0.7738	0.9159	0.8075	0.9356	0.0678
S2_3107847	2	3107847	S2_3109092	1	1.245	0.7703	0.9145	0.8030	0.9336	0.1364
S2_8752347	2	8752347	S2_8756273	1	3.926	0.7697	0.9542	0.8015	0.9737	0.1132
S2_21695763	2	21695763	S2_21705052	1	9.289	0.7680	0.9276	0.8103	0.9528	0.2175
S2_5338413	2	5338413	S2_5360639	3	22.226	0.7663	0.8754	0.8342	0.9133	0.1447
S2_23617669	2	23617669	S2_23617817	1	0.148	0.7562	1.0000	0.7399	0.9892	0.0777
S2_12353432	2	12353432	S2_12370274	1	16.842	0.7509	0.9063	0.7851	0.9266	0.1267

S2_15370373	2	15370373	S2_15370385	1	0.012	0.7505	1.0000	0.7793	1.0000	0.0958
S2_21339215	2	21339215	S2_21358372	1	19.157	0.7409	0.9473	0.7812	0.9726	0.0981
S2_12829503	2	12829503	S2_12964911	1	135.408	0.7368	1.0000	0.7550	1.0000	0.1340
S2_24052173	2	24052173	S2_24054406	1	2.233	0.7353	1.0000	0.7450	1.0000	0.1009
S2_308272	2	308272	S2_308300	1	0.028	0.7336	1.0000	0.7558	1.0000	0.1177
S2_23576258	2	23576258	S2_23617669	2	41.411	0.7333	0.9212	0.7827	0.9517	0.0695
S2_24332069	2	24332069	S2_24346034	3	13.965	0.7238	0.9429	0.7570	0.9642	0.0871
S2_21705052	2	21705052	S2_21720246	2	15.194	0.7141	0.9745	0.7138	0.9743	0.2021
S2_12964911	2	12964911	S2_12970708	1	5.797	0.7028	1.0000	0.7449	1.0000	0.1289
S2_12970875	2	12970875	S2_12972854	2	1.979	0.7025	1.0000	0.7256	1.0000	0.1277
S2_23239618	2	23239618	S2_23248224	2	8.606	0.6962	1.0000	0.6962	1.0000	0.1019
S2_5338373	2	5338373	S2_5338413	2	0.04	0.6875	0.9581	0.7405	0.9943	0.1276
S2_1623351	2	1623351	S2_1679255	2	55.904	0.6815	0.9499	0.6987	0.9618	0.2031
S2_22695793	2	22695793	S2_22780924	1	85.131	0.6815	0.9024	0.6753	0.8982	0.1138
S2_8895643	2	8895643	S2_8975324	1	79.681	0.6660	0.9146	0.6951	0.9344	0.0676
S2_20791297	2	20791297	S2_20834219	1	42.922	0.6570	0.8753	0.7401	0.9290	0.1153
S2_12970708	2	12970708	S2_12970871	1	0.163	0.6545	0.9594	0.7020	0.9936	0.1344
S2_17936204	2	17936204	S2_17951450	4	15.246	0.6531	0.9331	0.6957	0.9631	0.0769
S2_22634067	2	22634067	S2_22639434	4	5.367	0.6475	1.0000	0.6727	1.0000	0.0965
S2_5338280	2	5338280	S2_5338373	1	0.093	0.6344	0.9427	0.6521	0.9558	0.1005
S2_15884922	2	15884922	S2_15962857	1	77.935	0.6312	0.8279	0.6520	0.8414	0.1100
S2_23559582	2	23559582	S2_23576258	1	16.676	0.6289	0.8679	0.7265	0.9328	0.0761
S2_20834219	2	20834219	S2_20870112	1	35.893	0.6253	0.9152	0.7226	0.9838	0.0637
S2_308300	2	308300	S2_360780	1	52.48	0.6057	0.9090	0.6535	0.9442	0.1104
S2_21209077	2	21209077	S2_21209102	1	0.025	0.5592	1.0000	0.5696	1.0000	0.0885
S2_1805524	2	1805524	S2_1819256	1	13.732	0.5378	0.8025	0.5900	0.8406	0.0519
S2_7281378	2	7281378	S2_7292914	1	11.536	0.5283	0.9231	0.5565	0.9474	0.1374
S2_21209102	2	21209102	S2_21209159	1	0.057	0.5275	1.0000	0.5536	1.0000	0.0861
S2_5338140	2	5338140	S2_5338280	1	0.14	0.5254	0.7955	0.5435	0.8091	0.0813
S2_20875690	2	20875690	S2_20875718	1	0.028	0.5000	1.0000	0.5000	1.0000	0.0659

S2_6248554	2	6248554	S2_6250775	5	2.221	0.4750	1.0000	0.4807	1.0000	0.1272
S2_24170825	2	24170825	S2_24180581	2	9.756	0.4701	0.7625	0.4475	0.7439	0.0349
S2_17762155	2	17762155	S2_17834292	1	72.137	0.4461	0.8983	0.5449	0.9929	0.0522
S2_1819256	2	1819256	S2_1834327	2	15.071	0.4455	0.7469	0.4453	0.7467	0.0472
S2_22116934	2	22116934	S2_22117028	1	0.094	0.4156	0.9562	0.4220	0.9635	0.1278
S2_12207164	2	12207164	S2_12207203	1	0.039	0.3586	1.0000	0.3586	1.0000	0.0541
S2_12207203	2	12207203	S2_12208474	1	1.271	0.3586	1.0000	0.3735	1.0000	0.0552
S2_7280861	2	7280861	S2_7281378	1	0.517	0.2974	1.0000	0.2957	0.9972	0.1104
S2_7279885	2	7279885	S2_7280861	2	0.976	0.2970	1.0000	0.3059	1.0000	0.1132
S2_24180581	2	24180581	S2_24208490	2	27.909	0.2736	0.5965	0.2649	0.5869	0.0386
S2_1834327	2	1834327	S2_1891052	1	56.725	0.2430	0.5301	0.2827	0.5718	0.0401
S2_22117028	2	22117028	S2_22117081	1	0.053	0.2397	0.9468	0.2396	0.9467	0.0992
S2_20875730	2	20875730	S2_20891968	3	16.238	0.2152	1.0000	0.2225	1.0000	0.0232
S2_1621015	2	1621015	S2_1621032	1	0.017	0.0737	1.0000	0.0762	1.0000	0.0309
S2_1621033	2	1621033	S2_1621068	1	0.035	0.0644	1.0000	0.0658	1.0000	0.0286
S2_13295285	2	13295285	S2_13295303	1	0.018	0.0366	0.9997	0.0305	0.9125	-0.0215
S2_13295265	2	13295265	S2_13295285	1	0.02	0.0366	0.9997	0.0305	0.9125	-0.0215
S2_20875718	2	20875718	S2_20875730	1	0.012	0.0060	1.0000	0.0060	1.0000	-0.0052
S2_12249486	2	12249486	S2_12288666	1	39.18	0.0035	1.0000	0.0042	1.0000	-0.0034
S2_12208474	2	12208474	S2_12249486	4	41.012	0.0020	1.0000	0.0030	1.0000	-0.0023
S2_20870112	2	20870112	S2_20870192	1	0.08	0.0001	1.0000	0.0003	1.0000	-0.0001
S3_7342864	3	7342864	S3_7342931	1	0.067	1.0000	1.0000	1.0000	1.0000	0.0842
S3_11061295	3	11061295	S3_11078456	1	17.161	1.0000	1.0000	1.0000	1.0000	0.2461
S3_11078456	3	11078456	S3_11102665	2	24.209	1.0000	1.0000	1.0000	1.0000	0.2479
S3_17434839	3	17434839	S3_17434871	1	0.032	1.0000	1.0000	1.0000	1.0000	0.0642
S3_17434871	3	17434871	S3_17434874	1	0.003	1.0000	1.0000	1.0000	1.0000	0.0642
S3_23485104	3	23485104	S3_23485355	2	0.251	1.0000	1.0000	1.0000	1.0000	0.0771
S3_26049178	3	26049178	S3_26049206	1	0.028	1.0000	1.0000	1.0000	1.0000	0.2320
S3_8570119	3	8570119	S3_8570122	1	0.003	1.0000	1.0000	1.0000	1.0000	0.0598
S3_16110867	3	16110867	S3_16141813	1	30.946	1.0000	1.0000	1.0000	1.0000	0.0953

S3_17467577	3	17467577	S3_17467578	1	0.001	1.0000	1.0000	1.0000	1.0000	0.0779
S3_16141813	3	16141813	S3_16142962	1	1.149	1.0000	1.0000	1.0000	1.0000	0.0977
S3_24993753	3	24993753	S3_24993764	1	0.011	1.0000	1.0000	1.0000	1.0000	0.2499
S3_19175371	3	19175371	S3_19175380	1	0.009	1.0000	1.0000	1.0000	1.0000	0.2483
S3_14954857	3	14954857	S3_14954917	1	0.06	1.0000	1.0000	1.0000	1.0000	0.2320
S3_3639874	3	3639874	S3_3639897	1	0.023	1.0000	1.0000	1.0000	1.0000	0.2500
S3_22161060	3	22161060	S3_22161078	1	0.018	1.0000	1.0000	1.0000	1.0000	0.1914
S3_23317576	3	23317576	S3_23317577	1	0.001	1.0000	1.0000	1.0000	1.0000	0.1718
S3_19433031	3	19433031	S3_19433045	1	0.014	1.0000	1.0000	1.0000	1.0000	0.2045
S3_22670092	3	22670092	S3_22670095	1	0.003	1.0000	1.0000	1.0000	1.0000	0.1479
S3_22670095	3	22670095	S3_22670125	1	0.03	1.0000	1.0000	1.0000	1.0000	0.1479
S3_26294800	3	26294800	S3_26294850	1	0.05	1.0000	1.0000	1.0000	1.0000	0.1479
S3_22647995	3	22647995	S3_22648051	1	0.056	1.0000	1.0000	1.0000	1.0000	0.1459
S3_22647873	3	22647873	S3_22647995	1	0.122	1.0000	1.0000	1.0000	1.0000	0.1484
S3_26295755	3	26295755	S3_26295788	1	0.033	1.0000	1.0000	1.0000	1.0000	0.1607
S3_26295788	3	26295788	S3_26295810	1	0.022	1.0000	1.0000	1.0000	1.0000	0.1607
S3_25182650	3	25182650	S3_25182651	1	0.001	1.0000	1.0000	1.0000	1.0000	0.1279
S3_25182651	3	25182651	S3_25182652	1	0.001	1.0000	1.0000	1.0000	1.0000	0.1279
S3_25454038	3	25454038	S3_25454047	1	0.009	1.0000	1.0000	1.0000	1.0000	0.0965
S3_24899414	3	24899414	S3_24899482	1	0.068	1.0000	1.0000	1.0000	1.0000	0.0974
S3_3439646	3	3439646	S3_3439648	1	0.002	1.0000	1.0000	1.0000	1.0000	0.1791
S3_15971327	3	15971327	S3_15980914	1	9.587	1.0000	1.0000	1.0000	1.0000	0.0883
S3_15762885	3	15762885	S3_15762986	1	0.101	1.0000	1.0000	1.0000	1.0000	0.0892
S3_16142962	3	16142962	S3_16148061	2	5.099	1.0000	1.0000	1.0000	1.0000	0.0900
S3_10144486	3	10144486	S3_10144493	1	0.007	1.0000	1.0000	1.0000	1.0000	0.1046
S3_17954112	3	17954112	S3_17954113	1	0.001	1.0000	1.0000	1.0000	1.0000	0.2306
S3_15762986	3	15762986	S3_15763023	1	0.037	1.0000	1.0000	1.0000	1.0000	0.0788
S3_5750431	3	5750431	S3_5750432	1	0.001	1.0000	1.0000	1.0000	1.0000	0.2259
S3_7292180	3	7292180	S3_7292201	1	0.021	1.0000	1.0000	1.0000	1.0000	0.1931
S3_25225359	3	25225359	S3_25225360	1	0.001	1.0000	1.0000	1.0000	1.0000	0.0694

S3_20865752	3	20865752	S3_20865753	1	0.001	1.0000	1.0000	1.0000	1.0000	0.1600
S3_19977076	3	19977076	S3_19977077	1	0.001	1.0000	1.0000	1.0000	1.0000	0.2478
S3_688816	3	688816	S3_689437	1	0.621	1.0000	1.0000	1.0000	1.0000	0.1570
S3_20875857	3	20875857	S3_20875878	1	0.021	1.0000	1.0000	1.0000	1.0000	0.2031
S3_17636887	3	17636887	S3_17636892	1	0.005	1.0000	1.0000	1.0000	1.0000	0.1005
S3_21005190	3	21005190	S3_21005211	1	0.021	1.0000	1.0000	1.0000	1.0000	0.2024
S3_7084320	3	7084320	S3_7084323	1	0.003	1.0000	1.0000	1.0000	1.0000	0.2455
S3_7084323	3	7084323	S3_7084324	1	0.001	1.0000	1.0000	1.0000	1.0000	0.2455
S3_8788261	3	8788261	S3_8788263	1	0.002	1.0000	1.0000	1.0000	1.0000	0.1875
S3_23409864	3	23409864	S3_23409865	1	0.001	1.0000	1.0000	1.0000	1.0000	0.1631
S3_23409865	3	23409865	S3_23409866	1	0.001	1.0000	1.0000	1.0000	1.0000	0.1631
S3_20556812	3	20556812	S3_20556819	1	0.007	1.0000	1.0000	1.0000	1.0000	0.1606
S3_688309	3	688309	S3_688372	1	0.063	1.0000	1.0000	1.0000	1.0000	0.1619
S3_23772832	3	23772832	S3_23772837	1	0.005	1.0000	1.0000	1.0000	1.0000	0.2080
S3_745877	3	745877	S3_745888	1	0.011	1.0000	1.0000	1.0000	1.0000	0.1657
S3_573859	3	573859	S3_573860	1	0.001	1.0000	1.0000	1.0000	1.0000	0.2498
S3_573860	3	573860	S3_573862	1	0.002	1.0000	1.0000	1.0000	1.0000	0.2498
S3_18366316	3	18366316	S3_18366318	1	0.002	1.0000	1.0000	1.0000	1.0000	0.2440
S3_3439860	3	3439860	S3_3439896	1	0.036	1.0000	1.0000	1.0000	1.0000	0.0676
S3_26117111	3	26117111	S3_26117125	1	0.014	1.0000	1.0000	1.0000	1.0000	0.2009
S3_24197689	3	24197689	S3_24197690	1	0.001	1.0000	1.0000	1.0000	1.0000	0.2486
S3_24197690	3	24197690	S3_24197691	1	0.001	1.0000	1.0000	1.0000	1.0000	0.2486
S3_20852029	3	20852029	S3_20852127	2	0.098	1.0000	1.0000	1.0000	1.0000	0.0976
S3_8772894	3	8772894	S3_8772896	1	0.002	1.0000	1.0000	1.0000	1.0000	0.1913
S3_8772896	3	8772896	S3_8772900	1	0.004	1.0000	1.0000	1.0000	1.0000	0.1913
S3_7658304	3	7658304	S3_7658320	1	0.016	1.0000	1.0000	1.0000	1.0000	0.2500
S3_3523483	3	3523483	S3_3523501	1	0.018	1.0000	1.0000	1.0000	1.0000	0.1862
S3_21735942	3	21735942	S3_21735943	1	0.001	1.0000	1.0000	1.0000	1.0000	0.1614
S3_5525883	3	5525883	S3_5525905	1	0.022	1.0000	1.0000	1.0000	1.0000	0.2034
S3_5525905	3	5525905	S3_5525923	1	0.018	1.0000	1.0000	1.0000	1.0000	0.2034

S3_20789425	3	20789425	S3_20789445	1	0.02	1.0000	1.0000	1.0000	1.0000	0.1637
S3_17934709	3	17934709	S3_17934744	1	0.035	1.0000	1.0000	1.0000	1.0000	0.0965
S3_14731289	3	14731289	S3_14731304	1	0.015	1.0000	1.0000	1.0000	1.0000	0.2292
S3_23843551	3	23843551	S3_23843552	1	0.001	1.0000	1.0000	1.0000	1.0000	0.2117
S3_199559	3	199559	S3_199560	1	0.001	1.0000	1.0000	1.0000	1.0000	0.2470
S3_19833243	3	19833243	S3_19833261	1	0.018	1.0000	1.0000	1.0000	1.0000	0.2493
S3_6587312	3	6587312	S3_6587321	1	0.009	1.0000	1.0000	1.0000	1.0000	0.1888
S3_25938906	3	25938906	S3_25938907	1	0.001	1.0000	1.0000	1.0000	1.0000	0.2009
S3_25938907	3	25938907	S3_25938908	1	0.001	1.0000	1.0000	1.0000	1.0000	0.2009
S3_26564924	3	26564924	S3_26564938	1	0.014	1.0000	1.0000	1.0000	1.0000	0.0929
S3_20289935	3	20289935	S3_20289937	1	0.002	1.0000	1.0000	1.0000	1.0000	0.2001
S3_5526628	3	5526628	S3_5526668	1	0.04	1.0000	1.0000	1.0000	1.0000	0.1939
S3_21548077	3	21548077	S3_21548092	1	0.015	1.0000	1.0000	1.0000	1.0000	0.1038
S3_3389136	3	3389136	S3_3389137	1	0.001	1.0000	1.0000	1.0000	1.0000	0.2083
S3_3389137	3	3389137	S3_3389138	1	0.001	1.0000	1.0000	1.0000	1.0000	0.2083
S3_26537286	3	26537286	S3_26537288	1	0.002	1.0000	1.0000	1.0000	1.0000	0.0883
S3_26565265	3	26565265	S3_26565315	1	0.05	1.0000	1.0000	1.0000	1.0000	0.0883
S3_26411486	3	26411486	S3_26411487	1	0.001	1.0000	1.0000	1.0000	1.0000	0.0900
S3_26411487	3	26411487	S3_26411488	1	0.001	1.0000	1.0000	1.0000	1.0000	0.0900
S3_26375911	3	26375911	S3_26375912	1	0.001	1.0000	1.0000	1.0000	1.0000	0.2108
S3_18526166	3	18526166	S3_18526167	1	0.001	1.0000	1.0000	1.0000	1.0000	0.0965
S3_18526167	3	18526167	S3_18526170	1	0.003	1.0000	1.0000	1.0000	1.0000	0.0965
S3_18526170	3	18526170	S3_18526194	1	0.024	1.0000	1.0000	1.0000	1.0000	0.0965
S3_3025881	3	3025881	S3_3025908	1	0.027	0.9793	1.0000	0.9890	1.0000	0.2475
S3_19977077	3	19977077	S3_19980709	1	3.632	0.9792	1.0000	0.9893	1.0000	0.2467
S3_6149988	3	6149988	S3_6150214	1	0.226	0.9791	1.0000	0.9890	1.0000	0.2456
S3_18366318	3	18366318	S3_18366392	1	0.074	0.9791	1.0000	0.9891	1.0000	0.2423
S3_24441403	3	24441403	S3_24441438	1	0.035	0.9784	1.0000	0.9886	1.0000	0.2448
S3_25875968	3	25875968	S3_25876015	1	0.047	0.9774	1.0000	0.9881	1.0000	0.2239
S3_11102665	3	11102665	S3_11112187	2	9.522	0.9749	1.0000	0.9875	1.0000	0.2476

S3_25618566	3	25618566	S3_25618664	1	0.098	0.9717	1.0000	0.9851	1.0000	0.1782
S3_26175228	3	26175228	S3_26188028	4	12.8	0.9710	1.0000	0.9840	1.0000	0.1792
S3_22531466	3	22531466	S3_22531565	1	0.099	0.9680	1.0000	0.9821	1.0000	0.1589
S3_688372	3	688372	S3_688816	1	0.444	0.9679	1.0000	0.9833	1.0000	0.1602
S3_23048694	3	23048694	S3_23054984	1	6.29	0.9655	1.0000	0.9823	1.0000	0.1810
S3_2291343	3	2291343	S3_2291379	1	0.036	0.9592	1.0000	0.9570	0.9988	0.2444
S3_24074882	3	24074882	S3_24074893	1	0.011	0.9558	1.0000	0.9770	1.0000	0.1128
S3_23387004	3	23387004	S3_23387021	1	0.017	0.9558	1.0000	0.9537	0.9989	0.2248
S3_25939298	3	25939298	S3_25941815	2	2.517	0.9547	1.0000	0.9764	1.0000	0.2316
S3_17072690	3	17072690	S3_17072707	1	0.017	0.9544	1.0000	0.9756	1.0000	0.1090
S3_22885886	3	22885886	S3_22885890	1	0.004	0.9541	1.0000	0.9518	0.9988	0.2207
S3_22630590	3	22630590	S3_22633279	2	2.689	0.9538	0.9766	0.9762	0.9880	0.2458
S3_7550886	3	7550886	S3_7561562	2	10.676	0.9530	1.0000	0.9524	0.9997	0.2276
S3_25876105	3	25876105	S3_25882191	1	6.086	0.9515	1.0000	0.9749	1.0000	0.2048
S3_17768333	3	17768333	S3_17769213	1	0.88	0.9479	1.0000	0.9738	1.0000	0.2396
S3_26425614	3	26425614	S3_26425634	1	0.02	0.9446	1.0000	0.9669	1.0000	0.0889
S3_26189955	3	26189955	S3_26190126	1	0.171	0.9434	0.9713	0.9686	0.9842	0.1806
S3_7292201	3	7292201	S3_7293644	3	1.443	0.9420	0.9706	0.9697	0.9847	0.1901
S3_15959751	3	15959751	S3_15971327	1	11.576	0.9419	1.0000	0.9703	1.0000	0.0857
S3_26188028	3	26188028	S3_26189955	1	1.927	0.9413	0.9702	0.9673	0.9835	0.1776
S3_19432891	3	19432891	S3_19433031	1	0.14	0.9402	1.0000	0.9699	1.0000	0.2158
S3_19682453	3	19682453	S3_19682480	1	0.027	0.9399	1.0000	0.9492	1.0000	0.2432
S3_25761803	3	25761803	S3_25764733	1	2.93	0.9384	0.9789	0.9668	0.9936	0.2429
S3_568915	3	568915	S3_569126	1	0.211	0.9364	0.9782	0.9661	0.9935	0.2451
S3_26537288	3	26537288	S3_26564924	3	27.636	0.9348	1.0000	0.9650	1.0000	0.0888
S3_26564938	3	26564938	S3_26565265	1	0.327	0.9348	1.0000	0.9650	1.0000	0.0888
S3_26194682	3	26194682	S3_26194800	1	0.118	0.9346	0.9775	0.9633	0.9924	0.2328
S3_22808950	3	22808950	S3_22808991	1	0.041	0.9340	1.0000	0.9329	0.9994	0.1459
S3_26294742	3	26294742	S3_26294800	1	0.058	0.9340	1.0000	0.9329	0.9994	0.1459
S3_7733662	3	7733662	S3_7734012	1	0.35	0.9327	0.9769	0.9652	0.9937	0.2415

S3_26204845	3	26204845	S3_26204871	1	0.026	0.9269	1.0000	0.9339	1.0000	0.2329
S3_26199867	3	26199867	S3_26204845	1	4.978	0.9239	0.9737	0.9573	0.9912	0.2332
S3_9903356	3	9903356	S3_9903974	2	0.618	0.9222	0.9603	0.9591	0.9793	0.1313
S3_2038961	3	2038961	S3_2039022	1	0.061	0.9197	1.0000	0.9604	1.0000	0.1220
S3_19410141	3	19410141	S3_19432891	4	22.75	0.9194	0.9721	0.9587	0.9927	0.2172
S3_25225358	3	25225358	S3_25225359	1	0.001	0.9168	1.0000	0.9338	1.0000	0.0695
S3_25058370	3	25058370	S3_25058403	1	0.033	0.9164	1.0000	0.9164	1.0000	0.1231
S3_224195	3	224195	S3_260821	2	36.626	0.9161	0.9779	0.9539	0.9979	0.2338
S3_19931694	3	19931694	S3_19931731	3	0.037	0.9148	1.0000	0.9129	0.9990	0.2308
S3_23438864	3	23438864	S3_23439037	1	0.173	0.9137	0.9773	0.9546	0.9990	0.2394
S3_18396388	3	18396388	S3_18411505	1	15.117	0.9134	0.9772	0.9311	0.9866	0.2356
S3_23387021	3	23387021	S3_23387027	1	0.006	0.9118	1.0000	0.9076	0.9977	0.2148
S3_7658320	3	7658320	S3_7732233	1	73.913	0.9070	1.0000	0.9269	1.0000	-0.2404
S3_17598091	3	17598091	S3_17598192	1	0.101	0.9058	1.0000	0.9518	1.0000	0.2241
S3_24069637	3	24069637	S3_24069638	1	0.001	0.9041	1.0000	0.8986	0.9969	0.0485
S3_14731261	3	14731261	S3_14731289	1	0.028	0.9041	0.9746	0.9486	0.9984	0.2264
S3_25480716	3	25480716	S3_25481028	1	0.312	0.9036	1.0000	0.9497	1.0000	0.0546
S3_7732233	3	7732233	S3_7732321	1	0.088	0.8973	1.0000	0.9249	1.0000	0.2360
S3_8772900	3	8772900	S3_8774897	2	1.997	0.8970	0.9726	0.9182	0.9841	0.1856
S3_22229398	3	22229398	S3_22229455	1	0.057	0.8966	0.9725	0.9462	0.9991	0.2193
S3_573838	3	573838	S3_573859	1	0.021	0.8961	0.9568	0.9445	0.9823	0.2421
S3_23773363	3	23773363	S3_23773484	1	0.121	0.8960	0.9724	0.9461	0.9992	0.2033
S3_65075	3	65075	S3_67348	1	2.273	0.8943	0.9560	0.9421	0.9812	0.2326
S3_3466656	3	3466656	S3_3466698	2	0.042	0.8927	0.9553	0.9413	0.9809	0.2292
S3_26041244	3	26041244	S3_26049178	3	7.934	0.8919	0.9767	0.9162	0.9899	0.2200
S3_18528496	3	18528496	S3_18554916	3	26.42	0.8847	0.9406	0.9397	0.9694	0.0983
S3_23317585	3	23317585	S3_23325427	1	7.842	0.8782	1.0000	0.8782	1.0000	0.0792
S3_19682057	3	19682057	S3_19682453	1	0.396	0.8779	0.9569	0.9161	0.9775	0.2388
S3_21363717	3	21363717	S3_21363890	2	0.173	0.8767	0.9565	0.9145	0.9769	0.2388
S3_86977	3	86977	S3_127260	2	40.283	0.8765	0.9668	0.9369	0.9996	0.1672

S3_490863	3	490863	S3_492484	2	1.621	0.8755	0.9729	0.9355	1.0000	0.2381
S3_3638411	3	3638411	S3_3639874	1	1.463	0.8741	0.9555	0.9345	0.9880	0.2415
S3_7732321	3	7732321	S3_7733662	2	1.341	0.8740	0.9770	0.9122	0.9981	0.2332
S3_18667313	3	18667313	S3_18708739	5	41.426	0.8732	0.9551	0.9319	0.9867	0.1158
S3_25180508	3	25180508	S3_25182611	1	2.103	0.8723	1.0000	0.8986	1.0000	0.1671
S3_18148177	3	18148177	S3_18148303	1	0.126	0.8698	0.9453	0.9299	0.9774	0.1943
S3_8788140	3	8788140	S3_8788261	1	0.121	0.8674	0.9710	0.9019	0.9901	0.1817
S3_199457	3	199457	S3_199559	1	0.102	0.8651	0.9751	0.9042	0.9970	0.2337
S3_26382658	3	26382658	S3_26382663	1	0.005	0.8558	1.0000	0.8380	0.9896	0.0683
S3_930588	3	930588	S3_940282	1	9.694	0.8522	0.9333	0.9177	0.9685	0.2275
S3_20347799	3	20347799	S3_20347833	1	0.034	0.8503	0.9721	0.9199	1.0000	0.1953
S3_3382809	3	3382809	S3_3389136	1	6.327	0.8493	0.9460	0.9160	0.9825	0.1984
S3_2792335	3	2792335	S3_2792348	1	0.013	0.8483	1.0000	0.8677	1.0000	0.1767
S3_8774897	3	8774897	S3_8788140	2	13.243	0.8473	0.9453	0.8915	0.9696	0.1829
S3_17934744	3	17934744	S3_17954071	1	19.327	0.8425	1.0000	0.9159	1.0000	0.0863
S3_23314139	3	23314139	S3_23314140	1	0.001	0.8424	0.9178	0.8368	0.9148	0.1173
S3_21041029	3	21041029	S3_21046923	2	5.894	0.8371	0.9414	0.8614	0.9550	0.2036
S3_19348148	3	19348148	S3_19352715	2	4.567	0.8369	1.0000	0.8299	0.9958	0.0527
S3_573779	3	573779	S3_573838	1	0.059	0.8349	0.9137	0.9101	0.9540	0.2381
S3_26484221	3	26484221	S3_26537286	3	53.065	0.8346	0.9404	0.9110	0.9825	0.0959
S3_25562363	3	25562363	S3_25566903	1	4.54	0.8222	1.0000	0.9036	1.0000	0.0606
S3_9376259	3	9376259	S3_9377029	4	0.77	0.8079	1.0000	0.9015	1.0000	0.1371
S3_23314138	3	23314138	S3_23314139	1	0.001	0.8076	0.9169	0.8005	0.9128	0.1163
S3_7084179	3	7084179	S3_7084232	1	0.053	0.7975	1.0000	0.8253	1.0000	0.2177
S3_25329232	3	25329232	S3_25337337	1	8.105	0.7963	0.9424	0.8442	0.9703	0.1014
S3_6306421	3	6306421	S3_6312154	1	5.733	0.7956	0.9602	0.8217	0.9759	0.1575
S3_26247448	3	26247448	S3_26247472	1	0.024	0.7696	1.0000	0.7473	0.9854	0.0757
S3_8980131	3	8980131	S3_8997780	1	17.649	0.7623	1.0000	0.7760	1.0000	0.1051
S3_7084232	3	7084232	S3_7084320	1	0.088	0.7594	0.9429	0.7966	0.9657	0.2147
S3_22657440	3	22657440	S3_22659334	1	1.894	0.7585	0.9299	0.7764	0.9408	0.1386

S3_24899482	3	24899482	S3_24920422	1	20.94	0.7544	0.8917	0.7688	0.9002	0.0905
S3_21468859	3	21468859	S3_21468887	1	0.028	0.7505	1.0000	0.7450	0.9964	0.0937
S3_219381	3	219381	S3_223975	2	4.594	0.7410	0.8914	0.7920	0.9216	0.1554
S3_22670125	3	22670125	S3_22700253	1	30.128	0.7336	1.0000	0.7289	0.9968	0.1156
S3_20509769	3	20509769	S3_20509772	1	0.003	0.7333	1.0000	0.6649	0.9522	0.0546
S3_24799999	3	24799999	S3_24800655	2	0.656	0.7271	0.8527	0.7178	0.8473	0.0720
S3_24228511	3	24228511	S3_24228583	1	0.072	0.7252	0.9507	0.7775	0.9844	0.1063
S3_1015252	3	1015252	S3_1100863	1	85.611	0.7242	0.8510	0.7565	0.8698	0.0943
S3_25337337	3	25337337	S3_25384667	1	47.33	0.7233	0.9428	0.8044	0.9943	0.0914
S3_24876003	3	24876003	S3_24899414	1	23.411	0.7180	0.8920	0.7564	0.9155	0.0873
S3_25384667	3	25384667	S3_25415121	1	30.454	0.7130	0.8444	0.7520	0.8672	0.0939
S3_26379387	3	26379387	S3_26382658	1	3.271	0.7110	1.0000	0.7492	1.0000	0.0534
S3_23756796	3	23756796	S3_23772511	1	15.715	0.7028	0.8763	0.7545	0.9079	0.1987
S3_22161054	3	22161054	S3_22161060	1	0.006	0.6863	1.0000	0.7086	1.0000	0.1419
S3_23772615	3	23772615	S3_23772832	1	0.217	0.6826	1.0000	0.6963	1.0000	0.1609
S3_20751801	3	20751801	S3_20771687	5	19.886	0.6781	0.9107	0.7202	0.9386	0.1119
S3_22648051	3	22648051	S3_22657440	1	9.389	0.6631	0.8282	0.6699	0.8324	0.1250
S3_25329043	3	25329043	S3_25329232	1	0.189	0.6499	0.9324	0.6718	0.9481	0.0825
S3_20481508	3	20481508	S3_20509769	2	28.261	0.6499	0.9092	0.7870	1.0000	0.0593
S3_17954105	3	17954105	S3_17954112	1	0.007	0.6487	1.0000	0.6410	0.9941	0.1703
S3_24797760	3	24797760	S3_24799999	1	2.239	0.5860	0.8285	0.6354	0.8628	0.0689
S3_17954113	3	17954113	S3_17954141	1	0.028	0.5561	0.9382	0.5874	0.9643	0.1620
S3_23772511	3	23772511	S3_23772615	1	0.104	0.5294	1.0000	0.5596	1.0000	0.1502
S3_26425597	3	26425597	S3_26425614	1	0.017	0.4562	1.0000	0.5057	1.0000	0.0874
S3_25281079	3	25281079	S3_25329043	3	47.964	0.4420	0.8548	0.5101	0.9183	0.0703
S3_20875878	3	20875878	S3_20875947	1	0.069	0.3068	1.0000	0.3277	1.0000	0.0802
S3_20875947	3	20875947	S3_20876046	1	0.099	0.3068	1.0000	0.3277	1.0000	0.0802
S3_26194899	3	26194899	S3_26199867	1	4.968	0.0729	0.9997	0.0676	0.9622	-0.0383
S3_26194800	3	26194800	S3_26194899	1	0.099	0.0725	1.0000	0.0768	1.0000	-0.0406
S4_1890187	4	1890187	S4_1890201	1	0.014	1.0000	1.0000	1.0000	1.0000	0.2041

S4_1890201	4	1890201	S4_1890203	1	0.002	1.0000	1.0000	1.0000	1.0000	0.2041
S4_1890203	4	1890203	S4_1890205	1	0.002	1.0000	1.0000	1.0000	1.0000	0.2041
S4_2674636	4	2674636	S4_2674903	2	0.267	1.0000	1.0000	1.0000	1.0000	0.1060
S4_2993903	4	2993903	S4_2993999	1	0.096	1.0000	1.0000	1.0000	1.0000	0.2256
S4_6328212	4	6328212	S4_6328233	1	0.021	1.0000	1.0000	1.0000	1.0000	0.0942
S4_18188800	4	18188800	S4_18189082	1	0.282	1.0000	1.0000	1.0000	1.0000	0.2025
S4_21793447	4	21793447	S4_21795647	1	2.2	1.0000	1.0000	1.0000	1.0000	0.0842
S4_26225700	4	26225700	S4_26225732	1	0.032	1.0000	1.0000	1.0000	1.0000	0.2489
S4_27443946	4	27443946	S4_27443950	1	0.004	1.0000	1.0000	1.0000	1.0000	0.2493
S4_29169317	4	29169317	S4_29169354	1	0.037	1.0000	1.0000	1.0000	1.0000	0.2070
S4_29931057	4	29931057	S4_29931098	1	0.041	1.0000	1.0000	1.0000	1.0000	0.0542
S4_23350453	4	23350453	S4_23354401	1	3.948	1.0000	1.0000	1.0000	1.0000	0.0790
S4_197447	4	197447	S4_197448	1	0.001	1.0000	1.0000	1.0000	1.0000	0.2495
S4_12395788	4	12395788	S4_12395789	1	0.001	1.0000	1.0000	1.0000	1.0000	0.2143
S4_12395789	4	12395789	S4_12395790	1	0.001	1.0000	1.0000	1.0000	1.0000	0.2143
S4_10109682	4	10109682	S4_10109705	1	0.023	1.0000	1.0000	1.0000	1.0000	0.2500
S4_10109705	4	10109705	S4_10109718	1	0.013	1.0000	1.0000	1.0000	1.0000	0.2500
S4_1890280	4	1890280	S4_1890311	1	0.031	1.0000	1.0000	1.0000	1.0000	0.2064
S4_23667854	4	23667854	S4_23667866	1	0.012	1.0000	1.0000	1.0000	1.0000	0.2216
S4_27006205	4	27006205	S4_27006214	1	0.009	1.0000	1.0000	1.0000	1.0000	0.2052
S4_3112068	4	3112068	S4_3112086	1	0.018	1.0000	1.0000	1.0000	1.0000	0.1593
S4_3112086	4	3112086	S4_3112096	1	0.01	1.0000	1.0000	1.0000	1.0000	0.1593
S4_29396505	4	29396505	S4_29396507	1	0.002	1.0000	1.0000	1.0000	1.0000	0.1438
S4_29396507	4	29396507	S4_29396509	1	0.002	1.0000	1.0000	1.0000	1.0000	0.1438
S4_3109935	4	3109935	S4_3109945	1	0.01	1.0000	1.0000	1.0000	1.0000	0.1456
S4_3109945	4	3109945	S4_3109946	1	0.001	1.0000	1.0000	1.0000	1.0000	0.1456
S4_3109946	4	3109946	S4_3109950	1	0.004	1.0000	1.0000	1.0000	1.0000	0.1456
S4_10413307	4	10413307	S4_10418823	1	5.516	1.0000	1.0000	1.0000	1.0000	0.1323
S4_16709900	4	16709900	S4_16709901	1	0.001	1.0000	1.0000	1.0000	1.0000	0.1045
S4_10355843	4	10355843	S4_10413307	1	57.464	1.0000	1.0000	1.0000	1.0000	0.1104

S4_26882612	4	26882612	S4_26882617	1	0.005	1.0000	1.0000	1.0000	1.0000	0.1218
S4_26882617	4	26882617	S4_26882619	1	0.002	1.0000	1.0000	1.0000	1.0000	0.1218
S4_3569524	4	3569524	S4_3570964	3	1.44	1.0000	1.0000	1.0000	1.0000	0.0993
S4_2685273	4	2685273	S4_2685276	1	0.003	1.0000	1.0000	1.0000	1.0000	0.0882
S4_28587506	4	28587506	S4_28587519	1	0.013	1.0000	1.0000	1.0000	1.0000	0.0713
S4_26737767	4	26737767	S4_26737768	1	0.001	1.0000	1.0000	1.0000	1.0000	0.2497
S4_2266748	4	2266748	S4_2266810	1	0.062	1.0000	1.0000	1.0000	1.0000	0.0631
S4_5238915	4	5238915	S4_5238939	1	0.024	1.0000	1.0000	1.0000	1.0000	0.0637
S4_1366284	4	1366284	S4_1366314	1	0.03	1.0000	1.0000	1.0000	1.0000	0.1988
S4_15428654	4	15428654	S4_15428672	1	0.018	1.0000	1.0000	1.0000	1.0000	0.1662
S4_25879325	4	25879325	S4_25879412	1	0.087	1.0000	1.0000	1.0000	1.0000	0.1457
S4_3112206	4	3112206	S4_3112230	1	0.024	1.0000	1.0000	1.0000	1.0000	0.1389
S4_29936595	4	29936595	S4_29936631	3	0.036	1.0000	1.0000	1.0000	1.0000	0.0485
S4_8183349	4	8183349	S4_8183390	1	0.041	1.0000	1.0000	1.0000	1.0000	0.1412
S4_21772440	4	21772440	S4_21772446	1	0.006	1.0000	1.0000	1.0000	1.0000	0.1780
S4_2371418	4	2371418	S4_2371458	1	0.04	1.0000	1.0000	1.0000	1.0000	0.1307
S4_2371313	4	2371313	S4_2371388	1	0.075	1.0000	1.0000	1.0000	1.0000	0.1318
S4_2371388	4	2371388	S4_2371418	1	0.03	1.0000	1.0000	1.0000	1.0000	0.1318
S4_24787325	4	24787325	S4_24787327	1	0.002	1.0000	1.0000	1.0000	1.0000	0.1318
S4_25400331	4	25400331	S4_25400332	1	0.001	1.0000	1.0000	1.0000	1.0000	0.2414
S4_27393696	4	27393696	S4_27393732	1	0.036	1.0000	1.0000	1.0000	1.0000	0.1192
S4_7728100	4	7728100	S4_7729036	1	0.936	1.0000	1.0000	1.0000	1.0000	0.1523
S4_26506595	4	26506595	S4_26506621	1	0.026	1.0000	1.0000	1.0000	1.0000	0.1005
S4_409828	4	409828	S4_409833	1	0.005	1.0000	1.0000	1.0000	1.0000	0.1821
S4_15070024	4	15070024	S4_15070026	1	0.002	1.0000	1.0000	1.0000	1.0000	0.1667
S4_3920450	4	3920450	S4_3920460	1	0.01	1.0000	1.0000	1.0000	1.0000	0.1071
S4_3920460	4	3920460	S4_3920498	1	0.038	1.0000	1.0000	1.0000	1.0000	0.1071
S4_9447090	4	9447090	S4_9447131	1	0.041	1.0000	1.0000	1.0000	1.0000	0.2440
S4_26440953	4	26440953	S4_26440954	1	0.001	1.0000	1.0000	1.0000	1.0000	0.1479
S4_26440954	4	26440954	S4_26440971	1	0.017	1.0000	1.0000	1.0000	1.0000	0.1479

S4_24837403	4	24837403	S4_24837404	1	0.001	1.0000	1.0000	1.0000	1.0000	0.1515
S4_3512527	4	3512527	S4_3512532	1	0.005	1.0000	1.0000	1.0000	1.0000	0.2334
S4_3291382	4	3291382	S4_3291413	1	0.031	1.0000	1.0000	1.0000	1.0000	0.0670
S4_19181030	4	19181030	S4_19181045	1	0.015	1.0000	1.0000	1.0000	1.0000	0.1413
S4_3609668	4	3609668	S4_3609685	1	0.017	1.0000	1.0000	1.0000	1.0000	0.2009
S4_29825020	4	29825020	S4_29825031	1	0.011	1.0000	1.0000	1.0000	1.0000	0.1926
S4_19281669	4	19281669	S4_19281671	1	0.002	1.0000	1.0000	1.0000	1.0000	0.1533
S4_19281671	4	19281671	S4_19281673	1	0.002	1.0000	1.0000	1.0000	1.0000	0.1533
S4_19281673	4	19281673	S4_19281674	1	0.001	1.0000	1.0000	1.0000	1.0000	0.1533
S4_15211104	4	15211104	S4_15211116	1	0.012	1.0000	1.0000	1.0000	1.0000	0.1901
S4_19281547	4	19281547	S4_19281669	1	0.122	1.0000	1.0000	1.0000	1.0000	0.1624
S4_2462144	4	2462144	S4_2462146	1	0.002	1.0000	1.0000	1.0000	1.0000	0.1766
S4_22651310	4	22651310	S4_22665918	1	14.608	1.0000	1.0000	1.0000	1.0000	0.2496
S4_24908086	4	24908086	S4_24908092	1	0.006	1.0000	1.0000	1.0000	1.0000	0.2447
S4_28071694	4	28071694	S4_28071695	1	0.001	1.0000	1.0000	1.0000	1.0000	0.2330
S4_22807328	4	22807328	S4_22807348	1	0.02	1.0000	1.0000	1.0000	1.0000	0.2116
S4_7820582	4	7820582	S4_7820594	1	0.012	1.0000	1.0000	1.0000	1.0000	0.2500
S4_2584947	4	2584947	S4_2584950	2	0.003	1.0000	1.0000	1.0000	1.0000	0.0568
S4_27393453	4	27393453	S4_27393483	1	0.03	1.0000	1.0000	1.0000	1.0000	0.1307
S4_27393483	4	27393483	S4_27393486	1	0.003	1.0000	1.0000	1.0000	1.0000	0.1307
S4_3766272	4	3766272	S4_3766273	1	0.001	1.0000	1.0000	1.0000	1.0000	0.1782
S4_3766273	4	3766273	S4_3766274	1	0.001	1.0000	1.0000	1.0000	1.0000	0.1782
S4_26594218	4	26594218	S4_26594269	2	0.051	1.0000	1.0000	1.0000	1.0000	0.2161
S4_21962757	4	21962757	S4_21962758	1	0.001	1.0000	1.0000	1.0000	1.0000	0.2236
S4_21962758	4	21962758	S4_21962759	1	0.001	1.0000	1.0000	1.0000	1.0000	0.2236
S4_7821342	4	7821342	S4_7821356	1	0.014	1.0000	1.0000	1.0000	1.0000	0.2499
S4_2584951	4	2584951	S4_2584952	1	0.001	1.0000	1.0000	1.0000	1.0000	0.0756
S4_23667972	4	23667972	S4_23667973	1	0.001	1.0000	1.0000	1.0000	1.0000	0.0974
S4_23667973	4	23667973	S4_23667975	1	0.002	1.0000	1.0000	1.0000	1.0000	0.0974
S4_27936272	4	27936272	S4_27936276	1	0.004	1.0000	1.0000	1.0000	1.0000	0.0670

S4_28549093	4	28549093	S4_28549095	1	0.002	1.0000	1.0000	1.0000	1.0000	0.2186
S4_3442460	4	3442460	S4_3442596	3	0.136	0.9795	1.0000	0.9892	1.0000	0.2481
S4_28071695	4	28071695	S4_28071842	2	0.147	0.9778	1.0000	0.9884	1.0000	0.2310
S4_2487658	4	2487658	S4_2487665	1	0.007	0.9770	1.0000	0.9876	1.0000	0.2205
S4_1968899	4	1968899	S4_1969028	1	0.129	0.9760	1.0000	0.9879	1.0000	0.2157
S4_29343261	4	29343261	S4_29343710	2	0.449	0.9754	1.0000	0.9868	1.0000	0.2096
S4_23990650	4	23990650	S4_23990727	2	0.077	0.9752	1.0000	0.9873	1.0000	0.2199
S4_1366314	4	1366314	S4_1366319	1	0.005	0.9741	1.0000	0.9866	1.0000	0.1973
S4_1366319	4	1366319	S4_1366359	1	0.04	0.9739	1.0000	0.9863	1.0000	0.1936
S4_13224671	4	13224671	S4_13224850	1	0.179	0.9736	1.0000	0.9863	1.0000	0.2474
S4_24837365	4	24837365	S4_24837403	1	0.038	0.9651	1.0000	0.9817	1.0000	0.1484
S4_2876742	4	2876742	S4_2876769	1	0.027	0.9637	1.0000	0.9809	1.0000	0.1381
S4_8894636	4	8894636	S4_8958274	1	63.638	0.9634	1.0000	0.9808	1.0000	0.1428
S4_8958274	4	8958274	S4_8961505	2	3.231	0.9628	1.0000	0.9808	1.0000	0.1347
S4_24787327	4	24787327	S4_24802699	1	15.372	0.9617	1.0000	0.9807	1.0000	0.1334
S4_27753116	4	27753116	S4_27753190	1	0.074	0.9615	1.0000	0.9788	1.0000	0.1368
S4_25762458	4	25762458	S4_25762511	2	0.053	0.9594	1.0000	0.9793	1.0000	0.2464
S4_10109718	4	10109718	S4_10220204	1	110.486	0.9583	1.0000	0.9789	1.0000	-0.2473
S4_8027535	4	8027535	S4_8027676	1	0.141	0.9583	1.0000	0.9779	1.0000	0.1433
S4_23639365	4	23639365	S4_23639401	1	0.036	0.9583	0.9789	0.9782	0.9891	0.2468
S4_18666667	4	18666667	S4_18711254	3	44.587	0.9578	1.0000	0.9786	1.0000	0.2468
S4_16177707	4	16177707	S4_16327097	6	149.39	0.9575	1.0000	0.9791	1.0000	0.1341
S4_1304938	4	1304938	S4_1304948	1	0.01	0.9524	1.0000	0.9507	0.9991	0.2106
S4_11001241	4	11001241	S4_11003110	2	1.869	0.9519	0.9757	0.9744	0.9871	0.2205
S4_1891841	4	1891841	S4_1904091	2	12.25	0.9499	0.9746	0.9743	0.9871	0.2005
S4_29993169	4	29993169	S4_30004572	5	11.403	0.9488	1.0000	0.9717	1.0000	0.0998
S4_1890311	4	1890311	S4_1891841	1	1.53	0.9475	0.9734	0.9730	0.9864	0.2036
S4_29571735	4	29571735	S4_29571857	1	0.122	0.9467	0.9730	0.9726	0.9862	0.2172
S4_30048541	4	30048541	S4_30074906	1	26.365	0.9465	1.0000	0.9711	1.0000	0.0985
S4_5670570	4	5670570	S4_5671120	2	0.55	0.9458	0.9725	0.9715	0.9857	0.2015

S4_3920498	4	3920498	S4_3925413	1	4.915	0.9455	1.0000	0.9726	1.0000	0.1113
S4_10418823	4	10418823	S4_10438223	1	19.4	0.9451	1.0000	0.9724	1.0000	0.1163
S4_2217965	4	2217965	S4_2217975	1	0.01	0.9450	1.0000	0.9450	1.0000	0.1809
S4_2179165	4	2179165	S4_2190875	1	11.71	0.9386	1.0000	0.9675	1.0000	0.0872
S4_2674903	4	2674903	S4_2674978	1	0.075	0.9372	1.0000	0.9694	1.0000	0.1026
S4_14504373	4	14504373	S4_14506375	1	2.002	0.9354	0.9778	0.9655	0.9934	0.2412
S4_18951366	4	18951366	S4_18962106	1	10.74	0.9340	0.9773	0.9659	0.9939	0.2435
S4_27752996	4	27752996	S4_27753116	1	0.12	0.9325	1.0000	0.9629	1.0000	0.1462
S4_2991022	4	2991022	S4_2993891	2	2.869	0.9307	1.0000	0.9419	1.0000	0.2357
S4_28769353	4	28769353	S4_28772328	1	2.975	0.9279	0.9633	0.9626	0.9811	0.1692
S4_3154882	4	3154882	S4_3154974	1	0.092	0.9266	0.9626	0.9626	0.9811	0.1351
S4_29250258	4	29250258	S4_29254417	1	4.159	0.9250	0.9618	0.9603	0.9799	0.1458
S4_26194584	4	26194584	S4_26210438	4	15.854	0.9235	0.9736	0.9595	0.9924	0.2444
S4_27511601	4	27511601	S4_27532372	1	20.771	0.9231	0.9734	0.9318	0.9780	0.2247
S4_27902579	4	27902579	S4_27903126	1	0.547	0.9224	0.9732	0.9591	0.9924	0.2080
S4_23972458	4	23972458	S4_23972579	1	0.121	0.9216	1.0000	0.9622	1.0000	0.0823
S4_12132824	4	12132824	S4_12132992	1	0.168	0.9199	1.0000	0.9560	1.0000	0.1207
S4_26440928	4	26440928	S4_26440929	1	0.001	0.9186	1.0000	0.8834	0.9806	0.1712
S4_1890205	4	1890205	S4_1890280	1	0.075	0.9182	0.9717	0.9311	0.9785	0.1988
S4_2755375	4	2755375	S4_2755490	1	0.115	0.9177	1.0000	0.9573	1.0000	0.0619
S4_27393486	4	27393486	S4_27393696	1	0.21	0.9171	1.0000	0.9553	1.0000	0.1201
S4_10595594	4	10595594	S4_10595753	2	0.159	0.9155	0.9568	0.9343	0.9666	0.2356
S4_9446976	4	9446976	S4_9447070	1	0.094	0.9120	1.0000	0.9079	0.9978	0.1088
S4_409833	4	409833	S4_413001	1	3.168	0.9119	0.9694	0.9237	0.9757	0.1735
S4_11003110	4	11003110	S4_11003137	1	0.027	0.9105	1.0000	0.9280	1.0000	0.2139
S4_27370604	4	27370604	S4_27371889	1	1.285	0.9098	0.9538	0.9538	0.9767	0.1356
S4_22164780	4	22164780	S4_22233620	5	68.84	0.9042	1.0000	0.9499	1.0000	0.0498
S4_2470123	4	2470123	S4_2470127	2	0.004	0.9033	1.0000	0.9145	1.0000	0.0566
S4_1249850	4	1249850	S4_1249874	1	0.024	0.9027	1.0000	0.9015	0.9993	0.1951
S4_26693052	4	26693052	S4_26705555	1	12.503	0.9020	0.9658	0.9483	0.9903	0.1706

S4_12051026	4	12051026	S4_12064649	1	13.623	0.9015	0.9656	0.9097	0.9700	0.1457
S4_25572569	4	25572569	S4_25572695	1	0.126	0.9006	1.0000	0.9142	1.0000	0.1611
S4_1849326	4	1849326	S4_1890187	3	40.861	0.8947	0.9720	0.9201	0.9857	0.1934
S4_29927912	4	29927912	S4_29930626	2	2.714	0.8939	1.0000	0.9410	1.0000	0.0554
S4_2642795	4	2642795	S4_2654489	1	11.694	0.8935	1.0000	0.9066	1.0000	0.1546
S4_28468113	4	28468113	S4_28472508	1	4.395	0.8911	1.0000	0.8971	1.0000	0.2116
S4_3112096	4	3112096	S4_3112206	1	0.11	0.8902	1.0000	0.9452	1.0000	0.1440
S4_27942244	4	27942244	S4_27942375	1	0.131	0.8899	0.9614	0.9419	0.9891	0.1621
S4_30004572	4	30004572	S4_30048541	2	43.969	0.8886	1.0000	0.9380	1.0000	0.0931
S4_29847180	4	29847180	S4_29856813	1	9.633	0.8871	0.9529	0.9399	0.9808	0.2424
S4_27744942	4	27744942	S4_27745019	1	0.077	0.8866	0.9602	0.9396	0.9884	0.1306
S4_2739828	4	2739828	S4_2739845	1	0.017	0.8838	1.0000	0.8996	1.0000	0.1187
S4_5671120	4	5671120	S4_5757215	9	86.095	0.8838	1.0000	0.8892	1.0000	0.2007
S4_25852541	4	25852541	S4_25852620	1	0.079	0.8834	1.0000	0.8992	1.0000	0.1207
S4_29931098	4	29931098	S4_29936595	1	5.497	0.8830	1.0000	0.9383	1.0000	0.0486
S4_29930626	4	29930626	S4_29931057	1	0.431	0.8823	1.0000	0.9379	1.0000	0.0544
S4_2947491	4	2947491	S4_2947554	2	0.063	0.8816	1.0000	0.8899	1.0000	0.1969
S4_19281503	4	19281503	S4_19281547	3	0.044	0.8712	1.0000	0.8958	1.0000	0.1596
S4_27006001	4	27006001	S4_27006205	1	0.204	0.8699	1.0000	0.9332	1.0000	0.2037
S4_3137672	4	3137672	S4_3154882	1	17.21	0.8667	1.0000	0.8968	1.0000	0.1378
S4_27510607	4	27510607	S4_27511513	1	0.906	0.8666	0.9439	0.9031	0.9635	0.2121
S4_1304948	4	1304948	S4_1306145	1	1.197	0.8616	1.0000	0.9049	1.0000	0.2014
S4_11852354	4	11852354	S4_11852356	1	0.002	0.8599	1.0000	0.8963	1.0000	0.1934
S4_18189082	4	18189082	S4_18215664	5	26.582	0.8597	1.0000	0.8597	1.0000	0.1956
S4_7777402	4	7777402	S4_7820582	1	43.18	0.8580	0.9401	0.9229	0.9749	0.2395
S4_2674978	4	2674978	S4_2677147	1	2.169	0.8576	0.9493	0.9247	0.9857	0.1157
S4_29240303	4	29240303	S4_29250227	2	9.924	0.8571	0.9491	0.9013	0.9733	0.2360
S4_29396509	4	29396509	S4_29397705	1	1.196	0.8543	0.9603	0.8880	0.9790	0.1401
S4_3120686	4	3120686	S4_3120687	1	0.001	0.8476	1.0000	0.8601	1.0000	0.0584
S4_23258067	4	23258067	S4_23258166	1	0.099	0.8467	1.0000	0.9179	1.0000	0.0653

S4_8103264	4	8103264	S4_8183349	2	80.085	0.8419	1.0000	0.8352	0.9960	0.1278
S4_27976105	4	27976105	S4_27983873	2	7.768	0.8410	0.9429	0.9108	0.9812	0.1012
S4_2739845	4	2739845	S4_2747820	2	7.975	0.8404	0.9561	0.8736	0.9748	0.1194
S4_28549025	4	28549025	S4_28549093	1	0.068	0.8396	0.9499	0.9085	0.9882	0.2086
S4_27393732	4	27393732	S4_27411015	1	17.283	0.8351	0.9545	0.8663	0.9721	0.1175
S4_1245870	4	1245870	S4_1249850	1	3.98	0.8293	0.9465	0.8621	0.9650	0.1899
S4_29166209	4	29166209	S4_29169317	1	3.108	0.8255	0.9205	0.8746	0.9474	0.1949
S4_20348457	4	20348457	S4_20348478	1	0.021	0.8246	1.0000	0.8191	0.9967	0.1449
S4_2202204	4	2202204	S4_2217965	2	15.761	0.8167	0.9421	0.8279	0.9486	0.1801
S4_2459203	4	2459203	S4_2462144	1	2.941	0.8135	0.9320	0.8630	0.9600	0.1770
S4_12859048	4	12859048	S4_12938785	1	79.737	0.8125	0.9475	0.8511	0.9698	0.1027
S4_2371458	4	2371458	S4_2389707	2	18.249	0.8079	0.9561	0.8188	0.9625	0.1132
S4_12850189	4	12850189	S4_12859048	2	8.859	0.8043	0.9449	0.8481	0.9703	0.1036
S4_3154974	4	3154974	S4_3162514	1	7.54	0.8013	0.9615	0.8296	0.9784	0.1325
S4_28113058	4	28113058	S4_28113097	1	0.039	0.7945	1.0000	0.8381	1.0000	0.1018
S4_3112230	4	3112230	S4_3117460	1	5.23	0.7936	0.9241	0.8230	0.9410	0.1291
S4_26975999	4	26975999	S4_26976036	1	0.037	0.7888	1.0000	0.8078	1.0000	0.1169
S4_27725896	4	27725896	S4_27744942	8	19.046	0.7847	0.9063	0.8412	0.9384	0.1125
S4_9447533	4	9447533	S4_9448365	2	0.832	0.7814	1.0000	0.7996	1.0000	0.1256
S4_4883769	4	4883769	S4_4932562	2	48.793	0.7800	1.0000	0.7800	1.0000	0.0806
S4_9378114	4	9378114	S4_9441138	1	63.024	0.7748	0.9554	0.8076	0.9755	0.1500
S4_12406687	4	12406687	S4_12406775	1	0.088	0.7721	1.0000	0.7912	1.0000	0.1240
S4_24393074	4	24393074	S4_24407032	5	13.958	0.7649	0.8746	0.8161	0.9034	0.0912
S4_29402195	4	29402195	S4_29402488	1	0.293	0.7649	1.0000	0.7713	1.0000	0.1347
S4_3540910	4	3540910	S4_3569523	1	28.613	0.7561	0.9509	0.7895	0.9717	0.1255
S4_29397705	4	29397705	S4_29397890	2	0.185	0.7551	0.9197	0.7970	0.9449	0.1355
S4_27983873	4	27983873	S4_27983967	1	0.094	0.7544	0.8917	0.8606	0.9524	0.0957
S4_8183390	4	8183390	S4_8207976	3	24.586	0.7380	0.8765	0.7810	0.9017	0.1179
S4_24733298	4	24733298	S4_24787325	2	54.027	0.7379	0.9589	0.8199	1.0000	0.1283
S4_19186319	4	19186319	S4_19186353	1	0.034	0.7325	1.0000	0.7422	1.0000	0.1072

S4_12195979	4	12195979	S4_12197454	2	1.475	0.7274	0.9091	0.7711	0.9360	0.1085
S4_2584950	4	2584950	S4_2584951	1	0.001	0.7161	1.0000	0.6967	0.9863	0.0547
S4_14412069	4	14412069	S4_14412077	1	0.008	0.7135	1.0000	0.6823	0.9779	0.0769
S4_229167	4	229167	S4_239354	1	10.187	0.7131	1.0000	0.7340	1.0000	0.1341
S4_25773640	4	25773640	S4_25823403	2	49.763	0.7119	0.9137	0.7710	0.9508	0.1343
S4_29402115	4	29402115	S4_29402195	2	0.08	0.7062	0.9272	0.7411	0.9498	0.1351
S4_27975991	4	27975991	S4_27976105	1	0.114	0.7044	0.8668	0.7774	0.9106	0.0888
S4_12132992	4	12132992	S4_12195979	1	62.987	0.6940	0.8687	0.7513	0.9039	0.1088
S4_239354	4	239354	S4_274146	2	34.792	0.6917	1.0000	0.7226	1.0000	0.1342
S4_3217031	4	3217031	S4_3247166	2	30.135	0.6822	0.9317	0.7232	0.9593	0.0915
S4_2677353	4	2677353	S4_2685273	3	7.92	0.6763	1.0000	0.6854	1.0000	0.0856
S4_2642792	4	2642792	S4_2642795	1	0.003	0.6398	1.0000	0.6633	1.0000	0.1089
S4_2179040	4	2179040	S4_2179165	1	0.125	0.6353	0.9364	0.6634	0.9570	0.0778
S4_29564946	4	29564946	S4_29566910	1	1.964	0.6301	0.9417	0.6703	0.9712	0.0869
S4_27745019	4	27745019	S4_27752996	1	7.977	0.5899	0.8408	0.6656	0.8932	0.1172
S4_3410544	4	3410544	S4_3410612	1	0.068	0.5871	0.8740	0.5459	0.8428	0.0736
S4_10595753	4	10595753	S4_10730981	1	135.228	0.5805	0.9715	0.6335	1.0000	0.1839
S4_14412068	4	14412068	S4_14412069	1	0.001	0.5782	0.8227	0.5370	0.7929	0.0734
S4_22044681	4	22044681	S4_22044728	1	0.047	0.5588	0.7863	0.7491	0.9105	0.0480
S4_303003	4	303003	S4_328431	2	25.428	0.5450	0.9549	0.5885	0.9923	0.1164
S4_2654489	4	2654489	S4_2674636	3	20.147	0.5332	1.0000	0.5518	1.0000	0.1051
S4_25322990	4	25322990	S4_25387545	1	64.555	0.5245	0.9694	0.5754	1.0000	0.1755
S4_18142322	4	18142322	S4_18188800	1	46.478	0.5104	0.9614	0.5312	0.9808	0.1608
S4_29501518	4	29501518	S4_29510545	2	9.027	0.5065	0.8884	0.5701	0.9425	0.0586
S4_26480634	4	26480634	S4_26506595	2	25.961	0.4961	0.7400	0.5391	0.7714	0.0767
S4_29250227	4	29250227	S4_29250230	1	0.003	0.4446	1.0000	0.4551	1.0000	0.1472
S4_2755490	4	2755490	S4_2775010	1	19.52	0.4143	0.6437	0.4143	0.6437	0.0388
S4_29950541	4	29950541	S4_29950624	1	0.083	0.4041	1.0000	0.4363	1.0000	0.0472
S4_29950624	4	29950624	S4_29993169	5	42.545	0.4041	1.0000	0.4363	1.0000	0.0472
S4_3291413	4	3291413	S4_3313404	4	21.991	0.3470	0.6115	0.3719	0.6330	0.0421

S4_27371889	4	27371889	S4_27373852	1	1.963	0.3413	1.0000	0.3822	1.0000	0.0577
S4_27373852	4	27373852	S4_27393453	1	19.601	0.2909	0.9006	0.3469	0.9836	0.0532
S4_27490726	4	27490726	S4_27490735	1	0.009	0.2301	1.0000	0.2594	1.0000	0.0928
S5_231534	5	231534	S5_231590	1	0.056	1.0000	1.0000	1.0000	1.0000	0.2499
S5_1019194	5	1019194	S5_1019195	1	0.001	1.0000	1.0000	1.0000	1.0000	0.0656
S5_1019195	5	1019195	S5_1019196	1	0.001	1.0000	1.0000	1.0000	1.0000	0.0656
S5_1223471	5	1223471	S5_1223474	1	0.003	1.0000	1.0000	1.0000	1.0000	0.0489
S5_2910064	5	2910064	S5_2910108	1	0.044	1.0000	1.0000	1.0000	1.0000	0.0504
S5_6844686	5	6844686	S5_6849272	1	4.586	1.0000	1.0000	1.0000	1.0000	0.0604
S5_8638408	5	8638408	S5_8638419	1	0.011	1.0000	1.0000	1.0000	1.0000	0.0818
S5_8638419	5	8638419	S5_8638430	1	0.011	1.0000	1.0000	1.0000	1.0000	0.0818
S5_14089818	5	14089818	S5_14094433	1	4.615	1.0000	1.0000	1.0000	1.0000	0.1106
S5_14147256	5	14147256	S5_14221152	1	73.896	1.0000	1.0000	1.0000	1.0000	0.1307
S5_14221152	5	14221152	S5_14284899	1	63.747	1.0000	1.0000	1.0000	1.0000	0.1156
S5_14284899	5	14284899	S5_14284921	1	0.022	1.0000	1.0000	1.0000	1.0000	0.1156
S5_19851788	5	19851788	S5_19855371	2	3.583	1.0000	1.0000	1.0000	1.0000	0.0853
S5_27192719	5	27192719	S5_27192780	1	0.061	1.0000	1.0000	1.0000	1.0000	0.1334
S5_27192780	5	27192780	S5_27200659	1	7.879	1.0000	1.0000	1.0000	1.0000	0.1334
S5_27844881	5	27844881	S5_27844884	1	0.003	1.0000	1.0000	1.0000	1.0000	0.2440
S5_28022281	5	28022281	S5_28022309	1	0.028	1.0000	1.0000	1.0000	1.0000	0.0670
S5_2910108	5	2910108	S5_2910114	1	0.006	1.0000	1.0000	1.0000	1.0000	0.0499
S5_3750247	5	3750247	S5_3750398	1	0.151	1.0000	1.0000	1.0000	1.0000	0.0514
S5_19855402	5	19855402	S5_19855403	1	0.001	1.0000	1.0000	1.0000	1.0000	0.1202
S5_19855403	5	19855403	S5_19869862	1	14.459	1.0000	1.0000	1.0000	1.0000	0.1202
S5_15796591	5	15796591	S5_15796642	1	0.051	1.0000	1.0000	1.0000	1.0000	0.1114
S5_25765994	5	25765994	S5_25766006	1	0.012	1.0000	1.0000	1.0000	1.0000	0.2090
S5_25766006	5	25766006	S5_25766030	1	0.024	1.0000	1.0000	1.0000	1.0000	0.2090
S5_25766030	5	25766030	S5_25766037	1	0.007	1.0000	1.0000	1.0000	1.0000	0.2090
S5_4999060	5	4999060	S5_4999081	1	0.021	1.0000	1.0000	1.0000	1.0000	0.1479
S5_4999081	5	4999081	S5_4999132	2	0.051	1.0000	1.0000	1.0000	1.0000	0.1479

S5_1255888	5	1255888	S5_1255897	1	0.009	1.0000	1.0000	1.0000	1.0000	0.1343
S5_26271225	5	26271225	S5_26271226	1	0.001	1.0000	1.0000	1.0000	1.0000	0.1230
S5_1795426	5	1795426	S5_1795431	1	0.005	1.0000	1.0000	1.0000	1.0000	0.1336
S5_28325493	5	28325493	S5_28325528	1	0.035	1.0000	1.0000	1.0000	1.0000	0.1125
S5_1255897	5	1255897	S5_1259246	1	3.349	1.0000	1.0000	1.0000	1.0000	0.1305
S5_4101856	5	4101856	S5_4101905	1	0.049	1.0000	1.0000	1.0000	1.0000	0.0892
S5_26251525	5	26251525	S5_26251539	1	0.014	1.0000	1.0000	1.0000	1.0000	0.0892
S5_8638593	5	8638593	S5_8638638	1	0.045	1.0000	1.0000	1.0000	1.0000	0.0713
S5_8638638	5	8638638	S5_8638732	1	0.094	1.0000	1.0000	1.0000	1.0000	0.0713
S5_19789526	5	19789526	S5_19789643	1	0.117	1.0000	1.0000	1.0000	1.0000	0.0713
S5_19698064	5	19698064	S5_19698176	1	0.112	1.0000	1.0000	1.0000	1.0000	0.0780
S5_4794996	5	4794996	S5_4795028	1	0.032	1.0000	1.0000	1.0000	1.0000	0.2444
S5_8638532	5	8638532	S5_8638558	1	0.026	1.0000	1.0000	1.0000	1.0000	0.0677
S5_8638558	5	8638558	S5_8638593	1	0.035	1.0000	1.0000	1.0000	1.0000	0.0677
S5_14526119	5	14526119	S5_14526121	1	0.002	1.0000	1.0000	1.0000	1.0000	0.0535
S5_9371947	5	9371947	S5_9371960	1	0.013	1.0000	1.0000	1.0000	1.0000	0.0574
S5_1435189	5	1435189	S5_1435190	1	0.001	1.0000	1.0000	1.0000	1.0000	0.1931
S5_1435190	5	1435190	S5_1435198	1	0.008	1.0000	1.0000	1.0000	1.0000	0.1931
S5_605992	5	605992	S5_605993	1	0.001	1.0000	1.0000	1.0000	1.0000	0.1511
S5_605993	5	605993	S5_605996	1	0.003	1.0000	1.0000	1.0000	1.0000	0.1511
S5_23032658	5	23032658	S5_23032669	1	0.011	1.0000	1.0000	1.0000	1.0000	0.1933
S5_23032669	5	23032669	S5_23032682	1	0.013	1.0000	1.0000	1.0000	1.0000	0.1933
S5_23032682	5	23032682	S5_23032685	1	0.003	1.0000	1.0000	1.0000	1.0000	0.1933
S5_4209280	5	4209280	S5_4209532	1	0.252	1.0000	1.0000	1.0000	1.0000	0.1377
S5_28350172	5	28350172	S5_28350210	1	0.038	1.0000	1.0000	1.0000	1.0000	0.1246
S5_28181196	5	28181196	S5_28181199	1	0.003	1.0000	1.0000	1.0000	1.0000	0.0548
S5_28181199	5	28181199	S5_28181200	1	0.001	1.0000	1.0000	1.0000	1.0000	0.0548
S5_27110716	5	27110716	S5_27110718	1	0.002	1.0000	1.0000	1.0000	1.0000	0.0602
S5_26244626	5	26244626	S5_26244627	1	0.001	1.0000	1.0000	1.0000	1.0000	0.0969
S5_11028835	5	11028835	S5_11028836	1	0.001	1.0000	1.0000	1.0000	1.0000	0.0598

S5_11028836	5	11028836	S5_11028839	1	0.003	1.0000	1.0000	1.0000	1.0000	0.0598
S5_2007151	5	2007151	S5_2007183	1	0.032	1.0000	1.0000	1.0000	1.0000	0.1568
S5_641240	5	641240	S5_641266	1	0.026	1.0000	1.0000	1.0000	1.0000	0.1753
S5_1908137	5	1908137	S5_1908167	1	0.03	1.0000	1.0000	1.0000	1.0000	0.0835
S5_1702592	5	1702592	S5_1702607	1	0.015	1.0000	1.0000	1.0000	1.0000	0.2211
S5_27413999	5	27413999	S5_27414002	1	0.003	1.0000	1.0000	1.0000	1.0000	0.1990
S5_27414002	5	27414002	S5_27414003	1	0.001	1.0000	1.0000	1.0000	1.0000	0.1990
S5_844673	5	844673	S5_844686	1	0.013	1.0000	1.0000	1.0000	1.0000	0.2472
S5_4399456	5	4399456	S5_4399470	1	0.014	1.0000	1.0000	1.0000	1.0000	0.0489
S5_2952159	5	2952159	S5_2952186	1	0.027	1.0000	1.0000	1.0000	1.0000	0.1575
S5_26361514	5	26361514	S5_26361516	1	0.002	1.0000	1.0000	1.0000	1.0000	0.1967
S5_26603125	5	26603125	S5_26603128	1	0.003	1.0000	1.0000	1.0000	1.0000	0.1318
S5_26264580	5	26264580	S5_26264595	1	0.015	1.0000	1.0000	1.0000	1.0000	0.2499
S5_26194314	5	26194314	S5_26194316	1	0.002	1.0000	1.0000	1.0000	1.0000	0.2058
S5_12599779	5	12599779	S5_12599780	1	0.001	1.0000	1.0000	1.0000	1.0000	0.1951
S5_26591042	5	26591042	S5_26591064	1	0.022	1.0000	1.0000	1.0000	1.0000	0.1094
S5_2825669	5	2825669	S5_2825686	1	0.017	1.0000	1.0000	1.0000	1.0000	0.1447
S5_9548853	5	9548853	S5_9548888	1	0.035	1.0000	1.0000	1.0000	1.0000	0.0900
S5_7021827	5	7021827	S5_7021836	1	0.009	1.0000	1.0000	1.0000	1.0000	0.0540
S5_27759547	5	27759547	S5_27759548	1	0.001	1.0000	1.0000	1.0000	1.0000	0.2239
S5_26271367	5	26271367	S5_26271368	1	0.001	1.0000	1.0000	1.0000	1.0000	0.2461
S5_26271368	5	26271368	S5_26271369	1	0.001	1.0000	1.0000	1.0000	1.0000	0.2461
S5_23737584	5	23737584	S5_23737585	1	0.001	1.0000	1.0000	1.0000	1.0000	0.2404
S5_21290347	5	21290347	S5_21290348	1	0.001	1.0000	1.0000	1.0000	1.0000	0.1318
S5_26264697	5	26264697	S5_26264698	1	0.001	1.0000	1.0000	1.0000	1.0000	0.2500
S5_250141	5	250141	S5_250144	1	0.003	1.0000	1.0000	1.0000	1.0000	0.1494
S5_250144	5	250144	S5_250145	1	0.001	1.0000	1.0000	1.0000	1.0000	0.1494
S5_24167144	5	24167144	S5_24167150	1	0.006	1.0000	1.0000	1.0000	1.0000	0.1782
S5_26358941	5	26358941	S5_26358959	1	0.018	0.9788	1.0000	0.9888	1.0000	0.2396
S5_251810	5	251810	S5_252974	3	1.164	0.9753	1.0000	0.9858	1.0000	0.2049

S5_2797288	5	2797288	S5_2797300	1	0.012	0.9737	1.0000	0.9860	1.0000	0.2025
S5_27414003	5	27414003	S5_27414004	1	0.001	0.9735	1.0000	0.9662	0.9962	0.1968
S5_27414004	5	27414004	S5_27414005	1	0.001	0.9735	1.0000	0.9662	0.9962	0.1968
S5_15796670	5	15796670	S5_15797132	1	0.462	0.9718	1.0000	0.9856	1.0000	0.1928
S5_2858726	5	2858726	S5_2859581	3	0.855	0.9681	1.0000	0.9822	1.0000	0.1576
S5_20850943	5	20850943	S5_20863953	1	13.01	0.9679	1.0000	0.9833	1.0000	0.1602
S5_16955902	5	16955902	S5_16967685	1	11.783	0.9654	1.0000	0.9819	1.0000	0.1449
S5_16967685	5	16967685	S5_17000146	2	32.461	0.9654	1.0000	0.9819	1.0000	0.1449
S5_13279981	5	13279981	S5_13281675	1	1.694	0.9636	1.0000	0.9812	1.0000	0.1553
S5_12240278	5	12240278	S5_12240486	1	0.208	0.9633	1.0000	0.9818	1.0000	0.1840
S5_14299205	5	14299205	S5_14299259	1	0.054	0.9618	1.0000	0.9807	1.0000	0.1312
S5_1297362	5	1297362	S5_1315066	1	17.704	0.9589	1.0000	0.9792	1.0000	0.1355
S5_1197615	5	1197615	S5_1197966	2	0.351	0.9589	1.0000	0.9793	1.0000	0.1355
S5_23553223	5	23553223	S5_23553364	1	0.141	0.9587	1.0000	0.9784	1.0000	0.2468
S5_28350210	5	28350210	S5_28397992	3	47.782	0.9581	1.0000	0.9783	1.0000	0.1257
S5_26271226	5	26271226	S5_26271333	1	0.107	0.9569	1.0000	0.9784	1.0000	0.1208
S5_28075264	5	28075264	S5_28075504	1	0.24	0.9558	0.9777	0.9774	0.9886	0.2406
S5_1259246	5	1259246	S5_1260435	2	1.189	0.9545	1.0000	0.9763	1.0000	0.1310
S5_12875797	5	12875797	S5_12930620	2	54.823	0.9544	1.0000	0.9768	1.0000	0.1090
S5_1616110	5	1616110	S5_1623931	1	7.821	0.9542	1.0000	0.9767	1.0000	0.1110
S5_118870	5	118870	S5_125424	2	6.554	0.9535	1.0000	0.9757	1.0000	0.2159
S5_24977067	5	24977067	S5_24977176	1	0.109	0.9532	1.0000	0.9754	1.0000	0.2171
S5_15752467	5	15752467	S5_15754825	1	2.358	0.9524	1.0000	0.9758	1.0000	0.1350
S5_1435104	5	1435104	S5_1435189	1	0.085	0.9418	0.9704	0.9698	0.9848	0.1915
S5_22964278	5	22964278	S5_22964279	1	0.001	0.9392	1.0000	0.9352	0.9979	0.1674
S5_20948439	5	20948439	S5_20948481	1	0.042	0.9373	1.0000	0.9668	1.0000	0.1616
S5_12240486	5	12240486	S5_12242856	5	2.37	0.9357	1.0000	0.9684	1.0000	0.1922
S5_2180136	5	2180136	S5_2180151	1	0.015	0.9319	1.0000	0.9294	0.9987	0.1484
S5_2825645	5	2825645	S5_2825669	1	0.024	0.9278	0.9632	0.9595	0.9796	0.1418
S5_2798765	5	2798765	S5_2798779	1	0.014	0.9252	1.0000	0.9605	1.0000	0.1969

S5_28297486	5	28297486	S5_28297491	1	0.005	0.9241	1.0000	0.9227	0.9992	0.1255
S5_8638448	5	8638448	S5_8638532	1	0.084	0.9223	1.0000	0.9626	1.0000	0.0749
S5_1703889	5	1703889	S5_1705879	1	1.99	0.9222	0.9603	0.9587	0.9791	0.1313
S5_28325579	5	28325579	S5_28325602	2	0.023	0.9221	1.0000	0.9189	0.9983	0.1218
S5_28256255	5	28256255	S5_28258653	1	2.398	0.9220	1.0000	0.9187	0.9983	0.1228
S5_1197966	5	1197966	S5_1202703	1	4.737	0.9205	1.0000	0.9591	1.0000	0.1359
S5_28297491	5	28297491	S5_28306399	4	8.908	0.9199	1.0000	0.9605	1.0000	0.1210
S5_15566357	5	15566357	S5_15566366	1	0.009	0.9185	1.0000	0.9168	0.9991	0.1280
S5_16652194	5	16652194	S5_16656895	1	4.701	0.9177	1.0000	0.9573	1.0000	0.0619
S5_16277003	5	16277003	S5_16277009	1	0.006	0.9175	1.0000	0.9175	1.0000	0.1147
S5_27844864	5	27844864	S5_27844881	1	0.017	0.9173	0.9783	0.9533	0.9973	0.2374
S5_23737585	5	23737585	S5_23737638	1	0.053	0.9161	0.9779	0.9310	0.9859	0.2309
S5_9258879	5	9258879	S5_9311201	1	52.322	0.9114	1.0000	0.9516	1.0000	0.0571
S5_24030311	5	24030311	S5_24030323	1	0.012	0.9091	1.0000	0.9047	0.9976	0.2078
S5_28320997	5	28320997	S5_28325493	1	4.496	0.9081	1.0000	0.9513	1.0000	0.1137
S5_21465665	5	21465665	S5_21472954	1	7.289	0.9067	1.0000	0.9504	1.0000	0.2117
S5_15754855	5	15754855	S5_15796591	2	41.736	0.9050	1.0000	0.9520	1.0000	0.1339
S5_28053391	5	28053391	S5_28056620	2	3.229	0.9033	0.9744	0.9254	0.9862	0.2381
S5_844615	5	844615	S5_844673	1	0.058	0.9025	1.0000	0.9493	1.0000	0.1951
S5_12599778	5	12599778	S5_12599779	1	0.001	0.9010	1.0000	0.8565	0.9750	0.1848
S5_4758533	5	4758533	S5_4758534	1	0.001	0.8985	1.0000	0.9407	1.0000	0.0944
S5_4758534	5	4758534	S5_4758536	1	0.002	0.8985	1.0000	0.9407	1.0000	0.0944
S5_14743213	5	14743213	S5_14743228	1	0.015	0.8969	1.0000	0.8627	0.9808	0.2056
S5_20482918	5	20482918	S5_20490456	1	7.538	0.8930	0.9625	0.9421	0.9886	0.1483
S5_5366085	5	5366085	S5_5369824	3	3.739	0.8915	0.9442	0.9442	0.9717	0.0907
S5_23565973	5	23565973	S5_23566176	1	0.203	0.8895	1.0000	0.9424	1.0000	0.0872
S5_13249049	5	13249049	S5_13279981	1	30.932	0.8892	1.0000	0.9044	1.0000	0.1291
S5_26278135	5	26278135	S5_26289896	3	11.761	0.8884	0.9425	0.9382	0.9686	0.1855
S5_28258653	5	28258653	S5_28297486	4	38.833	0.8869	1.0000	0.9040	1.0000	0.1224
S5_1260435	5	1260435	S5_1297362	1	36.927	0.8847	1.0000	0.9024	1.0000	0.1336

S5_25850173	5	25850173	S5_25851010	1	0.837	0.8840	1.0000	0.9372	1.0000	0.0853
S5_28306399	5	28306399	S5_28306476	1	0.077	0.8836	1.0000	0.8991	1.0000	0.1197
S5_2043855	5	2043855	S5_2043925	2	0.07	0.8789	0.9675	0.9357	0.9983	0.1688
S5_2040887	5	2040887	S5_2040955	1	0.068	0.8789	1.0000	0.9080	1.0000	0.1756
S5_2042697	5	2042697	S5_2043654	2	0.957	0.8770	0.9566	0.8889	0.9630	0.1157
S5_28306476	5	28306476	S5_28320997	1	14.521	0.8766	1.0000	0.8928	1.0000	0.1132
S5_24466903	5	24466903	S5_24466941	1	0.038	0.8749	1.0000	0.9332	1.0000	0.1489
S5_12242856	5	12242856	S5_12379304	2	136.448	0.8738	0.9348	0.8718	0.9337	0.1909
S5_27686424	5	27686424	S5_27686513	1	0.089	0.8713	0.9545	0.9105	0.9757	0.2330
S5_28325528	5	28325528	S5_28325579	1	0.051	0.8675	1.0000	0.9317	1.0000	0.1143
S5_26746604	5	26746604	S5_26746791	2	0.187	0.8630	1.0000	0.9006	1.0000	0.1712
S5_2825686	5	2825686	S5_2858726	3	33.04	0.8625	0.9626	0.8837	0.9744	0.1392
S5_4186736	5	4186736	S5_4194580	1	7.844	0.8536	0.9676	0.8626	0.9727	0.1584
S5_14299259	5	14299259	S5_14452328	1	153.069	0.8479	0.9208	0.8479	0.9208	0.1224
S5_17842737	5	17842737	S5_17842764	1	0.027	0.8474	1.0000	0.8474	1.0000	0.0591
S5_2000399	5	2000399	S5_2007151	1	6.752	0.8435	1.0000	0.8551	1.0000	0.1560
S5_5227563	5	5227563	S5_5366085	1	138.522	0.8435	0.9438	0.8641	0.9553	0.0886
S5_15566366	5	15566366	S5_15566381	1	0.015	0.8392	1.0000	0.8359	0.9980	0.1184
S5_2180151	5	2180151	S5_2221831	1	41.68	0.8366	0.9304	0.8489	0.9372	0.1465
S5_1315066	5	1315066	S5_1331479	1	16.413	0.8338	0.9131	0.8304	0.9113	0.1141
S5_3186464	5	3186464	S5_3186489	1	0.025	0.8299	1.0000	0.7476	0.9491	0.1040
S5_17240714	5	17240714	S5_17242077	5	1.363	0.8290	0.9616	0.8449	0.9707	0.1485
S5_2396673	5	2396673	S5_2398417	1	1.744	0.8190	1.0000	0.8287	1.0000	0.0719
S5_20863953	5	20863953	S5_20948439	6	84.486	0.8114	0.9008	0.8986	0.9479	0.1541
S5_27857075	5	27857075	S5_27857114	1	0.039	0.8069	1.0000	0.8183	1.0000	0.1952
S5_26777775	5	26777775	S5_26781670	1	3.895	0.8063	0.9455	0.8484	0.9699	0.0969
S5_844686	5	844686	S5_844780	1	0.094	0.8057	0.9769	0.8360	0.9951	0.2273
S5_4372042	5	4372042	S5_4399456	1	27.414	0.8000	0.8944	0.8855	0.9410	0.0465
S5_690783	5	690783	S5_690799	1	0.016	0.7987	1.0000	0.8109	1.0000	0.1488
S5_6659643	5	6659643	S5_6659666	1	0.023	0.7886	1.0000	0.7813	0.9953	0.2110

S5_1150405	5	1150405	S5_1197615	2	47.21	0.7778	1.0000	0.7735	0.9972	0.1080
S5_24149689	5	24149689	S5_24151933	1	2.244	0.7601	1.0000	0.7676	1.0000	0.0829
S5_17842764	5	17842764	S5_17842792	1	0.028	0.7599	1.0000	0.8664	1.0000	0.0719
S5_28196759	5	28196759	S5_28212447	1	15.688	0.7591	0.9372	0.7904	0.9562	0.1836
S5_26246216	5	26246216	S5_26251525	1	5.309	0.7506	0.9405	0.7645	0.9491	0.0832
S5_15566381	5	15566381	S5_15618868	1	52.487	0.7450	1.0000	0.7548	1.0000	0.1170
S5_26890088	5	26890088	S5_26893832	2	3.744	0.7383	0.9536	0.7529	0.9630	0.1141
S5_26354302	5	26354302	S5_26358941	1	4.639	0.7335	1.0000	0.7774	1.0000	0.2045
S5_28350120	5	28350120	S5_28350172	1	0.052	0.7171	1.0000	0.7246	1.0000	0.1288
S5_9038609	5	9038609	S5_9193953	1	155.344	0.7151	0.9543	0.7381	0.9695	0.1208
S5_5084333	5	5084333	S5_5194293	5	109.96	0.7080	0.9388	0.8353	1.0000	0.1016
S5_26278133	5	26278133	S5_26278135	1	0.002	0.6983	1.0000	0.6872	0.9920	0.1728
S5_10767173	5	10767173	S5_10767275	1	0.102	0.6971	1.0000	0.7557	1.0000	0.0545
S5_28222523	5	28222523	S5_28222555	1	0.032	0.6961	1.0000	0.7034	1.0000	0.1068
S5_26271398	5	26271398	S5_26278133	1	6.735	0.6937	1.0000	0.7134	1.0000	0.1976
S5_25834139	5	25834139	S5_25850173	3	16.034	0.6892	0.8506	0.7301	0.8754	0.0918
S5_26087821	5	26087821	S5_26087848	1	0.027	0.6861	1.0000	0.7062	1.0000	0.2054
S5_19835842	5	19835842	S5_19851788	1	15.946	0.6781	1.0000	0.6781	1.0000	0.0947
S5_26289896	5	26289896	S5_26291163	1	1.267	0.6712	0.9413	0.7010	0.9620	0.1740
S5_2798703	5	2798703	S5_2798738	1	0.035	0.6622	0.9715	0.6758	0.9813	0.1804
S5_19855371	5	19855371	S5_19855402	1	0.031	0.6593	1.0000	0.6593	1.0000	0.0832
S5_28325602	5	28325602	S5_28350120	4	24.518	0.6588	1.0000	0.6644	1.0000	0.1198
S5_25706179	5	25706179	S5_25706200	1	0.021	0.6437	1.0000	0.6204	0.9818	0.0572
S5_2798738	5	2798738	S5_2798765	1	0.027	0.6330	1.0000	0.6419	1.0000	0.1719
S5_27844884	5	27844884	S5_27857075	1	12.191	0.6310	0.9466	0.6513	0.9617	0.1889
S5_26208335	5	26208335	S5_26244626	5	36.291	0.6277	0.9411	0.6384	0.9491	0.0888
S5_26647046	5	26647046	S5_26647049	1	0.003	0.6276	1.0000	0.6190	0.9931	0.1603
S5_9650925	5	9650925	S5_9660257	1	9.332	0.6231	0.9698	0.6555	0.9947	-0.1999
S5_23116617	5	23116617	S5_23212658	1	96.041	0.6202	0.7875	0.6202	0.7875	0.0436
S5_26271369	5	26271369	S5_26271380	1	0.011	0.5565	1.0000	0.5377	0.9829	0.1670

S5_2798779	5	2798779	S5_2825645	1	26.866	0.5499	0.9617	0.5896	0.9958	0.1369
S5_26271380	5	26271380	S5_26271398	1	0.018	0.4999	0.9680	0.5013	0.9694	0.1617
S5_16679253	5	16679253	S5_16733825	1	54.572	0.4949	0.8014	0.5795	0.8673	0.0526
S5_19789643	5	19789643	S5_19789779	1	0.136	0.4576	1.0000	0.4560	0.9983	0.0658
S5_19789779	5	19789779	S5_19789793	1	0.014	0.4576	1.0000	0.4560	0.9983	0.0658
S5_19782125	5	19782125	S5_19789526	1	7.401	0.4556	1.0000	0.4540	0.9982	0.0681
S5_17977074	5	17977074	S5_18086955	2	109.881	0.4059	0.8617	0.5992	1.0000	0.0519
S5_19789793	5	19789793	S5_19835842	1	46.049	0.4045	0.9215	0.4283	0.9483	0.0629
S5_26711939	5	26711939	S5_26742679	1	30.74	0.3810	1.0000	0.4067	1.0000	0.1223
S5_6519295	5	6519295	S5_6641188	1	121.893	0.3560	1.0000	0.3742	1.0000	0.0987
S5_3475637	5	3475637	S5_3475640	1	0.003	0.3108	1.0000	0.3021	0.9858	0.1169
S5_23876198	5	23876198	S5_23974004	2	97.806	0.2449	0.5692	0.2555	0.5814	0.0297
S6_187074	6	187074	S6_187075	1	0.001	1.0000	1.0000	1.0000	1.0000	0.0866
S6_3180492	6	3180492	S6_3180506	1	0.014	1.0000	1.0000	1.0000	1.0000	0.1613
S6_4133365	6	4133365	S6_4133366	1	0.001	1.0000	1.0000	1.0000	1.0000	0.2499
S6_4133366	6	4133366	S6_4133391	1	0.025	1.0000	1.0000	1.0000	1.0000	0.2499
S6_4133391	6	4133391	S6_4133401	1	0.01	1.0000	1.0000	1.0000	1.0000	0.2499
S6_5034987	6	5034987	S6_5035051	2	0.064	1.0000	1.0000	1.0000	1.0000	0.1171
S6_5035051	6	5035051	S6_5035062	1	0.011	1.0000	1.0000	1.0000	1.0000	0.1161
S6_5237489	6	5237489	S6_5246797	1	9.308	1.0000	1.0000	1.0000	1.0000	0.1071
S6_25247736	6	25247736	S6_25259805	5	12.069	1.0000	1.0000	1.0000	1.0000	0.1068
S6_25259805	6	25259805	S6_25260214	1	0.409	1.0000	1.0000	1.0000	1.0000	0.1211
S6_26378562	6	26378562	S6_26391515	3	12.953	1.0000	1.0000	1.0000	1.0000	0.0732
S6_27871648	6	27871648	S6_27871649	1	0.001	1.0000	1.0000	1.0000	1.0000	0.1467
S6_27871649	6	27871649	S6_27871651	1	0.002	1.0000	1.0000	1.0000	1.0000	0.1467
S6_7679938	6	7679938	S6_7679986	2	0.048	1.0000	1.0000	1.0000	1.0000	0.0764
S6_13465025	6	13465025	S6_13466204	1	1.179	1.0000	1.0000	1.0000	1.0000	0.1181
S6_28919195	6	28919195	S6_28920735	1	1.54	1.0000	1.0000	1.0000	1.0000	0.1986
S6_6931736	6	6931736	S6_6931737	1	0.001	1.0000	1.0000	1.0000	1.0000	0.1697
S6_516416	6	516416	S6_516441	1	0.025	1.0000	1.0000	1.0000	1.0000	0.1479

S6_5655454	6	5655454	S6_5657755	1	2.301	1.0000	1.0000	1.0000	1.0000	0.1515
S6_507668	6	507668	S6_507761	1	0.093	1.0000	1.0000	1.0000	1.0000	0.1528
S6_983587	6	983587	S6_983600	1	0.013	1.0000	1.0000	1.0000	1.0000	0.1435
S6_25222292	6	25222292	S6_25222293	1	0.001	1.0000	1.0000	1.0000	1.0000	0.1271
S6_5384680	6	5384680	S6_5384681	1	0.001	1.0000	1.0000	1.0000	1.0000	0.1607
S6_7642841	6	7642841	S6_7645851	1	3.01	1.0000	1.0000	1.0000	1.0000	0.1123
S6_7645851	6	7645851	S6_7645860	1	0.009	1.0000	1.0000	1.0000	1.0000	0.1123
S6_484476	6	484476	S6_484477	1	0.001	1.0000	1.0000	1.0000	1.0000	0.1045
S6_26454025	6	26454025	S6_26454057	1	0.032	1.0000	1.0000	1.0000	1.0000	0.1292
S6_9710972	6	9710972	S6_9710987	1	0.015	1.0000	1.0000	1.0000	1.0000	0.0953
S6_5638258	6	5638258	S6_5638269	1	0.011	1.0000	1.0000	1.0000	1.0000	0.2383
S6_9536763	6	9536763	S6_9536769	1	0.006	1.0000	1.0000	1.0000	1.0000	0.0713
S6_15866656	6	15866656	S6_15866708	1	0.052	1.0000	1.0000	1.0000	1.0000	0.2256
S6_25091365	6	25091365	S6_25091391	1	0.026	1.0000	1.0000	1.0000	1.0000	0.0535
S6_2375504	6	2375504	S6_2375511	1	0.007	1.0000	1.0000	1.0000	1.0000	0.1835
S6_12043352	6	12043352	S6_12043354	1	0.002	1.0000	1.0000	1.0000	1.0000	0.2474
S6_12043354	6	12043354	S6_12043355	1	0.001	1.0000	1.0000	1.0000	1.0000	0.2474
S6_12043355	6	12043355	S6_12043356	1	0.001	1.0000	1.0000	1.0000	1.0000	0.2474
S6_27871603	6	27871603	S6_27871641	1	0.038	1.0000	1.0000	1.0000	1.0000	0.1750
S6_8213143	6	8213143	S6_8213170	1	0.027	1.0000	1.0000	1.0000	1.0000	0.1942
S6_18071295	6	18071295	S6_18071296	1	0.001	1.0000	1.0000	1.0000	1.0000	0.1424
S6_9710831	6	9710831	S6_9710832	1	0.001	1.0000	1.0000	1.0000	1.0000	0.1543
S6_9710832	6	9710832	S6_9710833	1	0.001	1.0000	1.0000	1.0000	1.0000	0.1543
S6_7653426	6	7653426	S6_7653427	1	0.001	1.0000	1.0000	1.0000	1.0000	0.2490
S6_27752794	6	27752794	S6_27762053	2	9.259	1.0000	1.0000	1.0000	1.0000	0.1473
S6_7753657	6	7753657	S6_7753683	1	0.026	1.0000	1.0000	1.0000	1.0000	0.2038
S6_22382220	6	22382220	S6_22382236	1	0.016	1.0000	1.0000	1.0000	1.0000	0.1888
S6_973846	6	973846	S6_973972	1	0.126	1.0000	1.0000	1.0000	1.0000	0.0951
S6_14223599	6	14223599	S6_14223636	1	0.037	1.0000	1.0000	1.0000	1.0000	0.2366
S6_29092759	6	29092759	S6_29092777	1	0.018	1.0000	1.0000	1.0000	1.0000	0.1751

S6_26614401	6	26614401	S6_26614408	1	0.007	1.0000	1.0000	1.0000	1.0000	0.1692
S6_26614408	6	26614408	S6_26614413	1	0.005	1.0000	1.0000	1.0000	1.0000	0.1692
S6_26614413	6	26614413	S6_26614419	1	0.006	1.0000	1.0000	1.0000	1.0000	0.1692
S6_3416636	6	3416636	S6_3416644	1	0.008	1.0000	1.0000	1.0000	1.0000	0.2462
S6_4467966	6	4467966	S6_4468125	2	0.159	1.0000	1.0000	1.0000	1.0000	0.0670
S6_4468125	6	4468125	S6_4471947	1	3.822	1.0000	1.0000	1.0000	1.0000	0.0670
S6_4471947	6	4471947	S6_4471965	1	0.018	1.0000	1.0000	1.0000	1.0000	0.0670
S6_4471965	6	4471965	S6_4473150	1	1.185	1.0000	1.0000	1.0000	1.0000	0.0670
S6_20661563	6	20661563	S6_20661564	1	0.001	1.0000	1.0000	1.0000	1.0000	0.1343
S6_20661564	6	20661564	S6_20661565	1	0.001	1.0000	1.0000	1.0000	1.0000	0.1343
S6_20661565	6	20661565	S6_20661567	1	0.002	1.0000	1.0000	1.0000	1.0000	0.1343
S6_20661567	6	20661567	S6_20661582	1	0.015	1.0000	1.0000	1.0000	1.0000	0.1343
S6_7344330	6	7344330	S6_7344361	1	0.031	1.0000	1.0000	1.0000	1.0000	0.1986
S6_7344361	6	7344361	S6_7344364	1	0.003	1.0000	1.0000	1.0000	1.0000	0.1986
S6_7498707	6	7498707	S6_7498716	1	0.009	1.0000	1.0000	1.0000	1.0000	0.2246
S6_2966563	6	2966563	S6_2966587	2	0.024	1.0000	1.0000	1.0000	1.0000	0.2009
S6_1105384	6	1105384	S6_1105389	1	0.005	1.0000	1.0000	1.0000	1.0000	0.2466
S6_12039887	6	12039887	S6_12039895	1	0.008	1.0000	1.0000	1.0000	1.0000	0.2499
S6_14528685	6	14528685	S6_14528686	1	0.001	1.0000	1.0000	1.0000	1.0000	0.1754
S6_14528686	6	14528686	S6_14528687	1	0.001	1.0000	1.0000	1.0000	1.0000	0.1754
S6_14528687	6	14528687	S6_14528688	1	0.001	1.0000	1.0000	1.0000	1.0000	0.1754
S6_14528688	6	14528688	S6_14528689	1	0.001	1.0000	1.0000	1.0000	1.0000	0.1754
S6_14528689	6	14528689	S6_14528690	1	0.001	1.0000	1.0000	1.0000	1.0000	0.1754
S6_14528690	6	14528690	S6_14528691	1	0.001	1.0000	1.0000	1.0000	1.0000	0.1754
S6_14528691	6	14528691	S6_14528692	1	0.001	1.0000	1.0000	1.0000	1.0000	0.1754
S6_14528692	6	14528692	S6_14528693	1	0.001	1.0000	1.0000	1.0000	1.0000	0.1754
S6_14528693	6	14528693	S6_14528696	1	0.003	1.0000	1.0000	1.0000	1.0000	0.1754
S6_14528696	6	14528696	S6_14528699	1	0.003	1.0000	1.0000	1.0000	1.0000	0.1754
S6_14528699	6	14528699	S6_14528700	1	0.001	1.0000	1.0000	1.0000	1.0000	0.1754
S6_14528700	6	14528700	S6_14528701	1	0.001	1.0000	1.0000	1.0000	1.0000	0.1754

S6_14528701	6	14528701	S6_14528702	1	0.001	1.0000	1.0000	1.0000	1.0000	0.1754
S6_14528702	6	14528702	S6_14528705	1	0.003	1.0000	1.0000	1.0000	1.0000	0.1754
S6_14528705	6	14528705	S6_14528706	1	0.001	1.0000	1.0000	1.0000	1.0000	0.1754
S6_14528706	6	14528706	S6_14528707	1	0.001	1.0000	1.0000	1.0000	1.0000	0.1754
S6_14528707	6	14528707	S6_14528708	1	0.001	1.0000	1.0000	1.0000	1.0000	0.1754
S6_14528708	6	14528708	S6_14528710	1	0.002	1.0000	1.0000	1.0000	1.0000	0.1754
S6_20433874	6	20433874	S6_20433875	1	0.001	1.0000	1.0000	1.0000	1.0000	0.0748
S6_20433875	6	20433875	S6_20433876	1	0.001	1.0000	1.0000	1.0000	1.0000	0.0748
S6_29174101	6	29174101	S6_29174112	1	0.011	1.0000	1.0000	1.0000	1.0000	0.0883
S6_13878521	6	13878521	S6_13878527	1	0.006	1.0000	1.0000	1.0000	1.0000	0.1224
S6_13878527	6	13878527	S6_13878535	1	0.008	1.0000	1.0000	1.0000	1.0000	0.1224
S6_9793414	6	9793414	S6_9793416	1	0.002	1.0000	1.0000	1.0000	1.0000	0.1114
S6_9793416	6	9793416	S6_9793422	1	0.006	1.0000	1.0000	1.0000	1.0000	0.1114
S6_9793422	6	9793422	S6_9793423	1	0.001	1.0000	1.0000	1.0000	1.0000	0.1114
S6_28645037	6	28645037	S6_28645050	1	0.013	1.0000	1.0000	1.0000	1.0000	0.2462
S6_975636	6	975636	S6_975637	1	0.001	1.0000	1.0000	1.0000	1.0000	0.1913
S6_975637	6	975637	S6_975638	1	0.001	1.0000	1.0000	1.0000	1.0000	0.1913
S6_975638	6	975638	S6_975640	1	0.002	1.0000	1.0000	1.0000	1.0000	0.1913
S6_975640	6	975640	S6_975642	1	0.002	1.0000	1.0000	1.0000	1.0000	0.1913
S6_663212	6	663212	S6_663237	1	0.025	1.0000	1.0000	1.0000	1.0000	0.2450
S6_2306140	6	2306140	S6_2306181	1	0.041	1.0000	1.0000	1.0000	1.0000	0.0545
S6_14767583	6	14767583	S6_14767584	1	0.001	1.0000	1.0000	1.0000	1.0000	0.1084
S6_14767584	6	14767584	S6_14767585	1	0.001	1.0000	1.0000	1.0000	1.0000	0.1084
S6_10884661	6	10884661	S6_10920645	1	35.984	0.9774	1.0000	0.9878	1.0000	0.2239
S6_28293511	6	28293511	S6_28294334	1	0.823	0.9765	1.0000	0.9883	1.0000	0.2406
S6_875904	6	875904	S6_876012	2	0.108	0.9719	1.0000	0.9852	1.0000	0.1914
S6_2973905	6	2973905	S6_2973923	1	0.018	0.9703	1.0000	0.9841	1.0000	0.1697
S6_2255865	6	2255865	S6_2256075	2	0.21	0.9668	1.0000	0.9826	1.0000	0.1514
S6_516441	6	516441	S6_524359	1	7.918	0.9644	1.0000	0.9821	1.0000	0.1589
S6_13815972	6	13815972	S6_13815975	1	0.003	0.9627	1.0000	0.9800	1.0000	0.1357

S6_16155976	6	16155976	S6_16156024	1	0.048	0.9618	1.0000	0.9806	1.0000	0.1323
S6_916580	6	916580	S6_916747	1	0.167	0.9612	1.0000	0.9800	1.0000	0.1565
S6_25855716	6	25855716	S6_25856917	1	1.201	0.9596	1.0000	0.9795	1.0000	0.1251
S6_4135294	6	4135294	S6_4135333	1	0.039	0.9585	1.0000	0.9792	1.0000	0.2456
S6_28260082	6	28260082	S6_28269548	3	9.466	0.9571	1.0000	0.9778	1.0000	0.1393
S6_27747535	6	27747535	S6_27752794	1	5.259	0.9568	1.0000	0.9778	1.0000	0.1436
S6_24924606	6	24924606	S6_24930561	1	5.955	0.9563	0.9779	0.9769	0.9884	0.2380
S6_19616318	6	19616318	S6_19623934	1	7.616	0.9562	1.0000	0.9780	1.0000	0.2349
S6_11202028	6	11202028	S6_11202301	1	0.273	0.9534	1.0000	0.9763	1.0000	0.2355
S6_147144	6	147144	S6_157987	5	10.843	0.9527	1.0000	0.9740	1.0000	0.1060
S6_11201458	6	11201458	S6_11201908	1	0.45	0.9524	0.9759	0.9749	0.9874	0.2304
S6_11340847	6	11340847	S6_11341487	2	0.64	0.9523	0.9759	0.9754	0.9876	0.2351
S6_28269548	6	28269548	S6_28269727	1	0.179	0.9517	1.0000	0.9751	1.0000	0.1188
S6_15556698	6	15556698	S6_15556699	1	0.001	0.9511	1.0000	0.9486	0.9987	0.2260
S6_18190554	6	18190554	S6_18194993	1	4.439	0.9511	1.0000	0.9752	1.0000	0.2073
S6_29531236	6	29531236	S6_29546112	1	14.876	0.9505	0.9749	0.9749	0.9874	0.2265
S6_29531221	6	29531221	S6_29531236	1	0.015	0.9488	0.9741	0.9741	0.9870	0.2266
S6_15556699	6	15556699	S6_15556778	1	0.079	0.9477	0.9735	0.9722	0.9860	0.2241
S6_22382236	6	22382236	S6_22382275	1	0.039	0.9475	1.0000	0.9454	0.9989	0.1860
S6_6491771	6	6491771	S6_6491793	1	0.022	0.9473	0.9733	0.9731	0.9865	0.1924
S6_5593866	6	5593866	S6_5627403	4	33.537	0.9464	1.0000	0.9710	1.0000	0.1004
S6_18194993	6	18194993	S6_18195620	1	0.627	0.9458	0.9725	0.9718	0.9858	0.2164
S6_2890008	6	2890008	S6_2890225	1	0.217	0.9435	1.0000	0.9707	1.0000	0.1860
S6_25246620	6	25246620	S6_25247658	1	1.038	0.9431	1.0000	0.9722	1.0000	0.1078
S6_516413	6	516413	S6_516416	1	0.003	0.9340	1.0000	0.9329	0.9994	0.1459
S6_507761	6	507761	S6_516413	2	8.652	0.9337	1.0000	0.9326	0.9994	0.1482
S6_11305649	6	11305649	S6_11340847	1	35.198	0.9322	0.9767	0.9649	0.9937	0.2343
S6_12204185	6	12204185	S6_12351588	2	147.403	0.9321	1.0000	0.9609	1.0000	0.0741
S6_11305573	6	11305573	S6_11305649	1	0.076	0.9318	1.0000	0.9423	1.0000	0.2368
S6_983600	6	983600	S6_983624	1	0.024	0.9308	1.0000	0.9640	1.0000	0.1442

S6_15016293	6	15016293	S6_15082105	1	65.812	0.9289	0.9755	0.9623	0.9929	0.2159
S6_20610839	6	20610839	S6_20639963	1	29.124	0.9250	0.9741	0.9605	0.9926	0.2205
S6_29527928	6	29527928	S6_29531221	1	3.293	0.9231	0.9734	0.9599	0.9927	0.2148
S6_8023360	6	8023360	S6_8042369	1	19.009	0.9229	1.0000	0.9629	1.0000	0.0687
S6_11995870	6	11995870	S6_11998088	1	2.218	0.9194	1.0000	0.9372	1.0000	0.2398
S6_26232374	6	26232374	S6_26232382	1	0.008	0.9162	1.0000	0.9301	1.0000	0.1704
S6_7318607	6	7318607	S6_7318620	1	0.013	0.9149	1.0000	0.9262	1.0000	0.1673
S6_11202301	6	11202301	S6_11237645	1	35.344	0.9108	0.9544	0.9312	0.9650	0.2298
S6_4545831	6	4545831	S6_4545878	2	0.047	0.9097	1.0000	0.9235	1.0000	0.1730
S6_4133401	6	4133401	S6_4135294	1	1.893	0.9066	1.0000	0.9066	1.0000	0.2366
S6_5233209	6	5233209	S6_5237489	1	4.28	0.9060	1.0000	0.9512	1.0000	0.1018
S6_11981108	6	11981108	S6_11995870	2	14.762	0.9027	1.0000	0.9511	1.0000	0.2411
S6_25273783	6	25273783	S6_25279002	2	5.219	0.9025	1.0000	0.9450	1.0000	0.0976
S6_25360549	6	25360549	S6_25374549	3	14	0.9022	1.0000	0.9478	1.0000	0.0996
S6_4877574	6	4877574	S6_4877684	1	0.11	0.9011	0.9492	0.9492	0.9743	0.1432
S6_25644947	6	25644947	S6_25656435	1	11.488	0.8995	0.9583	0.9266	0.9726	0.2405
S6_25099323	6	25099323	S6_25099527	1	0.204	0.8981	1.0000	0.9483	1.0000	0.2352
S6_13031747	6	13031747	S6_13074491	1	42.744	0.8955	1.0000	0.8955	1.0000	0.1113
S6_3218313	6	3218313	S6_3218627	1	0.314	0.8951	1.0000	0.9191	1.0000	0.1922
S6_16233618	6	16233618	S6_16236696	1	3.078	0.8943	0.9630	0.9450	0.9899	0.1423
S6_3901793	6	3901793	S6_3901958	1	0.165	0.8936	1.0000	0.9435	1.0000	0.1703
S6_14223263	6	14223263	S6_14223599	2	0.336	0.8882	0.9701	0.9120	0.9831	0.2258
S6_26232382	6	26232382	S6_26232487	1	0.105	0.8873	0.9699	0.9145	0.9846	0.1741
S6_8337787	6	8337787	S6_8343811	3	6.024	0.8861	1.0000	0.9017	1.0000	0.1436
S6_27702886	6	27702886	S6_27747535	1	44.649	0.8830	0.9588	0.8948	0.9652	0.1262
S6_5593733	6	5593733	S6_5593866	1	0.133	0.8827	1.0000	0.9365	1.0000	0.0934
S6_28613464	6	28613464	S6_28613482	1	0.018	0.8816	1.0000	0.8753	0.9964	0.2295
S6_1363953	6	1363953	S6_1369646	3	5.693	0.8773	0.9567	0.9365	0.9884	0.1376
S6_12043356	6	12043356	S6_12048210	1	4.854	0.8725	0.9465	0.9062	0.9646	0.2344
S6_12351588	6	12351588	S6_12351612	1	0.024	0.8722	1.0000	0.9306	1.0000	0.0750

S6_908493	6	908493	S6_908595	1	0.102	0.8706	1.0000	0.8784	1.0000	0.1779
S6_27863356	6	27863356	S6_27871603	1	8.247	0.8685	0.9319	0.8957	0.9464	0.1656
S6_11237645	6	11237645	S6_11305573	1	67.928	0.8684	0.9759	0.9102	0.9991	0.2299
S6_906984	6	906984	S6_908493	2	1.509	0.8673	0.9442	0.8746	0.9481	0.1753
S6_19894038	6	19894038	S6_19894145	1	0.107	0.8648	0.9520	0.8841	0.9625	0.2089
S6_1451557	6	1451557	S6_1453469	1	1.912	0.8567	0.9684	0.9185	1.0000	0.1729
S6_5269877	6	5269877	S6_5278753	3	8.876	0.8546	1.0000	0.8762	1.0000	0.0920
S6_25260214	6	25260214	S6_25267372	1	7.158	0.8528	0.9474	0.9224	0.9853	0.1103
S6_7679936	6	7679936	S6_7679938	1	0.002	0.8462	1.0000	0.8462	1.0000	0.0655
S6_3696172	6	3696172	S6_3698514	1	2.342	0.8442	0.9654	0.9181	1.0000	0.2019
S6_254951	6	254951	S6_267162	2	12.211	0.8438	0.9747	0.8468	0.9765	0.2018
S6_5593685	6	5593685	S6_5593733	1	0.048	0.8419	0.9432	0.9162	0.9840	0.0985
S6_1083834	6	1083834	S6_1083931	1	0.097	0.8365	1.0000	0.8731	1.0000	0.1100
S6_973680	6	973680	S6_973782	1	0.102	0.8340	1.0000	0.9156	1.0000	0.0702
S6_19616245	6	19616245	S6_19616318	1	0.073	0.8271	0.9751	0.8465	0.9865	0.2223
S6_6044492	6	6044492	S6_6063081	1	18.589	0.8260	0.9608	0.8422	0.9702	0.1289
S6_16821151	6	16821151	S6_16846420	1	25.269	0.8257	1.0000	0.8412	1.0000	0.0844
S6_27994413	6	27994413	S6_28002154	1	7.741	0.8221	1.0000	0.9041	1.0000	0.1024
S6_25247658	6	25247658	S6_25247736	1	0.078	0.8161	1.0000	0.8473	1.0000	0.0848
S6_9718897	6	9718897	S6_9718940	1	0.043	0.8152	0.9206	0.8991	0.9668	0.1256
S6_26505383	6	26505383	S6_26565310	1	59.927	0.8136	0.9478	0.8521	0.9700	0.0990
S6_2341346	6	2341346	S6_2375504	1	34.158	0.8106	0.9132	0.8174	0.9171	0.1671
S6_527486	6	527486	S6_543412	1	15.926	0.8104	0.9184	0.8584	0.9452	0.1364
S6_4473150	6	4473150	S6_4473185	1	0.035	0.8098	1.0000	0.8252	1.0000	0.0665
S6_7066125	6	7066125	S6_7067908	1	1.783	0.8070	1.0000	0.8021	0.9970	0.0868
S6_28450007	6	28450007	S6_28450082	1	0.075	0.7821	0.9757	0.8194	0.9988	0.2154
S6_524359	6	524359	S6_527486	1	3.127	0.7768	0.9618	0.8155	0.9855	0.1519
S6_25656435	6	25656435	S6_25683450	1	27.015	0.7727	0.9765	0.8024	0.9951	0.2214
S6_25222293	6	25222293	S6_25222302	1	0.009	0.7714	1.0000	0.8151	1.0000	0.1252
S6_25222302	6	25222302	S6_25222311	1	0.009	0.7714	1.0000	0.8151	1.0000	0.1252

S6_3404454	6	3404454	S6_3416558	2	12.104	0.7667	1.0000	0.8106	1.0000	0.1359
S6_18059911	6	18059911	S6_18060077	1	0.166	0.7623	0.8956	0.8157	0.9264	0.0988
S6_1449724	6	1449724	S6_1451557	1	1.833	0.7553	0.9287	0.8275	0.9721	0.1593
S6_5542552	6	5542552	S6_5593685	2	51.133	0.7440	0.9032	0.8094	0.9421	0.1027
S6_24617530	6	24617530	S6_24617987	2	0.457	0.7425	1.0000	0.7807	1.0000	0.2164
S6_23964843	6	23964843	S6_23965103	2	0.26	0.7408	0.9542	0.7838	0.9814	0.1105
S6_28189440	6	28189440	S6_28204708	1	15.268	0.7354	1.0000	0.8522	1.0000	0.0935
S6_25229908	6	25229908	S6_25231860	1	1.952	0.7354	0.9459	0.7762	0.9718	0.1108
S6_16560975	6	16560975	S6_16561051	1	0.076	0.7351	1.0000	0.7811	1.0000	0.0536
S6_5118568	6	5118568	S6_5118708	5	0.14	0.7265	0.9436	0.7435	0.9545	0.1746
S6_28448546	6	28448546	S6_28450007	1	1.461	0.7185	0.9750	0.7844	1.0000	0.2114
S6_27795800	6	27795800	S6_27795818	1	0.018	0.7154	1.0000	0.7154	1.0000	0.1019
S6_25231860	6	25231860	S6_25246620	1	14.76	0.7143	1.0000	0.7143	1.0000	0.1042
S6_157987	6	157987	S6_162168	2	4.181	0.6955	0.9355	0.7343	0.9612	0.0943
S6_658122	6	658122	S6_663212	1	5.09	0.6943	0.9468	0.7304	0.9711	0.2038
S6_14517453	6	14517453	S6_14527972	1	10.519	0.6862	0.9274	0.7119	0.9445	0.1501
S6_2559235	6	2559235	S6_2586420	1	27.185	0.6730	0.8203	0.6730	0.8203	0.0550
S6_5246797	6	5246797	S6_5248700	2	1.903	0.6700	1.0000	0.6700	1.0000	0.0791
S6_15340282	6	15340282	S6_15353467	1	13.185	0.6696	1.0000	0.7049	1.0000	0.0904
S6_29174112	6	29174112	S6_29180833	1	6.721	0.6686	0.8694	0.7018	0.8907	0.0704
S6_3552112	6	3552112	S6_3557410	2	5.298	0.6675	0.8475	0.6878	0.8603	0.1190
S6_1964074	6	1964074	S6_1964077	1	0.003	0.6615	1.0000	0.6722	1.0000	0.0609
S6_9471109	6	9471109	S6_9536763	2	65.654	0.6420	1.0000	0.6844	1.0000	0.0638
S6_5265364	6	5265364	S6_5269877	2	4.513	0.6284	1.0000	0.6284	1.0000	0.0872
S6_29141136	6	29141136	S6_29141156	1	0.02	0.6259	0.9150	0.6492	0.9319	0.0575
S6_6027523	6	6027523	S6_6038373	2	10.85	0.6156	0.9385	0.6485	0.9633	0.1779
S6_13646504	6	13646504	S6_13646518	1	0.014	0.6098	1.0000	0.6028	0.9943	0.1260
S6_27634954	6	27634954	S6_27702886	1	67.932	0.5965	0.7885	0.6288	0.8095	0.1020
S6_9608123	6	9608123	S6_9612944	1	4.821	0.5746	0.8586	0.6090	0.8839	0.0727
S6_25267372	6	25267372	S6_25273783	5	6.411	0.5719	0.8350	0.5843	0.8440	0.0803

S6_1274460	6	1274460	S6_1302815	2	28.355	0.5274	0.7487	0.5791	0.7845	0.0761
S6_2999468	6	2999468	S6_2999484	1	0.016	0.5156	1.0000	0.4920	0.9768	0.1608
S6_3060289	6	3060289	S6_3061650	1	1.361	0.5054	1.0000	0.5263	1.0000	0.1646
S6_26266190	6	26266190	S6_26302680	2	36.49	0.5002	0.9582	0.5162	0.9734	0.1339
S6_8343811	6	8343811	S6_8394272	1	50.461	0.4999	0.9437	0.5111	0.9542	0.0881
S6_5248700	6	5248700	S6_5265364	1	16.664	0.4889	1.0000	0.4889	1.0000	0.0728
S6_1189474	6	1189474	S6_1198427	2	8.953	0.4749	0.7508	0.4880	0.7610	0.0630
S6_24280497	6	24280497	S6_24280515	1	0.018	0.4343	1.0000	0.4442	1.0000	0.1496
S6_5415363	6	5415363	S6_5417728	1	2.365	0.4290	1.0000	0.4471	1.0000	0.1055
S6_3416632	6	3416632	S6_3416636	1	0.004	0.4206	0.9997	0.0865	0.4533	-0.0696
S6_19787827	6	19787827	S6_19894038	1	106.211	0.3928	0.9177	0.4252	0.9548	0.1217
S6_25279002	6	25279002	S6_25360549	2	81.547	0.3909	0.6252	0.4638	0.6810	0.0669
S6_95582	6	95582	S6_95618	1	0.036	0.3609	1.0000	0.3742	1.0000	0.1223
S6_14527972	6	14527972	S6_14527974	1	0.002	0.3493	1.0000	0.3505	1.0000	0.0680
S6_14527974	6	14527974	S6_14528685	1	0.711	0.3376	1.0000	0.3444	1.0000	0.0680
S6_22406681	6	22406681	S6_22486409	2	79.728	0.3268	0.6610	0.3326	0.6669	0.0408
S6_2999484	6	2999484	S6_2999513	1	0.029	0.2780	1.0000	0.2451	0.9388	0.0969
S6_15555740	6	15555740	S6_15556698	1	0.958	0.2736	1.0000	0.2638	0.9818	0.0860
S7_45233	7	45233	S7_82922	2	37.689	1.0000	1.0000	1.0000	1.0000	0.1161
S7_82922	7	82922	S7_129216	1	46.294	1.0000	1.0000	1.0000	1.0000	0.1224
S7_226096	7	226096	S7_226112	1	0.016	1.0000	1.0000	1.0000	1.0000	0.1171
S7_295198	7	295198	S7_302252	1	7.054	1.0000	1.0000	1.0000	1.0000	0.1084
S7_4832357	7	4832357	S7_4832536	3	0.179	1.0000	1.0000	1.0000	1.0000	0.0703
S7_4995467	7	4995467	S7_4997966	1	2.499	1.0000	1.0000	1.0000	1.0000	0.1238
S7_9205291	7	9205291	S7_9205296	1	0.005	1.0000	1.0000	1.0000	1.0000	0.0842
S7_9205296	7	9205296	S7_9364833	1	159.537	1.0000	1.0000	1.0000	1.0000	0.0842
S7_9939850	7	9939850	S7_9940331	1	0.481	1.0000	1.0000	1.0000	1.0000	0.0842
S7_9940331	7	9940331	S7_10034504	1	94.173	1.0000	1.0000	1.0000	1.0000	0.0842
S7_10034504	7	10034504	S7_10039974	1	5.47	1.0000	1.0000	1.0000	1.0000	0.0900
S7_12542147	7	12542147	S7_12542148	1	0.001	1.0000	1.0000	1.0000	1.0000	0.0967

S7_12542148	7	12542148	S7_12542173	1	0.025	1.0000	1.0000	1.0000	1.0000	0.0967
S7_15749852	7	15749852	S7_15749882	1	0.03	1.0000	1.0000	1.0000	1.0000	0.1814
S7_22521435	7	22521435	S7_22521990	1	0.555	1.0000	1.0000	1.0000	1.0000	0.0988
S7_22665372	7	22665372	S7_22665378	1	0.006	1.0000	1.0000	1.0000	1.0000	0.1641
S7_23467123	7	23467123	S7_23467181	2	0.058	1.0000	1.0000	1.0000	1.0000	0.1171
S7_2035197	7	2035197	S7_2035204	1	0.007	1.0000	1.0000	1.0000	1.0000	0.0494
S7_9447413	7	9447413	S7_9447451	2	0.038	1.0000	1.0000	1.0000	1.0000	0.0918
S7_14147343	7	14147343	S7_14147354	1	0.011	1.0000	1.0000	1.0000	1.0000	0.0598
S7_20219527	7	20219527	S7_20221146	1	1.619	1.0000	1.0000	1.0000	1.0000	0.0781
S7_5264475	7	5264475	S7_5264506	1	0.031	1.0000	1.0000	1.0000	1.0000	0.2439
S7_15517985	7	15517985	S7_15518020	1	0.035	1.0000	1.0000	1.0000	1.0000	0.1629
S7_19484531	7	19484531	S7_19484542	1	0.011	1.0000	1.0000	1.0000	1.0000	0.0663
S7_18825236	7	18825236	S7_18825264	1	0.028	1.0000	1.0000	1.0000	1.0000	0.2057
S7_15058649	7	15058649	S7_15058650	1	0.001	1.0000	1.0000	1.0000	1.0000	0.1821
S7_15058650	7	15058650	S7_15058651	1	0.001	1.0000	1.0000	1.0000	1.0000	0.1821
S7_15058651	7	15058651	S7_15058656	1	0.005	1.0000	1.0000	1.0000	1.0000	0.1821
S7_24072817	7	24072817	S7_24072822	1	0.005	1.0000	1.0000	1.0000	1.0000	0.1619
S7_22589967	7	22589967	S7_22589999	1	0.032	1.0000	1.0000	1.0000	1.0000	0.1556
S7_24094209	7	24094209	S7_24094210	1	0.001	1.0000	1.0000	1.0000	1.0000	0.1783
S7_15517905	7	15517905	S7_15517912	1	0.007	1.0000	1.0000	1.0000	1.0000	0.1564
S7_276651	7	276651	S7_276653	1	0.002	1.0000	1.0000	1.0000	1.0000	0.2383
S7_13719933	7	13719933	S7_13719958	1	0.025	1.0000	1.0000	1.0000	1.0000	0.1054
S7_20024519	7	20024519	S7_20024520	1	0.001	1.0000	1.0000	1.0000	1.0000	0.2455
S7_22250439	7	22250439	S7_22250441	1	0.002	1.0000	1.0000	1.0000	1.0000	0.0800
S7_22250441	7	22250441	S7_22250442	1	0.001	1.0000	1.0000	1.0000	1.0000	0.0800
S7_22250442	7	22250442	S7_22250443	1	0.001	1.0000	1.0000	1.0000	1.0000	0.0800
S7_22250443	7	22250443	S7_22250444	1	0.001	1.0000	1.0000	1.0000	1.0000	0.0800
S7_20024520	7	20024520	S7_20024521	1	0.001	1.0000	1.0000	1.0000	1.0000	0.2461
S7_23884186	7	23884186	S7_23884190	1	0.004	1.0000	1.0000	1.0000	1.0000	0.2480
S7_2725530	7	2725530	S7_2727866	2	2.336	1.0000	1.0000	1.0000	1.0000	0.0631

S7_5327093	7	5327093	S7_5327109	1	0.016	1.0000	1.0000	1.0000	1.0000	0.2283
S7_21697416	7	21697416	S7_21697521	2	0.105	1.0000	1.0000	1.0000	1.0000	0.2090
S7_21846395	7	21846395	S7_21846396	1	0.001	1.0000	1.0000	1.0000	1.0000	0.0650
S7_21846396	7	21846396	S7_21846397	1	0.001	1.0000	1.0000	1.0000	1.0000	0.0650
S7_22590056	7	22590056	S7_22590067	1	0.011	1.0000	1.0000	1.0000	1.0000	0.1575
S7_22592335	7	22592335	S7_22592346	2	0.011	1.0000	1.0000	1.0000	1.0000	0.1600
S7_7689985	7	7689985	S7_7689986	1	0.001	1.0000	1.0000	1.0000	1.0000	0.1523
S7_20008792	7	20008792	S7_20008796	1	0.004	1.0000	1.0000	1.0000	1.0000	0.1449
S7_22396054	7	22396054	S7_22396918	1	0.864	1.0000	1.0000	1.0000	1.0000	0.1235
S7_21029174	7	21029174	S7_21029215	1	0.041	1.0000	1.0000	1.0000	1.0000	0.1161
S7_6673564	7	6673564	S7_6674353	1	0.789	1.0000	1.0000	1.0000	1.0000	0.1338
S7_24586049	7	24586049	S7_24586050	1	0.001	1.0000	1.0000	1.0000	1.0000	0.1213
S7_24586050	7	24586050	S7_24586052	1	0.002	1.0000	1.0000	1.0000	1.0000	0.1213
S7_14711393	7	14711393	S7_14711394	1	0.001	1.0000	1.0000	1.0000	1.0000	0.1986
S7_1962792	7	1962792	S7_1962819	1	0.027	1.0000	1.0000	1.0000	1.0000	0.1606
S7_2655314	7	2655314	S7_2655316	1	0.002	1.0000	1.0000	1.0000	1.0000	0.0927
S7_10797836	7	10797836	S7_10797843	1	0.007	1.0000	1.0000	1.0000	1.0000	0.2496
S7_1338217	7	1338217	S7_1338224	1	0.007	1.0000	1.0000	1.0000	1.0000	0.2482
S7_1338224	7	1338224	S7_1338226	1	0.002	1.0000	1.0000	1.0000	1.0000	0.2482
S7_1338226	7	1338226	S7_1338238	1	0.012	1.0000	1.0000	1.0000	1.0000	0.2482
S7_20491729	7	20491729	S7_20491731	1	0.002	1.0000	1.0000	1.0000	1.0000	0.1540
S7_19284238	7	19284238	S7_19284278	1	0.04	1.0000	1.0000	1.0000	1.0000	0.1423
S7_2729988	7	2729988	S7_2730017	1	0.029	1.0000	1.0000	1.0000	1.0000	0.0844
S7_18211556	7	18211556	S7_18211558	1	0.002	1.0000	1.0000	1.0000	1.0000	0.2006
S7_7381601	7	7381601	S7_7381613	1	0.012	1.0000	1.0000	1.0000	1.0000	0.2246
S7_22282205	7	22282205	S7_22282212	1	0.007	1.0000	1.0000	1.0000	1.0000	0.2499
S7_22282212	7	22282212	S7_22282221	1	0.009	1.0000	1.0000	1.0000	1.0000	0.2499
S7_272695	7	272695	S7_272705	1	0.01	1.0000	1.0000	1.0000	1.0000	0.2467
S7_12542334	7	12542334	S7_12542335	1	0.001	1.0000	1.0000	1.0000	1.0000	0.0519
S7_12542335	7	12542335	S7_12542336	1	0.001	1.0000	1.0000	1.0000	1.0000	0.0519

S7_21853721	7	21853721	S7_21853767	1	0.046	1.0000	1.0000	1.0000	1.0000	0.1675
S7_6220932	7	6220932	S7_6220933	1	0.001	1.0000	1.0000	1.0000	1.0000	0.2047
S7_6185461	7	6185461	S7_6185462	1	0.001	1.0000	1.0000	1.0000	1.0000	0.2175
S7_6185462	7	6185462	S7_6185463	1	0.001	1.0000	1.0000	1.0000	1.0000	0.2175
S7_6185463	7	6185463	S7_6185464	1	0.001	1.0000	1.0000	1.0000	1.0000	0.2175
S7_276635	7	276635	S7_276649	1	0.014	1.0000	1.0000	1.0000	1.0000	0.2468
S7_276682	7	276682	S7_276699	1	0.017	1.0000	1.0000	1.0000	1.0000	0.2468
S7_22264417	7	22264417	S7_22264418	1	0.001	1.0000	1.0000	1.0000	1.0000	0.2496
S7_22264418	7	22264418	S7_22264448	1	0.03	1.0000	1.0000	1.0000	1.0000	0.2496
S7_22264448	7	22264448	S7_22264465	1	0.017	1.0000	1.0000	1.0000	1.0000	0.2496
S7_19284188	7	19284188	S7_19284189	1	0.001	1.0000	1.0000	1.0000	1.0000	0.0625
S7_19284189	7	19284189	S7_19284190	1	0.001	1.0000	1.0000	1.0000	1.0000	0.0625
S7_1044552	7	1044552	S7_1044582	1	0.03	1.0000	1.0000	1.0000	1.0000	0.2317
S7_19284190	7	19284190	S7_19284191	1	0.001	1.0000	1.0000	1.0000	1.0000	0.0631
S7_22195287	7	22195287	S7_22195288	1	0.001	1.0000	1.0000	1.0000	1.0000	0.2133
S7_22195288	7	22195288	S7_22195290	1	0.002	1.0000	1.0000	1.0000	1.0000	0.2133
S7_2437414	7	2437414	S7_2437415	1	0.001	1.0000	1.0000	1.0000	1.0000	0.0696
S7_19867715	7	19867715	S7_19867728	1	0.013	1.0000	1.0000	1.0000	1.0000	0.1902
S7_19280115	7	19280115	S7_19280116	1	0.001	1.0000	1.0000	1.0000	1.0000	0.0764
S7_19280116	7	19280116	S7_19280117	1	0.001	1.0000	1.0000	1.0000	1.0000	0.0764
S7_368182	7	368182	S7_368184	1	0.002	1.0000	1.0000	1.0000	1.0000	0.2070
S7_2610945	7	2610945	S7_2610978	2	0.033	1.0000	1.0000	1.0000	1.0000	0.0683
S7_24021878	7	24021878	S7_24021879	1	0.001	0.9795	1.0000	0.9894	1.0000	0.2476
S7_24021872	7	24021872	S7_24021878	1	0.006	0.9793	1.0000	0.9893	1.0000	0.2471
S7_272705	7	272705	S7_276635	1	3.93	0.9791	1.0000	0.9890	1.0000	0.2456
S7_250280	7	250280	S7_250771	2	0.491	0.9767	1.0000	0.9882	1.0000	0.2171
S7_5598747	7	5598747	S7_5598748	1	0.001	0.9767	1.0000	0.9560	0.9894	0.2135
S7_5598748	7	5598748	S7_5598749	1	0.001	0.9767	1.0000	0.9560	0.9894	0.2135
S7_12077715	7	12077715	S7_12077799	1	0.084	0.9766	1.0000	0.9879	1.0000	0.2474
S7_250902	7	250902	S7_252210	1	1.308	0.9752	1.0000	0.9875	1.0000	0.2155

S7_14702976	7	14702976	S7_14711393	1	8.417	0.9745	1.0000	0.9870	1.0000	0.1984
S7_23369025	7	23369025	S7_23369061	1	0.036	0.9707	1.0000	0.9846	1.0000	0.1739
S7_23369061	7	23369061	S7_23369074	1	0.013	0.9707	1.0000	0.9846	1.0000	0.1739
S7_7689987	7	7689987	S7_7689988	1	0.001	0.9693	1.0000	0.9585	0.9944	0.1617
S7_22589999	7	22589999	S7_22590056	1	0.057	0.9674	1.0000	0.9833	1.0000	0.1558
S7_24072687	7	24072687	S7_24072780	1	0.093	0.9626	1.0000	0.9812	1.0000	0.1545
S7_22396942	7	22396942	S7_22396964	1	0.022	0.9608	1.0000	0.9797	1.0000	0.1276
S7_22396964	7	22396964	S7_22396990	1	0.026	0.9597	1.0000	0.9798	1.0000	0.1240
S7_17500421	7	17500421	S7_17500533	1	0.112	0.9595	1.0000	0.9794	1.0000	0.1273
S7_15517912	7	15517912	S7_15517985	1	0.073	0.9593	1.0000	0.9794	1.0000	0.1652
S7_5264321	7	5264321	S7_5264475	1	0.154	0.9577	0.9786	0.9759	0.9879	0.2409
S7_20352824	7	20352824	S7_20352896	1	0.072	0.9572	1.0000	0.9773	1.0000	0.1166
S7_4323647	7	4323647	S7_4323724	1	0.077	0.9565	1.0000	0.9780	1.0000	0.2471
S7_11332651	7	11332651	S7_11356316	1	23.665	0.9565	1.0000	0.9773	1.0000	0.2468
S7_22386063	7	22386063	S7_22396054	4	9.991	0.9545	1.0000	0.9776	1.0000	0.1311
S7_302252	7	302252	S7_311815	2	9.563	0.9544	1.0000	0.9768	1.0000	0.1090
S7_226112	7	226112	S7_226162	2	0.05	0.9540	1.0000	0.9766	1.0000	0.1140
S7_5381201	7	5381201	S7_5381344	2	0.143	0.9537	1.0000	0.9767	1.0000	0.1184
S7_13698182	7	13698182	S7_13719933	1	21.751	0.9509	1.0000	0.9753	1.0000	0.1021
S7_23303405	7	23303405	S7_23303418	1	0.013	0.9504	1.0000	0.9504	1.0000	0.2084
S7_21153478	7	21153478	S7_21153526	1	0.048	0.9490	1.0000	0.9737	1.0000	0.0972
S7_22496536	7	22496536	S7_22496560	1	0.024	0.9482	1.0000	0.9468	0.9993	0.1885
S7_23303418	7	23303418	S7_23305745	1	2.327	0.9474	1.0000	0.9474	1.0000	0.2048
S7_18211732	7	18211732	S7_18307790	1	96.058	0.9467	1.0000	0.9728	1.0000	0.0958
S7_14830113	7	14830113	S7_14830294	1	0.181	0.9461	0.9727	0.9725	0.9862	0.1862
S7_21697389	7	21697389	S7_21697416	1	0.027	0.9452	1.0000	0.9437	0.9992	0.2053
S7_248811	7	248811	S7_250280	1	1.469	0.9451	0.9721	0.9720	0.9859	0.2298
S7_15058583	7	15058583	S7_15058649	1	0.066	0.9424	1.0000	0.9710	1.0000	0.1766
S7_2641358	7	2641358	S7_2655314	3	13.956	0.9411	1.0000	0.9705	1.0000	0.0955
S7_11103638	7	11103638	S7_11103792	1	0.154	0.9351	0.9777	0.9657	0.9936	0.2453

S7_2676606	7	2676606	S7_2677355	2	0.749	0.9340	1.0000	0.9306	0.9981	0.1457
S7_20022577	7	20022577	S7_20022706	1	0.129	0.9321	0.9767	0.9632	0.9928	0.2195
S7_614603	7	614603	S7_617515	1	2.912	0.9319	1.0000	0.9637	1.0000	0.0764
S7_17765161	7	17765161	S7_17770548	1	5.387	0.9281	1.0000	0.9629	1.0000	0.0685
S7_1213345	7	1213345	S7_1213435	1	0.09	0.9273	0.9749	0.9604	0.9922	0.2044
S7_20008796	7	20008796	S7_20008894	1	0.098	0.9251	0.9618	0.9607	0.9801	0.1458
S7_23305745	7	23305745	S7_23311157	1	5.412	0.9243	1.0000	0.9364	1.0000	0.1982
S7_22496560	7	22496560	S7_22496620	1	0.06	0.9228	1.0000	0.9338	1.0000	0.1860
S7_22464536	7	22464536	S7_22464581	1	0.045	0.9226	0.9605	0.9572	0.9784	0.1279
S7_22464460	7	22464460	S7_22464536	1	0.076	0.9226	0.9605	0.9558	0.9776	0.1278
S7_1069410	7	1069410	S7_1070829	1	1.419	0.9224	0.9732	0.9574	0.9915	0.1908
S7_22396918	7	22396918	S7_22396942	1	0.024	0.9221	1.0000	0.9599	1.0000	0.1245
S7_6721503	7	6721503	S7_6728457	1	6.954	0.9216	0.9600	0.9592	0.9794	0.1530
S7_6728457	7	6728457	S7_6795232	1	66.775	0.9212	1.0000	0.9602	1.0000	0.1492
S7_5256974	7	5256974	S7_5259864	3	2.89	0.9189	0.9586	0.9560	0.9778	0.2438
S7_11119307	7	11119307	S7_11120892	1	1.585	0.9187	0.9787	0.9578	0.9993	0.2442
S7_4796769	7	4796769	S7_4802791	1	6.022	0.9179	1.0000	0.9534	1.0000	0.0594
S7_14618793	7	14618793	S7_14619774	2	0.981	0.9167	1.0000	0.9577	1.0000	0.2008
S7_11103792	7	11103792	S7_11119307	1	15.515	0.9153	0.9778	0.9557	0.9991	0.2442
S7_311815	7	311815	S7_317432	1	5.617	0.9148	1.0000	0.9130	0.9990	0.1118
S7_13637597	7	13637597	S7_13637655	1	0.058	0.9141	1.0000	0.9546	1.0000	0.1195
S7_142221	7	142221	S7_144340	1	2.119	0.9129	0.9771	0.9518	0.9976	0.2273
S7_19519544	7	19519544	S7_19520098	2	0.554	0.9109	1.0000	0.9539	1.0000	0.1189
S7_3483273	7	3483273	S7_3483281	1	0.008	0.9095	1.0000	0.9028	0.9963	0.2065
S7_21853633	7	21853633	S7_21853721	1	0.088	0.9072	1.0000	0.9543	1.0000	0.1672
S7_14577	7	14577	S7_45233	2	30.656	0.9066	0.9522	0.9522	0.9758	0.1097
S7_21681012	7	21681012	S7_21681253	1	0.241	0.9063	1.0000	0.9281	1.0000	0.2018
S7_2760948	7	2760948	S7_2763311	1	2.363	0.9034	1.0000	0.9493	1.0000	0.1978
S7_23299233	7	23299233	S7_23303405	1	4.172	0.9015	0.9739	0.9248	0.9864	0.2056
S7_9613998	7	9613998	S7_9614137	3	0.139	0.9006	1.0000	0.9253	1.0000	0.2392

S7_18804917	7	18804917	S7_18825236	1	20.319	0.9002	1.0000	0.9241	1.0000	0.1944
S7_15680816	7	15680816	S7_15701211	1	20.395	0.8980	1.0000	0.9201	1.0000	0.2173
S7_17765118	7	17765118	S7_17765123	1	0.005	0.8971	1.0000	0.9444	1.0000	0.1398
S7_1106234	7	1106234	S7_1107947	4	1.713	0.8881	0.9759	0.9406	1.0000	0.2341
S7_13596428	7	13596428	S7_13637597	5	41.169	0.8839	0.9402	0.9383	0.9687	0.1025
S7_15229916	7	15229916	S7_15230544	1	0.628	0.8820	1.0000	0.9427	1.0000	0.0979
S7_10797843	7	10797843	S7_10797933	1	0.09	0.8789	0.9779	0.9362	1.0000	0.2411
S7_2434656	7	2434656	S7_2435959	1	1.303	0.8773	1.0000	0.9378	1.0000	0.1740
S7_7689986	7	7689986	S7_7689987	1	0.001	0.8772	1.0000	0.8410	0.9791	0.1442
S7_22386051	7	22386051	S7_22386063	1	0.012	0.8768	1.0000	0.8956	1.0000	0.1332
S7_15749882	7	15749882	S7_15780042	1	30.16	0.8749	0.9558	0.9349	0.9880	0.1774
S7_24065322	7	24065322	S7_24066878	1	1.556	0.8741	0.9661	0.9033	0.9821	0.1811
S7_17627895	7	17627895	S7_17628030	2	0.135	0.8724	1.0000	0.8587	0.9921	0.0706
S7_24072822	7	24072822	S7_24074894	1	2.072	0.8716	0.9654	0.9016	0.9818	0.1594
S7_12918228	7	12918228	S7_13014054	1	95.826	0.8691	1.0000	0.8860	1.0000	0.1037
S7_10196472	7	10196472	S7_10221643	1	25.171	0.8628	0.9699	0.9261	1.0000	0.1739
S7_7381613	7	7381613	S7_7381752	1	0.139	0.8510	1.0000	0.8692	1.0000	0.2037
S7_13591516	7	13591516	S7_13596428	1	4.912	0.8440	1.0000	0.8440	1.0000	0.0762
S7_2763311	7	2763311	S7_2763540	1	0.229	0.8435	1.0000	0.8897	1.0000	0.1954
S7_23290781	7	23290781	S7_23290906	2	0.125	0.8425	0.9745	0.8735	0.9923	0.2240
S7_2677355	7	2677355	S7_2680359	1	3.004	0.8372	0.9636	0.8448	0.9680	0.1373
S7_20562770	7	20562770	S7_20567170	1	4.4	0.8329	0.9328	0.8808	0.9592	0.2313
S7_23006904	7	23006904	S7_23007056	1	0.152	0.8285	0.9102	0.9038	0.9507	0.0631
S7_23282061	7	23282061	S7_23290781	2	8.72	0.8269	0.9457	0.8604	0.9646	0.2219
S7_13694191	7	13694191	S7_13698182	1	3.991	0.8250	1.0000	0.8632	1.0000	0.1060
S7_14711404	7	14711404	S7_14711406	1	0.002	0.8214	1.0000	0.7710	0.9688	0.1379
S7_20221146	7	20221146	S7_20221322	1	0.176	0.8213	1.0000	0.8213	1.0000	0.0621
S7_18391465	7	18391465	S7_18392200	6	0.735	0.8172	0.9490	0.9066	0.9995	0.1153
S7_18189649	7	18189649	S7_18211450	1	21.801	0.8162	1.0000	0.8344	1.0000	0.1247
S7_18307790	7	18307790	S7_18312175	4	4.385	0.8135	1.0000	0.8994	1.0000	0.0976

S7_211947	7	211947	S7_226086	1	14.139	0.8134	1.0000	0.8294	1.0000	0.1147
S7_226086	7	226086	S7_226096	1	0.01	0.8134	1.0000	0.8294	1.0000	0.1147
S7_276649	7	276649	S7_276651	1	0.002	0.8088	1.0000	0.9008	1.0000	0.2301
S7_24705704	7	24705704	S7_24709560	1	3.856	0.8020	0.9274	0.8280	0.9424	0.1781
S7_4737787	7	4737787	S7_4753569	3	15.782	0.8010	0.9188	0.8373	0.9394	0.1795
S7_1484192	7	1484192	S7_1484995	1	0.803	0.7960	0.9603	0.8219	0.9758	0.1309
S7_19807132	7	19807132	S7_19814936	1	7.804	0.7948	0.9245	0.8180	0.9379	0.1518
S7_13991473	7	13991473	S7_14037250	5	45.777	0.7923	1.0000	0.8129	1.0000	0.1178
S7_5381344	7	5381344	S7_5445857	1	64.513	0.7877	0.9508	0.8461	0.9854	0.1138
S7_226162	7	226162	S7_226279	1	0.117	0.7854	0.9067	0.8418	0.9387	0.1105
S7_21029215	7	21029215	S7_21032418	1	3.203	0.7841	1.0000	0.7973	1.0000	0.0954
S7_13690465	7	13690465	S7_13694191	1	3.726	0.7762	0.9358	0.8202	0.9620	0.1000
S7_24074894	7	24074894	S7_24074982	1	0.088	0.7755	0.9273	0.8475	0.9695	0.1499
S7_2825983	7	2825983	S7_2825995	1	0.012	0.7728	1.0000	0.7530	0.9871	0.2077
S7_276653	7	276653	S7_276670	1	0.017	0.7708	0.9762	0.8780	1.0000	0.2272
S7_22654547	7	22654547	S7_22654706	1	0.159	0.7697	0.9252	0.8113	0.9499	0.1461
S7_24542539	7	24542539	S7_24572520	1	29.981	0.7600	1.0000	0.7496	0.9931	0.1310
S7_19507018	7	19507018	S7_19519544	2	12.526	0.7520	1.0000	0.7938	1.0000	0.1154
S7_23493169	7	23493169	S7_23495349	1	2.18	0.7436	1.0000	0.7349	0.9941	0.0730
S7_6800840	7	6800840	S7_6888692	1	87.852	0.7427	0.9726	0.7769	0.9947	0.1913
S7_24074982	7	24074982	S7_24075071	1	0.089	0.7310	0.9204	0.7535	0.9345	0.1264
S7_13637655	7	13637655	S7_13690465	1	52.81	0.7173	0.8733	0.7815	0.9115	0.0924
S7_22585869	7	22585869	S7_22589967	2	4.098	0.7129	0.9586	0.7228	0.9653	0.1218
S7_2438081	7	2438081	S7_2488638	4	50.557	0.7036	0.8855	0.7036	0.8855	0.0889
S7_200572	7	200572	S7_211947	2	11.375	0.6988	0.8833	0.6988	0.8833	0.0983
S7_17694172	7	17694172	S7_17765118	1	70.946	0.6726	0.8201	0.8053	0.8974	0.0602
S7_20221322	7	20221322	S7_20352824	4	131.502	0.6402	0.8730	0.6581	0.8851	0.0830
S7_6721461	7	6721461	S7_6721503	1	0.042	0.6383	1.0000	0.6164	0.9827	0.0894
S7_22565791	7	22565791	S7_22585869	5	20.078	0.6174	0.8770	0.6584	0.9056	0.1141
S7_23378651	7	23378651	S7_23467123	3	88.472	0.5914	0.8056	0.5914	0.8056	0.0911

S7_6674353	7	6674353	S7_6721461	1	47.108	0.5442	0.8877	0.5913	0.9253	0.0852
S7_176927	7	176927	S7_200572	2	23.645	0.5421	0.8787	0.5546	0.8888	0.0952
S7_18392236	7	18392236	S7_18521349	1	129.113	0.5072	0.7850	0.5571	0.8227	0.0445
S7_2676556	7	2676556	S7_2676606	1	0.05	0.4916	1.0000	0.5009	1.0000	0.1258
S7_19089573	7	19089573	S7_19135412	1	45.839	0.4686	1.0000	0.4997	1.0000	0.1420
S7_15780042	7	15780042	S7_15897101	5	117.059	0.4469	1.0000	0.4743	1.0000	0.1358
S7_15680708	7	15680708	S7_15680816	1	0.108	0.3169	1.0000	0.3020	0.9762	0.0957
S7_20567170	7	20567170	S7_20567180	1	0.01	0.2693	1.0000	0.2556	0.9740	0.0946
S8_113609	8	113609	S8_123227	1	9.618	1.0000	1.0000	1.0000	1.0000	0.1249
S8_350862	8	350862	S8_374532	1	23.67	1.0000	1.0000	1.0000	1.0000	0.1138
S8_2037236	8	2037236	S8_2037237	1	0.001	1.0000	1.0000	1.0000	1.0000	0.1652
S8_2134958	8	2134958	S8_2134990	1	0.032	1.0000	1.0000	1.0000	1.0000	0.0988
S8_3378579	8	3378579	S8_3378608	1	0.029	1.0000	1.0000	1.0000	1.0000	0.2486
S8_3378608	8	3378608	S8_3378615	1	0.007	1.0000	1.0000	1.0000	1.0000	0.2486
S8_5064054	8	5064054	S8_5064055	1	0.001	1.0000	1.0000	1.0000	1.0000	0.1631
S8_12506367	8	12506367	S8_12506368	1	0.001	1.0000	1.0000	1.0000	1.0000	0.1288
S8_13319277	8	13319277	S8_13324329	1	5.052	1.0000	1.0000	1.0000	1.0000	0.0560
S8_5487213	8	5487213	S8_5487248	2	0.035	1.0000	1.0000	1.0000	1.0000	0.0757
S8_14874102	8	14874102	S8_14874167	1	0.065	1.0000	1.0000	1.0000	1.0000	0.2467
S8_2795992	8	2795992	S8_2795994	1	0.002	1.0000	1.0000	1.0000	1.0000	0.1359
S8_21365821	8	21365821	S8_21365823	1	0.002	1.0000	1.0000	1.0000	1.0000	0.2298
S8_329316	8	329316	S8_329339	1	0.023	1.0000	1.0000	1.0000	1.0000	0.1479
S8_329369	8	329369	S8_329384	1	0.015	1.0000	1.0000	1.0000	1.0000	0.1479
S8_329384	8	329384	S8_329405	1	0.021	1.0000	1.0000	1.0000	1.0000	0.1479
S8_329405	8	329405	S8_329440	1	0.035	1.0000	1.0000	1.0000	1.0000	0.1479
S8_5837730	8	5837730	S8_5837754	1	0.024	1.0000	1.0000	1.0000	1.0000	0.1316
S8_20849893	8	20849893	S8_20849894	1	0.001	1.0000	1.0000	1.0000	1.0000	0.1104
S8_5322915	8	5322915	S8_5322918	1	0.003	1.0000	1.0000	1.0000	1.0000	0.1332
S8_1136866	8	1136866	S8_1136867	1	0.001	1.0000	1.0000	1.0000	1.0000	0.2205
S8_13616683	8	13616683	S8_13616700	1	0.017	1.0000	1.0000	1.0000	1.0000	0.0917

S8_22474357	8	22474357	S8_22474358	1	0.001	1.0000	1.0000	1.0000	1.0000	0.1412
S8_3836268	8	3836268	S8_3836284	1	0.016	1.0000	1.0000	1.0000	1.0000	0.2377
S8_17011797	8	17011797	S8_17011798	1	0.001	1.0000	1.0000	1.0000	1.0000	0.1314
S8_8677211	8	8677211	S8_8677228	1	0.017	1.0000	1.0000	1.0000	1.0000	0.2495
S8_23491657	8	23491657	S8_23491678	1	0.021	1.0000	1.0000	1.0000	1.0000	0.2455
S8_23491678	8	23491678	S8_23491704	1	0.026	1.0000	1.0000	1.0000	1.0000	0.2455
S8_2878637	8	2878637	S8_2878647	1	0.01	1.0000	1.0000	1.0000	1.0000	0.0842
S8_17195205	8	17195205	S8_17195206	1	0.001	1.0000	1.0000	1.0000	1.0000	0.1354
S8_16940645	8	16940645	S8_16940653	1	0.008	1.0000	1.0000	1.0000	1.0000	0.2488
S8_2671645	8	2671645	S8_2671656	1	0.011	1.0000	1.0000	1.0000	1.0000	0.2497
S8_3346186	8	3346186	S8_3346203	1	0.017	1.0000	1.0000	1.0000	1.0000	0.0689
S8_5710312	8	5710312	S8_5710318	1	0.006	1.0000	1.0000	1.0000	1.0000	0.2483
S8_5710318	8	5710318	S8_5710321	1	0.003	1.0000	1.0000	1.0000	1.0000	0.2483
S8_5710321	8	5710321	S8_5710348	1	0.027	1.0000	1.0000	1.0000	1.0000	0.2483
S8_4805337	8	4805337	S8_4805338	1	0.001	1.0000	1.0000	1.0000	1.0000	0.2292
S8_4450717	8	4450717	S8_4450720	1	0.003	1.0000	1.0000	1.0000	1.0000	0.2294
S8_6462306	8	6462306	S8_6462307	1	0.001	1.0000	1.0000	1.0000	1.0000	0.2357
S8_6462307	8	6462307	S8_6462308	1	0.001	1.0000	1.0000	1.0000	1.0000	0.2357
S8_859273	8	859273	S8_859282	1	0.009	1.0000	1.0000	1.0000	1.0000	0.0800
S8_6243138	8	6243138	S8_6243141	1	0.003	1.0000	1.0000	1.0000	1.0000	0.2487
S8_23229900	8	23229900	S8_23229908	1	0.008	1.0000	1.0000	1.0000	1.0000	0.2245
S8_4744506	8	4744506	S8_4744508	1	0.002	1.0000	1.0000	1.0000	1.0000	0.1606
S8_4744508	8	4744508	S8_4744511	1	0.003	1.0000	1.0000	1.0000	1.0000	0.1606
S8_4744511	8	4744511	S8_4744514	1	0.003	1.0000	1.0000	1.0000	1.0000	0.1606
S8_408751	8	408751	S8_408752	1	0.001	1.0000	1.0000	1.0000	1.0000	0.2432
S8_4261174	8	4261174	S8_4261175	1	0.001	1.0000	1.0000	1.0000	1.0000	0.0850
S8_19664737	8	19664737	S8_19664752	1	0.015	1.0000	1.0000	1.0000	1.0000	0.1005
S8_3346083	8	3346083	S8_3346093	2	0.01	1.0000	1.0000	1.0000	1.0000	0.2473
S8_18054923	8	18054923	S8_18054924	1	0.001	1.0000	1.0000	1.0000	1.0000	0.0969
S8_18054924	8	18054924	S8_18054925	1	0.001	1.0000	1.0000	1.0000	1.0000	0.0969

S8_18054925	8	18054925	S8_18054930	1	0.005	1.0000	1.0000	1.0000	1.0000	0.0969
S8_23259454	8	23259454	S8_23259477	1	0.023	1.0000	1.0000	1.0000	1.0000	0.2272
S8_3342265	8	3342265	S8_3342267	1	0.002	1.0000	1.0000	1.0000	1.0000	0.2398
S8_21492521	8	21492521	S8_21492522	1	0.001	1.0000	1.0000	1.0000	1.0000	0.2477
S8_8496900	8	8496900	S8_8499120	1	2.22	0.9793	1.0000	0.9892	1.0000	0.2445
S8_21492522	8	21492522	S8_21492523	1	0.001	0.9785	1.0000	0.9387	0.9794	0.2402
S8_2316226	8	2316226	S8_2316328	1	0.102	0.9783	1.0000	0.9892	1.0000	0.2382
S8_312130	8	312130	S8_312149	1	0.019	0.9693	1.0000	0.9835	1.0000	0.1638
S8_16561801	8	16561801	S8_16609647	2	47.846	0.9674	1.0000	0.9829	1.0000	0.2166
S8_172140	8	172140	S8_186817	1	14.677	0.9668	1.0000	0.9826	1.0000	0.1514
S8_186817	8	186817	S8_196583	1	9.766	0.9582	1.0000	0.9790	1.0000	0.1448
S8_14758503	8	14758503	S8_14760052	1	1.549	0.9565	1.0000	0.9560	0.9997	0.2362
S8_16251233	8	16251233	S8_16251323	2	0.09	0.9564	1.0000	0.9778	1.0000	0.1289
S8_8179977	8	8179977	S8_8180039	1	0.062	0.9552	0.9773	0.9764	0.9881	0.2250
S8_8485814	8	8485814	S8_8486571	1	0.757	0.9537	1.0000	0.9761	1.0000	0.2396
S8_4910084	8	4910084	S8_4911306	3	1.222	0.9498	1.0000	0.9736	1.0000	0.1994
S8_5096181	8	5096181	S8_5096292	1	0.111	0.9474	1.0000	0.9724	1.0000	0.1184
S8_8133583	8	8133583	S8_8134963	1	1.38	0.9466	1.0000	0.9723	1.0000	0.1951
S8_4490771	8	4490771	S8_4490776	1	0.005	0.9445	1.0000	0.9377	0.9964	0.0883
S8_16609647	8	16609647	S8_16615408	1	5.761	0.9434	1.0000	0.9702	1.0000	0.2063
S8_5710291	8	5710291	S8_5710312	1	0.021	0.9398	1.0000	0.9683	1.0000	0.2448
S8_8499120	8	8499120	S8_8499256	2	0.136	0.9380	0.9787	0.9676	0.9941	0.2412
S8_8485676	8	8485676	S8_8485814	1	0.138	0.9371	0.9784	0.9669	0.9938	0.2402
S8_859282	8	859282	S8_859440	2	0.158	0.9276	1.0000	0.9609	1.0000	0.0733
S8_8596289	8	8596289	S8_8596334	1	0.045	0.9262	1.0000	0.9621	1.0000	0.2040
S8_17195206	8	17195206	S8_17195268	1	0.062	0.9245	0.9615	0.9596	0.9796	0.1326
S8_23578708	8	23578708	S8_23670544	7	91.836	0.9179	1.0000	0.9590	1.0000	0.0601
S8_5837517	8	5837517	S8_5837569	1	0.052	0.9175	0.9783	0.9550	0.9981	0.2405
S8_8677228	8	8677228	S8_8677655	1	0.427	0.9146	1.0000	0.9346	1.0000	0.2405
S8_8486667	8	8486667	S8_8496900	2	10.233	0.9145	0.9563	0.9543	0.9769	0.2380

S8_8486571	8	8486571	S8_8486667	1	0.096	0.9057	0.9751	0.9255	0.9857	0.2330
S8_5373587	8	5373587	S8_5381241	1	7.654	0.9056	1.0000	0.9203	1.0000	0.1495
S8_2633472	8	2633472	S8_2633533	1	0.061	0.9039	0.9508	0.9476	0.9734	0.2040
S8_20849894	8	20849894	S8_20893478	1	43.584	0.9029	0.9502	0.9490	0.9741	0.1075
S8_22216940	8	22216940	S8_22216964	1	0.024	0.8981	1.0000	0.9048	1.0000	0.2280
S8_6125092	8	6125092	S8_6125200	1	0.108	0.8977	1.0000	0.9482	1.0000	0.1288
S8_14197720	8	14197720	S8_14202528	1	4.808	0.8959	0.9777	0.9452	1.0000	0.2421
S8_1494116	8	1494116	S8_1494165	1	0.049	0.8952	0.9462	0.9454	0.9723	0.0992
S8_336300	8	336300	S8_336420	1	0.12	0.8909	1.0000	0.9078	1.0000	0.1374
S8_8809574	8	8809574	S8_8809575	1	0.001	0.8889	0.9609	0.8644	0.9475	0.1264
S8_5837754	8	5837754	S8_5839144	2	1.39	0.8889	1.0000	0.9431	1.0000	0.1330
S8_23834259	8	23834259	S8_23904383	2	70.124	0.8876	0.9700	0.9114	0.9829	0.2100
S8_3833920	8	3833920	S8_3836268	1	2.348	0.8869	0.9528	0.9171	0.9689	0.2282
S8_6464624	8	6464624	S8_6464985	2	0.361	0.8820	0.9507	0.9390	0.9809	0.2185
S8_17079996	8	17079996	S8_17195205	2	115.209	0.8793	1.0000	0.9399	1.0000	0.1242
S8_1254192	8	1254192	S8_1255335	1	1.143	0.8770	0.9566	0.8942	0.9659	0.1357
S8_16615408	8	16615408	S8_16619432	1	4.024	0.8765	1.0000	0.9096	1.0000	0.1964
S8_5616976	8	5616976	S8_5623204	1	6.228	0.8748	0.9475	0.9089	0.9658	0.2272
S8_14171827	8	14171827	S8_14171934	1	0.107	0.8683	0.9318	0.9318	0.9653	0.0737
S8_2551864	8	2551864	S8_2551950	2	0.086	0.8672	0.9442	0.9261	0.9757	0.1804
S8_8567675	8	8567675	S8_8567741	1	0.066	0.8669	0.9528	0.9306	0.9872	0.1433
S8_5322918	8	5322918	S8_5324316	1	1.398	0.8608	0.9505	0.9276	0.9867	0.1262
S8_17828695	8	17828695	S8_17865920	1	37.225	0.8600	0.9502	0.9212	0.9834	0.1040
S8_21719128	8	21719128	S8_21719163	1	0.035	0.8561	1.0000	0.8534	0.9984	0.1291
S8_408752	8	408752	S8_409276	1	0.524	0.8552	1.0000	0.8572	1.0000	0.2274
S8_8809575	8	8809575	S8_8809577	1	0.002	0.8539	1.0000	0.8243	0.9825	0.1184
S8_1213985	8	1213985	S8_1221818	1	7.833	0.8535	0.9380	0.8923	0.9591	0.1665
S8_2551950	8	2551950	S8_2558861	1	6.911	0.8524	1.0000	0.8670	1.0000	0.1801
S8_2316328	8	2316328	S8_2322026	1	5.698	0.8517	0.9541	0.8612	0.9594	0.2201
S8_6895515	8	6895515	S8_6895521	1	0.006	0.8439	1.0000	0.8374	0.9961	0.1210

S8_6895521	8	6895521	S8_6895530	1	0.009	0.8439	1.0000	0.8374	0.9961	0.1210
S8_220621	8	220621	S8_235591	1	14.97	0.8402	0.9560	0.8782	0.9774	0.1390
S8_18763862	8	18763862	S8_18763877	1	0.015	0.8364	1.0000	0.8293	0.9958	0.0553
S8_20559515	8	20559515	S8_20559547	1	0.032	0.8305	1.0000	0.8455	1.0000	0.1565
S8_2818844	8	2818844	S8_2818921	1	0.077	0.8302	0.9111	0.9049	0.9513	0.0552
S8_329440	8	329440	S8_336300	1	6.86	0.8300	1.0000	0.8448	1.0000	0.1310
S8_4890203	8	4890203	S8_4910084	2	19.881	0.8285	0.9220	0.8544	0.9363	0.1879
S8_1573926	8	1573926	S8_1573956	1	0.03	0.8280	1.0000	0.8242	0.9977	0.1157
S8_23280855	8	23280855	S8_23280921	1	0.066	0.8271	0.9303	0.8586	0.9478	0.2109
S8_14386456	8	14386456	S8_14440342	1	53.886	0.8169	0.9489	0.8375	0.9608	0.2186
S8_2085144	8	2085144	S8_2085164	1	0.02	0.8130	1.0000	0.8099	0.9981	0.1400
S8_3346203	8	3346203	S8_3346233	1	0.03	0.8093	1.0000	0.8185	1.0000	0.0681
S8_3346233	8	3346233	S8_3346236	1	0.003	0.8093	1.0000	0.8185	1.0000	0.0681
S8_1490557	8	1490557	S8_1494116	1	3.559	0.8066	0.9457	0.8499	0.9707	0.0960
S8_196583	8	196583	S8_207031	1	10.448	0.8051	0.9287	0.8964	0.9800	0.0996
S8_7054109	8	7054109	S8_7055401	1	1.292	0.8046	0.9205	0.8237	0.9313	0.1890
S8_5071323	8	5071323	S8_5086050	3	14.727	0.7966	0.8925	0.8569	0.9257	0.1360
S8_8454519	8	8454519	S8_8481454	1	26.935	0.7958	0.9317	0.8285	0.9507	0.2157
S8_207031	8	207031	S8_220621	1	13.59	0.7945	0.9418	0.8428	0.9700	0.1068
S8_23259477	8	23259477	S8_23276145	1	16.668	0.7912	0.9516	0.8186	0.9679	0.2093
S8_23280921	8	23280921	S8_23287645	5	6.724	0.7895	0.9462	0.8337	0.9723	0.2110
S8_407789	8	407789	S8_408751	1	0.962	0.7894	0.9293	0.8187	0.9463	0.2215
S8_123227	8	123227	S8_123262	1	0.035	0.7832	1.0000	0.7961	1.0000	0.1371
S8_1494165	8	1494165	S8_1494349	1	0.184	0.7708	0.9341	0.8210	0.9640	0.0831
S8_21665318	8	21665318	S8_21665434	1	0.116	0.7708	0.8779	0.8200	0.9056	0.1075
S8_6326878	8	6326878	S8_6327050	1	0.172	0.7604	1.0000	0.7701	1.0000	0.1165
S8_5487248	8	5487248	S8_5494704	2	7.456	0.7599	1.0000	0.7599	1.0000	0.0673
S8_23122211	8	23122211	S8_23125253	1	3.042	0.7590	0.9097	0.8608	0.9688	0.0632
S8_1339133	8	1339133	S8_1348701	2	9.568	0.7571	0.9512	0.7901	0.9718	0.1038
S8_8809577	8	8809577	S8_8809578	1	0.001	0.7550	0.9498	0.7180	0.9262	0.0980

S8_646666	8	646666	S8_678802	3	32.136	0.7420	0.9024	0.8559	0.9692	0.0553
S8_22704205	8	22704205	S8_22704219	1	0.014	0.7399	1.0000	0.7445	1.0000	0.1097
S8_154155	8	154155	S8_154195	1	0.04	0.7331	1.0000	0.7554	1.0000	0.1186
S8_11747278	8	11747278	S8_11747320	1	0.042	0.7307	1.0000	0.7386	1.0000	0.1969
S8_8391544	8	8391544	S8_8395532	1	3.988	0.7117	0.9291	0.8520	1.0000	0.0896
S8_21675763	8	21675763	S8_21689519	1	13.756	0.7109	1.0000	0.7537	1.0000	0.0873
S8_5455330	8	5455330	S8_5487213	1	31.883	0.7025	1.0000	0.7025	1.0000	0.0738
S8_21689519	8	21689519	S8_21719128	3	29.609	0.6993	0.8363	0.7289	0.8537	0.1133
S8_4303552	8	4303552	S8_4314363	1	10.811	0.6957	1.0000	0.7288	1.0000	0.1761
S8_1494349	8	1494349	S8_1541486	1	47.137	0.6925	0.8805	0.7765	0.9324	0.0886
S8_9992964	8	9992964	S8_10003037	1	10.073	0.6905	0.9490	0.7335	0.9782	0.2095
S8_6291150	8	6291150	S8_6326878	1	35.728	0.6863	0.9136	0.6991	0.9221	0.1123
S8_6827621	8	6827621	S8_6845491	2	17.87	0.6861	0.9329	0.7291	0.9617	0.0843
S8_14440342	8	14440342	S8_14442202	2	1.86	0.6832	1.0000	0.7119	1.0000	0.1943
S8_18515778	8	18515778	S8_18515793	1	0.015	0.6687	1.0000	0.6560	0.9904	0.0515
S8_123262	8	123262	S8_154155	1	30.893	0.6654	0.9540	0.6925	0.9733	0.1176
S8_24022489	8	24022489	S8_24107075	1	84.586	0.6645	0.9362	0.6815	0.9481	0.0835
S8_6980608	8	6980608	S8_7023749	1	43.141	0.6592	0.8431	0.6842	0.8590	0.0652
S8_16190621	8	16190621	S8_16251233	1	60.612	0.6564	0.9245	0.7023	0.9563	0.0782
S8_17195352	8	17195352	S8_17195354	1	0.002	0.6540	1.0000	0.6801	1.0000	0.1292
S8_18147331	8	18147331	S8_18246933	2	99.602	0.6500	1.0000	0.8044	1.0000	0.0492
S8_6327050	8	6327050	S8_6342669	1	15.619	0.6444	0.8579	0.6889	0.8870	0.1040
S8_1221911	8	1221911	S8_1226471	1	4.56	0.6403	0.9059	0.7327	0.9690	0.0771
S8_21719163	8	21719163	S8_21721599	1	2.436	0.6310	0.9420	0.6783	0.9767	0.1079
S8_17195268	8	17195268	S8_17195352	1	0.084	0.6297	1.0000	0.6695	1.0000	0.1265
S8_235591	8	235591	S8_312130	1	76.539	0.6283	0.8726	0.6863	0.9120	0.1312
S8_9990658	8	9990658	S8_9990696	2	0.038	0.5724	1.0000	0.5963	1.0000	0.1726
S8_18696582	8	18696582	S8_18750115	9	53.533	0.5640	0.9177	0.6693	0.9998	0.0800
S8_10232237	8	10232237	S8_10239335	1	7.098	0.5437	0.9645	0.5721	0.9893	0.1638
S8_5064055	8	5064055	S8_5064752	1	0.697	0.5374	1.0000	0.5540	1.0000	0.0983

S8_6265636	8	6265636	S8_6291150	2	25.514	0.5186	0.8661	0.5362	0.8807	0.1038
S8_5064752	8	5064752	S8_5071323	1	6.571	0.5098	1.0000	0.5447	1.0000	0.0849
S8_23491704	8	23491704	S8_23491706	1	0.002	0.5050	0.9999	0.3764	0.8633	0.1362
S8_6244444	8	6244444	S8_6265636	1	21.192	0.4108	0.8433	0.4249	0.8576	0.0874
S8_9990696	8	9990696	S8_9992964	1	2.268	0.4011	0.9641	0.4396	1.0000	0.1508
S8_9256764	8	9256764	S8_9256783	1	0.019	0.3898	0.9998	0.1617	0.6439	-0.0940
S8_16854404	8	16854404	S8_16854422	1	0.018	0.2991	1.0000	0.2846	0.9754	0.1054
S8_16940653	8	16940653	S8_17011797	1	71.144	0.2306	0.9363	0.2536	0.9818	0.0940
S9_1109849	9	1109849	S9_1109867	1	0.018	1.0000	1.0000	1.0000	1.0000	0.0871
S9_1732810	9	1732810	S9_1732869	1	0.059	1.0000	1.0000	1.0000	1.0000	0.2317
S9_1732869	9	1732869	S9_1732892	1	0.023	1.0000	1.0000	1.0000	1.0000	0.2317
S9_4223533	9	4223533	S9_4258181	2	34.648	1.0000	1.0000	1.0000	1.0000	0.0900
S9_4258181	9	4258181	S9_4258281	1	0.1	1.0000	1.0000	1.0000	1.0000	0.0866
S9_20497046	9	20497046	S9_20497047	1	0.001	1.0000	1.0000	1.0000	1.0000	0.1024
S9_21505138	9	21505138	S9_21505188	1	0.05	1.0000	1.0000	1.0000	1.0000	0.1701
S9_21652254	9	21652254	S9_21652434	1	0.18	1.0000	1.0000	1.0000	1.0000	0.0678
S9_22897125	9	22897125	S9_22897208	1	0.083	1.0000	1.0000	1.0000	1.0000	0.1114
S9_4308164	9	4308164	S9_4308198	1	0.034	1.0000	1.0000	1.0000	1.0000	0.2298
S9_19509850	9	19509850	S9_19509851	1	0.001	1.0000	1.0000	1.0000	1.0000	0.2322
S9_19509851	9	19509851	S9_19509852	1	0.001	1.0000	1.0000	1.0000	1.0000	0.2322
S9_779650	9	779650	S9_779667	1	0.017	1.0000	1.0000	1.0000	1.0000	0.0694
S9_768453	9	768453	S9_768470	1	0.017	1.0000	1.0000	1.0000	1.0000	0.2436
S9_1556691	9	1556691	S9_1556692	1	0.001	1.0000	1.0000	1.0000	1.0000	0.1343
S9_1545309	9	1545309	S9_1556691	1	11.382	1.0000	1.0000	1.0000	1.0000	0.1456
S9_1143401	9	1143401	S9_1143456	1	0.055	1.0000	1.0000	1.0000	1.0000	0.1174
S9_17455149	9	17455149	S9_17455171	1	0.022	1.0000	1.0000	1.0000	1.0000	0.2009
S9_504661	9	504661	S9_504670	1	0.009	1.0000	1.0000	1.0000	1.0000	0.2303
S9_21738139	9	21738139	S9_21738140	1	0.001	1.0000	1.0000	1.0000	1.0000	0.1913
S9_2556989	9	2556989	S9_2556996	1	0.007	1.0000	1.0000	1.0000	1.0000	0.0722
S9_860999	9	860999	S9_861010	1	0.011	1.0000	1.0000	1.0000	1.0000	0.2490

S9_861010	9	861010	S9_861028	1	0.018	1.0000	1.0000	1.0000	1.0000	0.2490
S9_23943308	9	23943308	S9_23943320	1	0.012	1.0000	1.0000	1.0000	1.0000	0.1679
S9_4535923	9	4535923	S9_4535924	1	0.001	1.0000	1.0000	1.0000	1.0000	0.1445
S9_20777991	9	20777991	S9_20777992	1	0.001	1.0000	1.0000	1.0000	1.0000	0.0871
S9_20430902	9	20430902	S9_20430903	1	0.001	1.0000	1.0000	1.0000	1.0000	0.0548
S9_20776433	9	20776433	S9_20776438	1	0.005	1.0000	1.0000	1.0000	1.0000	0.2240
S9_20714401	9	20714401	S9_20714418	1	0.017	1.0000	1.0000	1.0000	1.0000	0.2468
S9_20714418	9	20714418	S9_20714420	1	0.002	1.0000	1.0000	1.0000	1.0000	0.2468
S9_17576318	9	17576318	S9_17576327	1	0.009	1.0000	1.0000	1.0000	1.0000	0.2391
S9_19488105	9	19488105	S9_19488129	1	0.024	1.0000	1.0000	1.0000	1.0000	0.2334
S9_6251637	9	6251637	S9_6251656	1	0.019	1.0000	1.0000	1.0000	1.0000	0.1607
S9_13639097	9	13639097	S9_13639103	1	0.006	1.0000	1.0000	1.0000	1.0000	0.2382
S9_8431491	9	8431491	S9_8431492	1	0.001	1.0000	1.0000	1.0000	1.0000	0.2130
S9_9421604	9	9421604	S9_9421630	1	0.026	1.0000	1.0000	1.0000	1.0000	0.1153
S9_23744870	9	23744870	S9_23744881	1	0.011	1.0000	1.0000	1.0000	1.0000	0.2496
S9_21203130	9	21203130	S9_21203131	1	0.001	1.0000	1.0000	1.0000	1.0000	0.1637
S9_20589016	9	20589016	S9_20589018	1	0.002	1.0000	1.0000	1.0000	1.0000	0.2292
S9_20767085	9	20767085	S9_20767111	1	0.026	1.0000	1.0000	1.0000	1.0000	0.2280
S9_17455120	9	17455120	S9_17455123	1	0.003	1.0000	1.0000	1.0000	1.0000	0.1901
S9_20714466	9	20714466	S9_20714554	1	0.088	1.0000	1.0000	1.0000	1.0000	0.2473
S9_151390	9	151390	S9_151405	1	0.015	1.0000	1.0000	1.0000	1.0000	0.1171
S9_22033231	9	22033231	S9_22033240	1	0.009	1.0000	1.0000	1.0000	1.0000	0.1084
S9_18461607	9	18461607	S9_18461647	1	0.04	1.0000	1.0000	1.0000	1.0000	0.1444
S9_20430701	9	20430701	S9_20430712	1	0.011	1.0000	1.0000	1.0000	1.0000	0.1988
S9_17455205	9	17455205	S9_17455216	1	0.011	1.0000	1.0000	1.0000	1.0000	0.2009
S9_17455216	9	17455216	S9_17455236	1	0.02	1.0000	1.0000	1.0000	1.0000	0.2009
S9_17455236	9	17455236	S9_17455248	1	0.012	1.0000	1.0000	1.0000	1.0000	0.2009
S9_641350	9	641350	S9_641362	1	0.012	1.0000	1.0000	1.0000	1.0000	0.2500
S9_20776473	9	20776473	S9_20776474	1	0.001	1.0000	1.0000	1.0000	1.0000	0.2205
S9_20776474	9	20776474	S9_20776517	1	0.043	1.0000	1.0000	1.0000	1.0000	0.2205

S9_779757	9	779757	S9_779764	1	0.007	1.0000	1.0000	1.0000	1.0000	0.1084
S9_4653828	9	4653828	S9_4653830	1	0.002	1.0000	1.0000	1.0000	1.0000	0.2455
S9_7995766	9	7995766	S9_7996638	1	0.872	1.0000	1.0000	1.0000	1.0000	0.1445
S9_8691945	9	8691945	S9_8691946	1	0.001	1.0000	1.0000	1.0000	1.0000	0.1836
S9_20762843	9	20762843	S9_20762858	1	0.015	1.0000	1.0000	1.0000	1.0000	0.2267
S9_13479997	9	13479997	S9_13479999	1	0.002	1.0000	1.0000	1.0000	1.0000	0.0842
S9_13479999	9	13479999	S9_13480000	1	0.001	1.0000	1.0000	1.0000	1.0000	0.0842
S9_811474	9	811474	S9_811486	1	0.012	1.0000	1.0000	1.0000	1.0000	0.0883
S9_304014	9	304014	S9_304038	1	0.024	1.0000	1.0000	1.0000	1.0000	0.1568
S9_6087817	9	6087817	S9_6087820	1	0.003	1.0000	1.0000	1.0000	1.0000	0.0598
S9_22113253	9	22113253	S9_22113557	2	0.304	0.9792	1.0000	0.9892	1.0000	0.2467
S9_4670131	9	4670131	S9_4670153	1	0.022	0.9790	1.0000	0.9890	1.0000	0.2466
S9_23118141	9	23118141	S9_23118178	1	0.037	0.9787	1.0000	0.9889	1.0000	0.2485
S9_15617413	9	15617413	S9_15617461	1	0.048	0.9787	1.0000	0.9890	1.0000	0.2404
S9_22859273	9	22859273	S9_22859387	1	0.114	0.9786	1.0000	0.9892	1.0000	0.2449
S9_19098398	9	19098398	S9_19098424	1	0.026	0.9780	1.0000	0.9883	1.0000	0.2323
S9_15732341	9	15732341	S9_15773062	1	40.721	0.9777	1.0000	0.9886	1.0000	0.2320
S9_545437	9	545437	S9_545530	1	0.093	0.9776	1.0000	0.9876	1.0000	0.2281
S9_20089889	9	20089889	S9_20089945	3	0.056	0.9745	1.0000	0.9868	1.0000	0.2045
S9_8691946	9	8691946	S9_8691986	1	0.04	0.9725	1.0000	0.9851	1.0000	0.1835
S9_21737950	9	21737950	S9_21738139	1	0.189	0.9714	1.0000	0.9854	1.0000	0.1821
S9_20251540	9	20251540	S9_20252472	2	0.932	0.9655	1.0000	0.9823	1.0000	0.1810
S9_16258884	9	16258884	S9_16263582	1	4.698	0.9627	1.0000	0.9812	1.0000	0.1531
S9_177091	9	177091	S9_177123	1	0.032	0.9591	1.0000	0.9783	1.0000	0.1330
S9_20714420	9	20714420	S9_20714466	1	0.046	0.9583	0.9789	0.9780	0.9889	0.2446
S9_16443347	9	16443347	S9_16443399	1	0.052	0.9578	1.0000	0.9781	1.0000	0.1304
S9_15617461	9	15617461	S9_15617632	1	0.171	0.9571	0.9783	0.9777	0.9888	0.2375
S9_545323	9	545323	S9_545437	1	0.114	0.9563	1.0000	0.9755	1.0000	0.2278
S9_5057080	9	5057080	S9_5057152	1	0.072	0.9560	1.0000	0.9771	1.0000	0.2470
S9_1143456	9	1143456	S9_1149544	1	6.088	0.9554	1.0000	0.9768	1.0000	0.1191

S9_5724665	9	5724665	S9_5724668	1	0.003	0.9546	0.9770	0.9759	0.9879	0.2217
S9_13475306	9	13475306	S9_13476479	1	1.173	0.9539	1.0000	0.9753	1.0000	0.2297
S9_20714554	9	20714554	S9_20714644	1	0.09	0.9497	0.9745	0.9735	0.9867	0.2420
S9_3141873	9	3141873	S9_3141881	1	0.008	0.9476	1.0000	0.9738	1.0000	0.2013
S9_13483685	9	13483685	S9_13484202	1	0.517	0.9470	0.9731	0.9722	0.9860	0.2384
S9_23442459	9	23442459	S9_23445705	1	3.246	0.9466	1.0000	0.9727	1.0000	0.0976
S9_4259571	9	4259571	S9_4260860	1	1.289	0.9357	1.0000	0.9670	1.0000	0.0787
S9_9232996	9	9232996	S9_9235284	1	2.288	0.9332	1.0000	0.9657	1.0000	0.1546
S9_17550491	9	17550491	S9_17555469	5	4.978	0.9305	0.9761	0.9631	0.9930	0.2212
S9_8427971	9	8427971	S9_8431491	1	3.52	0.9248	1.0000	0.9603	1.0000	0.2089
S9_21733484	9	21733484	S9_21734177	1	0.693	0.9237	1.0000	0.9615	1.0000	0.1314
S9_9417417	9	9417417	S9_9417707	2	0.29	0.9220	1.0000	0.9599	1.0000	0.1256
S9_17575809	9	17575809	S9_17575859	1	0.05	0.9179	1.0000	0.9590	1.0000	0.0596
S9_1109867	9	1109867	S9_1109906	1	0.039	0.9163	1.0000	0.9566	1.0000	0.0756
S9_1149544	9	1149544	S9_1166898	1	17.354	0.9128	0.9554	0.9539	0.9767	0.1154
S9_1540368	9	1540368	S9_1545309	1	4.941	0.9112	0.9546	0.9540	0.9768	0.1472
S9_18460999	9	18460999	S9_18461607	1	0.608	0.9065	0.9521	0.9510	0.9752	0.1340
S9_8692016	9	8692016	S9_8692057	2	0.041	0.9044	1.0000	0.9166	1.0000	0.1552
S9_303830	9	303830	S9_303961	1	0.131	0.9032	0.9662	0.9457	0.9887	0.1528
S9_16263582	9	16263582	S9_16263583	1	0.001	0.9009	1.0000	0.9480	1.0000	0.1626
S9_22531898	9	22531898	S9_22531924	1	0.026	0.8985	1.0000	0.8930	0.9969	0.0919
S9_303961	9	303961	S9_304014	1	0.053	0.8965	0.9638	0.9409	0.9874	0.1453
S9_18814147	9	18814147	S9_18814174	1	0.027	0.8961	1.0000	0.8948	0.9992	0.1796
S9_20834611	9	20834611	S9_20834689	1	0.078	0.8943	1.0000	0.9442	1.0000	0.1388
S9_6478721	9	6478721	S9_6485739	2	7.018	0.8922	1.0000	0.9121	1.0000	0.1726
S9_18460952	9	18460952	S9_18460999	1	0.047	0.8824	0.9586	0.9385	0.9886	0.1327
S9_9417371	9	9417371	S9_9417417	1	0.046	0.8787	0.9572	0.8973	0.9673	0.1285
S9_19098424	9	19098424	S9_19098532	1	0.108	0.8782	0.9736	0.9370	1.0000	0.2091
S9_19098532	9	19098532	S9_19100567	1	2.035	0.8777	1.0000	0.9126	1.0000	0.2080
S9_4653830	9	4653830	S9_4670131	3	16.301	0.8776	0.9777	0.9353	1.0000	0.2384

S9_1109906	9	1109906	S9_1143401	1	33.495	0.8737	0.9347	0.9337	0.9663	0.0861
S9_811486	9	811486	S9_811614	1	0.128	0.8727	0.9342	0.9271	0.9629	0.0904
S9_13484202	9	13484202	S9_13484301	2	0.099	0.8719	0.9720	0.9323	1.0000	0.2339
S9_9316293	9	9316293	S9_9316338	1	0.045	0.8715	1.0000	0.9325	1.0000	0.1808
S9_9446159	9	9446159	S9_9452230	1	6.071	0.8710	0.9544	0.9332	0.9878	0.1259
S9_9427009	9	9427009	S9_9446159	1	19.15	0.8706	1.0000	0.8898	1.0000	0.1201
S9_168205	9	168205	S9_170704	1	2.499	0.8705	0.9541	0.9290	0.9857	0.1280
S9_16140442	9	16140442	S9_16140564	1	0.122	0.8697	0.9538	0.8870	0.9633	0.1470
S9_9417707	9	9417707	S9_9421604	1	3.897	0.8687	0.9535	0.9301	0.9866	0.1141
S9_22154572	9	22154572	S9_22159336	1	4.764	0.8674	0.9530	0.9302	0.9869	0.1206
S9_20762720	9	20762720	S9_20762761	1	0.041	0.8637	0.9701	0.9281	1.0000	0.2052
S9_3466385	9	3466385	S9_3468796	2	2.411	0.8613	0.9415	0.8985	0.9616	0.1714
S9_19697053	9	19697053	S9_19715019	1	17.966	0.8590	0.9739	0.8782	0.9847	0.2224
S9_15617632	9	15617632	S9_15732341	2	114.709	0.8527	0.9764	0.8806	0.9922	0.2213
S9_1327	9	1327	S9_1391	1	0.064	0.8493	0.9461	0.9177	0.9834	0.1986
S9_9452230	9	9452230	S9_9452297	1	0.067	0.8481	0.9209	0.9160	0.9571	0.1262
S9_20776444	9	20776444	S9_20776473	1	0.029	0.8458	0.9447	0.9167	0.9835	0.2002
S9_8692057	9	8692057	S9_8715989	1	23.932	0.8397	0.9643	0.8861	0.9905	0.1690
S9_22057864	9	22057864	S9_22093357	2	35.493	0.8393	1.0000	0.8629	1.0000	0.0981
S9_540689	9	540689	S9_540700	1	0.011	0.8369	1.0000	0.9172	1.0000	0.0554
S9_304038	9	304038	S9_310308	2	6.27	0.8340	0.9627	0.8701	0.9834	0.1488
S9_151510	9	151510	S9_167601	1	16.091	0.8338	0.9480	0.8874	0.9780	0.2141
S9_16212944	9	16212944	S9_16258884	3	45.94	0.8322	1.0000	0.8442	1.0000	0.1388
S9_88935	9	88935	S9_151390	1	62.455	0.8297	0.9109	0.9030	0.9502	0.1113
S9_6087775	9	6087775	S9_6087817	1	0.042	0.8294	0.9107	0.8916	0.9443	0.0570
S9_1391	9	1391	S9_35658	3	34.267	0.8271	0.9213	0.8779	0.9491	0.1929
S9_15034871	9	15034871	S9_15040549	1	5.678	0.8241	1.0000	0.8495	1.0000	0.0915
S9_17575859	9	17575859	S9_17575879	1	0.02	0.8216	1.0000	0.9033	1.0000	0.0635
S9_4462963	9	4462963	S9_4465017	1	2.054	0.8215	0.9503	0.9006	0.9950	0.1237
S9_9421630	9	9421630	S9_9427009	1	5.379	0.8191	0.9496	0.9016	0.9962	0.1086

S9_310336	9	310336	S9_343084	1	32.748	0.8086	0.9392	0.8388	0.9566	0.1754
S9_8715989	9	8715989	S9_8741703	1	25.714	0.8083	0.9631	0.8383	0.9808	0.1641
S9_19100567	9	19100567	S9_19106241	1	5.674	0.8048	0.9743	0.8294	0.9891	0.2074
S9_24058424	9	24058424	S9_24105992	4	47.568	0.7983	0.9608	0.8873	1.0000	0.1441
S9_3876843	9	3876843	S9_4014494	3	137.651	0.7908	0.9646	0.8543	1.0000	0.1843
S9_14237173	9	14237173	S9_14237181	1	0.008	0.7900	1.0000	0.8045	1.0000	0.1787
S9_8691986	9	8691986	S9_8692016	1	0.03	0.7887	0.9642	0.8221	0.9844	0.1497
S9_16140564	9	16140564	S9_16212944	3	72.38	0.7801	1.0000	0.7759	0.9973	0.1361
S9_5546816	9	5546816	S9_5559056	5	12.24	0.7774	1.0000	0.7797	1.0000	0.2004
S9_4191489	9	4191489	S9_4223532	2	32.043	0.7753	0.8805	0.8424	0.9178	0.1252
S9_22382080	9	22382080	S9_22382098	1	0.018	0.7746	1.0000	0.7735	0.9993	0.1968
S9_7996638	9	7996638	S9_8031462	2	34.824	0.7591	0.9580	0.8264	0.9996	0.1232
S9_290206	9	290206	S9_303830	2	13.624	0.7586	0.9210	0.8247	0.9603	0.1297
S9_1166898	9	1166898	S9_1166910	1	0.012	0.7424	1.0000	0.7770	1.0000	0.0921
S9_1166910	9	1166910	S9_1166930	1	0.02	0.7424	1.0000	0.7770	1.0000	0.0921
S9_1166930	9	1166930	S9_1167001	1	0.071	0.7424	1.0000	0.7770	1.0000	0.0921
S9_23442367	9	23442367	S9_23442459	1	0.092	0.7405	1.0000	0.7608	1.0000	0.0745
S9_17576343	9	17576343	S9_17580874	3	4.531	0.7213	0.8934	0.8479	0.9686	0.0511
S9_23128498	9	23128498	S9_23242572	2	114.074	0.7163	0.9047	0.7628	0.9336	0.1115
S9_6251546	9	6251546	S9_6251568	1	0.022	0.7145	1.0000	0.7243	1.0000	0.1416
S9_23329822	9	23329822	S9_23442367	4	112.545	0.7007	1.0000	0.7389	1.0000	0.0801
S9_20502687	9	20502687	S9_20502836	1	0.149	0.6988	1.0000	0.6988	1.0000	0.0967
S9_20502836	9	20502836	S9_20502881	1	0.045	0.6988	1.0000	0.6988	1.0000	0.0967
S9_20497047	9	20497047	S9_20502687	2	5.64	0.6980	1.0000	0.6980	1.0000	0.0983
S9_22159336	9	22159336	S9_22254315	5	94.979	0.6863	0.9416	0.7027	0.9528	0.0938
S9_14237181	9	14237181	S9_14277318	1	40.137	0.6826	0.9317	0.6929	0.9387	0.1707
S9_545530	9	545530	S9_545554	1	0.024	0.6797	1.0000	0.6947	1.0000	0.1788
S9_151405	9	151405	S9_151492	1	0.087	0.6666	0.8550	0.7644	0.9155	0.0998
S9_7009141	9	7009141	S9_7068485	2	59.344	0.5980	0.9650	0.6497	1.0000	0.1520
S9_4258396	9	4258396	S9_4259479	1	1.083	0.5894	0.8649	0.6483	0.9071	0.0481

S9_20179596	9	20179596	S9_20208649	1	29.053	0.4697	1.0000	0.4774	1.0000	0.1316
S9_23319225	9	23319225	S9_23329822	2	10.597	0.4369	0.8857	0.4479	0.8968	0.0877
S9_23293192	9	23293192	S9_23319225	2	26.033	0.4165	0.8726	0.4459	0.9029	0.0770
S9_19074660	9	19074660	S9_19098398	1	23.738	0.3928	0.9579	0.4056	0.9734	0.1267
S9_23118178	9	23118178	S9_23118186	1	0.008	0.1880	1.0000	0.1985	1.0000	0.0789
S9_23118186	9	23118186	S9_23118202	1	0.016	0.1880	1.0000	0.1985	1.0000	0.0789
S9_4259479	9	4259479	S9_4259571	2	0.092	0.0712	0.9994	0.0312	0.6622	0.0037
S9_4258281	9	4258281	S9_4258396	1	0.115	0.0002	0.0192	0.0003	0.0215	0.0011
S10_68527	10	68527	S10_71248	1	2.721	1.0000	1.0000	1.0000	1.0000	0.1318
S10_71248	10	71248	S10_77384	1	6.136	1.0000	1.0000	1.0000	1.0000	0.1257
S10_91258	10	91258	S10_94131	1	2.873	1.0000	1.0000	1.0000	1.0000	0.1338
S10_94131	10	94131	S10_99499	1	5.368	1.0000	1.0000	1.0000	1.0000	0.1318
S10_77384	10	77384	S10_91258	4	13.874	1.0000	1.0000	1.0000	1.0000	0.1271
S10_111359	10	111359	S10_111370	1	0.011	1.0000	1.0000	1.0000	1.0000	0.1202
S10_111370	10	111370	S10_231901	3	120.531	1.0000	1.0000	1.0000	1.0000	0.1082
S10_11753315	10	11753315	S10_11753330	1	0.015	1.0000	1.0000	1.0000	1.0000	0.2240
S10_1448387	10	1448387	S10_1448401	1	0.014	1.0000	1.0000	1.0000	1.0000	0.2440
S10_11763990	10	11763990	S10_11764010	1	0.02	1.0000	1.0000	1.0000	1.0000	0.1861
S10_14959578	10	14959578	S10_14959592	1	0.014	1.0000	1.0000	1.0000	1.0000	0.1875
S10_7376951	10	7376951	S10_7376997	1	0.046	1.0000	1.0000	1.0000	1.0000	0.1365
S10_6748523	10	6748523	S10_6748526	1	0.003	1.0000	1.0000	1.0000	1.0000	0.1271
S10_2777014	10	2777014	S10_2777015	1	0.001	1.0000	1.0000	1.0000	1.0000	0.1257
S10_2777015	10	2777015	S10_2777016	1	0.001	1.0000	1.0000	1.0000	1.0000	0.1257
S10_5013281	10	5013281	S10_5013289	1	0.008	1.0000	1.0000	1.0000	1.0000	0.0883
S10_1486096	10	1486096	S10_1486105	1	0.009	1.0000	1.0000	1.0000	1.0000	0.0900
S10_3443111	10	3443111	S10_3443138	1	0.027	1.0000	1.0000	1.0000	1.0000	0.1862
S10_15269521	10	15269521	S10_15269555	1	0.034	1.0000	1.0000	1.0000	1.0000	0.0746
S10_9393279	10	9393279	S10_9393284	1	0.005	1.0000	1.0000	1.0000	1.0000	0.2222
S10_9393284	10	9393284	S10_9393316	1	0.032	1.0000	1.0000	1.0000	1.0000	0.2222
S10_5790263	10	5790263	S10_5790276	1	0.013	1.0000	1.0000	1.0000	1.0000	0.1707

S10_1498840	10	1498840	S10_1499219	1	0.379	1.0000	1.0000	1.0000	1.0000	0.1235
S10_1499219	10	1499219	S10_1499276	1	0.057	1.0000	1.0000	1.0000	1.0000	0.1235
S10_4620467	10	4620467	S10_4620504	1	0.037	1.0000	1.0000	1.0000	1.0000	0.1377
S10_1415805	10	1415805	S10_1415860	1	0.055	1.0000	1.0000	1.0000	1.0000	0.1888
S10_1232844	10	1232844	S10_1232862	1	0.018	1.0000	1.0000	1.0000	1.0000	0.2445
S10_1376969	10	1376969	S10_1376981	1	0.012	1.0000	1.0000	1.0000	1.0000	0.1556
S10_14939979	10	14939979	S10_14939980	1	0.001	1.0000	1.0000	1.0000	1.0000	0.2347
S10_724764	10	724764	S10_724795	1	0.031	1.0000	1.0000	1.0000	1.0000	0.1688
S10_747681	10	747681	S10_747696	1	0.015	1.0000	1.0000	1.0000	1.0000	0.1621
S10_3946922	10	3946922	S10_3946931	1	0.009	1.0000	1.0000	1.0000	1.0000	0.2034
S10_3946931	10	3946931	S10_3946964	1	0.033	1.0000	1.0000	1.0000	1.0000	0.2034
S10_746143	10	746143	S10_746144	1	0.001	1.0000	1.0000	1.0000	1.0000	0.0900
S10_746144	10	746144	S10_746145	1	0.001	1.0000	1.0000	1.0000	1.0000	0.0900
S10_3946964	10	3946964	S10_3946988	1	0.024	1.0000	1.0000	1.0000	1.0000	0.1998
S10_3946988	10	3946988	S10_3947000	1	0.012	1.0000	1.0000	1.0000	1.0000	0.1998
S10_12393034	10	12393034	S10_12393035	1	0.001	1.0000	1.0000	1.0000	1.0000	0.1964
S10_12393069	10	12393069	S10_12393092	1	0.023	1.0000	1.0000	1.0000	1.0000	0.1954
S10_2227433	10	2227433	S10_2227434	1	0.001	1.0000	1.0000	1.0000	1.0000	0.1962
S10_3946889	10	3946889	S10_3946894	1	0.005	1.0000	1.0000	1.0000	1.0000	0.2053
S10_5403341	10	5403341	S10_5403382	1	0.041	1.0000	1.0000	1.0000	1.0000	0.1343
S10_604024	10	604024	S10_604043	1	0.019	1.0000	1.0000	1.0000	1.0000	0.0988
S10_604043	10	604043	S10_604071	1	0.028	1.0000	1.0000	1.0000	1.0000	0.0988
S10_3775830	10	3775830	S10_3775848	1	0.018	1.0000	1.0000	1.0000	1.0000	0.1849
S10_9706319	10	9706319	S10_9706320	1	0.001	1.0000	1.0000	1.0000	1.0000	0.1377
S10_9706320	10	9706320	S10_9706321	1	0.001	1.0000	1.0000	1.0000	1.0000	0.1377
S10_14564819	10	14564819	S10_14564820	1	0.001	1.0000	1.0000	1.0000	1.0000	0.2493
S10_13807940	10	13807940	S10_13966767	1	158.827	0.9773	1.0000	0.9882	1.0000	0.2277
S10_4765849	10	4765849	S10_4772501	1	6.652	0.9756	1.0000	0.9869	1.0000	0.2072
S10_2227543	10	2227543	S10_2227568	1	0.025	0.9736	1.0000	0.9860	1.0000	0.1912
S10_14047776	10	14047776	S10_14047809	2	0.033	0.9702	1.0000	0.9843	1.0000	0.1974

S10_5401922	10	5401922	S10_5403341	1	1.419	0.9592	1.0000	0.9774	1.0000	0.1317
S10_13362887	10	13362887	S10_13362917	1	0.03	0.9584	1.0000	0.9777	1.0000	0.1224
S10_1438806	10	1438806	S10_1438871	1	0.065	0.9565	0.9780	0.9773	0.9886	0.2320
S10_4381310	10	4381310	S10_4402874	1	21.564	0.9557	1.0000	0.9782	1.0000	0.1149
S10_5743914	10	5743914	S10_5757812	1	13.898	0.9552	1.0000	0.9767	1.0000	0.2245
S10_1398632	10	1398632	S10_1398683	1	0.051	0.9516	0.9755	0.9746	0.9872	0.2101
S10_13141301	10	13141301	S10_13141311	1	0.01	0.9427	1.0000	0.9409	0.9991	0.1805
S10_11724294	10	11724294	S10_11734542	2	10.248	0.9405	1.0000	0.9387	0.9990	0.1775
S10_1039591	10	1039591	S10_1039597	1	0.006	0.9400	1.0000	0.0002	0.0153	-0.0037
S10_443437	10	443437	S10_443512	2	0.075	0.9389	1.0000	0.9694	1.0000	0.2442
S10_9155852	10	9155852	S10_9156617	1	0.765	0.9369	1.0000	0.9682	1.0000	0.1935
S10_11734542	10	11734542	S10_11734628	2	0.086	0.9342	0.9665	0.9657	0.9827	0.1650
S10_5774449	10	5774449	S10_5790263	1	15.814	0.9338	0.9663	0.9652	0.9824	0.1677
S10_4651214	10	4651214	S10_4651262	1	0.048	0.9331	1.0000	0.9626	1.0000	0.1556
S10_5352241	10	5352241	S10_5352262	1	0.021	0.9326	0.9657	0.9635	0.9816	0.1507
S10_748099	10	748099	S10_757253	1	9.154	0.9303	0.9645	0.9636	0.9816	0.1667
S10_38125	10	38125	S10_68527	1	30.402	0.9259	1.0000	0.9620	1.0000	0.1327
S10_3946894	10	3946894	S10_3946922	1	0.028	0.9244	0.9739	0.9596	0.9923	0.2004
S10_3946736	10	3946736	S10_3946889	1	0.153	0.9236	0.9736	0.9586	0.9919	0.1980
S10_10311344	10	10311344	S10_10311849	1	0.505	0.9202	0.9724	0.9320	0.9786	0.2366
S10_711803	10	711803	S10_711868	1	0.065	0.9180	1.0000	0.9591	1.0000	0.0590
S10_711705	10	711705	S10_711803	1	0.098	0.9179	1.0000	0.9590	1.0000	0.0596
S10_3356695	10	3356695	S10_3357756	1	1.061	0.9169	0.9712	0.9566	0.9920	0.2156
S10_2393585	10	2393585	S10_2393839	1	0.254	0.9169	0.9712	0.9565	0.9920	0.2361
S10_14692420	10	14692420	S10_14692442	1	0.022	0.9145	1.0000	0.9281	1.0000	0.1995
S10_11753361	10	11753361	S10_11763990	1	10.629	0.9129	0.9698	0.9550	0.9919	0.1887
S10_1291780	10	1291780	S10_1291921	1	0.141	0.9099	0.9539	0.9528	0.9761	0.2228
S10_1202206	10	1202206	S10_1203261	2	1.055	0.9019	0.9658	0.9445	0.9883	0.1702
S10_1446217	10	1446217	S10_1448387	2	2.17	0.9016	0.9739	0.9249	0.9865	0.2366
S10_3541676	10	3541676	S10_3541866	1	0.19	0.9007	0.9490	0.9485	0.9739	0.2389

S10_14047711	10	14047711	S10_14047776	1	0.065	0.8996	1.0000	0.9485	1.0000	0.1792
S10_720305	10	720305	S10_724764	1	4.459	0.8982	1.0000	0.9482	1.0000	0.1744
S10_5401860	10	5401860	S10_5401887	1	0.027	0.8969	1.0000	0.9093	1.0000	0.1383
S10_9370771	10	9370771	S10_9393279	2	22.508	0.8957	0.9464	0.9191	0.9587	0.2130
S10_2603419	10	2603419	S10_2603628	1	0.209	0.8935	0.9557	0.9434	0.9820	0.2384
S10_12393035	10	12393035	S10_12393069	1	0.034	0.8934	0.9716	0.9424	0.9979	0.1934
S10_11671672	10	11671672	S10_11724294	2	52.622	0.8908	1.0000	0.9159	1.0000	0.1924
S10_3380555	10	3380555	S10_3380584	1	0.029	0.8899	0.9763	0.8957	0.9795	0.2131
S10_3802858	10	3802858	S10_3806705	3	3.847	0.8822	0.9393	0.9384	0.9687	0.1900
S10_14047809	10	14047809	S10_14047898	1	0.089	0.8805	1.0000	0.9071	1.0000	0.1869
S10_14651936	10	14651936	S10_14692420	1	40.484	0.8805	0.9383	0.8805	0.9383	0.1951
S10_4890428	10	4890428	S10_4890664	1	0.236	0.8751	0.9728	0.8853	0.9785	0.2251
S10_5013289	10	5013289	S10_5018218	1	4.929	0.8708	1.0000	0.8665	0.9975	0.0800
S10_3939171	10	3939171	S10_3946736	2	7.565	0.8687	0.9448	0.9014	0.9624	0.1878
S10_11734711	10	11734711	S10_11736190	1	1.479	0.8680	1.0000	0.8793	1.0000	0.1706
S10_11671624	10	11671624	S10_11671672	1	0.048	0.8667	1.0000	0.8771	1.0000	0.1816
S10_1232666	10	1232666	S10_1232844	1	0.178	0.8642	0.9428	0.9007	0.9625	0.2339
S10_13988557	10	13988557	S10_14047711	1	59.154	0.8640	0.9295	0.8956	0.9463	0.1790
S10_1448401	10	1448401	S10_1448499	1	0.098	0.8600	0.9502	0.9035	0.9739	0.2327
S10_1438871	10	1438871	S10_1441130	1	2.259	0.8556	0.9769	0.9024	1.0000	0.2265
S10_2842851	10	2842851	S10_2848636	3	5.785	0.8516	0.9470	0.8672	0.9556	0.1113
S10_14940060	10	14940060	S10_14940093	1	0.033	0.8488	1.0000	0.8488	1.0000	0.1736
S10_1441130	10	1441130	S10_1446217	3	5.087	0.8477	0.9527	0.8765	0.9688	0.2279
S10_5317405	10	5317405	S10_5319676	1	2.271	0.8475	1.0000	0.8533	1.0000	0.1437
S10_2246820	10	2246820	S10_2251704	1	4.884	0.8458	0.9577	0.8777	0.9756	0.1520
S10_3827075	10	3827075	S10_3870253	1	43.178	0.8375	1.0000	0.8609	1.0000	0.1723
S10_441209	10	441209	S10_441737	1	0.528	0.8286	1.0000	0.8374	1.0000	0.2278
S10_1777091	10	1777091	S10_1785453	2	8.362	0.8272	0.9375	0.8505	0.9506	0.1729
S10_747828	10	747828	S10_748099	1	0.271	0.8234	0.9601	0.9063	1.0000	0.1560
S10_4620504	10	4620504	S10_4623463	3	2.959	0.8200	0.9592	0.8337	0.9672	0.1305

S10_7018253	10	7018253	S10_7018503	1	0.25	0.8166	0.9488	0.8567	0.9718	0.1144
S10_7018236	10	7018236	S10_7018253	1	0.017	0.8142	1.0000	0.8603	1.0000	0.1325
S10_4210686	10	4210686	S10_4226224	2	15.538	0.8116	0.9471	0.8474	0.9678	0.2298
S10_1486148	10	1486148	S10_1486749	1	0.601	0.8111	0.9311	0.8346	0.9445	0.0745
S10_11734628	10	11734628	S10_11734711	1	0.083	0.8027	0.9670	0.8130	0.9732	0.1587
S10_2684693	10	2684693	S10_2715483	4	30.79	0.8013	0.9615	0.8296	0.9784	0.1325
S10_3905231	10	3905231	S10_3939171	1	33.94	0.8011	0.9189	0.8389	0.9403	0.1854
S10_1709565	10	1709565	S10_1709640	1	0.075	0.7926	1.0000	0.8222	1.0000	0.1243
S10_3677010	10	3677010	S10_3775830	2	98.82	0.7867	0.9680	0.7978	0.9748	0.1683
S10_15768156	10	15768156	S10_15768170	1	0.014	0.7818	1.0000	0.7818	1.0000	0.0747
S10_747696	10	747696	S10_747828	2	0.132	0.7713	0.9606	0.8421	1.0000	0.1539
S10_1010796	10	1010796	S10_1021141	1	10.345	0.7437	0.9548	0.7847	0.9807	0.1220
S10_2206450	10	2206450	S10_2207870	1	1.42	0.7414	0.9146	0.7877	0.9427	0.1329
S10_3667586	10	3667586	S10_3677010	4	9.424	0.7356	0.9360	0.7695	0.9573	0.1653
S10_7667839	10	7667839	S10_7721606	3	53.767	0.7182	0.9055	0.7337	0.9153	0.1242
S10_3649694	10	3649694	S10_3667586	2	17.892	0.6815	0.9660	0.6932	0.9743	0.1527
S10_3802662	10	3802662	S10_3802858	2	0.196	0.6794	0.9079	0.6933	0.9172	0.1567
S10_4651262	10	4651262	S10_4712268	2	61.006	0.6234	0.8793	0.6838	0.9209	0.1210
S10_4623463	10	4623463	S10_4651214	1	27.751	0.6029	0.7764	0.6458	0.8036	0.1214
S10_3775848	10	3775848	S10_3802662	1	26.814	0.5481	0.7619	0.5613	0.7710	0.1374
S10_12080880	10	12080880	S10_12084150	1	3.27	0.5128	1.0000	0.5258	1.0000	0.1571
S10_3299693	10	3299693	S10_3356546	2	56.853	0.4848	0.9634	0.5037	0.9820	0.1678
S10_10270718	10	10270718	S10_10270766	1	0.048	0.4373	1.0000	0.4642	1.0000	0.1073
S10_405964	10	405964	S10_441209	1	35.245	0.3314	0.9540	0.3423	0.9696	0.1271
S10_13362917	10	13362917	S10_13362940	1	0.023	0.2149	1.0000	0.2070	0.9814	0.0784
S10_3380584	10	3380584	S10_3415789	2	35.205	0.2039	0.9275	0.2046	0.9291	0.0684
S11_1356826	11	1356826	S11_1380885	1	24.059	1.0000	1.0000	1.0000	1.0000	0.0615
S11_1380885	11	1380885	S11_1380920	1	0.035	1.0000	1.0000	1.0000	1.0000	0.0635
S11_1460770	11	1460770	S11_1462355	1	1.585	1.0000	1.0000	1.0000	1.0000	0.0670
S11_8262863	11	8262863	S11_8262880	1	0.017	1.0000	1.0000	1.0000	1.0000	0.0740

S11_8783466	11	8783466	S11_8783583	1	0.117	1.0000	1.0000	1.0000	1.0000	0.0866
S11_9253439	11	9253439	S11_9253458	1	0.019	1.0000	1.0000	1.0000	1.0000	0.0842
S11_11385820	11	11385820	S11_11385866	1	0.046	1.0000	1.0000	1.0000	1.0000	0.0794
S11_17612413	11	17612413	S11_17612420	1	0.007	1.0000	1.0000	1.0000	1.0000	0.0629
S11_21349831	11	21349831	S11_21353380	3	3.549	1.0000	1.0000	1.0000	1.0000	0.0951
S11_21353380	11	21353380	S11_21353459	1	0.079	1.0000	1.0000	1.0000	1.0000	0.0951
S11_22857466	11	22857466	S11_22861038	2	3.572	1.0000	1.0000	1.0000	1.0000	0.1457
S11_23105595	11	23105595	S11_23105689	1	0.094	1.0000	1.0000	1.0000	1.0000	0.1213
S11_1417567	11	1417567	S11_1460770	7	43.203	1.0000	1.0000	1.0000	1.0000	0.0781
S11_1581824	11	1581824	S11_1581851	1	0.027	1.0000	1.0000	1.0000	1.0000	0.0757
S11_2485963	11	2485963	S11_2557173	1	71.21	1.0000	1.0000	1.0000	1.0000	0.0499
S11_7950823	11	7950823	S11_7953675	1	2.852	1.0000	1.0000	1.0000	1.0000	0.1600
S11_8656444	11	8656444	S11_8703314	1	46.87	1.0000	1.0000	1.0000	1.0000	0.0757
S11_8703314	11	8703314	S11_8703350	1	0.036	1.0000	1.0000	1.0000	1.0000	0.0757
S11_23068032	11	23068032	S11_23068075	1	0.043	1.0000	1.0000	1.0000	1.0000	0.1427
S11_23105689	11	23105689	S11_23106176	1	0.487	1.0000	1.0000	1.0000	1.0000	0.1290
S11_4991234	11	4991234	S11_4991254	1	0.02	1.0000	1.0000	1.0000	1.0000	0.1114
S11_1724609	11	1724609	S11_1724615	1	0.006	1.0000	1.0000	1.0000	1.0000	0.2044
S11_1068801	11	1068801	S11_1068804	1	0.003	1.0000	1.0000	1.0000	1.0000	0.1848
S11_22157260	11	22157260	S11_22157266	1	0.006	1.0000	1.0000	1.0000	1.0000	0.2006
S11_24673133	11	24673133	S11_24673141	1	0.008	1.0000	1.0000	1.0000	1.0000	0.1537
S11_25515248	11	25515248	S11_25515292	1	0.044	1.0000	1.0000	1.0000	1.0000	0.1271
S11_27460078	11	27460078	S11_27460080	1	0.002	1.0000	1.0000	1.0000	1.0000	0.1523
S11_19616979	11	19616979	S11_19616997	1	0.018	1.0000	1.0000	1.0000	1.0000	0.1305
S11_24049264	11	24049264	S11_24049328	1	0.064	1.0000	1.0000	1.0000	1.0000	0.1316
S11_26539994	11	26539994	S11_26539995	1	0.001	1.0000	1.0000	1.0000	1.0000	0.1412
S11_22891696	11	22891696	S11_22891698	1	0.002	1.0000	1.0000	1.0000	1.0000	0.1219
S11_16091614	11	16091614	S11_16091634	1	0.02	1.0000	1.0000	1.0000	1.0000	0.1263
S11_19647690	11	19647690	S11_19647692	1	0.002	1.0000	1.0000	1.0000	1.0000	0.1153
S11_23750393	11	23750393	S11_23750399	1	0.006	1.0000	1.0000	1.0000	1.0000	0.1484

S11_7153732	11	7153732	S11_7153754	1	0.022	1.0000	1.0000	1.0000	1.0000	0.1054
S11_7153754	11	7153754	S11_7153763	1	0.009	1.0000	1.0000	1.0000	1.0000	0.1054
S11_15075142	11	15075142	S11_15075182	1	0.04	1.0000	1.0000	1.0000	1.0000	0.1242
S11_16190823	11	16190823	S11_16218609	1	27.786	1.0000	1.0000	1.0000	1.0000	0.1011
S11_26736573	11	26736573	S11_26736591	1	0.018	1.0000	1.0000	1.0000	1.0000	0.1284
S11_5716518	11	5716518	S11_5716519	1	0.001	1.0000	1.0000	1.0000	1.0000	0.1902
S11_16786270	11	16786270	S11_16786465	1	0.195	1.0000	1.0000	1.0000	1.0000	0.0993
S11_20391466	11	20391466	S11_20391483	1	0.017	1.0000	1.0000	1.0000	1.0000	0.0847
S11_23272945	11	23272945	S11_23272987	1	0.042	1.0000	1.0000	1.0000	1.0000	0.2434
S11_22386456	11	22386456	S11_22386457	1	0.001	1.0000	1.0000	1.0000	1.0000	0.0699
S11_27497494	11	27497494	S11_27497495	1	0.001	1.0000	1.0000	1.0000	1.0000	0.0699
S11_25844053	11	25844053	S11_25844079	1	0.026	1.0000	1.0000	1.0000	1.0000	0.1915
S11_18698530	11	18698530	S11_18698531	1	0.001	1.0000	1.0000	1.0000	1.0000	0.0545
S11_25844079	11	25844079	S11_25844140	1	0.061	1.0000	1.0000	1.0000	1.0000	0.1942
S11_22629114	11	22629114	S11_22629507	1	0.393	1.0000	1.0000	1.0000	1.0000	0.0580
S11_25842827	11	25842827	S11_25844053	1	1.226	1.0000	1.0000	1.0000	1.0000	0.2012
S11_3433835	11	3433835	S11_3454413	1	20.578	1.0000	1.0000	1.0000	1.0000	0.0853
S11_4177853	11	4177853	S11_4177870	1	0.017	1.0000	1.0000	1.0000	1.0000	0.1511
S11_1325831	11	1325831	S11_1325847	1	0.016	1.0000	1.0000	1.0000	1.0000	0.1494
S11_17711444	11	17711444	S11_17848873	1	137.429	1.0000	1.0000	1.0000	1.0000	0.1235
S11_17848873	11	17848873	S11_17848885	1	0.012	1.0000	1.0000	1.0000	1.0000	0.1235
S11_6540273	11	6540273	S11_6540319	1	0.046	1.0000	1.0000	1.0000	1.0000	0.1643
S11_17244284	11	17244284	S11_17256854	1	12.57	1.0000	1.0000	1.0000	1.0000	0.1279
S11_17650099	11	17650099	S11_17650109	1	0.01	1.0000	1.0000	1.0000	1.0000	0.1338
S11_14986475	11	14986475	S11_14987387	2	0.912	1.0000	1.0000	1.0000	1.0000	0.1084
S11_12470979	11	12470979	S11_12470996	1	0.017	1.0000	1.0000	1.0000	1.0000	0.1283
S11_12470996	11	12470996	S11_12471022	1	0.026	1.0000	1.0000	1.0000	1.0000	0.1283
S11_3970093	11	3970093	S11_3970102	1	0.009	1.0000	1.0000	1.0000	1.0000	0.2252
S11_10604340	11	10604340	S11_10604388	1	0.048	1.0000	1.0000	1.0000	1.0000	0.1005
S11_25059160	11	25059160	S11_25059188	1	0.028	1.0000	1.0000	1.0000	1.0000	0.1015

S11_23464432	11	23464432	S11_23464438	1	0.006	1.0000	1.0000	1.0000	1.0000	0.2477
S11_2621445	11	2621445	S11_2621446	1	0.001	1.0000	1.0000	1.0000	1.0000	0.0925
S11_11422523	11	11422523	S11_11576622	1	154.099	1.0000	1.0000	1.0000	1.0000	0.0925
S11_11586618	11	11586618	S11_11638658	1	52.04	1.0000	1.0000	1.0000	1.0000	0.0925
S11_11638658	11	11638658	S11_11638659	1	0.001	1.0000	1.0000	1.0000	1.0000	0.0925
S11_23836500	11	23836500	S11_23836517	1	0.017	1.0000	1.0000	1.0000	1.0000	0.1954
S11_26749931	11	26749931	S11_26749932	1	0.001	1.0000	1.0000	1.0000	1.0000	0.2499
S11_26749932	11	26749932	S11_26749933	1	0.001	1.0000	1.0000	1.0000	1.0000	0.2499
S11_2621446	11	2621446	S11_2624112	1	2.666	1.0000	1.0000	1.0000	1.0000	0.0850
S11_23998920	11	23998920	S11_23998921	1	0.001	1.0000	1.0000	1.0000	1.0000	0.1667
S11_3387303	11	3387303	S11_3387327	1	0.024	1.0000	1.0000	1.0000	1.0000	0.1606
S11_3433803	11	3433803	S11_3433805	1	0.002	1.0000	1.0000	1.0000	1.0000	0.0977
S11_3433805	11	3433805	S11_3433835	1	0.03	1.0000	1.0000	1.0000	1.0000	0.0977
S11_24199837	11	24199837	S11_24199890	2	0.053	1.0000	1.0000	1.0000	1.0000	0.0786
S11_24832788	11	24832788	S11_24832812	1	0.024	1.0000	1.0000	1.0000	1.0000	0.2148
S11_46545	11	46545	S11_46584	3	0.039	1.0000	1.0000	1.0000	1.0000	0.2066
S11_23678567	11	23678567	S11_23678568	1	0.001	1.0000	1.0000	1.0000	1.0000	0.0580
S11_26683930	11	26683930	S11_26683955	1	0.025	1.0000	1.0000	1.0000	1.0000	0.2491
S11_15890783	11	15890783	S11_15890787	1	0.004	1.0000	1.0000	1.0000	1.0000	0.1123
S11_15890787	11	15890787	S11_15890797	1	0.01	1.0000	1.0000	1.0000	1.0000	0.1123
S11_3396418	11	3396418	S11_3403527	2	7.109	1.0000	1.0000	1.0000	1.0000	0.0671
S11_23450053	11	23450053	S11_23450054	1	0.001	1.0000	1.0000	1.0000	1.0000	0.2272
S11_26650241	11	26650241	S11_26650242	1	0.001	1.0000	1.0000	1.0000	1.0000	0.1613
S11_26650242	11	26650242	S11_26650270	1	0.028	1.0000	1.0000	1.0000	1.0000	0.1613
S11_14061081	11	14061081	S11_14061082	1	0.001	1.0000	1.0000	1.0000	1.0000	0.0599
S11_23464167	11	23464167	S11_23464209	1	0.042	1.0000	1.0000	1.0000	1.0000	0.2468
S11_6692070	11	6692070	S11_6692072	1	0.002	1.0000	1.0000	1.0000	1.0000	0.0993
S11_6692072	11	6692072	S11_6692073	1	0.001	1.0000	1.0000	1.0000	1.0000	0.0993
S11_6692073	11	6692073	S11_6692074	1	0.001	1.0000	1.0000	1.0000	1.0000	0.0993
S11_16709151	11	16709151	S11_16709154	1	0.003	1.0000	1.0000	1.0000	1.0000	0.1136

S11_16709154	11	16709154	S11_16709155	1	0.001	1.0000	1.0000	1.0000	1.0000	0.1136
S11_281031	11	281031	S11_281035	1	0.004	1.0000	1.0000	1.0000	1.0000	0.2495
S11_281035	11	281035	S11_281036	1	0.001	1.0000	1.0000	1.0000	1.0000	0.2495
S11_23970245	11	23970245	S11_23970247	1	0.002	1.0000	1.0000	1.0000	1.0000	0.2358
S11_23970248	11	23970248	S11_23970250	1	0.002	1.0000	1.0000	1.0000	1.0000	0.2358
S11_3968947	11	3968947	S11_3968950	1	0.003	1.0000	1.0000	1.0000	1.0000	0.2210
S11_24175392	11	24175392	S11_24175398	1	0.006	1.0000	1.0000	1.0000	1.0000	0.0764
S11_1727143	11	1727143	S11_1731653	1	4.51	0.9741	1.0000	0.9868	1.0000	0.2035
S11_1229670	11	1229670	S11_1231800	1	2.13	0.9736	1.0000	0.9859	1.0000	0.1912
S11_1724797	11	1724797	S11_1725698	2	0.901	0.9732	1.0000	0.9865	1.0000	0.2079
S11_1724615	11	1724615	S11_1724797	1	0.182	0.9719	1.0000	0.9859	1.0000	0.2102
S11_7755317	11	7755317	S11_7755324	1	0.007	0.9703	1.0000	0.9846	1.0000	0.1697
S11_21718635	11	21718635	S11_21722613	1	3.978	0.9650	1.0000	0.9824	1.0000	0.1510
S11_10351965	11	10351965	S11_10352585	1	0.62	0.9634	1.0000	0.9816	1.0000	0.1428
S11_24672995	11	24672995	S11_24673133	1	0.138	0.9627	1.0000	0.9808	1.0000	0.1670
S11_2008668	11	2008668	S11_2012678	1	4.01	0.9617	1.0000	0.9803	1.0000	0.1508
S11_12358915	11	12358915	S11_12359206	2	0.291	0.9606	1.0000	0.9798	1.0000	0.1309
S11_24049328	11	24049328	S11_24049400	1	0.072	0.9603	1.0000	0.9795	1.0000	0.1344
S11_19616997	11	19616997	S11_19617110	1	0.113	0.9596	1.0000	0.9797	1.0000	0.1251
S11_27484864	11	27484864	S11_27496446	3	11.582	0.9589	1.0000	0.9772	1.0000	0.1354
S11_13208976	11	13208976	S11_13208997	1	0.021	0.9572	1.0000	0.9773	1.0000	0.1166
S11_5694008	11	5694008	S11_5716486	1	22.478	0.9571	1.0000	0.9772	1.0000	0.1186
S11_4396488	11	4396488	S11_4396676	1	0.188	0.9570	1.0000	0.9788	1.0000	0.1408
S11_4396676	11	4396676	S11_4396835	2	0.159	0.9557	1.0000	0.9777	1.0000	0.1380
S11_16252537	11	16252537	S11_16252990	4	0.453	0.9556	1.0000	0.9769	1.0000	0.1159
S11_16252990	11	16252990	S11_16255267	1	2.277	0.9556	1.0000	0.9769	1.0000	0.1159
S11_19617110	11	19617110	S11_19647690	1	30.58	0.9556	1.0000	0.9769	1.0000	0.1169
S11_23449871	11	23449871	S11_23450053	1	0.182	0.9547	0.9771	0.9763	0.9881	0.2245
S11_15890797	11	15890797	S11_15890809	1	0.012	0.9544	1.0000	0.9756	1.0000	0.1090
S11_16757467	11	16757467	S11_16758855	1	1.388	0.9543	1.0000	0.9756	1.0000	0.1099

S11_11998928	11	11998928	S11_12123995	1	125.067	0.9537	1.0000	0.9767	1.0000	0.1184
S11_16009913	11	16009913	S11_16017096	2	7.183	0.9527	1.0000	0.9756	1.0000	0.1061
S11_46584	11	46584	S11_46619	1	0.035	0.9504	0.9749	0.9743	0.9870	0.2027
S11_1725698	11	1725698	S11_1725894	1	0.196	0.9491	0.9742	0.9737	0.9868	0.1995
S11_28659	11	28659	S11_33212	1	4.553	0.9482	1.0000	0.9734	1.0000	0.1974
S11_23337294	11	23337294	S11_23337452	1	0.158	0.9468	1.0000	0.9454	0.9992	0.1835
S11_16752817	11	16752817	S11_16757467	1	4.65	0.9467	1.0000	0.9728	1.0000	0.0958
S11_25842807	11	25842807	S11_25842827	1	0.02	0.9463	1.0000	0.9448	0.9992	0.2000
S11_9253458	11	9253458	S11_9320544	1	67.086	0.9418	1.0000	0.9702	1.0000	0.0874
S11_24129399	11	24129399	S11_24129447	1	0.048	0.9391	1.0000	0.9655	1.0000	0.0806
S11_9214087	11	9214087	S11_9253439	1	39.352	0.9356	1.0000	0.9669	1.0000	0.0795
S11_4177786	11	4177786	S11_4177853	1	0.067	0.9354	1.0000	0.9332	0.9988	0.1490
S11_22022626	11	22022626	S11_22023380	1	0.754	0.9342	0.9774	0.9656	0.9937	0.2340
S11_131216	11	131216	S11_135599	3	4.383	0.9327	0.9769	0.9630	0.9926	0.2237
S11_26234271	11	26234271	S11_26244265	3	9.994	0.9319	1.0000	0.9637	1.0000	0.0764
S11_23079657	11	23079657	S11_23079753	1	0.096	0.9310	0.9649	0.9646	0.9821	0.1488
S11_7953675	11	7953675	S11_7955258	2	1.583	0.9305	1.0000	0.9655	1.0000	0.1467
S11_3391273	11	3391273	S11_3396418	1	5.145	0.9280	1.0000	0.9598	1.0000	0.0697
S11_10348094	11	10348094	S11_10351965	1	3.871	0.9279	1.0000	0.9643	1.0000	0.1352
S11_23213834	11	23213834	S11_23213858	1	0.024	0.9272	1.0000	0.9616	1.0000	0.2093
S11_21069739	11	21069739	S11_21069859	1	0.12	0.9270	1.0000	0.9624	1.0000	0.0799
S11_19134172	11	19134172	S11_19251738	1	117.566	0.9224	0.9604	0.9588	0.9792	0.1302
S11_17711420	11	17711420	S11_17711444	1	0.024	0.9221	1.0000	0.9189	0.9983	0.1218
S11_24049400	11	24049400	S11_24049466	1	0.066	0.9218	1.0000	0.9597	1.0000	0.1266
S11_23836475	11	23836475	S11_23836500	1	0.025	0.9214	1.0000	0.9326	1.0000	0.1922
S11_23836517	11	23836517	S11_23852313	1	15.796	0.9190	0.9720	0.9579	0.9923	0.1924
S11_6143661	11	6143661	S11_6143703	1	0.042	0.9174	1.0000	0.9574	1.0000	0.1182
S11_18698502	11	18698502	S11_18698530	1	0.028	0.9115	1.0000	0.9517	1.0000	0.0554
S11_16368412	11	16368412	S11_16401326	1	32.914	0.9102	1.0000	0.9315	1.0000	0.2385
S11_51990	11	51990	S11_53325	1	1.335	0.9065	1.0000	0.9523	1.0000	0.2098

S11_26650033	11	26650033	S11_26650241	1	0.208	0.9012	1.0000	0.9463	1.0000	0.1611
S11_26278941	11	26278941	S11_26279021	1	0.08	0.8989	0.9481	0.9460	0.9726	0.1054
S11_26104881	11	26104881	S11_26106963	1	2.082	0.8985	0.9783	0.9261	0.9932	0.2359
S11_21591279	11	21591279	S11_21591326	1	0.047	0.8961	0.9466	0.9459	0.9726	0.0939
S11_3454413	11	3454413	S11_3478148	1	23.735	0.8932	1.0000	0.9406	1.0000	0.0605
S11_25433091	11	25433091	S11_25433136	1	0.045	0.8894	1.0000	0.9046	1.0000	0.1280
S11_27270494	11	27270494	S11_27270631	1	0.137	0.8890	1.0000	0.8824	0.9963	0.0877
S11_7755324	11	7755324	S11_7877392	1	122.068	0.8813	1.0000	0.8776	0.9979	0.1543
S11_17179905	11	17179905	S11_17244284	1	64.379	0.8791	1.0000	0.9398	1.0000	0.1253
S11_19121917	11	19121917	S11_19134172	2	12.255	0.8787	0.9572	0.9372	0.9886	0.1314
S11_8703350	11	8703350	S11_8783466	1	80.116	0.8787	1.0000	0.8787	1.0000	0.0762
S11_23450054	11	23450054	S11_23451756	1	1.702	0.8767	0.9732	0.9361	1.0000	0.2158
S11_4997766	11	4997766	S11_4997829	1	0.063	0.8762	1.0000	0.8927	1.0000	0.1152
S11_11923233	11	11923233	S11_11963281	1	40.048	0.8748	0.9558	0.9343	0.9877	0.1298
S11_33212	11	33212	S11_46545	1	13.333	0.8732	0.9468	0.9326	0.9784	0.1979
S11_11910298	11	11910298	S11_11923233	1	12.935	0.8718	1.0000	0.8912	1.0000	0.1146
S11_24199890	11	24199890	S11_24199976	1	0.086	0.8673	0.9313	0.9276	0.9631	0.0788
S11_14987387	11	14987387	S11_15019160	2	31.773	0.8644	0.9518	0.8829	0.9620	0.1074
S11_1380990	11	1380990	S11_1382918	3	1.928	0.8628	0.9699	0.8998	0.9905	0.2083
S11_26155338	11	26155338	S11_26155455	1	0.117	0.8622	1.0000	0.8825	1.0000	0.1117
S11_23464209	11	23464209	S11_23464432	2	0.223	0.8607	0.9743	0.9024	0.9976	0.2339
S11_15890809	11	15890809	S11_16009913	1	119.104	0.8603	0.9503	0.9253	0.9855	0.1033
S11_23068075	11	23068075	S11_23075097	1	7.022	0.8578	1.0000	0.8904	1.0000	0.1413
S11_25844140	11	25844140	S11_25844569	1	0.429	0.8572	0.9612	0.9252	0.9985	0.1478
S11_46619	11	46619	S11_51990	2	5.371	0.8559	0.9682	0.8951	0.9901	0.1949
S11_4991254	11	4991254	S11_4991344	1	0.09	0.8540	0.9479	0.9230	0.9854	0.1051
S11_22019819	11	22019819	S11_22022626	1	2.807	0.8532	0.9546	0.9000	0.9804	0.2252
S11_21591326	11	21591326	S11_21660462	3	69.136	0.8479	1.0000	0.8736	1.0000	0.0923
S11_18695690	11	18695690	S11_18698502	1	2.812	0.8475	1.0000	0.9109	1.0000	0.0607
S11_20726709	11	20726709	S11_20726764	1	0.055	0.8444	1.0000	0.8412	0.9981	0.1371

S11_27111786	11	27111786	S11_27111818	1	0.032	0.8417	1.0000	0.9217	1.0000	0.0907
S11_25480121	11	25480121	S11_25480310	1	0.189	0.8411	1.0000	0.9154	1.0000	0.1683
S11_3227590	11	3227590	S11_3247921	1	20.331	0.8374	1.0000	0.8529	1.0000	0.1053
S11_9881674	11	9881674	S11_10006867	1	125.193	0.8368	0.9412	0.9129	0.9831	0.0872
S11_18698531	11	18698531	S11_18698622	1	0.091	0.8366	1.0000	0.9078	1.0000	0.0567
S11_23075097	11	23075097	S11_23079657	1	4.56	0.8324	0.9624	0.8783	0.9886	0.1414
S11_16786465	11	16786465	S11_16808056	2	21.591	0.8317	1.0000	0.8661	1.0000	0.1051
S11_18924689	11	18924689	S11_18924755	1	0.066	0.8234	1.0000	0.8938	1.0000	0.0546
S11_12359206	11	12359206	S11_12470979	1	111.773	0.8230	1.0000	0.8615	1.0000	0.1132
S11_4483697	11	4483697	S11_4572819	2	89.122	0.8185	1.0000	0.8309	1.0000	0.1171
S11_26153927	11	26153927	S11_26155338	1	1.411	0.8162	0.9487	0.8564	0.9717	0.1155
S11_27451178	11	27451178	S11_27459944	1	8.766	0.8156	1.0000	0.8468	1.0000	0.0867
S11_7581953	11	7581953	S11_7590511	3	8.558	0.8007	1.0000	0.8176	1.0000	0.1140
S11_21294914	11	21294914	S11_21349831	1	54.917	0.7994	1.0000	0.7994	1.0000	0.0830
S11_24049466	11	24049466	S11_24049496	1	0.03	0.7974	1.0000	0.8104	1.0000	0.1023
S11_22779181	11	22779181	S11_22800505	1	21.324	0.7955	1.0000	0.7955	1.0000	0.0960
S11_16808056	11	16808056	S11_16873673	2	65.617	0.7937	0.9106	0.8862	0.9622	0.1110
S11_3247921	11	3247921	S11_3327154	3	79.233	0.7927	1.0000	0.8842	1.0000	0.1217
S11_479034	11	479034	S11_500910	1	21.876	0.7907	0.9406	0.8372	0.9678	0.0885
S11_21056055	11	21056055	S11_21069706	3	13.651	0.7907	0.9516	0.8860	1.0000	0.1073
S11_23750399	11	23750399	S11_23750404	1	0.005	0.7906	1.0000	0.7868	0.9976	0.1413
S11_20796769	11	20796769	S11_20925900	2	129.131	0.7872	0.9506	0.8025	0.9598	0.1118
S11_17612420	11	17612420	S11_17612423	1	0.003	0.7852	1.0000	0.8817	1.0000	0.0668
S11_17612423	11	17612423	S11_17612424	1	0.001	0.7852	1.0000	0.8817	1.0000	0.0668
S11_23852313	11	23852313	S11_23862058	1	9.745	0.7832	0.9197	0.8321	0.9480	0.1909
S11_4858156	11	4858156	S11_4932526	5	74.37	0.7786	0.9483	0.7988	0.9606	0.1134
S11_500910	11	500910	S11_541028	1	40.118	0.7728	0.9347	0.8226	0.9644	0.0769
S11_17111963	11	17111963	S11_17179905	13	67.942	0.7699	0.8774	0.8373	0.9150	0.1204
S11_26540048	11	26540048	S11_26559272	1	19.224	0.7657	0.9124	0.7883	0.9258	0.1641
S11_15075182	11	15075182	S11_15091873	1	16.691	0.7594	0.9518	0.8293	0.9946	0.1213

S11_7153763	11	7153763	S11_7294402	1	140.639	0.7589	0.9429	0.7782	0.9548	0.0908
S11_25480440	11	25480440	S11_25492624	2	12.184	0.7566	1.0000	0.7460	0.9929	0.1007
S11_16091634	11	16091634	S11_16103058	1	11.424	0.7558	1.0000	0.7958	1.0000	0.1292
S11_20391459	11	20391459	S11_20391466	1	0.007	0.7493	1.0000	0.7629	1.0000	0.0829
S11_26279021	11	26279021	S11_26326127	1	47.106	0.7449	0.9388	0.8085	0.9780	0.1002
S11_25426440	11	25426440	S11_25433091	1	6.651	0.7385	0.8768	0.7811	0.9018	0.1169
S11_15019160	11	15019160	S11_15075142	1	55.982	0.7369	0.8584	0.7729	0.8792	0.1136
S11_7294402	11	7294402	S11_7294548	1	0.146	0.7354	1.0000	0.7562	1.0000	0.0881
S11_16873673	11	16873673	S11_16890437	1	16.764	0.7119	0.8628	0.8007	0.9150	0.1177
S11_4396835	11	4396835	S11_4403094	1	6.259	0.7118	0.9476	0.7216	0.9541	0.1099
S11_21069706	11	21069706	S11_21069739	1	0.033	0.7105	0.9395	0.8367	1.0000	0.0957
S11_26923049	11	26923049	S11_26950648	4	27.599	0.7003	1.0000	0.7003	1.0000	0.0788
S11_2012817	11	2012817	S11_2071043	3	58.226	0.6885	1.0000	0.7049	1.0000	0.0880
S11_25492624	11	25492624	S11_25515248	9	22.624	0.6885	0.9487	0.7187	0.9693	0.0986
S11_18924670	11	18924670	S11_18924689	1	0.019	0.6622	0.8664	0.8057	0.9557	0.0511
S11_26736591	11	26736591	S11_26749863	1	13.272	0.6601	0.8943	0.7265	0.9382	0.1167
S11_10482420	11	10482420	S11_10604340	3	121.92	0.6589	0.8811	0.6683	0.8873	0.0770
S11_606034	11	606034	S11_672728	1	66.694	0.6545	1.0000	0.6630	1.0000	0.0703
S11_24369991	11	24369991	S11_24374053	2	4.062	0.6542	0.9334	0.6968	0.9633	0.0748
S11_17612424	11	17612424	S11_17650099	3	37.675	0.6404	1.0000	0.6830	1.0000	0.0673
S11_10204016	11	10204016	S11_10348094	2	144.078	0.6398	0.8675	0.6897	0.9007	0.1053
S11_16890437	11	16890437	S11_16890442	1	0.005	0.6272	1.0000	0.6413	1.0000	0.0901
S11_4403094	11	4403094	S11_4403190	1	0.096	0.6265	1.0000	0.6189	0.9939	0.1083
S11_27111818	11	27111818	S11_27153355	3	41.537	0.6210	0.9329	0.6556	0.9586	0.0754
S11_16252288	11	16252288	S11_16252537	1	0.249	0.6184	0.9391	0.6667	0.9751	0.0834
S11_1726969	11	1726969	S11_1727143	1	0.174	0.6173	1.0000	0.6155	0.9985	0.1421
S11_1725894	11	1725894	S11_1726969	1	1.075	0.6034	1.0000	0.6180	1.0000	0.1400
S11_27208416	11	27208416	S11_27208433	1	0.017	0.5958	1.0000	0.5672	0.9757	0.1260
S11_27496755	11	27496755	S11_27497494	3	0.739	0.5845	0.9141	0.6502	0.9641	0.0652
S11_27208433	11	27208433	S11_27209349	1	0.916	0.5625	0.9593	0.5488	0.9476	0.1241

S11_26155455	11	26155455	S11_26234271	3	78.816	0.5296	0.7793	0.5585	0.8003	0.0586
S11_27496446	11	27496446	S11_27496755	1	0.309	0.5222	1.0000	0.5341	1.0000	0.0855
S11_27183484	11	27183484	S11_27208416	1	24.932	0.5135	0.9669	0.5396	0.9911	0.1573
S11_24129447	11	24129447	S11_24173641	1	44.194	0.4924	0.7253	0.5320	0.7540	0.0567
S11_21660462	11	21660462	S11_21718635	1	58.173	0.4874	0.8878	0.4967	0.8962	0.0918
S11_11255498	11	11255498	S11_11385820	1	130.322	0.4859	0.8156	0.4859	0.8156	0.0533
S11_21722613	11	21722613	S11_21742524	1	19.911	0.4782	0.8484	0.4959	0.8640	0.0882
S11_16103058	11	16103058	S11_16190823	1	87.765	0.4666	0.8914	0.4839	0.9077	0.0913
S11_27497495	11	27497495	S11_27568651	3	71.156	0.4324	0.9089	0.4892	0.9668	0.0717
S11_4261063	11	4261063	S11_4315854	6	54.791	0.4122	0.6421	0.4319	0.6572	0.0405
S11_1231800	11	1231800	S11_1233477	1	1.677	0.3111	1.0000	0.3244	1.0000	0.0752
S11_27209349	11	27209349	S11_27227367	2	18.018	0.2582	1.0000	0.2766	1.0000	0.0638
S11_16401326	11	16401326	S11_16499762	1	98.436	0.1448	1.0000	0.1612	1.0000	0.0577
S11_1337626	11	1337626	S11_1337761	1	0.135	0.0045	1.0000	0.0048	1.0000	-0.0041
S11_1337761	11	1337761	S11_1356826	3	19.065	0.0037	1.0000	0.0039	1.0000	-0.0034
S11_24175317	11	24175317	S11_24175392	1	0.075	0.0008	1.0000	0.0027	1.0000	-0.0013
S11_24173641	11	24173641	S11_24175317	1	1.676	0.0007	1.0000	0.0026	1.0000	-0.0013
S11_23332317	11	23332317	S11_23332331	1	0.014	0.0000	0.0000	0.0000	0.0000	0.0000
S12_2213598	12	2213598	S12_2213765	1	0.167	1.0000	1.0000	1.0000	1.0000	0.1134
S12_2213765	12	2213765	S12_2308592	1	94.827	1.0000	1.0000	1.0000	1.0000	0.1286
S12_14807434	12	14807434	S12_14807435	1	0.001	1.0000	1.0000	1.0000	1.0000	0.0710
S12_19649015	12	19649015	S12_19649233	1	0.218	1.0000	1.0000	1.0000	1.0000	0.0818
S12_21592658	12	21592658	S12_21592667	1	0.009	1.0000	1.0000	1.0000	1.0000	0.0542
S12_22910242	12	22910242	S12_22910245	1	0.003	1.0000	1.0000	1.0000	1.0000	0.2228
S12_22910245	12	22910245	S12_22910290	1	0.045	1.0000	1.0000	1.0000	1.0000	0.2228
S12_23665674	12	23665674	S12_23665678	1	0.004	1.0000	1.0000	1.0000	1.0000	0.1049
S12_23665678	12	23665678	S12_23665685	1	0.007	1.0000	1.0000	1.0000	1.0000	0.1049
S12_9786958	12	9786958	S12_9911841	1	124.883	1.0000	1.0000	1.0000	1.0000	0.1376
S12_19240071	12	19240071	S12_19240136	2	0.065	1.0000	1.0000	1.0000	1.0000	0.0494
S12_18714094	12	18714094	S12_18714107	1	0.013	1.0000	1.0000	1.0000	1.0000	0.1416

S12_19240136	12	19240136	S12_19250140	1	10.004	1.0000	1.0000	1.0000	1.0000	0.0671
S12_21467627	12	21467627	S12_21467637	1	0.01	1.0000	1.0000	1.0000	1.0000	0.2458
S12_24220759	12	24220759	S12_24220760	1	0.001	1.0000	1.0000	1.0000	1.0000	0.2261
S12_472751	12	472751	S12_472781	1	0.03	1.0000	1.0000	1.0000	1.0000	0.1271
S12_10014043	12	10014043	S12_10014107	1	0.064	1.0000	1.0000	1.0000	1.0000	0.1271
S12_21310420	12	21310420	S12_21310426	1	0.006	1.0000	1.0000	1.0000	1.0000	0.2078
S12_1084320	12	1084320	S12_1084342	1	0.022	1.0000	1.0000	1.0000	1.0000	0.1198
S12_21606754	12	21606754	S12_21606755	1	0.001	1.0000	1.0000	1.0000	1.0000	0.2211
S12_22355333	12	22355333	S12_22355350	1	0.017	1.0000	1.0000	1.0000	1.0000	0.2222
S12_1137513	12	1137513	S12_1137514	1	0.001	1.0000	1.0000	1.0000	1.0000	0.0637
S12_14873347	12	14873347	S12_14903006	2	29.659	1.0000	1.0000	1.0000	1.0000	0.0699
S12_14676203	12	14676203	S12_14676204	1	0.001	1.0000	1.0000	1.0000	1.0000	0.2087
S12_749232	12	749232	S12_749337	1	0.105	1.0000	1.0000	1.0000	1.0000	0.1809
S12_4225543	12	4225543	S12_4225559	1	0.016	1.0000	1.0000	1.0000	1.0000	0.0586
S12_23001518	12	23001518	S12_23001542	1	0.024	1.0000	1.0000	1.0000	1.0000	0.1649
S12_21737332	12	21737332	S12_21737388	1	0.056	1.0000	1.0000	1.0000	1.0000	0.1511
S12_17525402	12	17525402	S12_17525446	1	0.044	1.0000	1.0000	1.0000	1.0000	0.1587
S12_13770094	12	13770094	S12_13770110	1	0.016	1.0000	1.0000	1.0000	1.0000	0.0530
S12_13770110	12	13770110	S12_13770121	1	0.011	1.0000	1.0000	1.0000	1.0000	0.0530
S12_13770121	12	13770121	S12_13770135	1	0.014	1.0000	1.0000	1.0000	1.0000	0.0530
S12_21998026	12	21998026	S12_21998032	1	0.006	1.0000	1.0000	1.0000	1.0000	0.2129
S12_20135373	12	20135373	S12_20135387	1	0.014	1.0000	1.0000	1.0000	1.0000	0.1005
S12_20135387	12	20135387	S12_20135399	1	0.012	1.0000	1.0000	1.0000	1.0000	0.1005
S12_21902217	12	21902217	S12_21902235	1	0.018	1.0000	1.0000	1.0000	1.0000	0.2191
S12_20648279	12	20648279	S12_20648300	1	0.021	1.0000	1.0000	1.0000	1.0000	0.2024
S12_27991	12	27991	S12_27992	1	0.001	1.0000	1.0000	1.0000	1.0000	0.1249
S12_8604900	12	8604900	S12_8604901	1	0.001	1.0000	1.0000	1.0000	1.0000	0.2460
S12_8604901	12	8604901	S12_8604902	1	0.001	1.0000	1.0000	1.0000	1.0000	0.2460
S12_23000863	12	23000863	S12_23000889	1	0.026	1.0000	1.0000	1.0000	1.0000	0.1606
S12_24786330	12	24786330	S12_24786531	1	0.201	1.0000	1.0000	1.0000	1.0000	0.1932

S12_22574877	12	22574877	S12_22574901	1	0.024	1.0000	1.0000	1.0000	1.0000	0.1773
S12_9312877	12	9312877	S12_9312912	1	0.035	1.0000	1.0000	1.0000	1.0000	0.2489
S12_23723877	12	23723877	S12_23723880	1	0.003	1.0000	1.0000	1.0000	1.0000	0.2222
S12_21357709	12	21357709	S12_21357729	1	0.02	1.0000	1.0000	1.0000	1.0000	0.2361
S12_21357729	12	21357729	S12_21357749	1	0.02	1.0000	1.0000	1.0000	1.0000	0.2361
S12_23828003	12	23828003	S12_23828004	1	0.001	1.0000	1.0000	1.0000	1.0000	0.2467
S12_1053374	12	1053374	S12_1055849	1	2.475	1.0000	1.0000	1.0000	1.0000	0.1649
S12_23001572	12	23001572	S12_23001609	1	0.037	1.0000	1.0000	1.0000	1.0000	0.1587
S12_21739295	12	21739295	S12_21739309	1	0.014	1.0000	1.0000	1.0000	1.0000	0.1665
S12_23397338	12	23397338	S12_23397344	1	0.006	1.0000	1.0000	1.0000	1.0000	0.2100
S12_23405131	12	23405131	S12_23405176	1	0.045	1.0000	1.0000	1.0000	1.0000	0.2242
S12_24285962	12	24285962	S12_24286071	1	0.109	1.0000	1.0000	1.0000	1.0000	0.2245
S12_23995911	12	23995911	S12_23995935	1	0.024	1.0000	1.0000	1.0000	1.0000	0.2492
S12_5184139	12	5184139	S12_5184175	1	0.036	1.0000	1.0000	1.0000	1.0000	0.0625
S12_5184175	12	5184175	S12_5184191	1	0.016	1.0000	1.0000	1.0000	1.0000	0.0625
S12_24321830	12	24321830	S12_24321835	1	0.005	1.0000	1.0000	1.0000	1.0000	0.0925
S12_19345634	12	19345634	S12_19345644	1	0.01	1.0000	1.0000	1.0000	1.0000	0.2327
S12_22072570	12	22072570	S12_22072572	1	0.002	1.0000	1.0000	1.0000	1.0000	0.0676
S12_24018837	12	24018837	S12_24018889	1	0.052	1.0000	1.0000	1.0000	1.0000	0.2108
S12_1214330	12	1214330	S12_1214331	1	0.001	1.0000	1.0000	1.0000	1.0000	0.1053
S12_1214331	12	1214331	S12_1214333	1	0.002	1.0000	1.0000	1.0000	1.0000	0.1053
S12_27989	12	27989	S12_27990	1	0.001	1.0000	1.0000	1.0000	1.0000	0.1363
S12_261976	12	261976	S12_261977	1	0.001	1.0000	1.0000	1.0000	1.0000	0.1338
S12_3707100	12	3707100	S12_3707106	1	0.006	0.9784	1.0000	0.9882	1.0000	0.2351
S12_1214410	12	1214410	S12_1215392	1	0.982	0.9773	1.0000	0.9883	1.0000	0.2302
S12_23000889	12	23000889	S12_23001518	1	0.629	0.9686	1.0000	0.9840	1.0000	0.1621
S12_218728	12	218728	S12_248500	2	29.772	0.9627	1.0000	0.9811	1.0000	0.1531
S12_9911841	12	9911841	S12_9915377	2	3.536	0.9601	1.0000	0.9803	1.0000	0.1381
S12_18728808	12	18728808	S12_18728841	1	0.033	0.9595	1.0000	0.9796	1.0000	0.1273
S12_18714107	12	18714107	S12_18728808	1	14.701	0.9583	1.0000	0.9789	1.0000	0.1434

S12_21403595	12	21403595	S12_21403643	1	0.048	0.9579	0.9787	0.9778	0.9888	0.2419
S12_17738703	12	17738703	S12_17739231	1	0.528	0.9575	0.9785	0.9777	0.9888	0.2426
S12_21467637	12	21467637	S12_21467646	1	0.009	0.9571	1.0000	0.9566	0.9998	0.2397
S12_23723880	12	23723880	S12_23723985	1	0.105	0.9536	1.0000	0.9758	1.0000	0.2189
S12_23963194	12	23963194	S12_23963195	1	0.001	0.9528	1.0000	0.9579	1.0000	0.2380
S12_20135324	12	20135324	S12_20135373	1	0.049	0.9490	1.0000	0.9733	1.0000	0.0972
S12_19466940	12	19466940	S12_19471218	1	4.278	0.9487	1.0000	0.9731	1.0000	0.1018
S12_22582039	12	22582039	S12_22582043	1	0.004	0.9401	1.0000	0.9382	0.9990	0.1802
S12_22582043	12	22582043	S12_22582052	1	0.009	0.9401	1.0000	0.9382	0.9990	0.1802
S12_10130882	12	10130882	S12_10131421	1	0.539	0.9391	0.9691	0.9688	0.9843	0.1641
S12_21403499	12	21403499	S12_21403595	1	0.096	0.9362	0.9781	0.9659	0.9935	0.2392
S12_21310417	12	21310417	S12_21310420	1	0.003	0.9353	1.0000	0.9342	0.9994	0.1980
S12_21737388	12	21737388	S12_21739295	1	1.907	0.9342	1.0000	0.9653	1.0000	0.1604
S12_22999506	12	22999506	S12_22999629	1	0.123	0.9280	1.0000	0.9625	1.0000	0.1641
S12_3281751	12	3281751	S12_3284633	1	2.882	0.9279	1.0000	0.9639	1.0000	0.0705
S12_24282982	12	24282982	S12_24283083	1	0.101	0.9260	0.9745	0.9611	0.9928	0.2211
S12_10014107	12	10014107	S12_10014138	1	0.031	0.9241	1.0000	0.9227	0.9992	0.1255
S12_24400771	12	24400771	S12_24407742	4	6.971	0.9230	0.9734	0.9595	0.9924	0.2148
S12_24283083	12	24283083	S12_24283198	1	0.115	0.9207	0.9726	0.9307	0.9778	0.2101
S12_18713839	12	18713839	S12_18714071	2	0.232	0.9201	0.9592	0.9592	0.9794	0.1464
S12_17518555	12	17518555	S12_17525402	1	6.847	0.9201	0.9592	0.9579	0.9787	0.1463
S12_471725	12	471725	S12_472751	2	1.026	0.9197	1.0000	0.9181	0.9991	0.1193
S12_21808935	12	21808935	S12_21809056	1	0.121	0.9196	0.9722	0.9588	0.9927	0.1963
S12_18714071	12	18714071	S12_18714094	1	0.023	0.9196	1.0000	0.9196	1.0000	0.1395
S12_17738701	12	17738701	S12_17738703	1	0.002	0.9192	1.0000	0.9369	1.0000	0.2368
S12_24407742	12	24407742	S12_24411649	2	3.907	0.9174	0.9714	0.9570	0.9921	0.2255
S12_21511082	12	21511082	S12_21511310	1	0.228	0.9174	0.9783	0.9572	0.9993	0.2428
S12_20637068	12	20637068	S12_20637255	2	0.187	0.9153	0.9778	0.9553	0.9989	0.2317
S12_284684	12	284684	S12_369786	2	85.102	0.9153	1.0000	0.9153	1.0000	0.1302
S12_9312774	12	9312774	S12_9312877	1	0.103	0.9142	0.9775	0.9544	0.9987	0.2437

S12_3882021	12	3882021	S12_3885644	1	3.623	0.9141	0.9561	0.9546	0.9770	0.1483
S12_8936932	12	8936932	S12_8937088	1	0.156	0.9130	0.9555	0.9540	0.9767	0.1144
S12_17810992	12	17810992	S12_17811037	1	0.045	0.9121	1.0000	0.9532	1.0000	0.1641
S12_24220712	12	24220712	S12_24220759	1	0.047	0.9079	1.0000	0.9474	1.0000	0.2120
S12_24286071	12	24286071	S12_24289561	6	3.49	0.9065	0.9753	0.9516	0.9993	0.2250
S12_19688946	12	19688946	S12_19698806	1	9.86	0.9059	1.0000	0.9059	1.0000	0.1003
S12_8854756	12	8854756	S12_8936932	2	82.176	0.9055	0.9516	0.9498	0.9746	0.1170
S12_22762229	12	22762229	S12_22766143	1	3.914	0.9040	0.9508	0.9497	0.9745	0.2201
S12_3860446	12	3860446	S12_3882021	2	21.575	0.8996	1.0000	0.9134	1.0000	0.1396
S12_22513871	12	22513871	S12_22514019	1	0.148	0.8989	0.9784	0.9457	1.0000	0.2419
S12_27992	12	27992	S12_68313	1	40.321	0.8970	0.9471	0.9471	0.9732	0.1179
S12_23397344	12	23397344	S12_23405131	1	7.787	0.8950	0.9721	0.9154	0.9831	0.2151
S12_22052186	12	22052186	S12_22052358	1	0.172	0.8906	0.9708	0.9398	0.9972	0.1882
S12_24238335	12	24238335	S12_24238467	2	0.132	0.8905	1.0000	0.8874	0.9982	0.1112
S12_19345566	12	19345566	S12_19345634	1	0.068	0.8897	0.9540	0.9391	0.9801	0.2248
S12_21514104	12	21514104	S12_21514130	1	0.026	0.8861	0.9525	0.9406	0.9813	0.2320
S12_23223123	12	23223123	S12_23223127	1	0.004	0.8843	1.0000	0.8808	0.9980	0.0811
S12_7902983	12	7902983	S12_7933453	5	30.47	0.8817	0.9743	0.9338	1.0000	0.2253
S12_7274060	12	7274060	S12_7296867	1	22.807	0.8772	0.9670	0.9318	0.9966	0.1738
S12_24233839	12	24233839	S12_24238335	1	4.496	0.8761	1.0000	0.9364	1.0000	0.1567
S12_23001542	12	23001542	S12_23001572	1	0.03	0.8760	0.9666	0.9353	0.9988	0.1577
S12_23067689	12	23067689	S12_23082938	1	15.249	0.8730	0.9343	0.9321	0.9655	0.0897
S12_22999629	12	22999629	S12_23000863	1	1.234	0.8717	0.9336	0.9317	0.9652	0.1582
S12_21457268	12	21457268	S12_21457362	1	0.094	0.8687	0.9713	0.9287	1.0000	0.2137
S12_13740569	12	13740569	S12_13740610	1	0.041	0.8681	0.9532	0.8858	0.9629	0.2317
S12_7274009	12	7274009	S12_7274060	1	0.051	0.8680	0.9317	0.9271	0.9629	0.1700
S12_1138746	12	1138746	S12_1145296	2	6.55	0.8651	1.0000	0.8552	0.9943	0.0671
S12_19698806	12	19698806	S12_19700012	1	1.206	0.8595	0.9500	0.8814	0.9620	0.1046
S12_401616	12	401616	S12_401701	2	0.085	0.8550	0.9483	0.9001	0.9730	0.2187
S12_27990	12	27990	S12_27991	1	0.001	0.8507	1.0000	0.9274	1.0000	0.1384

S12_23747591	12	23747591	S12_23762640	1	15.049	0.8494	0.9461	0.8721	0.9587	0.2069
S12_22098752	12	22098752	S12_22102287	1	3.535	0.8477	0.9662	0.8864	0.9880	0.1978
S12_14943935	12	14943935	S12_14955004	1	11.069	0.8475	1.0000	0.9303	1.0000	0.0613
S12_22581779	12	22581779	S12_22581832	1	0.053	0.8456	1.0000	0.8979	1.0000	0.2352
S12_20648300	12	20648300	S12_20648354	1	0.054	0.8433	0.9706	0.8900	0.9972	0.1829
S12_22840504	12	22840504	S12_22854722	1	14.218	0.8402	0.9359	0.9102	0.9741	0.2359
S12_18457851	12	18457851	S12_18461450	1	3.599	0.8372	0.9414	0.9125	0.9828	0.2046
S12_24760513	12	24760513	S12_24763895	1	3.382	0.8347	0.9136	0.8835	0.9399	0.1737
S12_23994280	12	23994280	S12_23995911	1	1.631	0.8331	0.9538	0.8854	0.9833	0.2349
S12_14676204	12	14676204	S12_14676275	1	0.071	0.8319	0.9726	0.8861	1.0000	0.2013
S12_17902031	12	17902031	S12_18048906	3	146.875	0.8316	0.9622	0.8752	0.9871	0.1675
S12_22581778	12	22581778	S12_22581779	1	0.001	0.8048	0.9552	0.8333	0.9720	0.2271
S12_16695188	12	16695188	S12_16696789	1	1.601	0.8033	1.0000	0.8412	1.0000	0.1019
S12_24285959	12	24285959	S12_24285962	1	0.003	0.8006	1.0000	0.8058	1.0000	0.2076
S12_1137514	12	1137514	S12_1138746	1	1.232	0.7987	1.0000	0.8206	1.0000	0.0635
S12_19341211	12	19341211	S12_19341241	1	0.03	0.7915	1.0000	0.7846	0.9956	0.2143
S12_22366	12	22366	S12_27989	2	5.623	0.7899	0.9514	0.8964	1.0000	0.1319
S12_19701148	12	19701148	S12_19701204	1	0.056	0.7877	0.8875	0.7877	0.8875	0.0877
S12_16696789	12	16696789	S12_16696897	1	0.108	0.7770	0.9027	0.8780	0.9596	0.1133
S12_12375085	12	12375085	S12_12375225	1	0.14	0.7756	1.0000	0.8096	1.0000	0.1164
S12_19341241	12	19341241	S12_19345566	1	4.325	0.7576	1.0000	0.7682	1.0000	0.2109
S12_16684521	12	16684521	S12_16691210	2	6.689	0.7542	0.8684	0.8011	0.8950	0.0841
S12_22089317	12	22089317	S12_22089329	1	0.012	0.7460	1.0000	0.7649	1.0000	0.1839
S12_16691210	12	16691210	S12_16695188	1	3.978	0.7440	0.9249	0.7968	0.9572	0.0829
S12_24283198	12	24283198	S12_24285959	1	2.761	0.7341	0.9454	0.7631	0.9638	0.1992
S12_16696916	12	16696916	S12_16697103	2	0.187	0.7131	1.0000	0.8430	1.0000	0.0693
S12_23175105	12	23175105	S12_23175229	1	0.124	0.7025	0.9416	0.7279	0.9585	0.1983
S12_17811037	12	17811037	S12_17819931	1	8.894	0.6972	0.9082	0.7209	0.9236	0.1245
S12_16696897	12	16696897	S12_16696916	1	0.019	0.6899	0.8941	0.7758	0.9481	0.1007
S12_24786531	12	24786531	S12_24788462	1	1.931	0.6865	0.9329	0.7270	0.9600	0.1770

S12_12587679	12	12587679	S12_12729237	1	141.558	0.6803	0.9020	0.7458	0.9445	0.0535
S12_18231734	12	18231734	S12_18318554	1	86.82	0.6767	0.9393	0.6973	0.9534	0.0872
S12_19701204	12	19701204	S12_19820777	1	119.573	0.6654	0.8375	0.6838	0.8490	0.0861
S12_19700012	12	19700012	S12_19700995	2	0.983	0.6626	0.9430	0.6967	0.9669	0.1034
S12_19700995	12	19700995	S12_19701148	1	0.153	0.6618	1.0000	0.6791	1.0000	0.0867
S12_23064931	12	23064931	S12_23067689	1	2.758	0.6324	0.8520	0.7276	0.9139	0.0718
S12_15860794	12	15860794	S12_15860952	2	0.158	0.5971	1.0000	0.6229	1.0000	0.0430
S12_20135399	12	20135399	S12_20247645	7	112.246	0.4931	0.7456	0.5581	0.7931	0.0789
S12_1270858	12	1270858	S12_1270875	1	0.017	0.4788	1.0000	0.4593	0.9794	0.1580
S12_22583853	12	22583853	S12_22583880	1	0.027	0.4648	1.0000	0.4779	1.0000	0.1576
S12_22582052	12	22582052	S12_22583853	2	1.801	0.4026	1.0000	0.4260	1.0000	0.1441
S12_19671939	12	19671939	S12_19688946	1	17.007	0.3685	0.6826	0.3767	0.6901	0.0680
S12_19340454	12	19340454	S12_19340479	1	0.025	0.3497	0.9997	0.0731	0.4570	0.0589
S12_19671851	12	19671851	S12_19671939	1	0.088	0.0018	1.0000	0.0021	1.0000	-0.0018
S12_11957639	12	11957639	S12_11957641	1	0.002	0.0000	0.0000	0.0000	0.0000	0.0000
S12_11957641	12	11957641	S12_11957682	1	0.041	0.0000	0.0000	0.0000	0.0000	0.0000
S12_11957682	12	11957682	S12_11957685	1	0.003	0.0000	0.0000	0.0000	0.0000	0.0000
