# Supplementary material for: Genome-Wide Differentiation of Various Melon Horticultural Groups for Use in GWAS for Fruit Firmness and Construction of a High Resolution Genetic Map
Source: Front Plant Sci. 2016 Sep 22;7:1437. doi: 10.3389/fpls.2016.01437 (PMC5031849; doi:10.3389/fpls.2016.01437)
Supplement: Table S7 — Linkage disequilibrium (LD) analysis for adjacent SNP pairs across melon genome. [file Table7.pdf]

Table S7: Chromosome-wide distribution of loci skewed toward female and male parent

	Parent A	Parent B	Total number of distorted markers
chr1	14	31	53
chr2	2	21	34
chr3	6	45	63
chr4	18	42	75
chr5	54	3	66
chr6	31	20	72
chr7	42	3	45
chr8	21	22	57
chr9	3	33	53
chr10	5	11	19
chr11	42	7	50
chr12	32	10	45
